# Mechanism, reactivity, and selectivity of the iridium-catalyzed C(sp^3^)–H borylation of chlorosilanes†
†Electronic supplementary information (ESI) available: Additional results not shown in the text, table of calculated energies, and Cartesian coordinates of all optimized structures discussed in the paper. See DOI: 10.1039/c4sc01592d



**DOI:** 10.1039/c4sc01592d

**Published:** 2014-12-04

**Authors:** Genping Huang, Marcin Kalek, Rong-Zhen Liao, Fahmi Himo

**Affiliations:** a Department of Organic Chemistry , Arrhenius Laboratory , Stockholm University , SE-106 91 Stockholm , Sweden . Email: himo@organ.su.se

## Abstract

The iridium-catalyzed C(sp^3^)–H borylation of methylchlorosilanes is investigated by means of density functional theory, using the B3LYP and M06 functionals. The calculations establish that the resting state of the catalyst is a seven-coordinate Ir(v) species that has to be converted into an Ir(iii)tris(boryl) complex in order to effect the oxidative addition of the C–H bond. This is then followed by a C–B reductive elimination to yield the borylated product, and the catalytic cycle is finally completed by the regeneration of the active catalyst over two facile steps. The two employed functionals give somewhat different conclusions concerning the nature of the rate-determining step, and whether reductive elimination occurs directly or after a prior isomerization of the Ir(v) hydride intermediate complex. The calculations reproduce quite well the experimentally-observed trends in the reactivities of substrates with different substituents. It is demonstrated that the reactivity can be correlated to the Ir–C bond dissociation energies of the corresponding Ir(v) hydride intermediates. The effect of the chlorosilyl group is identified to originate from the α-carbanion-stabilizing effect of the silicon, which is further reinforced by the presence of an electron-withdrawing chlorine substituent. Furthermore, the source of selectivity for the borylation of primary over secondary C(sp^3^)–H can be explained on a steric basis, by repulsion between the alkyl group and the Ir/ligand moiety. Finally, the difference in the reactivity between C(sp^3^)–H and C(sp^2^)–H borylation is investigated and rationalized in terms of distortion/interaction analysis.

## Abstract 2

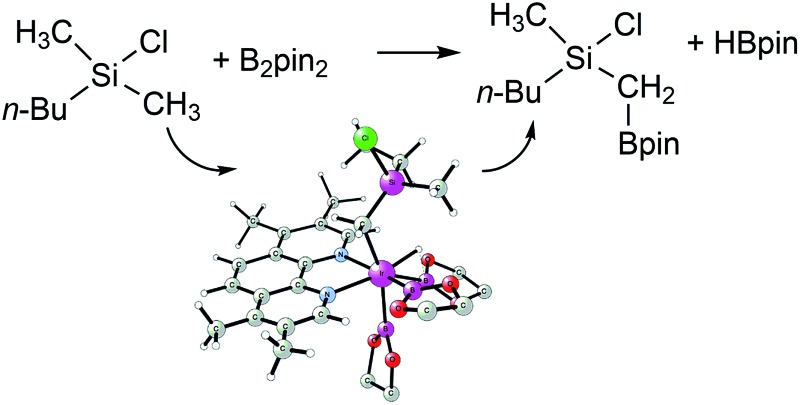
DFT calculations are used to elucidate the reaction mechanism, the role of the chlorosilyl group, and primary *vs.* secondary and C(sp^3^)–H *vs.* C(sp^2^)–H selectivity of the iridium-catalyzed borylation of chlorosilanes.

## 1. Introduction

Transition metal-catalyzed C–H functionalizations are today some of the most pursued chemical transformations. Despite the tremendous progress in this area during the last ten years,^1^ development of these types of reactions remains a central challenge in organic chemistry. In particular, the activation of C(sp^3^)–H bonds with high efficiency and selectivity represents an important and long-standing goal.

A number of approaches have been developed to achieve selective functionalization of a single C(sp^3^)–H bond in a molecule containing several CH_*n*_ groups. Probably the most widespread strategy relies on the use of a coordinating moiety incorporated into the structure of the starting material that ligates the metal catalyst and thus directs the C–H cleavage.^2^ The selectivity of the C–H cleavage may also be governed by electronic effects. In this case a functional group present in the substrate molecule activates a certain C–H bond for the reaction with the metal. Classical examples of this approach are α-functionalizations of carbonyl compounds,^3^ and allylic^4^ and benzylic^5^ C–H functionalizations. Furthermore, some methods allow for the selective activation of C–H bonds based on steric criteria. Reactions occurring favorably at less hindered methyl groups^6^ and those preferentially functionalizing tertiary C–H bonds have been developed.^7^

**Scheme 1:**
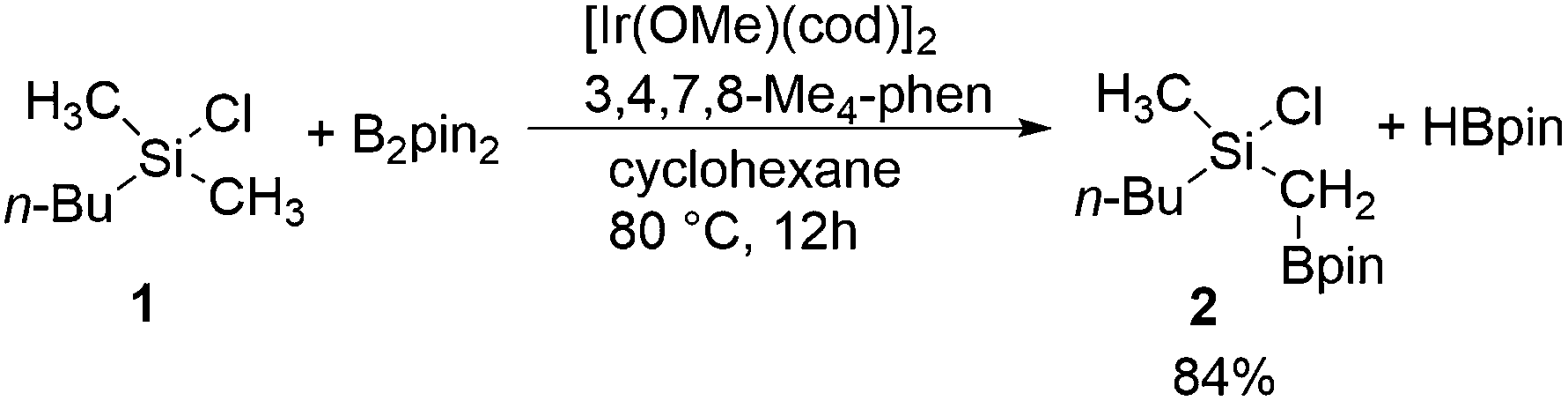
Iridium-catalyzed C(sp^3^)–H borylation of methylchlorosilane **1** (pin = (–OCMe_2_)_2_).^8^

Especially useful from a synthetic point of view are methods for C–H functionalization in which the employed directing group itself can be used in subsequent transformations. This way, a rapid increase of molecular complexity is possible during the synthesis, which in turn allows for the shortening of synthetic routes. In this context, an excellent contribution was recently made by Suginome and coworkers, who reported an iridium-catalyzed C(sp^3^)–H borylation of chlorosilanes (Scheme 1).^8^ C–H activation for the construction of the C–B bond has been achieved using a number of catalytic systems,^9,10^ but the novelty of the reaction shown in Scheme 1 stems from the fact that it makes use of a novel directing group for controlling the reactivity and selectivity. Namely, the reaction takes place at the methyl group adjacent to a chlorosilyl moiety, thus generating compounds containing 1-silyl-1-boryl-substituted methylene.^11^ Both the boronate functionality introduced in the reaction and the silyl group are synthetically very useful,^10^ as they can be employed in, for instance, Suzuki^12^ and Hiyama^13^ couplings, respectively. Since the silyl substituent is compatible with the conditions of the Suzuki reaction,^14^ a sequential application of these transformations opens access to non-symmetrical *gem*-difunctionalized methylenes.

Experimentally, it has been established that the reaction in Scheme 1 takes place selectively at the primary methyl C–H bond on the silicon atom, without a secondary borylation product being observed.^8^ Importantly, it was found that the presence of the chlorosilyl group is crucial for achieving a good reactivity under the mild reaction conditions used. Namely, no borylation product could be observed upon the replacement of the silicon by a carbon. Also, when changing the chlorine substituent to a methyl group, a much higher reaction temperature had to be applied in order to observe the formation of product.^15^ Moreover, replacing the chlorine by other substituents, such as chloromethyl or methoxy groups, led to a considerable decrease in the product yield.^8^ Based on these observations, it was proposed that the chlorine functions as a directing group by coordination to iridium or preferably to one of the boron atoms in the active species [(3,4,7,8-Me_4_-phen)Ir(Bpin)_3_] (3,4,7,8-Me_4_-phen = 3,4,7,8-tetramethylphenanthroline), thus positioning the reactant for a facile C–H activation.^8^

Due to the significance of this transformation, it is important to elucidate the detailed reaction mechanism, including the origins of the observed reactivity and selectivity. Although the C(sp^2^)–H borylation reaction has been studied extensively, both experimentally and theoretically,^16,17^ mechanistic insights into C(sp^3^)–H borylation remain scarce.^18,19^ Understanding the mechanism, especially the role of the chlorosilyl group, could have important implications for the future design of new catalytic systems for C–H functionalization. Therefore, we decided to investigate the details of the reaction mechanism using density functional theory (DFT) calculations. The generally accepted mechanism for Ir-catalyzed C(sp^2^)–H borylation involves Ir(iii)/Ir(v) intermediates and consists of three steps: C–H oxidative addition, C–B reductive elimination, and regeneration of active catalyst.

In a very recent paper, Hartwig and coworkers used DFT calculations to investigate the mechanism and regioselectivity of the C(sp^3^)–H borylation of alkylamines and alkyl ethers using (η^6^-mes)Ir(Bpin)_3_ (mes = mesitylene) and the 3,4,7,8-Me_4_-phen ligand, *i.e.* involving the same catalytically active species that is engaged in the reaction studied herein.^19^ The mechanism was found to be very similar to the C(sp^2^)–H borylation. These results are very relevant to the current study and the similarities and differences compared to our findings will be discussed in the appropriate sections below.

## 2. Computational details

All the calculations were performed using the Gaussian 09 package.^20^ Geometry optimizations were carried out using the B3LYP functional^21^ with a combined basis set, in which Ir was described by LANL2DZ,^22^ and 6-31G(d,p) was used for all other atoms. Frequencies were computed analytically at the same level of theory to confirm whether the structures are minima (no imaginary frequencies) or transition states (only one imaginary frequency), and also to calculate the free energy corrections. Solvation effects were evaluated by performing single-point calculations with the polarizable continuum model (PCM).^23^ The parameters for cyclohexane (*ε* = 2.0165), corresponding to the experimental conditions, and UFF atomic radii were used in these calculations.

To obtain better accuracy, single-point energies for the optimized geometries were recalculated using both the B3LYP and M06 functionals with a larger basis set, which is LANL2TZ(f) for Ir and 6-311+G(2d,2p) for all other atoms. The free energies reported in the article (Δ*G*_sol_) are the large basis set single-point energies corrected by a gas-phase Gibbs free energy correction (at 298.15 K) and solvation correction. For B3LYP, a dispersion correction using the D3 method developed by Grimme was added.^24^

In the reactivity analysis, the bond dissociation energies (BDEs) were calculated in a similar manner but including only the zero-point energy part of the Gibbs free energy correction.

It is important to point out that for each stationary point discussed in the paper there exists a number of rotamers of both the substrate and ligands. A thorough conformational analysis has therefore been performed in order to ensure that the lowest energy conformation is used.

## 3. Results and discussion

**Scheme 2:**
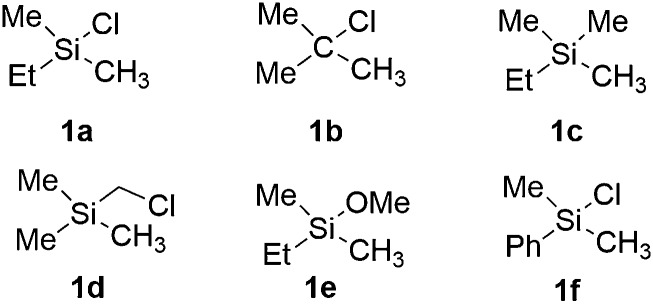
Model substrates investigated in the current study.

In this section, we first focus on the primary C(sp^3^)–H borylation of the model substrate EtMe_2_SiCl **1a** (Scheme 2) and evaluate the mechanism step by step. The results calculated with both B3LYP and M06 are presented. Some details regarding the mechanism turned out to be somewhat different using the two methods and this will be discussed in detail below. Next, the energy profiles for substrates **1b–1e** are calculated and compared with that for **1a** in order to elucidate the origin of the observed reactivity differences. Then, the secondary C(sp^3^)–H borylation of **1a** is studied and the 1° *vs.* 2° regioselectivity is rationalized. Finally, we present a comparison of the C(sp^2^)–H and C(sp^3^)–H borylation of substrate **1f** and analyze the origins of the observed selectivity.

### 3.1. Results with B3LYP

**Scheme 3:**
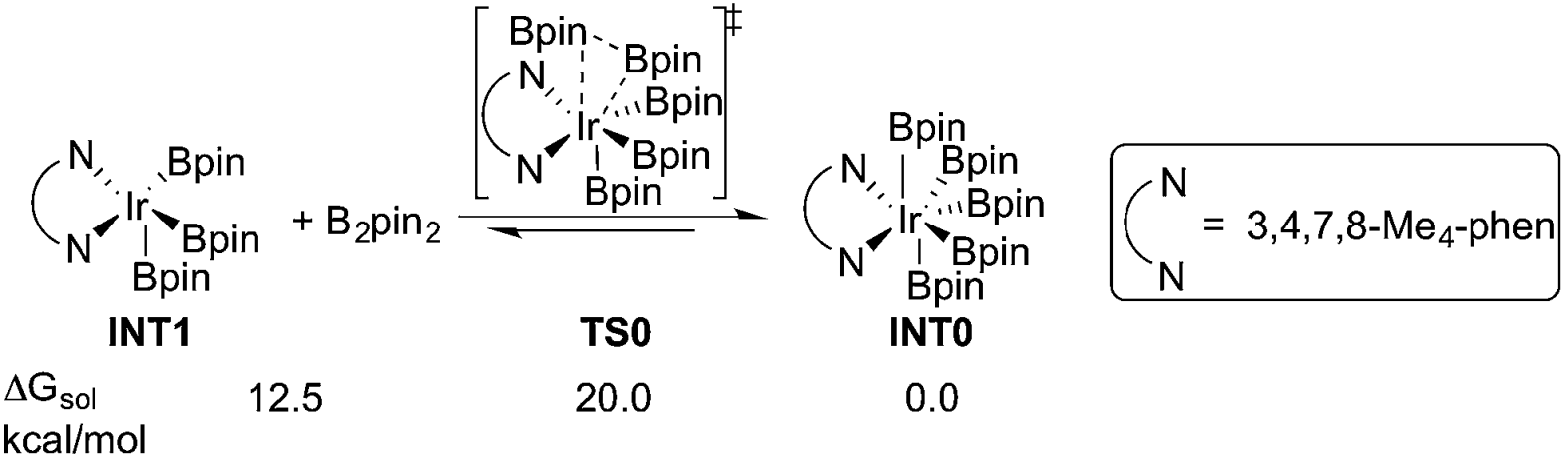
Energetics of the reaction between **INT1** and B_2_pin_2_.

Experimentally, the iridium(i) complex [Ir(OMe)(cod)]_2_ (cod = 1,5-cyclooctadiene) is used as the catalyst precursor, and 3,4,7,8-Me_4_-phen as the ligand.^8^ It has been suggested that under the reaction conditions, the pre-catalyst undergoes an initiation process to generate an Ir(iii)tris(boryl) complex, being the active catalytic species.^16a,17,19^ Thus, in the current calculations, we use **INT1** (Scheme 3) as the starting point, in line with the previous studies.^16a,17,19^

**INT1** is a 16-electron complex that is able to effect the C–H bond cleavage. However, as discussed previously,^16*a*^ it may also react with another molecule of B_2_pin_2_ to generate a seven-coordinate 18-electron Ir(v)-species **INT0**. The transformation of **INT1** into **INT0** is calculated to be exergonic by 12.5 kcal mol^–1^ and the energy of the transition state (**TS0**) is 7.5 kcal mol^–1^ higher than that of **INT1** (Scheme 3). These results regarding the stability of the Ir(v) species are consistent with the previous findings in the case of C(sp^2^)–H borylation.^16*a*^ As will be shown below, the coordinately saturated 18-electron Ir(v) complex **INT0** is in fact the lowest lying intermediate, and thus constitutes the resting state of the catalyst.

In order for the reaction to take place, **INT0** must thus first be converted into **INT1** by a reversible reductive elimination of B_2_pin_2_. Therefore, we set the sum of the free energies of complex **INT0** and the substrate to zero on the relative free energy scale.

**Fig. 1:**
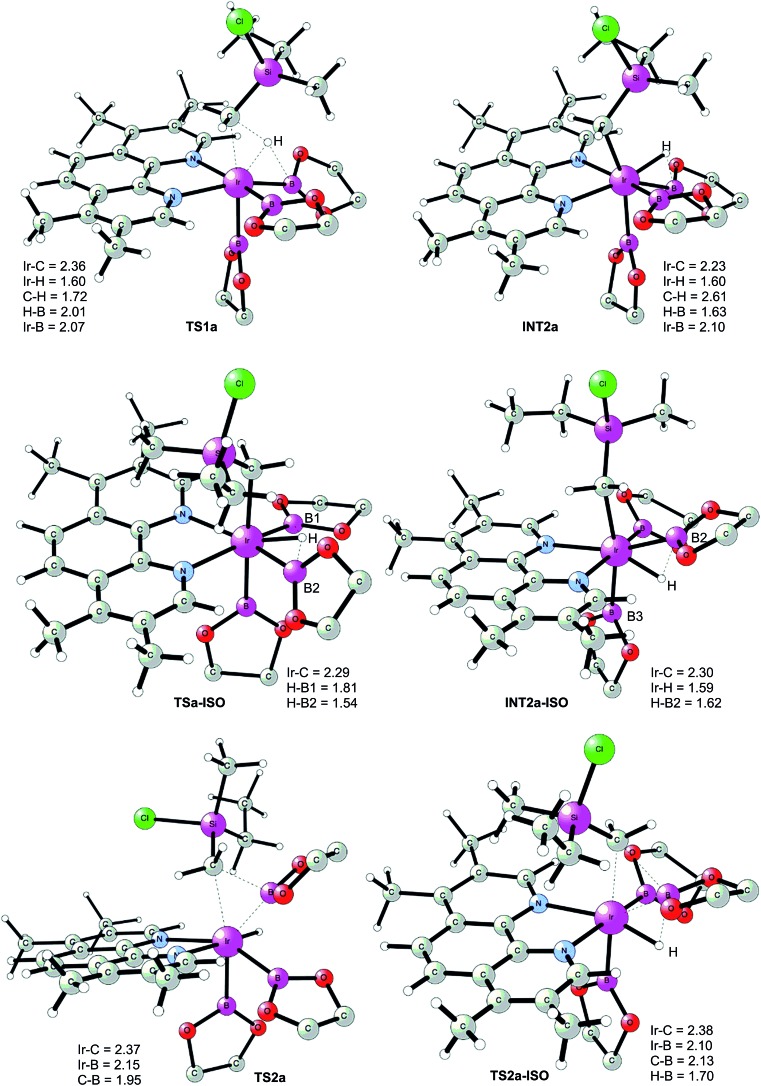
Optimized structures of stationary points for primary C–H borylation of **1a** (distances are given in Å). The methyl groups on the Bpin ligands are omitted for clarity.

**Fig. 2:**
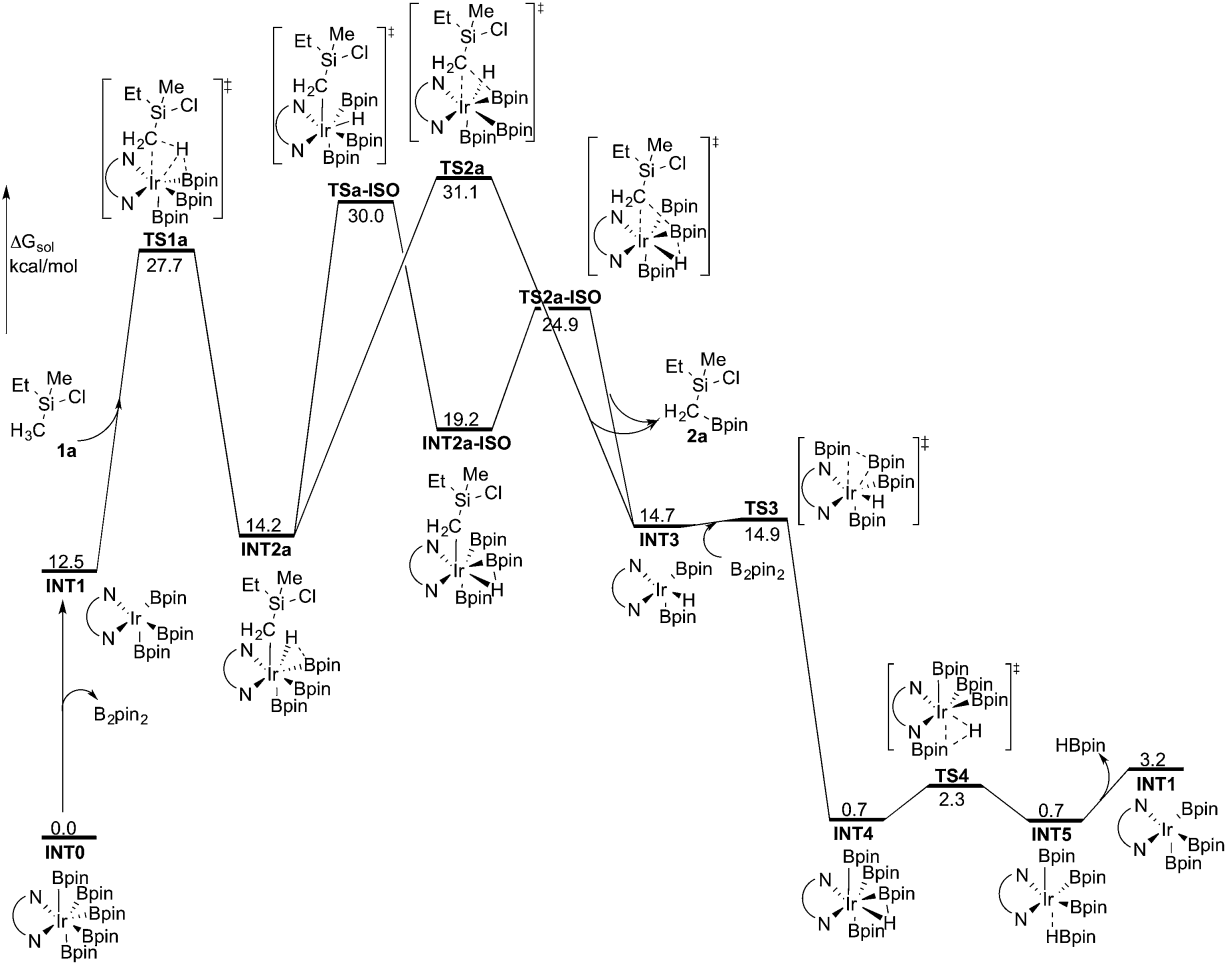
Overall energy profile calculated at the B3LYP-D3 level of theory for the Ir-catalyzed primary C(sp^3^)–H borylation of **1a**.

The first step of the mechanism is the oxidative addition of the C–H bond in the methyl group of **1a** to the Ir(iii) complex **INT1**. As mentioned in the introduction, it has been suggested that the C–H oxidative addition might be assisted by the coordination of the chlorine atom to the iridium or to the boron atom.^8^ However, such a scenario could not be confirmed by the calculations, as no coordination of the chlorine atom to the iridium complex could be observed, neither in the pre-complex nor in the C–H oxidative addition transition state. Instead, the calculations always converged to transition state **TS1a**, in which there is no direct interaction between the chlorine and the boron or the iridium (Fig. 1). The energy barrier is calculated to be 15.2 kcal mol^–1^ relative to **INT1**, *i.e.* 27.7 kcal mol^–1^ relative to **INT0** (see Fig. 2 for energy profile).^25^

The C(sp^3^)–H oxidative addition transition state **TS1a** shows many similarities to transition states for C(sp^2^)–H oxidative additions reported before.^16a,17^ Importantly, the previously-observed multi-center interactions between the Ir, H, C, and B atoms are also found in **TS1a**, as seen from the C–H, Ir–H, Ir–C, and H–B distances being 1.72, 1.60, 2.36, and 2.01 Å, respectively (Fig. 1). This indicates that the interaction of the empty p orbital of boron with the hydride is important for a facile C–H oxidative addition to the iridium center.^16a,18b,c,19,26^

The oxidative addition is calculated to be endergonic by 1.7 kcal mol^–1^ relative to **INT1** (14.2 kcal mol^–1^ relative to **INT0**, Fig. 2) and the resulting intermediate **INT2a** is a seven-coordinated Ir(v) hydride complex, which has a capped octahedron configuration.^27^ Importantly, an even stronger interaction between the hydride ligand and the boron center is found in **INT2a** compared to **TS1a** (H–B distance 1.63 *vs.* 2.01 Å).

Upon formation of **INT2a**, the subsequent step of the reaction is a C–B bond-forming reductive elimination, as in the case of the C(sp^2^)–H borylation.^16*a*^ From **INT2a** the energy barrier for the reductive elimination *via* **TS2a** was calculated to be 31.1 kcal mol^–1^ relative to **INT0**, *i.e.* 3.4 kcal mol^–1^ higher than that for the preceding **TS1a** for the oxidative addition.^28^ This is different from the case of the C(sp^2^)–B reductive elimination, for which previous calculations have shown that it is very facile, with a barrier lower than for the preceding C(sp^2^)–H oxidative addition.^16*a*^ It is also different from the results of Hartwig and coworkers on the Ir-catalyzed C(sp^3^)–H borylation of triethylamine, where it was found that the transition state for the reductive elimination is 6.4 kcal mol^–1^ lower in energy than the transition state for the oxidative addition.^19^

This result was somewhat surprising and we therefore examined alternative pathways for this process, involving an initial ligand rearrangement in **INT2a**. Indeed, we found that the reductive elimination is more favorable from an isomeric Ir(v) complex **INT2a-ISO**, in which the hydride ligand occupies a coordination site adjacent to the B2 and B3 ligands, instead of the one adjacent to the alkyl group and B1 (see Fig. 1). **INT2a-ISO** is 5.0 kcal mol^–1^ higher in energy than **INT2a**, but the transition state for the reductive elimination from **INT2a-ISO** (**TS2a-ISO**) is 6.2 kcal mol^–1^ lower in energy than **TS2a** (Fig. 2), *i.e.* 24.9 kcal mol^–1^ relative to **INT0**. Comparison of the structures of **TS2a** and **TS2a-ISO** identifies two factors that may be responsible for the energy difference between these transition states. First, in **TS2a-ISO** the interaction between the hydride ligand and the empty p orbital of the B atom of one of the boryl ligands, which is present in both intermediates **INT2a** and **INT2a-ISO**, is maintained (H–B distance 1.70 Å, Fig. 1), while in **TS2a** it has to be broken in order for the reductive elimination to occur. Second, there is some steric repulsion between the two Bpin groups in **TS2a**, which can contribute to raising the energy of this transition state.

Nevertheless, although the C–B bond formation *via* **TS2a-ISO** is facile, the prior isomerization of **INT2a** into **INT2a-ISO** has to take place. The isomerization was found to occur *via* transition state **TSa-ISO**, which has a pentagonal-bipyramidal configuration,^27,29^ in which the hydride ligand is practically coplanar with the 3,4,7,8-Me_4_-phen and boryl ligands, and it interacts with two boron centers simultaneously (B–H distances 1.81 and 1.54 Å).^30^ **TSa-ISO** is calculated to be 15.8 kcal mol^–1^ higher in energy than **INT2a**, *i.e.* 30.0 kcal mol^–1^ higher in energy than **INT0**. The energy barrier is thus only 1.1 kcal mol^–1^ lower than that of **TS2a**, and it is therefore not possible on the basis of these calculations to conclude which one of these two options is the favored path for the reductive elimination step.

The reductive elimination results in the final reaction product **2a** and the Ir(iii) complex **INT3**, which is 0.5 kcal mol^–1^ higher in energy than **INT2a** (Fig. 2). The catalytic cycle is completed by the regeneration of active catalyst **INT1**. This process consists of two steps, namely an oxidative addition of B_2_pin_2_ to **INT3** *via* **TS3**, followed by a reductive elimination of HBpin (**TS4**). The calculations show that both steps are very facile, with very low energy barriers, as shown in Fig. 2 (see ESI† for optimized structures of the TSs).

Here, it is important to emphasize that the proposed coordination of the silicon-bound chlorine atom to the catalyst could not be observed in our calculations at any stage of the mechanism. Therefore, such a scenario is apparently not responsible for the observed reactivity and selectivity of the reaction. A detailed investigation of these issues will be presented below.

### 3.2. Results with M06

**Fig. 3:**
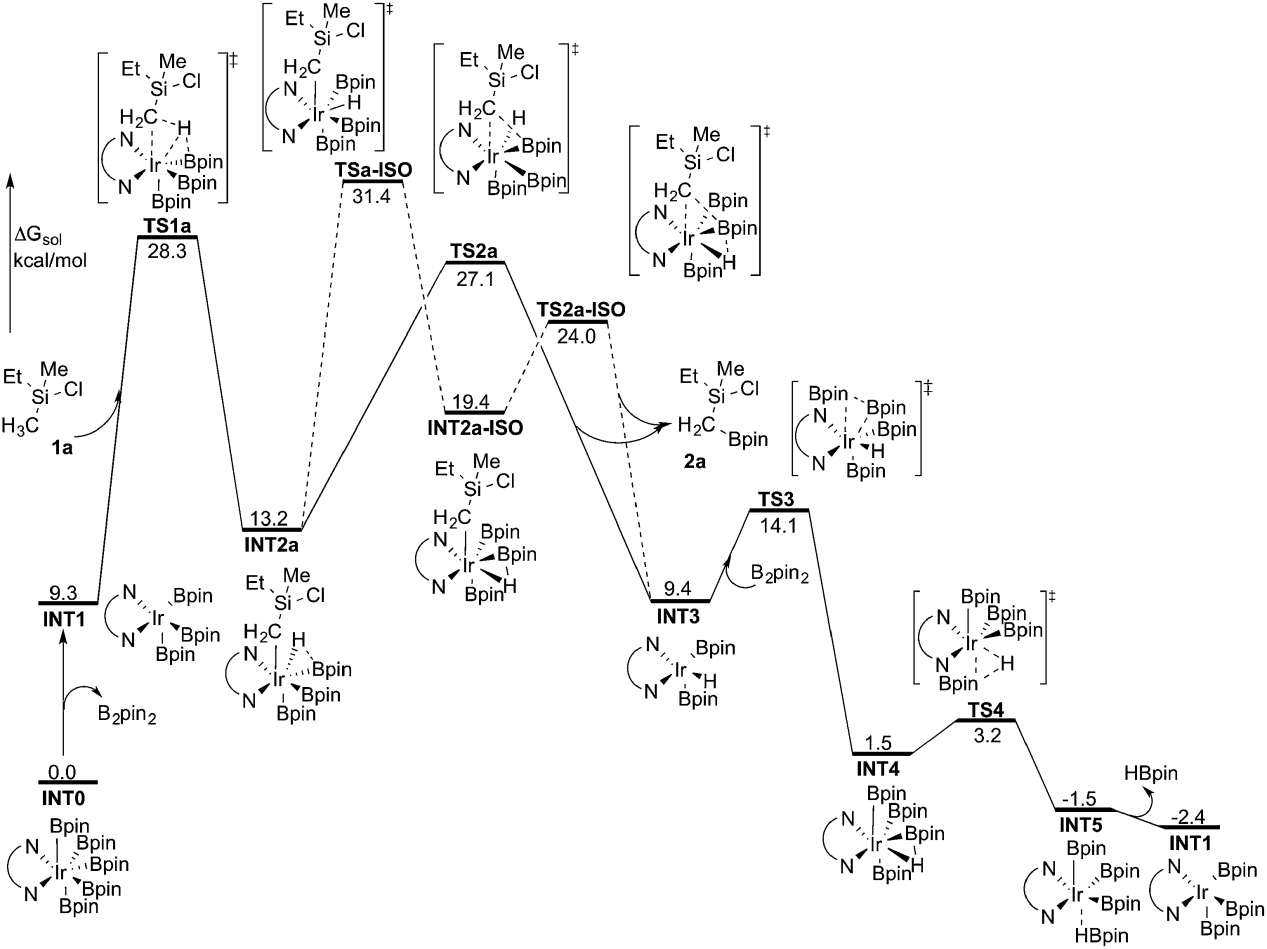
Overall energy profile calculated at the M06 level of theory for the Ir-catalyzed primary C(sp^3^)–H borylation of **1a**.

As discussed above, the current results are somewhat different compared with the recent results of Hartwig and coworkers, where they used the M06 functional to study the borylation of alkylamines.^19^ To investigate whether this discrepancy is due to the employed functional, we recalculated the free energy graph using the M06 method by performing single-point calculations on the B3LYP-optimized geometries (see Fig. 3 for the calculated free energy profile). We also performed test geometry optimizations with M06, but the results were very similar, within 1 kcal mol^–1^.

Indeed, the calculations show that the energy profile changes depending on the functional employed. A significant change is the energy difference between **TSa-ISO** and **TS2a**. In the B3LYP calculations, the energy of **TS2a** was 1.1 kcal mol^–1^ higher than that of **TSa-ISO**, whereas in the M06 calculations, the energy of **TS2a** is 4.3 kcal mol^–1^ lower than that of **TSa-ISO**. This indicates that the direct C–B reductive elimination pathway would be more favored compared to the isomerization step. Another difference compared to the B3LYP calculations is that the C–H oxidative addition with M06 turns out to be the highest point of the overall energy profile, as **TS2a** is calculated to be 1.2 kcal mol^–1^ lower in energy than **TS1a**. This result is more consistent with the calculations on the borylation of triethylamine.^19^ However, since the energy difference between oxidative addition and direct reductive elimination is rather small (only 1.2 kcal mol^–1^), and the results calculated using the B3LYP and M06 functionals point in somewhat different directions regarding the nature of the rate-determining step, it is not possible to draw definitive conclusions regarding this matter on the basis of the current calculations.

Nevertheless, taken together, both the B3LYP and M06 functionals give a quite similar overall picture of the reaction mechanism. Namely, both predict the resting state to be the seven-coordinate Ir(v) complex **INT0**. This saturated complex has to convert into the active catalyst **INT1** in order to perform the C–H oxidative addition, which is then followed by C–B reductive elimination and regeneration of the active catalyst. The B3LYP results suggest that an isomerization step is needed prior to the C–B reductive elimination, while M06 suggests that the direct reductive elimination is preferred. It should also be noted that the energy difference between the oxidative addition and reductive elimination steps is rather small for both methods and falls within the accuracy limits.

### 3.3. Kinetic isotope effect

An important test of the validity of a calculated mechanism is whether it complies with the results of kinetic isotope effect (KIE) experiments. In the experimental report, when the substrate *n*-C_6_H_13_(CH_3_)(CD_3_)SiCl, containing one of the methyl groups labeled with deuterium, was used in the reaction, a 2.7 ratio of C–H : C–D functionalization products was obtained.^8^

To examine the KIE for the calculated mechanism we evaluated the zero-point energies (ZPE) with the deuterium incorporated in the appropriate positions according to the experiment (by using the deuterated **1a** Et(CH_3_)(CD_3_)SiCl). For the oxidative addition transition state **TS1a**, the calculated ZPE difference is 0.78 kcal mol^–1^, corresponding to a normal KIE of 3.0 at the temperature used in the experiment (80 °C), which agrees very well with the experimentally-observed KIE. For the direct reductive elimination transition state **TS2a**, on the other hand, the calculated ZPE difference is only 0.23 kcal mol^–1^, corresponding to a KIE of only 1.4.

Interestingly, the ZPE difference for **TSa-ISO** was calculated to be 0.45 kcal mol^–1^, corresponding to a KIE of 1.9, which is also in reasonable agreement with the experimental value. This KIE originates mainly from the equilibrium isotope effect between **INT1** and **INT2a**,^31^ since the calculated the ZPE difference for **INT2a** was 0.37 kcal mol^–1^, corresponding to a KIE value of 1.7.

The fact that the calculated KIE for **TS1a** agrees better with the experimental value as compared to **TSa-ISO** indicates that it is more likely that the oxidative addition is the rate-determining step, which would support the M06 results. However, considering that the values are quite close, a definitive assignment cannot be done on the basis of these results solely.

### 3.4. Reactivity of other substrates

Considering that there is no interaction between the chlorine atom and iridium or boron at any stage of the mechanism, the results presented above raise the question of how the chlorosilyl group facilitates the reaction. To rationalize this effect, we calculated the energy profiles for the borylation of substrates **1b–e** (Scheme 2), at both the B3LYP and M06 levels. The energies are listed in Table 1.

**Table 1:** Calculated energies (kcal mol^–1^) for the Ir-catalyzed C(sp^3^)–H borylation of substrates **1a–e**. For each substrate, the energies are relative to (**INT0** + substrate)

Substrate	**TS1**	**INT2**	**TS2**	**TS-ISO**	**INT2-ISO**	**TS2-ISO**	**INT3**
**B3LYP-D3**							
**1a**	27.7	14.2	31.1	30.0	19.2	24.9	14.7
**1b**	31.1	18.2	40.8	36.0	23.5	30.0	18.6
**1c**	30.7	19.2	36.4	36.7	25.8	30.4	14.5
**1d**	28.6	15.1	34.1	33.2	22.6	25.2	14.5
**1e**	29.7	17.1	33.7	32.4	22.1	25.1	14.5

**M06**							
**1a**	28.3	13.2	27.1	31.4	19.4	24.0	9.4
**1b**	31.0	17.0	36.7	36.9	23.0	27.7	13.1
**1c**	30.9	17.7	32.5	37.6	26.2	29.1	8.9
**1d**	28.8	13.7	30.5	34.6	23.4	24.4	9.1
**1e**	30.0	15.9	30.5	33.7	23.6	24.0	8.9

For the B3LYP calculations, the results show that in all cases except for substrate **1c**, the barriers of isomerization (**TS-ISO**) are slightly lower than those of direct C–B reductive elimination (**TS2**). For **1c**, the direct C–B reductive elimination was found to be slightly favored, by only 0.3 kcal mol^–1^. Moreover, the barriers of isomerization are significantly higher than those of C–H oxidative addition, showing that the former is rate-determining. The results are thus in line with the energy profile for **1a** using B3LYP.

For the M06 calculations, the direct C–B reductive elimination was found to be more favorable compared to the isomerization in all cases, in line with the results for substrate **1a**. Very importantly, however, the reductive elimination step is found to be rate-determining for substrates **1b–e**, which is different from the case of substrate **1a**. One contributing factor for this could be the increased steric repulsion between the C and B groups that undergo direct C–B reductive elimination, as compared to the steric repulsion for the C–H oxidative addition (see structures of optimized transition states **TS2a–e** in ESI†).

Generally, both M06 and B3LYP calculations reproduce quite well the experimental trends in the relative reactivity of the different substrates (Table 1). Here, we will use the M06 results to compare and rationalize the reactivity. Note that, since no detailed kinetic experiments have been conducted, the comparison with the experimental results is only qualitative.

For substrate **1b**, in which the silicon atom is replaced by a carbon atom, the overall energy barrier was calculated to be 36.7 kcal mol^–1^ (**TS2b**), which is 8.4 kcal mol^–1^ higher than that for substrate **1a**. This is consistent with the fact that no desired product formation was observed experimentally for **1b**.

Experimentally, the Cl substituent on silicon was found to be very important for the reactivity. In the original report, no reaction was observed with substrate *n*-C_8_H_17_SiMe_3_.^8^ However, as described in a subsequent article, the application of a much higher temperature led to the formation of the desired product for this substrate.^15^ The calculations show that the energy barrier for the reaction with model substrate **1c** is 32.5 kcal mol^–1^ (**TS2c**), which is 4.2 kcal mol^–1^ higher than for **1a**, thus in agreement with the experiments.

The reaction with **1d**, in which the chlorine is substituted for a chloromethyl group and for which the product is formed in only a 20% yield experimentally, was calculated to have an overall barrier of 30.5 kcal mol^–1^ (**TS2d**), *i.e.* only 2.2 kcal mol^–1^ higher than for **1a**.

Finally, the reaction with model substrate **1e**, in which the Cl atom is replaced by a methoxide group, was also investigated and the results show that the energy barrier is 2.2 kcal mol^–1^ higher than that for **1a**. This is qualitatively consistent with the experimentally observed yield of less than 5% for substrate *n*-BuMe_2_Si(OMe).

**Scheme 4:**
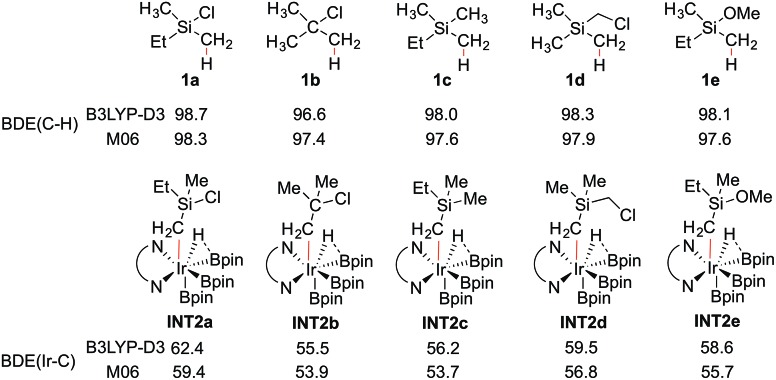
Calculated BDEs (kcal mol^–1^) of the C–H and Ir–C bonds.

To shed light on the origin of the observed reactivity difference, the homolytic bond dissociation energies (BDEs) of the C–H bonds of substrates **1a–e**, and the Ir–C bonds of intermediates **INT2a–e** were calculated (Scheme 4). Interestingly, the results show that the C–H BDEs of substrates containing a Si atom are almost identical, with energy differences within 0.7 kcal mol^–1^. The C–H BDE of **1b** is the lowest, which does not correlate with the observed low reactivity of this substrate. In contrast, the Ir–C BDE in **INT2** was found to be more sensitive to the substituent of the alkyl group, ranging from 53.7 to 59.4 kcal mol^–1^. Importantly, we find that there is a good correlation between the calculated barriers of the C–H oxidative addition step and the Ir–C BDEs (see ESI†). That is, the stronger the Ir–C bond, the lower the energy barrier. A similar trend can also be observed for the direct C–B reductive elimination step. These correlations are found for both the M06 and B3LYP functionals.

The trends of reactivity can thus be predicted by the stability of the Ir–C bond in **INT2**. The results of the current study on C(sp^3^)–H borylation are consistent with the results of Houk and coworkers, who very recently showed that in the Ir-catalyzed C(sp^2^)–H borylation, the Ir–C BDEs of the aryl iridium hydride intermediates, resulting from the C–H oxidative addition step, can be used to predict the regioselectivity of the reaction.^32^

In general, it has been found that the BDEs of M–C bonds correlate quite well with the M–C bond ionicity, where an increased bond ionicity leads to a larger BDE.^33^ Thus, an electron-withdrawing substituent on the alkyl group can polarize the Ir–C bond, which results in a higher BDE. In the current study, it is found that the Ir–C BDE of **INT2a**, with a Si atom, is higher than that of **INT2b**, with a C atom. The reason is that the Si atom is well-known to stabilize the negative charge at the α position (the so-called α-effect),^34^ which thus makes the Ir–C bond stronger. Furthermore, comparison of the Ir–C BDEs of **INT2a** *vs.* **INT2c–e** indicates that the presence of the electron-withdrawing Cl substituent on the Si atom further reinforces this stabilization effect. This suggests that other substituents at the Si with even stronger electron-withdrawing ability could lead to an increased reactivity, although the balance between the electronic and steric effects must also be considered. It should be noted that an analogous dependence of reactivity on the stabilization of negative charge on the arene ligand was recently found for the related iridium-catalyzed C(sp^2^)–H borylation.^17^

### 3.5. Primary *vs.* secondary C(sp^3^)–H borylation

**Fig. 4:**
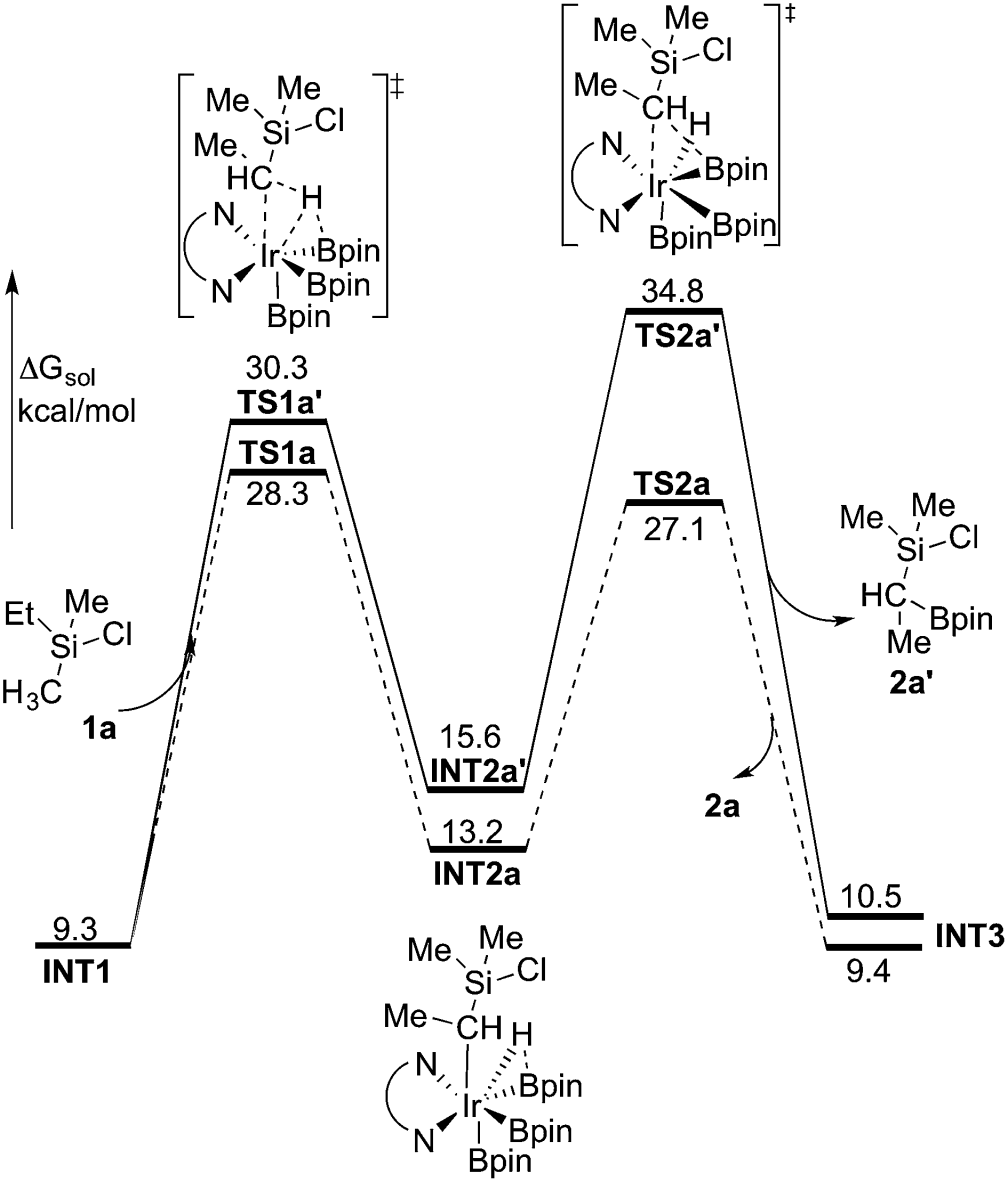
Energy profile calculated at the M06 level of theory for the Ir-catalyzed secondary C(sp^3^)–H borylation of **1a** (solid line). The primary C(sp^3^)–H borylation pathway is also shown for comparison (dashed line).

We now turn to the issue of the selective borylation of the primary position in the presence of secondary C(sp^3^)–hydrogens. Experimentally, it was shown that the reaction did not take place for substrates such as Et_3_SiCl, *i.e.* without a methyl group on silicon.^8^ To shed light on the origin of this selectivity, we calculated the full free energy profile for the C(sp^3^)–H borylation at the secondary position in substrate **1a** (Fig. 4, solid line). Both M06 and B3LYP results reproduced well the observed selectivity. Here, the M06 results will be presented, and the B3LYP results are given in the ESI.†

**Fig. 5:**
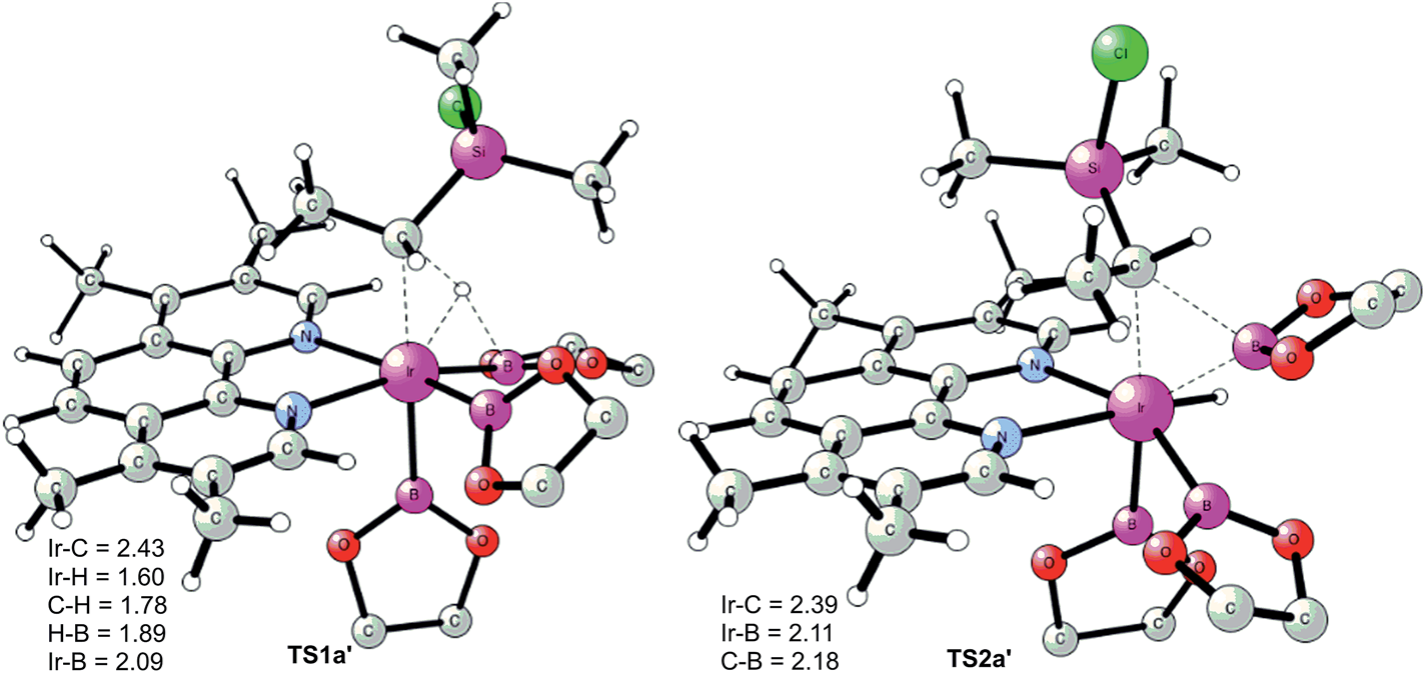
Optimized transition state structures for borylation at the secondary position.

The calculations show that for the secondary C(sp^3^)–H borylation the direct C–B reductive elimination (**TS2a′**) is the rate-determining step, with an overall energy barrier of 34.8 kcal mol^–1^ relative to **INT0** (Fig. 4). The activation energy difference of 6.5 kcal mol^–1^ between the primary and secondary borylation thus reproduces the experimentally observed exclusive formation of product **2a**. Inspection of the optimized structures (Fig. 5) indicates that the selectivity is mainly caused by the steric repulsion between the alkyl group and the Ir/ligand moiety in **TS2a′**. A similar steric origin of the selectivity has been proposed for the Rh-catalyzed C(sp^3^)–H borylation of terminal methyl groups of unactivated alkanes.^18*a*^

### 3.6. C(sp^3^)–H *vs.* C(sp^2^)–H borylation

**Fig. 6:**
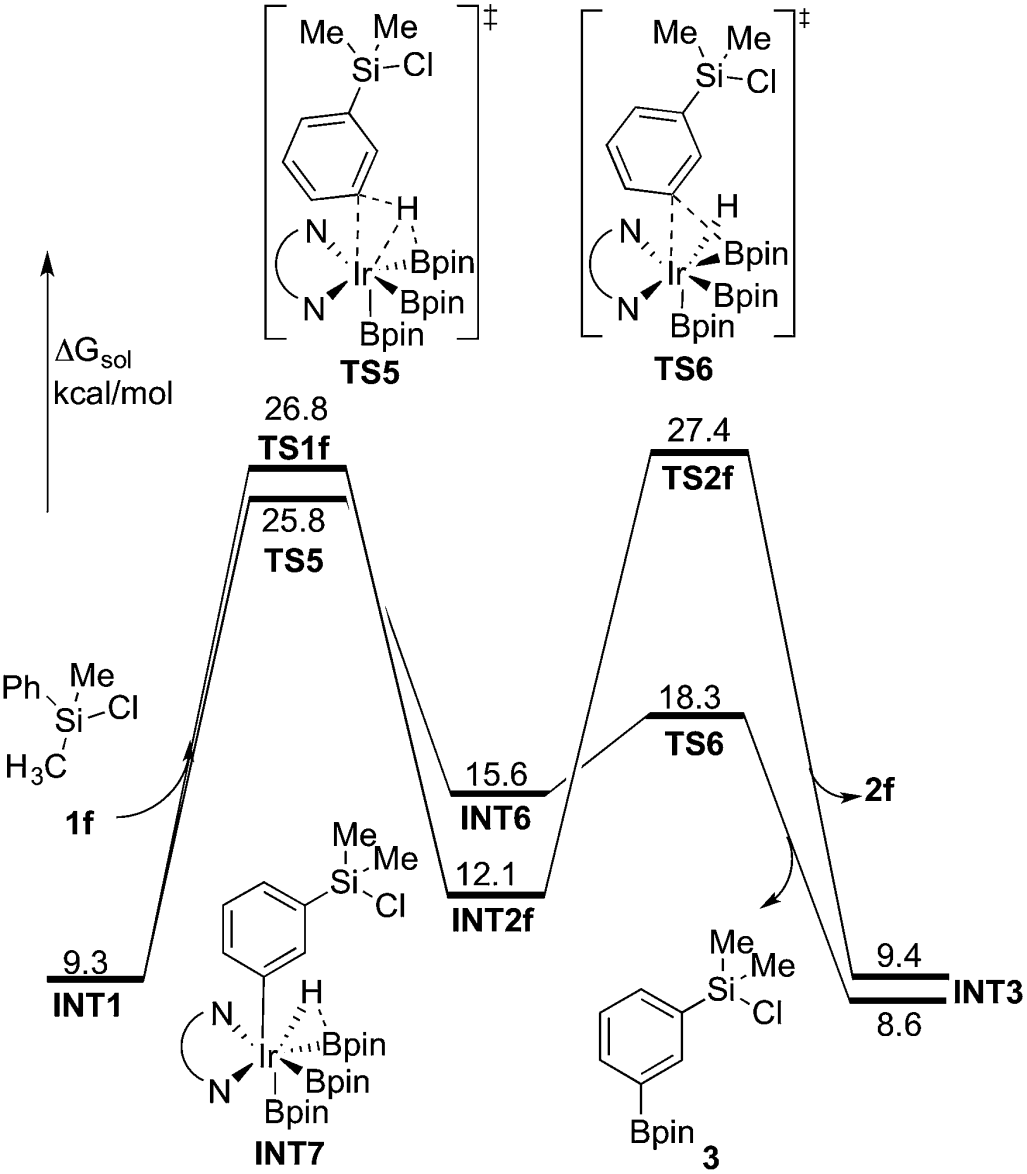
Energy profile calculated at M06 level of theory for the Ir-catalyzed C(sp^3^)–H and C(sp^2^)–H borylation of **1f**.

In general, it is considered that functionalizations of C(sp^2^)–H are easier than of C(sp^3^)–H.^1a,g^ For the borylation reaction investigated in the present work, when substrate **1f** was used (Scheme 2), C(sp^2^)–H borylation was exclusively observed (*ortho* : *meta* : *para* = 0 : 67 : 33).^8^ In order to investigate the origin of this selectivity, we have examined both the C(sp^2^)–H and C(sp^3^)–H borylation of **1f** at both M06 and B3LYP levels. The results for both functionals are in quite good agreement with the experimental observations. The energy profiles calculated using M06 for both C(sp^2^)–H and C(sp^3^)–H borylation of **1f** are shown in Fig. 6 (see ESI† for B3LYP results).

As shown in Fig. 6, the energy barrier for the C(sp^2^)–H oxidative addition at the *meta* position^35^ (**TS5**, 25.8 kcal mol^–1^ relative to **INT0**) was found to be 1.0 kcal mol^–1^ lower than that for the C(sp^3^)–H oxidative addition (**TS1f**, 26.8 kcal mol^–1^). However, in the former case, the resulting intermediate **INT6** is higher in energy than **INT2f**, by 3.5 kcal mol^–1^. Interestingly, **INT6** can undergo a facile direct reductive elimination (**TS6**) with an energy barrier of only 2.7 kcal mol^–1^ relative to **INT6**, *i.e.* 18.3 kcal mol^–1^ relative to **INT0**. Hence, the rate-determining step for the C(sp^2^)–H borylation is the C–H oxidative addition step, with an overall barrier of 25.8 kcal mol^–1^. In the case of the C(sp^3^)–H borylation, like for the substrates **1b–e**, the direct C–B reductive elimination from **INT2f** (**TS2f**) constitutes the rate-determining step, with an overall barrier of 27.4 kcal mol^–1^. The calculated 1.6 kcal mol^–1^ energy difference between C(sp^3^)–H and C(sp^2^)–H borylation is in qualitative agreement with the experimental results.

**Fig. 7:**
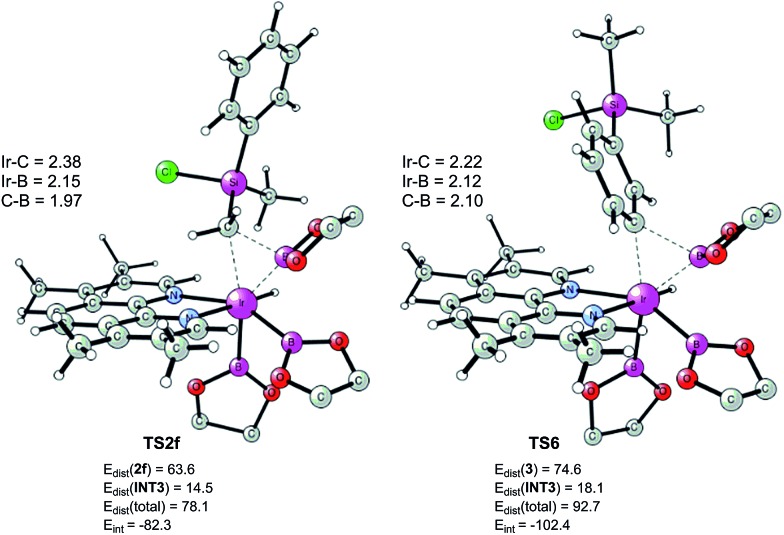
Optimized structures of reductive elimination transition states from **INT2f** and **INT6**. Distortion/interaction energies calculated using M06 are given in kcal mol^–1^.

According to these results, we find that a significant difference between C(sp^2^)–H and C(sp^3^)–H borylation originates from the different reactivity of the intermediates resulting from the C–H oxidative addition (**INT6** *vs.* **INT2f**) in the direct reductive elimination process (**TS6** *vs.* **TS2f**). In order to understand the origins of this difference, we performed a distortion/interaction analysis^36^ of **TS2f** and **TS6** (see Fig. 7). It turns out that the total distortion energy of **TS2f** is 14.6 kcal mol^–1^ lower than that of **TS6**, while the interaction energy of **TS6** was found to be higher than that of **TS2f** by as much as 20.1 kcal mol^–1^. Thus, a significant interaction energy difference between **TS2f** and **TS6** determines the reactivity. One plausible explanation for such a large interaction energy difference is that in **TS6** the presence of a Ph–B π* orbital can enhance the back-bonding interaction with the d orbital of iridium, while no such strong orbital interaction exists in **TS2f**. A similar explanation has been proposed by Lin and coworkers to account for the reactivity differences in the oxidative addition of alkyl and aryl halides to palladium.^37^

## 4. Conclusions

In the current study we have presented a detailed investigation of the iridium-catalyzed C(sp^3^)–H borylation of methylchlorosilanes by means of B3LYP and M06 density functional theory calculations. The two methods give quite a similar overall view of the catalytic mechanism, namely that the saturated seven-coordinate Ir(v) complex **INT0** is the resting state of the catalyst and it has to be converted into the active catalyst **INT1** to effect the oxidative addition of the C–H bond, which is then followed by C–B reductive elimination and regeneration of the active catalyst.

The two methods differ, however, in that B3LYP predicts that an isomerization step is needed prior to the C–B reductive elimination, whereas M06 predicts that the direct reductive elimination is preferred. The energy difference between the two scenarios for both methods is quite small and therefore no definitive conclusion can be drawn regarding this issue. Furthermore, for the model substrate EtMe_2_SiCl (**1a**), B3LYP predicts the isomerization step to be rate-determining, whereas M06 predicts the oxidative addition to be rate-determining. Again, the energy differences are rather small and both scenarios are calculated to be consistent with the experimentally-observed kinetic isotope effect.

A number of substrates with different substituents were also examined in the present work, and the calculations reproduce quite well the experimentally-observed trends in reactivity. A good correlation was found between the Ir–C bond dissociation energies of the Ir(v) hydride intermediate complex and the calculated energy barriers for the reaction. The accelerating role of the chlorosilyl group was ascribed to its strong α-carbanion stabilizing property, stemming from a combination of the silicon α-effect and the high electronegativity of the chlorine substituent.

Additionally, the origin of selectivity for the borylation of primary over secondary C(sp^3^)–H was investigated and explained in terms of a steric repulsion between the alkyl group and the Ir/ligand moiety.

Finally, the difference between C(sp^2^)–H and C(sp^3^)–H borylation was considered and this was found to originate from the difference in the reductive elimination step, which is much more facile for the C(sp^2^) case. It is argued that the origin of this difference is due to the presence of a strong back-bonding interaction between the Ph–B π* orbital and the d orbital of iridium in the C(sp^2^) case, which stabilizes the transition state for direct reductive elimination.

The present calculations thus provide a number of important insights into the mechanism of iridium-catalyzed borylation reactions, in particular in terms of selectivity and reactivity, and could be extended to other related systems.^38^ Therefore, we believe that the results will have important implications for the design of more efficient catalytic systems and directing groups in the field of metal-catalyzed C–H functionalization.
