# Supplementary material for: Mechanism, reactivity, and selectivity of the iridium-catalyzed C(sp3)–H borylation of chlorosilanes
Source: Chem Sci. 2014 Dec 4;6(3):1735–46. doi: 10.1039/c4sc01592d (PMC5644124; doi:10.1039/c4sc01592d)
Supplement: Supplementary file 1 [file SC-006-C4SC01592D-s001.pdf]

Electronic Supplementary Material (ESI) for Chemical Science.
This journal is © The Royal Society of Chemistry 2014

Supporting Information

Mechanism, Reactivity, and Selectivity of Iridium-Catalyzed C(*sp*^{3})-H Borylation of Chlorosilanes

Genping Huang, Marcin Kalek, Rong-Zhen Liao, and Fahmi Himo*

Department of Organic Chemistry

Arrhenius Laboratory

Stockholm University

SE-106 91 Stockholm, Sweden

Corresponding author e-mail: himo@organ.su.se

Table of Contents:

1. Alternative reaction pathways---S2
2. Optimized structures of various transition states--S4
3. Correlation between Ir-C BDEs in INT2 and the calculated energy barriers ----------------S5
4. B3LYP results for the secondary C-H borylation of 1a--S6
5. Results of the reaction of substrate 1f---S7
6. Table of calculated energies and energy corrections ---S8
7. Cartesian coordinates ---S10

1. Alternative reaction pathways.

1.1. C-H oxidative addition trans to nitrogen.

An alternative geometry for the C-H oxidative addition TS with the C-H bond positioned *trans* to the nitrogen ligand was considered (as opposed to **TS1a**, in which the C-H bond is *trans* to boryl ligand). Using B3LYP, the energy barrier for such configuration is 43.9 kcal/mol relative to **INT0**, which is 16.2 kcal/mol higher than **TS1a**. Using M06, the energy barrier is 46.8 kcal/mol relative to **INT0**, which is 18.5 kcal/mol higher than **TS1a**.

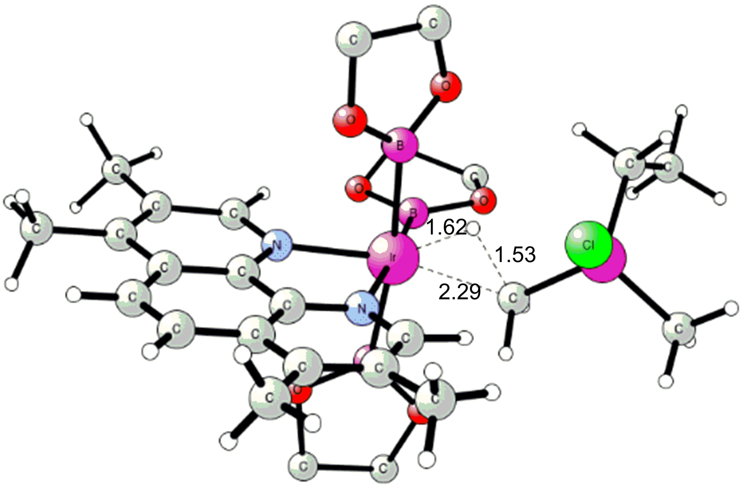

B3LYP-D3: 43.9
M06: 46.8

1.2. Si-Cl oxidative addition.

Si-Cl oxidative addition of **1a** was considered, and using B3LYP it was found to have an energy barrier of 40.9 kcal/mol relative to **INT0** (38.9 kcal/mol using M06), which is much higher than the C-H oxidative addition pathway.

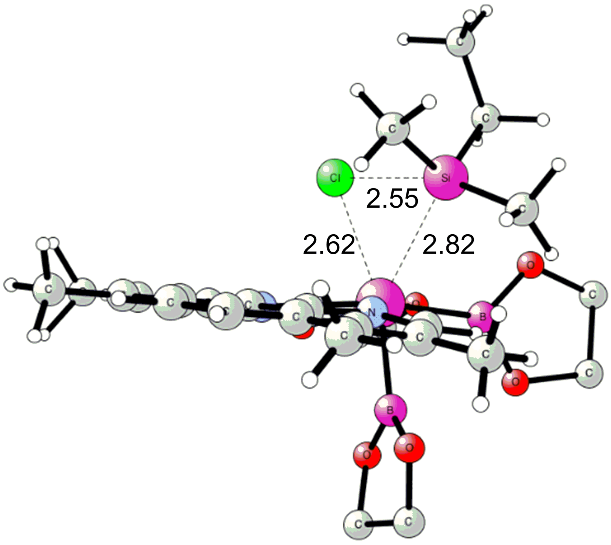

B3LYP-D3: 40.9
M06: 38.9

1.3. HBpin reductive elimination from INT2a.

A TS for a reductive elimination of HBpin from **INT2a** was optimized. The energy barrier relative to **INT0** is 53.5 and 51.2 kcal/mol, using B3LYP and M06, respectively.

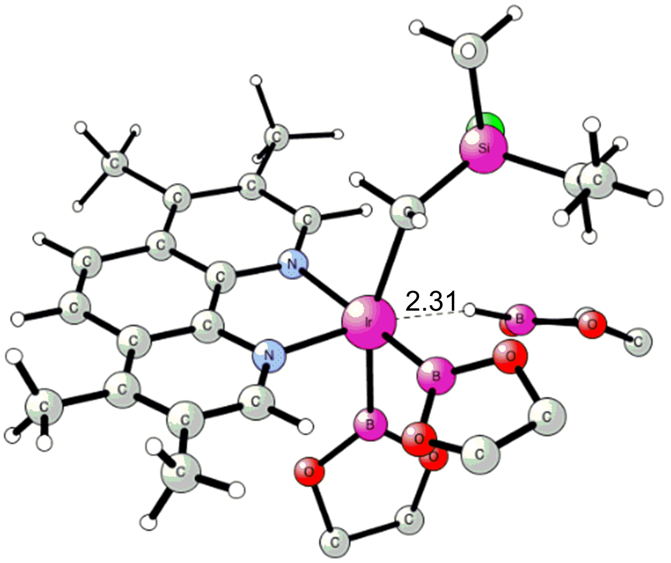

B3LYP-D3: 53.5
M06: 51.2

1.4. Alternative TS for the isomerization.

The isomerization of **INT2a** can also take place via a transition state involving exchanging the positions of H and B1 ligands. The calculated energy barrier relative to **INT0** is 31.6 and 33.8 kcal/mol using B3LYP and M06, respectively.

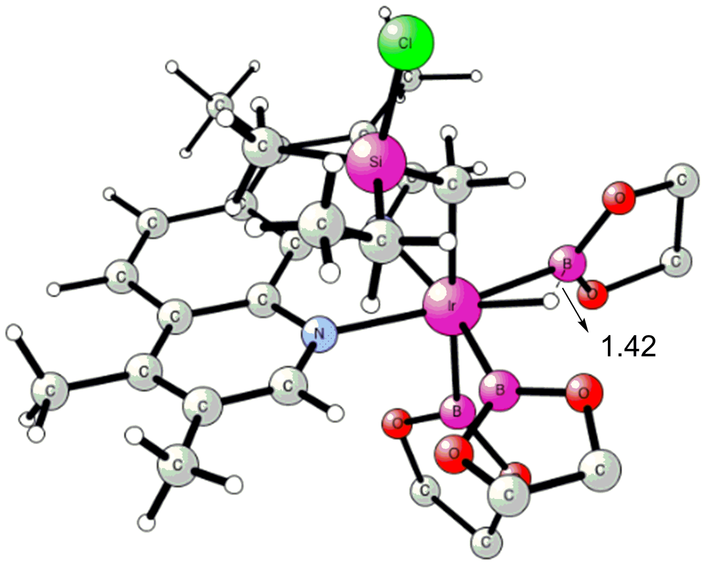

B3LYP-D3: 31.6
M06: 33.8

2. Optimized structures of various transition states.

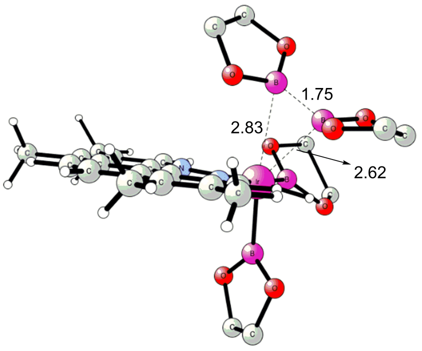

TS3

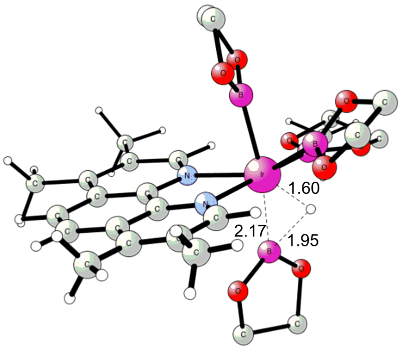

TS4

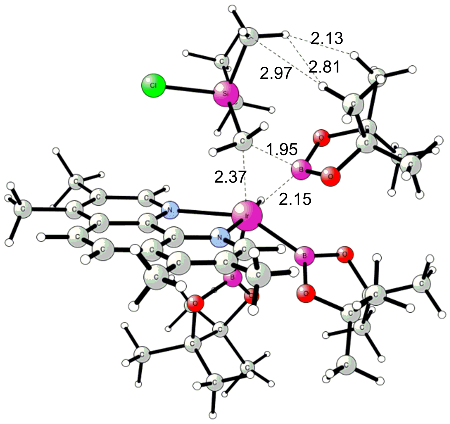

TS2a

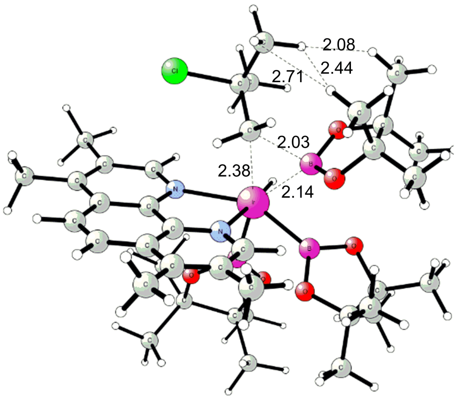

TS2b

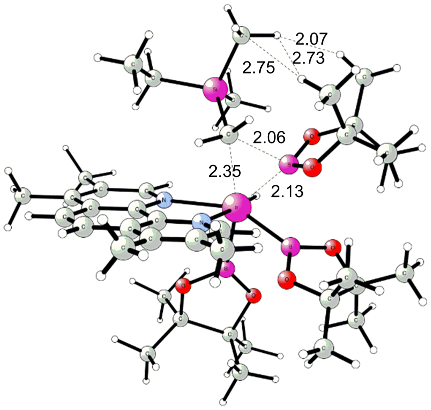

TS2c

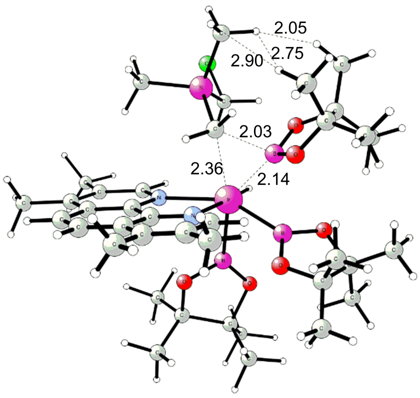

TS2d

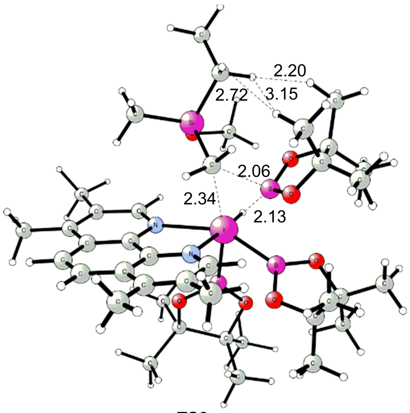

TS2e

3. Correlation between Ir-C BDEs in INT2 and calculated energy barriers.

B3LYP-D3 Results

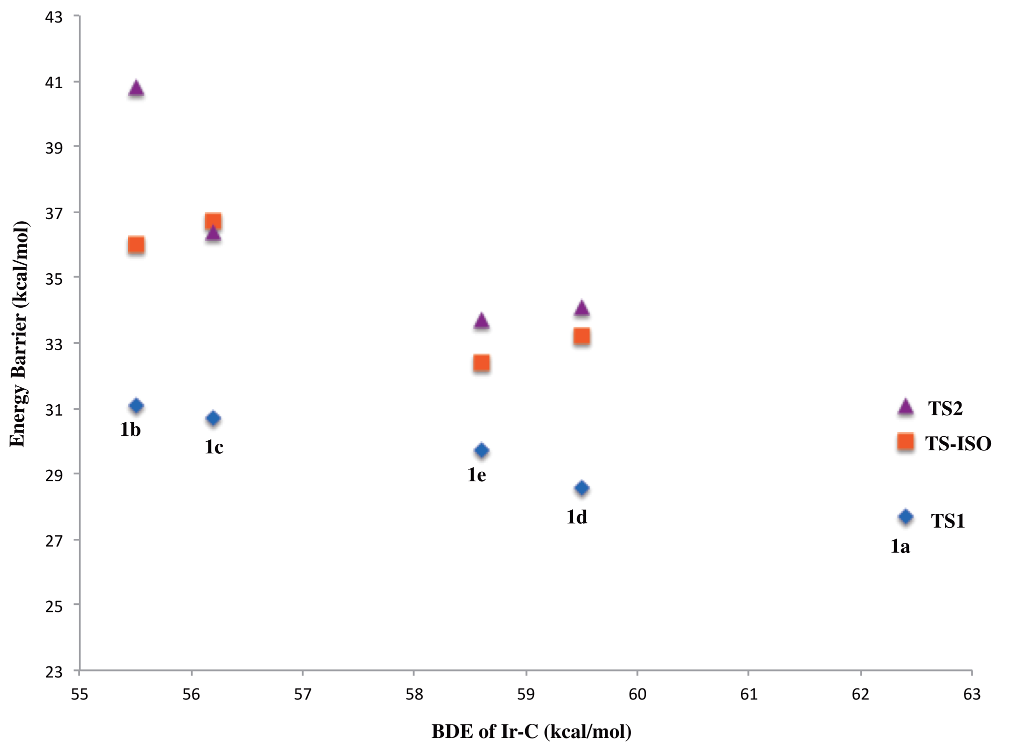

M06 Results

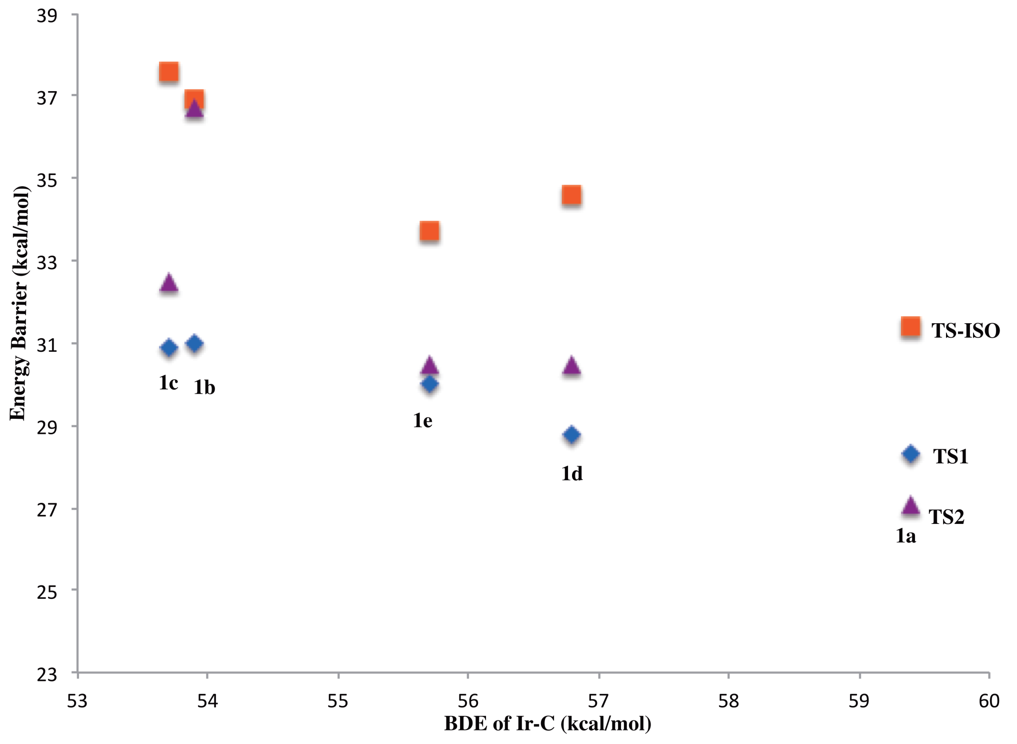

4. B3LYP results for the secondary C-H borylation of 1a.

Similarly to the primary C-H borylation, the B3LYP results show that for the secondary C-H borylation of **1a**, the direct C-B reductive elimination (**TS2a'**) is 2.1 kcal/mol higher than that of isomerization (**TSa-ISO'**). The calculated energy barriers for the direct C-B reductive elimination and isomerization are 36.6 and 34.5 kcal/mol, which are 7.0 and 4.9 kcal/mol higher than that of **TS1a'**, respectively. The energy difference between the primary and secondary C-H borylation is 4.5 kcal/mol, which is thus consistent with the experimental result.

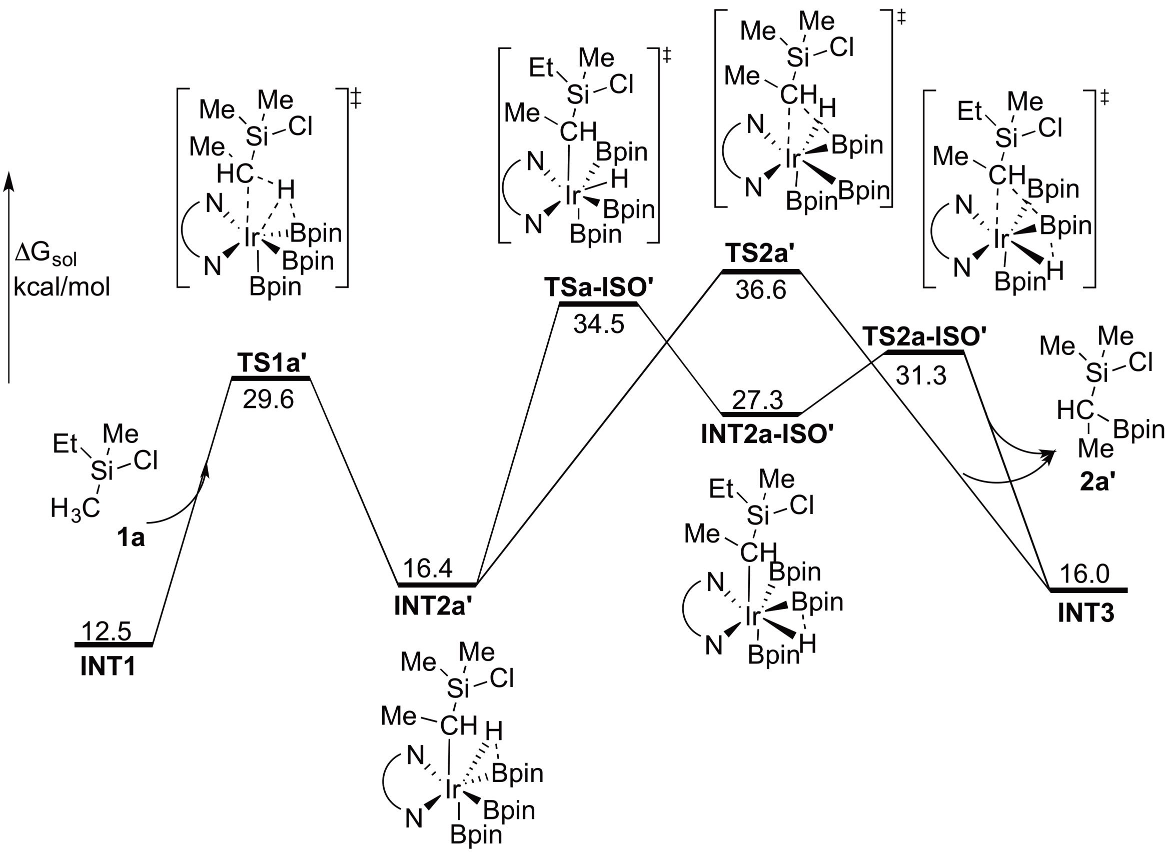

5. Results of the reaction of substrate 1f.

5.1 B3LYP results for the reaction of 1f

The B3LYP results show that the energy barrier for the C(*sp*^{3})-H borylation of **1f** is 27.7 kcal/mol (**TSf-ISO**), whereas for the C(*sp*^{2})-H borylation it is 24.0 kcal/mol. The 3.7 kcal/mol energy difference reproduces well the experimentally-observed selectivity.

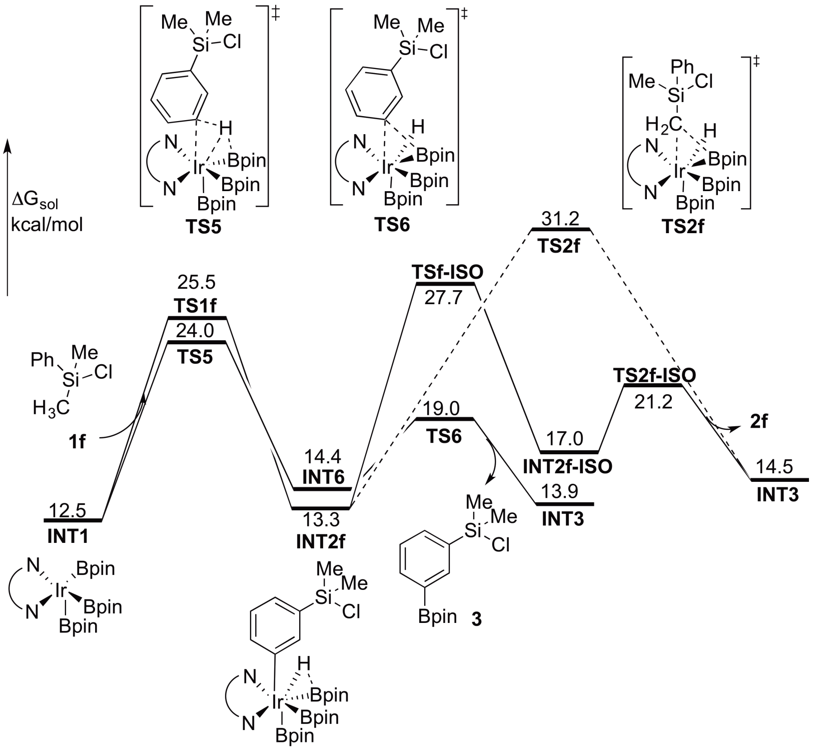

5.2. Ortho and para C(sp^2)-H oxidative additions.

The *ortho* and *para* C(*sp*^{2})-H oxidative additions of **1f** were calculated. For B3LYP, the energy barriers were found to be 33.9 and 25.9 kcal/mol relative to **INT0**, respectively, which are 9.9 and 1.9 kcal/mol higher in energy than **TS5**, respectively. For M06, the energy barriers were found to be 35.2 and 27.3 kcal/mol relative to **INT0**, respectively, which are 9.4 and 1.5 kcal/mol higher in energy than **TS5**, respectively.

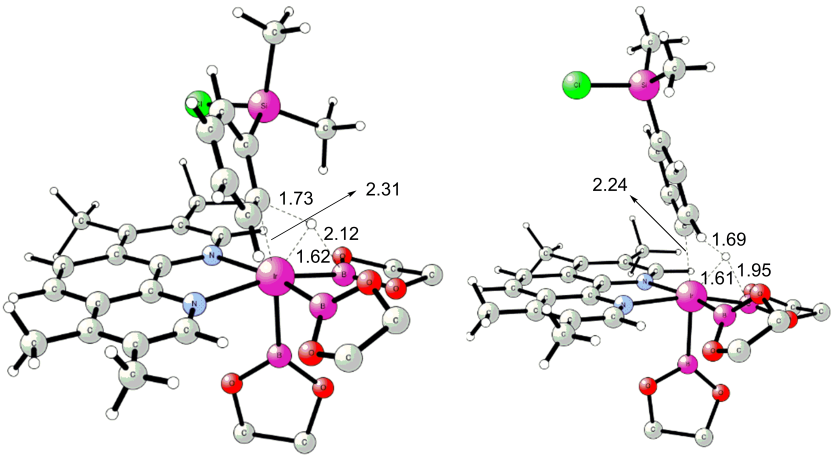

B3LYP-D3: 33.9
M06: 35.2

B3LYP-D3: 25.9
M06: 27.3

6. Table of calculated energies and energy corrections

Stationary point	Single-point energy-M06 6-311+G(2d,2p), lanl2tz*f* (a.u.)	Single-point energy-B3LYP 6-311+G(2d,2p), lanl2tz*f* (a.u.)	Thermal correction to Gibbs free energy at 298.15 K (a.u.)	Optimization energy 6-31G(d,p) , lanl2dz (a.u.)	Solvation energy (cyclohexane) (a.u.)	Dispersion correction (kcal/mol)
**INT0**	-2888.991618	-2890.911634	1.093011	-2890.106955	-0.00639999	-236.9144
**TS0**	-2888.9572322	-2890.884817	1.091314	-2890.088742	-0.00683532	-232.4316
**INT1**	-2066.680903	-2068.105260	0.745353	-2067.542338	-0.0080924	-150.3233
**1a**	-908.739620	-908.952029	0.106917	-908.841333	-0.001424071	-17.4772
**B_2pin_2**	-822.258991	-822.811388	0.315433	-822.565464	-0.002962325	-47.8436
**HBpin**	-411.725966	-412.005379	0.157545	-411.881560	-0.001656357	-22.8166
**2a**	-1319.292770	-1319.770092	0.266044	-1319.538965	-0.00258687	-45.0197
**TS1**	-2975.412549	-2977.022188	0.871930	-2976.346049	-0.00688698	-188.6044
**INT2a**	-2975.441683	-2977.042708	0.877817	-2976.363810	-0.00775644	-192.3087
**TSa-ISO**	-2975.406297	-2977.016414	0.871951	-2976.333726	-0.00829621	-189.0458
**INT2a-ISO**	-2975.429840	-2977.034716	0.876042	-2976.354574	-0.00797805	-191.0710
**TS2a-ISO**	-2975.423913	-2977.027583	0.877325	-2976.348900	-0.00773727	-190.8671
**TS2a**	-2975.422469	-2977.014774	0.880484	-2976.339532	-0.00740821	-194.8609
**INT3**	-1656.122743	-1657.287031	0.584253	-1656.840273	-0.009855	-117.5398
**TS3**	-2478.406430	-2480.090676	0.926906	-2479.406452	-0.00783496	-190.2806
**INT4**	-2478.425321	-2480.109852	0.925228	-2479.415472	-0.00727408	-191.7473
**TS4**	-2478.423152	-2480.108434	0.924948	-2479.414846	-0.00658897	-191.2958
**INT5**	-2478.427797	-2480.114422	0.923317	-2479.429326	-0.0077544	-187.3372
**2a'**	-1319.291843	-1319.767218	0.266821	-1319.536862	-0.00251514	-46.0610
**TS1a'**	-2975.412119	-2977.014759	0.873627	-2976.337375	-0.00580791	-193.0497
**INT2a'**	-2975.439065	-2977.034619	0.879130	-2976.355997	-0.00774824	-196.0271
**TSa-ISO'**	-2975.402376	-2977.007894	0.876495	-2976.326023	-0.00863207	-192.5274
**INT2a-ISO'**	-2975.416801	-2977.020376	0.879213	-2976.340074	-0.0077237	-194.1238
**TS2a-ISO'**	-2975.415319	-2977.014849	0.880030	-2976.337115	-0.00736971	-194.3895
**TS2a'**	-2975.408934	-2977.001308	0.879862	-2976.324519	-0.00801689	-197.0557
**1b**	-617.970403	-618.144178	0.093104	-618.065813	-0.001355306	-12.7616
**2b**	-1028.517516	-1028.956716	0.252194	-1028.758305	-0.00259034	-39.8249
**TS1b**	-2684.637942	-2686.211487	0.857336	-2685.566178	-0.00715239	-181.5162
**INT2b**	-2684.663631	-2686.230533	0.861635	-2685.582211	-0.00810698	-184.6063
**TSb-ISO**	-2684.629165	-2686.202836	0.860007	-2685.551291	-0.00919186	-182.4274
**INT2b-ISO**	-2684.654203	-2686.224055	0.862467	-2685.578484	-0.00875512	-183.5174

**TS2b-ISO**	-2684.648385	-2686.214891	0.863375	-2685.568508	-0.00795315	-183.8183
**TS2b**	-2684.636585	-2686.190852	0.865184	-2685.546155	-0.00723792	-189.6922
**1c**	-488.385304	-488.604585	0.141912	-488.517227	-0.000204068	-18.4295
**2c**	-898.938859	-899.422347	0.300807	-899.214354	-0.001569897	-46.0968
**TS1c**	-2555.054405	-2556.670087	0.906973	-2556.016741	-0.00542901	-189.5824
**INT2c**	-2555.079520	-2556.688883	0.911453	-2556.032617	-0.00580575	-191.8721
**TSc-ISO**	-2555.044337	-2556.660840	0.908143	-2556.001053	-0.00604158	-189.7742
**INT2c-ISO**	-2555.065424	-2556.679123	0.911194	-2556.021002	-0.00614852	-191.0600
**TS2c-ISO**	-2555.061482	-2556.672840	0.911552	-2556.017782	-0.00577748	-190.8302
**TS2c**	-2555.058922	-2556.656275	0.915391	-2556.003758	-0.00671741	-197.0482
**1d**	-908.688750	-908.903496	0.104592	-908.792170	-0.00132855	-17.4083
**2d**	-1319.241850	-1319.721437	0.263188	-1319.489430	-0.00254026	-44.8759
**TS1d**	-2975.362982	-2976.973238	0.870831	-2976.295667	-0.0058487	-189.1816
**INT2d**	-2975.387480	-2976.991076	0.871630	-2976.309979	-0.00636168	-191.7521
**TSd-ISO**	-2975.352070	-2976.965047	0.870450	-2976.280687	-0.00716547	-188.7257
**INT2d-ISO**	-2975.372105	-2976.981944	0.872741	-2976.299488	-0.00728216	-190.0850
**TS2d-ISO**	-2975.371670	-2976.977739	0.873877	-2976.297754	-0.00725319	-190.8774
**TS2d**	-2975.366503	-2976.959849	0.878871	-2976.283289	-0.00773288	-196.0128
**1e**	-563.627523	-563.876891	0.145267	-563.760488	-0.00098528	-19.3251
**2e**	-974.183628	-974.695896	0.306389	-974.459878	-0.001882725	-47.9519
**TS1e**	-2630.298409	-2631.944290	0.910847	-2631.262353	-0.00629029	-190.6566
**INT2e**	-2630.324699	-2631.963458	0.915302	-2631.278563	-0.00701469	-193.4945
**TSe-ISO**	-2630.292822	-2631.938350	0.910892	-2631.249530	-0.00603428	-191.8060
**INT2e-ISO**	-2630.312011	-2631.956736	0.914038	-2631.269107	-0.00616918	-192.4653
**TS2e**	-2630.306705	-2631.933986	0.920038	-2631.253122	-0.00638056	-198.7662
**TS2e-ISO**	-2630.313481	-2631.951564	0.916267	-2631.266877	-0.00629713	-194.0266
**1f**	-1061.081019	-1061.412573	0.128772	-1061.260595	-0.00209796	-27.3790
**3**	-1471.634909	-1472.233256	0.287248	-1471.962317	-0.00306497	-53.8204
**2f**	-1471.636199	-1472.230902	0.289743	-1471.959363	-0.00306631	-56.2528
**TS1f**	-3127.758968	-3129.477718	0.896530	-3128.759217	-0.00772073	-205.4917
**INT2f**	-3127.783715	-3129.504984	0.897544	-3128.782831	-0.00733376	-201.3965
**TSf-ISO**	-3127.755090	-3129.477792	0.898140	-3128.754434	-0.00866447	-203.5931
**INT2f-ISO**	-3127.773871	-3129.495056	0.899120	-3128.773018	-0.00805619	-204.4755
**TS2f-ISO**	-3127.771867	-3129.488625	0.900497	-3128.769590	-0.00784109	-205.3284
**TS2f**	-3127.765556	-3129.472631	0.904304	-3128.756543	-0.00792908	-207.7292
**TS5**	-3127.759323	-3129.490163	0.894467	-3128.769302	-0.00685265	-198.3918
**INT6**	-3127.777550	-3129.504761	0.896979	-3128.782583	-0.00739555	-200.0200
**TS6**	-3127.773270	-3129.491651	0.897553	-3128.772666	-0.00800471	-203.6155

7. Cartesian coordinates

1a

Si	1.61714000	0.92079600	3.52991300
C	-0.26195200	0.88859800	3.45279300
H	-0.67047500	1.89987200	3.36451800
H	-0.61344800	0.30687400	2.59525800
H	-0.67572800	0.43564000	4.36134000
C	2.22767700	1.98888100	4.95322500
H	1.91424800	1.56330000	5.91381200
H	3.31891600	2.06494900	4.95728200
H	1.82087400	3.00242300	4.88531600
Cl	2.30006400	1.81863700	1.74268700
C	2.31820300	-0.83110100	3.59647800
H	1.89309400	-1.31044300	4.49070500
H	1.91273100	-1.38785700	2.74181500
C	3.85201700	-0.94642700	3.61841400
H	4.29672200	-0.49332500	2.72714900
H	4.28220700	-0.44635400	4.49273200
H	4.17063300	-1.99377600	3.65201400

2a

B	0.49170000	9.06941500	-1.30120200
O	1.01242600	9.70964600	-2.40487300
C	2.39521000	10.05658500	-2.10763600
C	2.41071400	10.05200300	-0.52759500
O	1.31819400	9.14507400	-0.20720100
C	-0.90437700	8.35930300	-1.30000400
H	-0.91350500	7.56471600	-2.05779900
C	2.05354200	11.40922900	0.09301600
H	1.88854400	11.27562400	1.16554200
H	2.85655200	12.13962200	-0.04369400
H	1.13647400	11.81970000	-0.33920700
C	3.68950300	9.50960200	0.10820900
H	3.58883300	9.51800100	1.19719400
H	3.89137300	8.48297800	-0.20211000
H	4.55087300	10.13164200	-0.15685300
C	3.26569800	8.95669500	-2.72981200
H	3.05010100	7.98061400	-2.28626700
H	3.04897100	8.89601100	-3.79981600
H	4.33228200	9.16613200	-2.60550500
C	2.70645200	11.40279600	-2.75843100
H	3.72418400	11.72713700	-2.51739000
H	2.63037900	11.31055700	-3.84563100
H	2.00893100	12.17821200	-2.43689300
H	-1.09231500	7.89135400	-0.32824800
Si	-2.33132300	9.51821400	-1.72471300
Cl	-4.13137000	8.46196500	-1.36969400
C	-2.36761000	11.02529300	-0.59755600
H	-1.49295300	11.66091800	-0.77975400
H	-3.26402100	11.62899500	-0.76772400
H	-2.35605900	10.72869600	0.45583800
C	-2.29893100	9.97918900	-3.55360900
H	-1.30179300	10.39540000	-3.75179000
H	-2.34971800	9.04650200	-4.13056100
C	-3.39546600	10.94942800	-4.02402400
H	-3.33764300	11.90924900	-3.49925900
H	-3.30760400	11.15852600	-5.09582000
H	-4.39452700	10.53774300	-3.84903700

B_2pin_2

B	3.38658100	-2.08766500	-2.69773300
O	3.74712700	-3.40487400	-2.53818100
C	5.02818300	-3.43950400	-1.84387700
C	5.06791800	-2.02828900	-1.14173900
O	4.20426200	-1.22888800	-2.00193000
B	2.06925600	-1.58284000	-3.65438400

O	2.20338900	-0.96258100	-4.87378000
C	0.88470000	-0.50255400	-5.29205900
C	-0.07641500	-1.41759600	-4.44095600
O	0.75451000	-1.75875200	-3.29317000
C	-1.34526700	-0.73657600	-3.93143300
H	-1.97813900	-0.41400800	-4.76520800
H	-1.92014200	-1.44173900	-3.32430300
H	-1.11708100	0.13169700	-3.31097400
C	-0.43083900	-2.74226600	-5.13036300
H	-0.92221100	-3.39740100	-4.40593700
H	-1.10995400	-2.59011400	-5.97486000
H	0.46558300	-3.25368000	-5.49148000
C	0.80379700	0.98396300	-4.91957100
H	1.63330300	1.51341400	-5.39608900
H	-0.13399200	1.43548500	-5.25767700
H	0.89016200	1.12702400	-3.83899400
C	0.77497500	-0.67119700	-6.80638600
H	-0.22324700	-0.39157000	-7.16000500
H	1.50297900	-0.02178700	-7.30095500
H	0.97831600	-1.69848600	-7.11361900
C	6.10458900	-3.61623900	-2.92305000
H	5.88488000	-4.52063300	-3.49705500
H	6.11221000	-2.77096100	-3.61662600
H	7.10284800	-3.71868900	-2.48678800
C	5.02978400	-4.63987200	-0.89901500
H	4.95703500	-5.56308600	-1.48100800
H	5.95701900	-4.67876000	-0.31727200
H	4.18540600	-4.61122500	-0.20829300
C	6.44231700	-1.36329900	-1.08993800
H	6.35489500	-0.37873000	-0.62159200
H	7.14307300	-1.95961400	-0.49576100
H	6.86153700	-1.22406000	-2.08785700
C	4.42451500	-2.01954800	0.25149100
H	5.04236000	-2.54442300	0.98655500
H	4.30444100	-0.98333700	0.57940500
H	3.43461300	-2.48373200	0.23329700

HBpin

B	1.90049200	-0.63103100	-1.74760500
O	3.23877700	-0.57777200	-2.02135100
C	3.53563500	-1.66078900	-2.95302200
C	2.11746000	-1.96989200	-3.57737900
O	1.20840900	-1.51056600	-2.53262300
C	4.59089700	-1.16924500	-3.94192400
H	5.52003000	-0.95231800	-3.40729200
H	4.80480800	-1.93439000	-4.69558000
H	4.27544800	-0.25671100	-4.45058600
C	4.09917900	-2.81354600	-2.11158900
H	4.41421900	-3.65453400	-2.73660800
H	4.96878400	-2.45294300	-1.55556700
H	3.36359800	-3.17530000	-1.38772300
C	1.83095300	-3.44551200	-3.84841500
H	0.81550100	-3.55449900	-4.23976700
H	2.52568000	-3.84780500	-4.59332000
H	1.90699600	-4.04578900	-2.94022700
C	1.80270600	-1.13342600	-4.82437400
H	2.39629800	-1.45293800	-5.68615300
H	0.74437000	-1.25180800	-5.07193200
H	1.98999400	-0.07058700	-4.64793800
H	1.38540300	0.02772300	-0.90354900

INT0

Ir	-0.52471900	0.27394900	-0.57466800
N	0.35219000	-1.16149600	0.88600100
C	-0.05129400	-1.01338400	2.17939100
C	0.39160200	-1.88933400	3.20160100
C	1.27487200	-2.95363900	2.86651200
C	1.67910000	-3.07525000	1.53687600
C	1.19115700	-2.15180500	0.59082400

C	-0.94046700	0.07508800	2.49094500
N	-1.32910700	0.87856900	1.46992300
C	-2.12642700	1.90219600	1.74165400
C	-2.61234900	2.20386600	3.03157500
C	-2.23951700	1.38332400	4.09567700
C	-1.37324900	0.28741500	3.82309500
B	0.29511800	-0.44821400	-2.31489300
O	0.28677200	0.13183200	-3.57535500
C	1.24443300	-0.53326400	-4.41842400
C	1.35019400	-1.95303400	-3.74903600
O	1.01290100	-1.65984100	-2.37289900
B	1.40896700	1.17231600	-0.06836400
O	1.67556400	2.08176400	0.95165300
C	3.07541900	1.98542500	1.32651000
C	3.72226000	1.28319600	0.06957100
O	2.58886500	0.60346900	-0.52549700
H	1.47518100	-2.21905700	-0.45394900
H	-2.38768700	2.52615900	0.89565500
C	-2.72197800	1.63112900	5.50466800
H	-3.26820800	0.76457000	5.89450100
H	-1.88245000	1.81430100	6.18537400
H	-3.38683400	2.49275100	5.56199100
C	-3.51690000	3.40380400	3.19102400
H	-4.50566500	3.12183000	3.57029000
H	-3.09907300	4.14094600	3.88585900
H	-3.66326300	3.90217700	2.22978100
C	2.61734600	-4.15732700	1.05259500
H	2.20610600	-5.15896100	1.22162500
H	2.80017100	-4.05167800	-0.01916800
H	3.58757100	-4.11435800	1.55995300
C	1.74969700	-3.90715200	3.93584500
H	2.38428200	-4.69355800	3.52764300
H	2.32588700	-3.38323700	4.70752000
H	0.90471500	-4.39032700	4.43895400
C	-0.07460200	-1.65431700	4.53898100
H	0.25270100	-2.31528400	5.33348900
C	-0.91144900	-0.61933300	4.83478600
H	-1.23537400	-0.47622600	5.85965500
C	3.13369800	1.13556900	2.60446100
H	4.15383000	1.05629800	2.99352600
H	2.50952300	1.60526100	3.37008900
H	2.74965900	0.12752700	2.43071100
C	4.79260300	0.23844600	0.39153100
H	5.14390700	-0.21935100	-0.53785800
H	5.65493400	0.69573600	0.88914900
H	4.40274900	-0.55656000	1.02982600
C	4.25812500	2.26660000	-0.97856500
H	5.12634500	2.82253700	-0.61013100
H	4.56169700	1.70678400	-1.86754300
H	3.48144400	2.96966400	-1.28244700
C	3.59908800	3.38989500	1.62839200
H	3.05196300	3.80985900	2.47780000
H	4.66236000	3.36392000	1.89136100
H	3.46998100	4.06260000	0.77899200
C	0.71573300	-0.52881700	-5.85324000
H	0.64502700	0.50291400	-6.21029600
H	1.38652000	-1.07499200	-6.52638500
H	-0.27979200	-0.97230200	-5.91281100
C	0.30569200	-2.94859600	-4.27379100
H	0.50425500	-3.23925500	-5.31064200
H	0.33424300	-3.85090400	-3.65587200
H	-0.69999900	-2.52647100	-4.20613800
C	2.74090900	-2.58767000	-3.78739000
H	2.71914100	-3.55936800	-3.28249000
H	3.06911200	-2.75565300	-4.81922400
H	3.47918100	-1.96075200	-3.28417000
C	2.55200400	0.27114100	-4.33844500
H	2.34408800	1.30321800	-4.63302500
H	2.92930800	0.29084900	-3.31334300

H	3.32457500	-0.13267800	-5.00180800
B	-1.97471100	-1.35166800	-0.69897800
O	-2.40249400	-1.94879200	0.48375100
C	-3.36852000	-2.98516200	0.19171100
C	-3.06713300	-3.30138800	-1.32217300
O	-2.46525800	-2.06448000	-1.78965200
B	-2.25241200	0.86648800	-1.64398400
O	-2.37890000	1.25091300	-2.97140600
C	-3.77827400	1.36665900	-3.31618000
C	-4.46556100	1.54936700	-1.90857400
O	-3.50301500	0.94313000	-1.01059400
C	-4.61949500	3.01386800	-1.47450700
H	-5.34532000	3.55175700	-2.09287300
H	-4.97425400	3.03709600	-0.43903300
H	-3.66036100	3.53176500	-1.51320200
C	-5.80853600	0.83361000	-1.75295100
H	-6.17278400	0.95398300	-0.72810100
H	-6.55772800	1.26096400	-2.42874700
H	-5.72905300	-0.23434400	-1.96169400
C	-3.95917800	2.54299300	-4.27682100
H	-3.38931700	2.35672000	-5.19162500
H	-5.01305200	2.66088100	-4.55285300
H	-3.60952500	3.48303500	-3.84700700
C	-4.16313900	0.06369300	-4.03286600
H	-5.20902800	0.06717900	-4.35754400
H	-3.52896300	-0.04320600	-4.91752500
H	-3.98173700	-0.80090200	-3.39221400
C	-4.75833400	-2.38173100	0.43521200
H	-4.81965800	-2.05023300	1.47598000
H	-4.91854200	-1.50885800	-0.19970800
H	-5.55847400	-3.10792800	0.25861700
C	-3.13529700	-4.14898900	1.15703500
H	-3.33147600	-3.81689000	2.18104000
H	-3.80937800	-4.98497500	0.93961500
H	-2.10617400	-4.51022600	1.11270400
C	-4.29648300	-3.60691900	-2.17741400
H	-3.99280500	-3.75882700	-3.21739300
H	-4.79348300	-4.52084900	-1.83384000
H	-5.02078400	-2.79096200	-2.15359300
C	-2.01891000	-4.40628000	-1.51604900
H	-2.40070400	-5.38670400	-1.21388400
H	-1.74653800	-4.45386000	-2.57292900
H	-1.11013800	-4.19065400	-0.94793700
B	-0.22213900	2.25036000	-1.29345800
O	-1.05779200	3.31209600	-0.90674000
C	-0.48290700	4.56359000	-1.36569700
C	0.44740600	4.08578700	-2.54064400
O	0.77063500	2.73277700	-2.14221000
C	0.28080400	5.16595400	-0.17733200
H	0.72699900	6.13392900	-0.42812800
H	-0.41926600	5.31822900	0.64995000
H	1.06064300	4.48648100	0.17139300
C	1.74251000	4.88230100	-2.69856100
H	2.35454500	4.43198000	-3.48540600
H	1.52935400	5.91729000	-2.98838800
H	2.33009200	4.89828400	-1.77898900
C	-0.26650200	3.98677700	-3.89574600
H	-0.53415100	4.97267100	-4.29036500
H	0.40538500	3.50063800	-4.60837700
H	-1.15970500	3.36549600	-3.81589000
C	-1.60483400	5.51307600	-1.78479600
H	-2.23313800	5.75137400	-0.92087700
H	-1.19064400	6.45294300	-2.16618900
H	-2.23927400	5.07956600	-2.55911000

TS0

Ir	-0.29196600	0.53908600	-0.47516300
N	0.23479600	-1.43320800	0.60828500
C	0.05833500	-1.39787000	1.95715900

C	0.54113100	-2.42769300	2.80349500
C	1.27389500	-3.50707600	2.22875300
C	1.46722500	-3.50527400	0.84890100
C	0.91396400	-2.45014800	0.09008800
C	-0.63520300	-0.26383500	2.51962900
N	-1.00437600	0.73656600	1.67756500
C	-1.66235300	1.77011900	2.18295400
C	-2.00686200	1.90718100	3.54481300
C	-1.61344500	0.90853600	4.43226700
C	-0.90015700	-0.20884600	3.91000100
B	0.49361600	0.14032500	-2.32964600
O	0.50122600	0.88470400	-3.50683100
C	1.38334000	0.27223700	-4.46953700
C	1.42101700	-1.22121100	-3.98389000
O	1.18824500	-1.07161100	-2.56642300
B	1.60123100	1.12172700	-0.06490300
O	2.52327200	1.81796300	-0.84862000
C	3.70752100	2.08529700	-0.05581900
C	3.62104600	0.97311600	1.05236000
O	2.19571600	0.75479800	1.14819500
H	1.04254200	-2.41398100	-0.98718000
H	-1.91172700	2.55316800	1.47805900
C	-1.91633200	0.98733200	5.90906200
H	-2.50625900	0.12508900	6.24029500
H	-0.99464400	0.99298100	6.50237200
H	-2.47671500	1.88706000	6.16226300
C	-2.77086500	3.13985400	3.96902200
H	-3.73652100	2.88625400	4.42059300
H	-2.21351000	3.73392800	4.70189600
H	-2.96963400	3.78161600	3.10748700
C	2.23077600	-4.58466500	0.11624500
H	1.77804000	-5.57303200	0.25486200
H	2.24911400	-4.37691400	-0.95623800
H	3.27010400	-4.65141200	0.45685100
C	1.81472100	-4.60737900	3.10953400
H	2.36142900	-5.35531100	2.53558200
H	2.49597300	-4.20872600	3.86980500
H	1.00692800	-5.12322200	3.64161400
C	0.27178800	-2.33183700	4.20991400
H	0.62784000	-3.11469800	4.87010600
C	-0.42355400	-1.28250600	4.73408600
H	-0.61128800	-1.25225600	5.80154500
C	3.57304100	3.50633700	0.50804100
H	4.44346100	3.78884000	1.10881700
H	3.49364400	4.21073900	-0.32451900
H	2.67129300	3.60173800	1.11734300
C	4.14634000	1.37813600	2.42911400
H	4.01323600	0.54872900	3.13062800
H	5.21461100	1.61853000	2.38980600
H	3.60740100	2.24136700	2.82383600
C	4.25113900	-0.36076200	0.62077200
H	5.34400200	-0.30622200	0.58339500
H	3.96970000	-1.13205400	1.34386300
H	3.88034300	-0.66904400	-0.36080600
C	4.93415600	2.00317500	-0.96410300
H	4.87772500	2.78449500	-1.72756700
H	5.85570500	2.15627100	-0.39123100
H	4.99824600	1.04019700	-1.47378400
C	0.81690600	0.47914300	-5.87484100
H	0.75866500	1.55023600	-6.08995400
H	1.46416500	0.01737200	-6.62945300
H	-0.18546800	0.06036500	-5.97671800
C	0.26964700	-2.07113000	-4.54405400
H	0.39167200	-2.27309000	-5.61358700
H	0.24906700	-3.03058800	-4.01757100
H	-0.69206600	-1.57911700	-4.37832500
C	2.75408500	-1.94100900	-4.18999300
H	2.69120200	-2.95920500	-3.79118700
H	3.00212900	-2.01594300	-5.25470300

H	3.57058800	-1.42735500	-3.67848500
C	2.74099400	0.98341200	-4.35527300
H	2.59438900	2.05045800	-4.54557500
H	3.14745200	0.88477000	-3.34666400
H	3.46794500	0.60153700	-5.08012800
B	-2.63856100	-1.12139600	-0.60517100
O	-3.17960600	-1.08918400	0.66252900
C	-3.86927300	-2.34302600	0.91280000
C	-3.28070100	-3.30953800	-0.20294900
O	-2.67112500	-2.38731700	-1.14448600
B	-2.73420300	0.19821500	-1.75725900
O	-2.70614500	-0.11864400	-3.09366600
C	-3.55699500	0.80734900	-3.82819900
C	-4.39479000	1.50128300	-2.67179900
O	-3.60668700	1.23077300	-1.47850900
C	-4.54979900	3.01346800	-2.80935800
H	-5.11040400	3.27216000	-3.71396600
H	-5.09983400	3.40424400	-1.94745500
H	-3.57436100	3.49530900	-2.84538300
C	-5.76901400	0.86097700	-2.42554000
H	-6.19767200	1.28348300	-1.51237000
H	-6.45964000	1.05458100	-3.25213800
H	-5.68858500	-0.22094500	-2.28965900
C	-2.65321200	1.76694500	-4.60637500
H	-2.05889700	1.19216800	-5.31977300
H	-3.25215600	2.49016100	-5.17053200
H	-1.96283300	2.29037200	-3.94489900
C	-4.39009300	-0.02329900	-4.80918900
H	-5.08863300	0.61127100	-5.36514600
H	-3.72286900	-0.50193400	-5.53135600
H	-4.95631800	-0.80914300	-4.30599300
C	-5.36735300	-2.04427400	0.75209700
H	-5.64936500	-1.26436900	1.46464600
H	-5.59770200	-1.67397800	-0.24969800
H	-5.98133900	-2.92885500	0.94763000
C	-3.59510600	-2.77219700	2.35510900
H	-3.98593900	-2.01254200	3.03804000
H	-4.09565200	-3.71971600	2.58157200
H	-2.52920000	-2.88580700	2.55493200
C	-4.32823000	-4.12216300	-0.97043400
H	-3.83012900	-4.71217900	-1.74493100
H	-4.85689400	-4.81211600	-0.30431500
H	-5.06108400	-3.47969300	-1.46123400
C	-2.18488400	-4.25516900	0.29824300
H	-2.59603500	-5.02626600	0.95786300
H	-1.72653700	-4.75018600	-0.56252900
H	-1.40202400	-3.71967600	0.83373600
B	-0.51619900	2.51001400	-0.98661700
O	-0.23961600	3.50683900	-0.01739900
C	-0.54942000	4.81051000	-0.55884800
C	-0.47554100	4.54069500	-2.10111400
O	-0.88146400	3.15487000	-2.17466600
C	0.45790800	5.82867700	-0.02578000
H	0.27696600	6.82038900	-0.45569100
H	0.36055500	5.91115000	1.06151500
H	1.48348200	5.53459800	-0.24950800
C	0.95188500	4.61473600	-2.66725600
H	0.93710800	4.22766200	-3.68964000
H	1.33284100	5.64151800	-2.68995500
H	1.63180900	3.98501800	-2.08923800
C	-1.40581100	5.40138800	-2.95572700
H	-1.10513900	6.45424500	-2.91054100
H	-1.34702900	5.08141500	-4.00029200
H	-2.44717700	5.33113900	-2.63751900
C	-1.96173900	5.18274500	-0.07813700
H	-1.97721400	5.16599200	1.01659600
H	-2.25719400	6.18619600	-0.40156400
H	-2.70076000	4.46429200	-0.44306300

INT1

B	-0.65754300	-0.42803400	1.36935400
B	-1.43588300	-1.01905600	-1.15879300
B	-1.43217800	1.62154500	-0.36617900
C	1.94834400	2.76043300	0.00306600
H	0.98032300	3.25415100	0.03731400
C	3.14738100	3.49974900	0.10746300
C	4.32894700	1.39549700	-0.06778100
C	3.07334500	0.75049900	-0.17191400
C	3.00187100	-0.68508100	-0.30510200
C	4.18391200	-1.46386700	-0.30693500
C	2.79126700	-3.43526700	-0.47124000
C	1.67996100	-2.56574100	-0.47427600
H	0.67123700	-2.96389300	-0.51849700
N	1.90017600	1.43925200	-0.13327100
N	1.76743400	-1.24310900	-0.41024400
O	-1.72339500	0.07833300	2.11218300
O	-0.00841100	-1.44264600	2.08459300
O	-1.78378900	-0.91240300	-2.51339400
O	-2.12779300	-2.09834300	-0.58608900
O	-1.14276900	2.97276000	-0.06717200
O	-2.80363300	1.50844900	-0.61453800
C	5.51044600	0.58018300	-0.10109900
C	5.44155400	-0.77812000	-0.21067100
H	6.48183600	1.05651300	-0.02998500
H	6.35948000	-1.35513600	-0.22275300
C	4.36125900	2.81406900	0.07176000
C	4.06996900	-2.88136500	-0.39982100
C	-0.82902800	-1.79730800	3.22187500
C	-1.67459800	-0.48524200	3.44213200
C	-3.40243200	2.81871800	-0.67106200
C	-2.38273000	3.68475600	0.15421200
C	-2.60846500	-2.03628800	-2.88555600
C	-3.17843200	-2.50486400	-1.49544100
Ir	0.00672500	0.17487600	-0.40890100
C	-3.47642900	3.22049800	-2.15288500
H	-4.02347700	2.44733200	-2.69947700
H	-3.99216500	4.17600400	-2.29581100
H	-2.47637800	3.29236100	-2.58931500
C	-4.81137600	2.73994600	-0.08168300
H	-5.27713400	3.73086400	-0.03447500
H	-5.43845100	2.10354900	-0.71368700
H	-4.79950800	2.30830200	0.92056600
C	-2.21791800	5.12789100	-0.32272200
H	-3.16038600	5.68011000	-0.23654300
H	-1.47461100	5.64002700	0.29722000
H	-1.88243800	5.17417600	-1.36074800
C	-2.65432500	3.65569600	1.66632900
H	-1.80838400	4.11618900	2.18631000
H	-3.56075300	4.21029200	1.93090300
H	-2.74639900	2.62544600	2.01886700
C	-3.65853400	-1.55872200	-3.88947800
H	-3.16471300	-1.22996700	-4.80913900
H	-4.35525600	-2.36416500	-4.14859500
H	-4.22742200	-0.71565400	-3.49428800
C	-1.69300700	-3.07805100	-3.54899500
H	-2.25425100	-3.93678000	-3.93224700
H	-1.17186900	-2.60569700	-4.38684400
H	-0.93834600	-3.44297200	-2.84626100
C	-4.44933900	-1.74858100	-1.07892800
H	-5.31125000	-2.02485000	-1.69554500
H	-4.68038700	-1.99555600	-0.03839600
H	-4.29135000	-0.66896400	-1.13724700
C	-3.39424100	-4.01171800	-1.35610900
H	-3.76634400	-4.24005000	-0.35265600
H	-4.13508600	-4.37072300	-2.07923900
H	-2.46644600	-4.56851700	-1.50406800
C	-3.10755500	-0.70976100	3.92494900
H	-3.12253500	-1.19775200	4.90608600

H	-3.61875900	0.25311500	4.01984700
H	-3.67230400	-1.32158700	3.21954200
C	-0.97224900	0.54168100	4.34386200
H	-1.51284000	1.49076400	4.28770300
H	-0.94835300	0.22062500	5.39037500
H	0.05383600	0.71898800	4.00962600
C	0.08807700	-2.17876400	4.38468400
H	-0.49219000	-2.39785000	5.28802200
H	0.65547200	-3.07752500	4.12381900
H	0.80297200	-1.38589600	4.61329800
C	-1.68002400	-3.00695300	2.80684100
H	-1.01061600	-3.82672500	2.52790200
H	-2.32213800	-3.35442500	3.62286300
H	-2.29262200	-2.76901800	1.93437400
C	3.04828300	5.00038200	0.25633600
H	2.00207800	5.31481600	0.26022800
H	3.50228700	5.34844400	1.19084300
H	3.54821000	5.52586100	-0.56498400
C	5.68615500	3.52856900	0.17997200
H	6.25625300	3.17475100	1.04657600
H	6.30537400	3.34960200	-0.70653500
H	5.56161600	4.60609300	0.28386100
C	5.31278800	-3.73747300	-0.40728100
H	5.96685600	-3.47853300	-1.24776900
H	5.89602700	-3.59560000	0.50978400
H	5.07690700	-4.79826100	-0.48761300
C	2.53715000	-4.92255500	-0.54967500
H	2.98744300	-5.36766000	-1.44402500
H	2.94519600	-5.45071700	0.31908100
H	1.46434000	-5.12678200	-0.58417300

TS1a

Ir	0.99937000	-0.01539500	-0.00376000
N	-0.16567600	-1.82101300	0.81812800
C	-0.45517100	-1.75595000	2.14275000
C	-1.16761800	-2.78401500	2.80952700
C	-1.59373600	-3.91893800	2.06189400
C	-1.28793300	-3.96147900	0.70177400
C	-0.57107100	-2.88435400	0.13655400
C	-0.00724800	-0.59209100	2.86686300
N	0.67409000	0.36618300	2.17776100
C	1.09801200	1.43967700	2.83943100
C	0.88060500	1.65368900	4.21664200
C	0.18292800	0.69008900	4.94370300
C	-0.27511600	-0.46813600	4.25287200
B	2.85352400	-0.84568000	0.56881700
O	3.96719000	-0.13259000	0.99663900
C	5.07165300	-1.05032200	1.19612300
C	4.32821200	-2.42118300	1.41209600
O	3.09097800	-2.21791200	0.68586000
B	1.70070200	-0.91818900	-1.73170400
O	2.95311300	-0.80855300	-2.32430600
C	2.93545200	-1.47722200	-3.60707000
C	1.77151600	-2.51543700	-3.41998600
O	0.91984000	-1.83364500	-2.46220400
B	1.99631600	1.68458200	-0.53162300
O	2.29183700	2.12228700	-1.82411300
C	3.16752300	3.26975500	-1.74509200
C	2.80777100	3.85848600	-0.33360900
O	2.40019800	2.66999200	0.38522500
H	-0.31078700	-2.88387100	-0.91772400
H	1.64280400	2.16412800	2.24175100
C	-0.09107700	0.84630200	6.41944600
H	-1.16803000	0.85491700	6.62302400
H	0.33795000	0.01621900	6.99213100
H	0.32685900	1.77149800	6.81517200
C	1.41932900	2.92502700	4.83111200
H	0.62043400	3.54078000	5.25902100
H	2.13706000	2.71732100	5.63243900

H	1.93249900	3.52571000	4.07679000
C	-1.68685300	-5.10602200	-0.20039300
H	-2.77407300	-5.23620200	-0.23715800
H	-1.34277600	-4.92582300	-1.22157700
H	-1.25444000	-6.05646900	0.13155500
C	-2.35491600	-5.02916200	2.74492900
H	-2.59416800	-5.84114500	2.05877100
H	-1.77694400	-5.45402600	3.57332300
H	-3.29828100	-4.66198600	3.16537800
C	-1.42595900	-2.62555600	4.21193700
H	-1.97249100	-3.39938100	4.73908100
C	-1.00137700	-1.52546000	4.89700800
H	-1.21815700	-1.44515600	5.95611500
C	-1.08599200	1.02573900	-0.36933000
H	0.12747600	0.26291200	-1.31859300
H	-0.88352400	1.98420200	0.11673200
C	2.88003100	4.19236700	-2.92897000
H	3.47935500	5.10778700	-2.86997700
H	3.13799500	3.68169700	-3.86164800
H	1.82514400	4.46859700	-2.97947000
C	1.60299300	4.81096600	-0.36518900
H	1.27954000	5.01367400	0.66031600
H	1.84989600	5.76619700	-0.83940600
H	0.76294300	4.36304300	-0.90245200
C	3.96878000	4.51064500	0.41705500
H	4.36258000	5.36913900	-0.13809500
H	3.62358700	4.87323800	1.39083100
H	4.78072200	3.80252400	0.59042800
C	4.60856300	2.74265100	-1.82432700
H	4.71966700	2.16322500	-2.74477900
H	5.34248600	3.55537300	-1.83484300
H	4.82400100	2.07728800	-0.98441900
C	5.92052000	-1.01471900	-0.08263900
H	6.27334400	0.00778600	-0.24498500
H	6.79469900	-1.66986700	-0.01022100
H	5.32421800	-1.30117700	-0.95183300
C	5.89874500	-0.56337700	2.38600300
H	6.71691800	-1.25751000	2.60820900
H	6.33786600	0.41063900	2.15087100
H	5.28711300	-0.44665000	3.28277200
C	3.94617000	-2.67995000	2.87775900
H	3.27109000	-3.53964000	2.92120700
H	4.82166800	-2.89817800	3.49770200
H	3.42415100	-1.81832200	3.30335000
C	5.03780900	-3.64746300	0.83890100
H	6.00236200	-3.81252100	1.33182600
H	4.42129400	-4.53721900	0.99981000
H	5.21019900	-3.54613700	-0.23405400
C	2.63297300	-0.41020600	-4.66975800
H	3.37694000	0.38629000	-4.58849500
H	2.67296700	-0.82224300	-5.68334400
H	1.64928600	0.03878800	-4.51140700
C	0.95361200	-2.81482800	-4.67528900
H	1.57841300	-3.27392100	-5.44929500
H	0.14973600	-3.51719500	-4.43387100
H	0.49780200	-1.91222800	-5.08629900
C	2.23313700	-3.82542200	-2.76402500
H	1.35494600	-4.42690600	-2.50894000
H	2.86144500	-4.41744000	-3.43702600
H	2.78548200	-3.62725700	-1.84163300
C	4.31379500	-2.08604400	-3.86350200
H	5.05837300	-1.28799900	-3.93766800
H	4.61639700	-2.75528400	-3.05585200
H	4.32677700	-2.64745200	-4.80424600
H	-1.71059600	0.41530500	0.28846500
Si	-1.96869700	1.31525400	-1.98435600
C	-1.02333900	2.47852500	-3.12364200
H	-1.01839800	3.49277100	-2.71097100
H	0.01895900	2.15782100	-3.22310200

H	-1.48232500	2.52533700	-4.11641200
C	-2.42911600	-0.28906400	-2.87611900
H	-2.86424200	-0.01807100	-3.84714700
H	-1.48795800	-0.81283000	-3.09241400
C	-3.38537200	-1.22815600	-2.12171200
H	-2.95446600	-1.55726900	-1.16981700
H	-4.33615300	-0.73370000	-1.89945800
Cl	-3.82122400	2.29970000	-1.56669200
H	-3.60716500	-2.12750500	-2.70857500

INT2a

Ir	0.89142300	0.02484400	-0.02899300
N	-0.28786300	-1.82747900	0.73451500
C	-0.48475400	-1.81837900	2.07707500
C	-1.22635200	-2.82814400	2.74041200
C	-1.78056700	-3.88953500	1.97011800
C	-1.55434400	-3.88635700	0.59462100
C	-0.80028100	-2.83137100	0.03570300
C	0.10038600	-0.73702600	2.82586800
N	0.81253100	0.20487400	2.14214300
C	1.36421900	1.20308900	2.83018300
C	1.25447800	1.35185900	4.22708000
C	0.52985700	0.40548800	4.95003100
C	-0.06412000	-0.67053800	4.23204500
B	2.79255600	-0.83364900	0.47081500
O	3.88716500	-0.05669500	0.82285500
C	4.99278500	-0.92137800	1.18716200
C	4.25696800	-2.26627400	1.55154400
O	3.03416900	-2.17180400	0.77431100
B	1.78170000	-1.02503300	-1.61389400
O	2.96157300	-0.76820000	-2.30120400
C	2.95862300	-1.53734300	-3.52939000
C	2.00187300	-2.73436900	-3.16854800
O	1.12769800	-2.12656500	-2.18477200
B	1.85608500	1.75968500	-0.51018800
O	2.17360200	2.18582000	-1.79661600
C	3.05809600	3.32700300	-1.70842200
C	2.69629400	3.91752600	-0.29614700
O	2.24858600	2.73517500	0.41380700
H	-0.58717900	-2.81191300	-1.02671700
H	1.90974700	1.92981200	2.23912400
C	0.36444400	0.49909000	6.44685100
H	-0.69298200	0.58151400	6.72295800
H	0.75908800	-0.39321200	6.94586900
H	0.88144700	1.36473000	6.85987800
C	1.92964000	2.54362000	4.86590000
H	1.20875800	3.19443700	5.37293300
H	2.67531900	2.24034800	5.60919100
H	2.44136200	3.14322200	4.10985300
C	-2.08383900	-4.95465200	-0.33324600
H	-3.17848100	-4.99833200	-0.31925400
H	-1.77683300	-4.75420000	-1.36238300
H	-1.71260000	-5.94972200	-0.06411100
C	-2.58273500	-4.97433600	2.64661300
H	-2.93854400	-5.72018000	1.93640400
H	-1.98591300	-5.49539900	3.40391200
H	-3.45891000	-4.55824800	3.15693500
C	-1.38170900	-2.72856300	4.16279200
H	-1.95142600	-3.48783600	4.68644300
C	-0.82959400	-1.70275500	4.87095100
H	-0.97065900	-1.66357900	5.94492000
C	-1.02025400	1.16669400	0.13906700
H	0.60526700	0.10683300	-1.60117800
H	-0.79208000	2.14698100	0.57566600
C	2.78138200	4.25454900	-2.89092100
H	3.38877300	5.16433100	-2.82841200
H	3.03726100	3.74305800	-3.82365000
H	1.72912400	4.53970300	-2.94307100
C	1.51791300	4.90070300	-0.32958000

H	1.19466900	5.10556000	0.69530200
H	1.79387300	5.85086900	-0.79793300
H	0.66909100	4.47799500	-0.87223700
C	3.86651100	4.53220000	0.47160300
H	4.28822400	5.38414400	-0.07278500
H	3.52000900	4.89730100	1.44394900
H	4.65893000	3.80250300	0.64685900
C	4.49390900	2.78586500	-1.78532500
H	4.60247000	2.21426400	-2.71087600
H	5.23581900	3.59134100	-1.78487400
H	4.69769500	2.10862100	-0.95209100
C	5.89931400	-1.03414300	-0.04576100
H	6.24776600	-0.03500000	-0.32189300
H	6.77542700	-1.66140900	0.14769400
H	5.34864800	-1.43722000	-0.89809400
C	5.75917300	-0.26822400	2.33757600
H	6.57798000	-0.91042100	2.68036600
H	6.19281800	0.67631200	1.99639300
H	5.10792200	-0.05116300	3.18632100
C	3.83804800	-2.35289200	3.02678400
H	3.17328000	-3.21183500	3.15579400
H	4.70045500	-2.48369100	3.68812300
H	3.29489100	-1.45623700	3.33719200
C	4.99541400	-3.54318300	1.15173900
H	5.94419700	-3.63183300	1.69211800
H	4.38256500	-4.41480600	1.40027200
H	5.20403200	-3.57223300	0.08085400
C	2.39762100	-0.62252100	-4.62783200
H	3.00405800	0.28552200	-4.67404200
H	2.41801300	-1.10598400	-5.60965400
H	1.36980100	-0.32316200	-4.40645800
C	1.14612700	-3.24974700	-4.32552100
H	1.77461200	-3.65025300	-5.12841800
H	0.49829700	-4.05916700	-3.97463100
H	0.51004300	-2.46525900	-4.73924100
C	2.71805400	-3.90522600	-2.48136200
H	1.96860700	-4.61142800	-2.11181400
H	3.37843200	-4.43897800	-3.17208700
H	3.29732900	-3.55836700	-1.62308500
C	4.39498000	-1.93308800	-3.86629600
H	4.99156700	-1.03284100	-4.03998500
H	4.86206600	-2.49695200	-3.05672000
H	4.42880500	-2.54143500	-4.77668700
H	-1.64420800	0.63431100	0.86888900
Si	-1.98687000	1.41089900	-1.42178600
C	-1.16497600	2.55865300	-2.67429000
H	-1.11154900	3.57680100	-2.27442000
H	-0.14472500	2.23614700	-2.90204100
H	-1.74492600	2.60013200	-3.60219600
C	-2.52679300	-0.19346600	-2.28399400
H	-2.91800900	0.08069900	-3.27283900
H	-1.61595200	-0.77938500	-2.46902800
C	-3.56654200	-1.05056400	-1.54335100
H	-3.20846700	-1.35327500	-0.55370900
H	-4.49843400	-0.49663700	-1.39624500
Cl	-3.84093500	2.40936300	-0.95806100
H	-3.80614000	-1.96485300	-2.10075100

TSa-ISO

Ir	0.46138700	-0.47075300	-0.32761400
N	-0.96483000	-2.07738600	0.19108200
C	-1.01225200	-2.48546000	1.48960600
C	-1.88205900	-3.51238400	1.93203200
C	-2.77302300	-4.11242700	1.00141500
C	-2.74656500	-3.64484000	-0.31179700
C	-1.82534200	-2.63770300	-0.65916800
C	-0.15401400	-1.81274800	2.41867100
N	0.58775700	-0.78313500	1.93479800
C	1.41120800	-0.17241800	2.78269400

C	1.53898700	-0.49611500	4.14779100
C	0.76113800	-1.53104400	4.66767000
C	-0.11267300	-2.21161000	3.77792300
B	0.46080900	-0.84876500	-2.36902800
O	1.18843700	-0.14306600	-3.32474500
C	0.75776400	-0.54175200	-4.64621700
C	0.14815400	-1.96549000	-4.37414900
O	-0.28765000	-1.84599800	-2.99216400
B	2.11859700	-1.79134000	-0.41271800
O	3.08676600	-1.91577400	-1.41125500
C	4.16178000	-2.75893500	-0.92699800
C	3.44242500	-3.60386800	0.18708000
O	2.37927800	-2.71456400	0.60421000
B	1.81265900	1.16123100	-0.23491000
O	1.56884900	2.46575500	-0.64623200
C	2.82506800	3.18735800	-0.67213100
C	3.69334100	2.37979000	0.36515200
O	3.07994400	1.05745300	0.32291700
H	-1.76346900	-2.29494000	-1.68231400
H	2.03102800	0.60606400	2.35958000
C	0.82963500	-1.93414500	6.12001400
H	-0.14328000	-1.81509600	6.61059600
H	1.11686000	-2.98647700	6.22532700
H	1.55340100	-1.33801000	6.67514400
C	2.51268100	0.30047400	4.98485600
H	2.01380600	0.80642600	5.81871000
H	3.29723600	-0.33527900	5.41011900
H	3.00154500	1.06825400	4.38141900
C	-3.65618100	-4.17557100	-1.39578300
H	-4.71259900	-4.01910800	-1.15123700
H	-3.45890500	-3.67011900	-2.34389600
H	-3.51362100	-5.24985700	-1.55762900
C	-3.71376200	-5.20523000	1.44526200
H	-4.32953000	-5.57026400	0.62371000
H	-3.16336700	-6.06070200	1.85320300
H	-4.38872100	-4.85121700	2.23297100
C	-1.82302200	-3.89226800	3.31481400
H	-2.47134700	-4.68513400	3.66996100
C	-0.97519400	-3.28172200	4.19015000
H	-0.96036500	-3.60194700	5.22571300
C	-1.34260400	0.93644800	-0.39110000
H	0.96459600	0.57626100	-1.37661300
H	-1.08772800	1.72507600	-1.10765600
C	2.77756300	-4.88012600	-0.35110300
H	3.51472800	-5.63920700	-0.63262100
H	2.13421900	-5.29906600	0.42831800
H	2.15314500	-4.65983200	-1.22122000
C	4.30023500	-3.94049300	1.40713000
H	3.69968500	-4.49372800	2.13586800
H	5.15549000	-4.56706800	1.13046900
H	4.67317800	-3.03801600	1.89509200
C	4.72178000	-3.56677900	-2.09738600
H	5.16104000	-2.88803800	-2.83476300
H	5.50771000	-4.25251300	-1.76138500
H	3.94557500	-4.14911400	-2.59721000
C	5.24864300	-1.82469000	-0.37491000
H	6.12466900	-2.37934900	-0.02285000
H	5.56841200	-1.14952800	-1.17363500
H	4.85879200	-1.21167000	0.44097500
C	1.19294900	-3.08873500	-4.41505900
H	1.54804400	-3.27496200	-5.43358600
H	2.04595200	-2.84465300	-3.77744700
H	0.73715400	-4.00990800	-4.03900800
C	-1.06115300	-2.33320500	-5.23166500
H	-1.43167500	-3.32150100	-4.94172300
H	-1.87523300	-1.61601300	-5.11092700
H	-0.78969500	-2.37625200	-6.29189300
C	1.96870300	-0.52223800	-5.57812600
H	1.70357300	-0.88932500	-6.57573700

H	2.33250700	0.50416600	-5.68267800
H	2.78514400	-1.12982100	-5.18425200
C	-0.28278500	0.48789800	-5.11122100
H	-1.15810900	0.49122700	-4.45584400
H	0.16658100	1.48392300	-5.07252300
H	-0.61353000	0.29921200	-6.13754700
C	3.56670800	2.90370200	1.80144900
H	4.05691800	2.19989900	2.48131800
H	4.05287700	3.87676900	1.91889700
H	2.51972900	3.00542100	2.10008100
C	5.16933100	2.23037300	-0.00124800
H	5.67966900	1.63099100	0.75848200
H	5.29499100	1.73108100	-0.96293500
H	5.66027300	3.20854600	-0.04551300
C	3.35254500	3.09647500	-2.11201300
H	3.51395400	2.05721800	-2.41052600
H	2.60426500	3.52287000	-2.78547000
H	4.28691400	3.65219200	-2.23816800
C	2.55820700	4.64690700	-0.30647800
H	3.49220800	5.21809100	-0.27054000
H	1.91487200	5.09922700	-1.06626000
H	2.05346600	4.74033100	0.65664500
H	-2.14053700	0.33942600	-0.85506900
Si	-2.04715300	1.75919100	1.09808400
Cl	-3.62698600	3.10137000	0.48672900
C	-2.93748200	0.60389300	2.30781300
H	-2.23251600	-0.03102800	2.85455300
H	-3.51486300	1.17672600	3.04015100
H	-3.63514200	-0.04938000	1.77330200
C	-0.82901600	2.87596300	2.03485600
H	-0.06347200	2.21293600	2.46194900
H	-0.30942400	3.48704800	1.28664000
C	-1.41879600	3.76510600	3.14118100
H	-1.90762700	3.16855600	3.91998500
H	-0.64488600	4.36842300	3.63180500
H	-2.16987900	4.45113700	2.73895500

INT2a-ISO

Ir	1.30149300	-0.30513600	-0.54944100
N	-0.33181100	-1.60897300	0.07250900
C	-0.50520200	-1.78718800	1.41406900
C	-1.51747300	-2.63480000	1.92806500
C	-2.37078200	-3.32388700	1.02018800
C	-2.16547600	-3.13103900	-0.34507100
C	-1.13468100	-2.26463700	-0.76016500
C	0.38182500	-1.09132000	2.31199500
N	1.33887700	-0.30336000	1.75982800
C	2.16856600	0.34230100	2.56727500
C	2.12190600	0.26167300	3.97645700
C	1.14585400	-0.54022100	4.56768900
C	0.24616800	-1.24118700	3.71443800
B	1.22151400	-0.61798700	-2.56378900
O	2.33241500	-0.74712900	-3.39792500
C	1.90139700	-0.85599500	-4.77154100
C	0.41703100	-1.36235700	-4.61774800
O	0.05196700	-0.84882700	-3.30842300
B	2.47281500	-2.07144900	-0.52811700
O	3.74785400	-2.16437600	0.01850400
C	4.11269100	-3.56117200	0.13883200
C	3.16667200	-4.24427400	-0.91784000
O	2.04545600	-3.32102500	-0.96865700
B	2.33515200	1.48813400	-0.86784000
O	2.41654100	2.22197700	-2.03748000
C	3.41422400	3.26180900	-1.86336100
C	3.44323900	3.43963800	-0.29798500
O	3.03404400	2.12295700	0.15956000
H	-0.95764400	-2.08172000	-1.81355500
H	2.89885400	0.97449100	2.07283400
C	1.02338700	-0.67736600	6.06576700

H	0.03328500	-0.35999200	6.41201500
H	1.15612400	-1.71885400	6.38060800
H	1.76484100	-0.07689200	6.59207200
C	3.13153700	1.05691000	4.77111100
H	2.64752100	1.78531100	5.43111900
H	3.75624000	0.41169100	5.39867800
H	3.79600800	1.60841900	4.10181900
C	-3.00379200	-3.79989900	-1.40921100
H	-4.05871000	-3.51459000	-1.33178500
H	-2.65812100	-3.51463700	-2.40530300
H	-2.95228900	-4.89206000	-1.34123800
C	-3.45958800	-4.22719200	1.54464100
H	-4.03616500	-4.67990000	0.73862400
H	-3.04308500	-5.03878000	2.15224500
H	-4.15842200	-3.67331200	2.18155100
C	-1.63144100	-2.76081600	3.35347000
H	-2.40374600	-3.40146700	3.76340700
C	-0.79477200	-2.09891400	4.20296500
H	-0.91734100	-2.22521600	5.27272500
C	-0.24886800	1.35681700	-0.21455200
H	2.83940400	-0.05210500	-0.88497400
C	3.77331700	-4.29398800	-2.32718000
H	4.61821100	-4.98769900	-2.38252100
H	3.00861400	-4.63468700	-3.03134500
H	4.10630300	-3.30283300	-2.64507200
C	2.64160900	-5.62547600	-0.52776100
H	1.96202900	-5.99106500	-1.30343500
H	3.46243300	-6.34487500	-0.43278000
H	2.09188300	-5.59834500	0.41497200
C	5.60940700	-3.69613600	-0.13990200
H	6.17434800	-3.16063400	0.62896900
H	5.92237800	-4.74589100	-0.11967600
H	5.87547600	-3.26985400	-1.10863300
C	3.81206700	-3.98009800	1.58613500
H	4.11864900	-5.01190900	1.78542500
H	4.36133300	-3.32133400	2.26483500
H	2.74621200	-3.88406500	1.81152700
C	0.30327800	-2.89268100	-4.54510500
H	0.55378800	-3.36656800	-5.49929000
H	0.95370500	-3.29665200	-3.76496200
H	-0.72910200	-3.16252100	-4.29945900
C	-0.56802400	-0.81800800	-5.65140900
H	-1.57652600	-1.17761300	-5.42520100
H	-0.59396400	0.27272200	-5.65429500
H	-0.30622000	-1.16217100	-6.65806200
C	2.83698600	-1.82174700	-5.50141100
H	2.51069200	-1.98151400	-6.53515700
H	3.84605200	-1.39978500	-5.52907200
H	2.89182200	-2.78860000	-4.99864600
C	2.01605000	0.54012100	-5.40175800
H	1.39124500	1.26738300	-4.87957300
H	3.05340900	0.87560800	-5.31571500
H	1.74468300	0.53157700	-6.46243500
C	2.41494300	4.45181000	0.22469200
H	2.35390600	4.36580400	1.31357800
H	2.70117600	5.47943100	-0.01993500
H	1.41862500	4.26410800	-0.18224100
C	4.81919700	3.74553800	0.29324000
H	4.74211600	3.82957200	1.38176600
H	5.53957200	2.95804100	0.06520100
H	5.20686500	4.69631100	-0.08825000
C	4.72908100	2.71390500	-2.43759400
H	5.06069400	1.82670800	-1.89116200
H	4.56265900	2.42246900	-3.47795700
H	5.52846500	3.46117500	-2.41061800
C	2.97843400	4.49783600	-2.64869200
H	3.69029500	5.31912100	-2.51084600
H	2.94259800	4.25890700	-3.71552100
H	1.98809500	4.84320600	-2.34734200

H	-0.84496900	0.94091900	0.60850400
Si	-1.45546000	1.97272200	-1.48217300
Cl	-2.41255600	3.71593800	-0.63219900
C	-0.76529100	2.59296400	-3.11823300
H	-0.49570700	1.72692800	-3.72835900
H	-1.51254900	3.18567100	-3.65488100
H	0.13453700	3.19438200	-2.97629300
C	-2.89430300	0.78992200	-1.84852700
H	-3.51427400	1.26480700	-2.62065800
H	-2.45069900	-0.09554300	-2.32292300
C	-3.77575100	0.38798800	-0.65601600
H	-4.23798700	1.26715300	-0.19647500
H	-4.58314800	-0.29083600	-0.95881400
H	-3.19685900	-0.11944600	0.12341200
H	0.24523600	2.23128900	0.22333300

TS2a-ISO

Ir	1.26665400	-0.28729300	-0.38603300
N	-0.42788700	-1.51167100	0.23126800
C	-0.50748200	-1.80977400	1.56031000
C	-1.48352800	-2.69882300	2.07056000
C	-2.42369400	-3.28014400	1.17242000
C	-2.33738600	-2.93966200	-0.17729000
C	-1.31804000	-2.05813500	-0.59153300
C	0.43630400	-1.18165300	2.44812700
N	1.32227200	-0.31101300	1.89770600
C	2.20576600	0.27781300	2.69329000
C	2.28641800	0.05432300	4.08465600
C	1.38019300	-0.82895500	4.67416000
C	0.42046600	-1.46559800	3.83536800
B	1.19774700	-0.61527200	-2.40130200
O	2.26790100	-0.83881300	-3.26358600
C	1.79554000	-0.77493300	-4.62937000
C	0.26800300	-1.12505800	-4.47200300
O	-0.00672800	-0.68301600	-3.11814100
B	2.41594600	-2.02818200	-0.37076400
O	3.76975700	-2.09331300	-0.05919900
C	4.15841100	-3.48071800	0.07767500
C	3.03838200	-4.22486900	-0.74059300
O	1.92077500	-3.30535900	-0.63532900
B	1.96233800	1.66856800	-0.72835000
O	2.33021100	2.22633600	-1.96057400
C	3.36562900	3.20441600	-1.72974200
C	3.08778700	3.64298300	-0.24034600
O	2.47437500	2.45280800	0.32072100
H	-1.21218000	-1.78062300	-1.63537200
H	2.86973700	0.98424400	2.20542400
C	1.39309300	-1.11652000	6.15565600
H	0.43248100	-0.86067800	6.61698600
H	1.57107500	-2.18028500	6.35103800
H	2.16809500	-0.55231400	6.67367100
C	3.34701800	0.79391900	4.86636900
H	2.91007400	1.43636900	5.63879600
H	4.03819900	0.10588000	5.36560400
H	3.93712200	1.43023200	4.20276500
C	-3.28299500	-3.47658100	-1.22632400
H	-4.32258800	-3.20384800	-1.01396700
H	-3.03152400	-3.07512800	-2.21064900
H	-3.23768900	-4.56912000	-1.29348900
C	-3.47620300	-4.22836400	1.69176800
H	-4.13563000	-4.57938000	0.89859700
H	-3.01907500	-5.10896800	2.15758300
H	-4.10015500	-3.74772100	2.45371000
C	-1.47515700	-2.96707500	3.48139700
H	-2.20953600	-3.65208000	3.88961800
C	-0.56878900	-2.38599800	4.31952600
H	-0.59862900	-2.62183900	5.37733900
C	-0.16525400	1.58990400	-0.68090000

H	2.75986300	0.16496200	-0.66975000
C	3.36896700	-4.35876900	-2.23407500
H	4.19063800	-5.06059700	-2.40918400
H	2.48433000	-4.73316000	-2.75824900
H	3.62934100	-3.38840800	-2.66383300
C	2.60966300	-5.57968900	-0.17746200
H	1.80516800	-5.99356800	-0.79295200
H	3.44176200	-6.29250300	-0.18791700
H	2.23911400	-5.49309700	0.84587200
C	5.57876700	-3.64265600	-0.46380900
H	6.27332400	-3.06278700	0.15150500
H	5.89610400	-4.69107600	-0.43502300
H	5.65879200	-3.28033800	-1.49006800
C	4.13317700	-3.80704900	1.57895600
H	4.48164200	-4.82463400	1.78327900
H	4.79112700	-3.10716000	2.10202400
H	3.12545200	-3.69431200	1.98874000
C	-0.01501800	-2.63396500	-4.52064900
H	0.13689300	-3.04565500	-5.52332000
H	0.62374000	-3.17446000	-3.81705600
H	-1.05794300	-2.81153600	-4.23898400
C	-0.67257300	-0.38437600	-5.42171300
H	-1.70853500	-0.66810900	-5.21128600
H	-0.59001800	0.69701100	-5.30161500
H	-0.45911700	-0.63898000	-6.46567800
C	2.60167200	-1.76210300	-5.47413000
H	2.24308200	-1.77852200	-6.50939200
H	3.65201400	-1.45630100	-5.48701100
H	2.55227200	-2.77572000	-5.07223100
C	2.04278600	0.65701000	-5.12722200
H	1.52006400	1.38691600	-4.50533800
H	3.11277100	0.87117000	-5.05328400
H	1.73773400	0.78399300	-6.17112600
C	2.08174700	4.79687500	-0.12741500
H	1.81147700	4.93809500	0.92291300
H	2.50617900	5.73628800	-0.49533300
H	1.16490000	4.59288600	-0.68540200
C	4.33607000	3.96928300	0.58043900
H	4.04628600	4.23108400	1.60303300
H	5.01946300	3.11945400	0.62764200
H	4.87225300	4.82451800	0.15517600
C	4.71750300	2.49426100	-1.91060100
H	4.84877900	1.69245100	-1.17865200
H	4.74421300	2.04108100	-2.90508800
H	5.55943000	3.18875100	-1.82327300
C	3.22301200	4.31651700	-2.76946600
H	3.94649100	5.11970100	-2.59099700
H	3.41167200	3.90914600	-3.76706600
H	2.21832800	4.74333500	-2.76536900
H	0.14012300	2.48442800	-1.23134500
Si	-1.27075000	2.20966700	0.68637800
Cl	-2.21909100	3.97874700	-0.08041300
C	-2.72309400	1.06500400	1.07891300
H	-2.39231100	0.17516600	1.62402100
H	-3.46289400	1.58351100	1.69676300
H	-3.22245100	0.73821200	0.16175300
C	-0.43303600	2.75091500	2.29747500
H	-0.13552900	1.82797500	2.81177500
H	0.50479500	3.25374400	2.03991100
C	-1.27563100	3.62054200	3.24526500
H	-2.19994300	3.11472800	3.54708600
H	-0.72315800	3.86115500	4.16157500
H	-1.56274500	4.56360100	2.77159700
H	-0.78200800	1.05673700	-1.41235400

TS2a

Ir	1.22585200	-0.03919700	-0.19376900
N	-0.58362600	-1.40025200	-0.22824500
C	-0.91621100	-1.93768700	0.97369500

C	-1.99221900	-2.84786600	1.11125100
C	-2.73134000	-3.22063200	-0.04761700
C	-2.35596700	-2.67256400	-1.27352700
C	-1.27573500	-1.76799200	-1.29679800
C	-0.13349500	-1.55426000	2.12313600
N	0.90596200	-0.69939400	1.92607000
C	1.61097000	-0.31245900	2.98152300
C	1.36394800	-0.74725300	4.30099400
C	0.31946700	-1.64353800	4.52463100
C	-0.45690300	-2.06023200	3.40571400
B	3.27245100	0.19972500	-0.00730900
O	4.18729800	0.88672700	-0.79914600
C	5.53674000	0.52834300	-0.41379900
C	5.33805300	-0.02123000	1.05062400
O	3.95661000	-0.44968000	1.03530400
B	2.14855900	-1.80969200	-0.83408500
O	2.99638500	-2.04435500	-1.91166900
C	2.97836100	-3.46138700	-2.21876100
C	2.54717100	-4.09581200	-0.84530200
O	1.78196300	-3.02262000	-0.24694000
B	1.52046300	2.08481100	-0.04700600
O	1.43052800	3.01917900	-1.09986100
C	2.23604300	4.18122900	-0.80254700
C	2.40070700	4.08841400	0.76643900
O	2.23347900	2.67424700	1.02406500
H	-0.96557500	-1.32147900	-2.23522300
H	2.42464100	0.37074300	2.77344600
C	-0.00258800	-2.16727300	5.90295300
H	0.06677900	-3.26049800	5.93692700
H	0.67492400	-1.77007800	6.65834100
H	-1.02343000	-1.90036100	6.19959700
C	2.25195100	-0.22040000	5.40436000
H	1.67965100	0.32453600	6.16333100
H	2.78728900	-1.02786600	5.91612800
H	2.99964600	0.46580600	4.99959100
C	-3.06513800	-2.97875700	-2.57126800
H	-4.11036900	-2.65231800	-2.54298100
H	-2.58423700	-2.46055700	-3.40441100
H	-3.05898900	-4.05012800	-2.79907300
C	-3.88994800	-4.17934100	0.07424400
H	-4.36188900	-4.37297900	-0.88853400
H	-3.56682100	-5.14174000	0.48711000
H	-4.65885600	-3.78102900	0.74574200
C	-2.28588500	-3.35093400	2.42271400
H	-3.10872800	-4.04529600	2.54897100
C	-1.55855700	-2.97385300	3.51288800
H	-1.81731800	-3.37487600	4.48630900
C	-0.34634400	1.65815300	0.31869800
H	1.33825900	0.33112100	-1.71947900
H	-0.21398300	2.52285200	0.97752500
C	1.50751700	5.43583500	-1.29233800
H	2.10667100	6.33143600	-1.09386400
H	1.35580000	5.36905700	-2.37371700
H	0.53275500	5.56825400	-0.82142700
C	1.30753700	4.83731600	1.54905200
H	1.37550900	4.55441800	2.60379800
H	1.42818700	5.92298400	1.47776300
H	0.30368000	4.58719200	1.19762600
C	3.76606900	4.53264700	1.29148400
H	3.93329400	5.59657600	1.09111100
H	3.81256200	4.38400700	2.37497900
H	4.57635200	3.96562900	0.83339000
C	3.55027500	4.04064300	-1.58276900
H	3.31511800	3.99843400	-2.65062200
H	4.21649700	4.89347400	-1.41474400
H	4.05880500	3.11371000	-1.32157900
C	6.01995900	-0.53187600	-1.41290100
H	5.97121400	-0.10833800	-2.41997800
H	7.05321100	-0.83624600	-1.21596100

H	5.37034300	-1.40694400	-1.39775600
C	6.44172500	1.75722700	-0.51852900
H	7.46450500	1.50842000	-0.21416600
H	6.47559500	2.10182600	-1.55608700
H	6.09259500	2.58418800	0.10089700
C	5.45998600	1.05563600	2.13913300
H	5.18144500	0.61463600	3.10201300
H	6.48179500	1.43764200	2.22897700
H	4.78185800	1.88775400	1.94056800
C	6.22007100	-1.21567200	1.41675400
H	7.27848100	-0.93245100	1.42645900
H	5.95748100	-1.57193500	2.41754600
H	6.09252000	-2.04529100	0.71978300
C	1.93727900	-3.65658300	-3.33251000
H	2.20415300	-3.01757700	-4.17870900
H	1.89876300	-4.69389900	-3.68024900
H	0.93896700	-3.36574400	-2.99348000
C	1.65310000	-5.33151300	-0.95218900
H	2.17208600	-6.15127300	-1.46118000
H	1.38223900	-5.67586200	0.05057000
H	0.73036800	-5.11419400	-1.49324900
C	3.72676400	-4.38375100	0.09296500
H	3.33643500	-4.63778600	1.08266100
H	4.33930800	-5.21942600	-0.26050500
H	4.35876300	-3.49980300	0.20242500
C	4.35523800	-3.88453500	-2.72881400
H	4.58508600	-3.34855700	-3.65430600
H	5.14428100	-3.66394000	-2.00834700
H	4.37548400	-4.95829100	-2.94547900
H	-0.84092800	0.94347700	0.97963300
Si	-1.55105500	2.17984300	-1.03227400
C	-2.16486600	3.95111500	-0.79816800
H	-2.46368700	4.12917000	0.23996600
H	-1.38128600	4.66905500	-1.05846400
H	-3.03231700	4.15155000	-1.43438100
C	-1.06323700	1.87671400	-2.83143900
H	-0.09614000	2.37526300	-2.96037700
H	-0.85740800	0.80888000	-2.96357700
C	-2.07842500	2.35894600	-3.88075100
H	-3.05134800	1.87140600	-3.75727400
H	-2.24411700	3.43988100	-3.81547700
Cl	-3.33710800	1.01547100	-0.72034500
H	-1.72818800	2.14678400	-4.89756200

INT3

Ir	1.41426700	-1.05336900	-0.40503300
N	-0.41482700	-2.17600300	0.01795600
C	-0.91963900	-2.06148600	1.27910100
C	-2.08524900	-2.74792100	1.69269100
C	-2.74583000	-3.60238000	0.76377100
C	-2.19940700	-3.72468100	-0.51450900
C	-1.03815300	-2.98922000	-0.83164600
C	-0.21034200	-1.21180200	2.20172800
N	0.92214000	-0.60725000	1.74885900
C	1.59511700	0.17365400	2.58648500
C	1.21434600	0.41965100	3.92243700
C	0.05679200	-0.19277600	4.40697200
C	-0.67897400	-1.03712400	3.52566600
B	1.50821400	-1.23614600	-2.41640300
O	1.71045100	-0.15997300	-3.28751700
C	1.34825500	-0.55025400	-4.62736300
C	1.50552000	-2.11489200	-4.57295400
O	1.23689100	-2.39576200	-3.17881300
B	2.57239200	-2.66704400	-0.47979400
O	3.84667200	-2.77569700	-1.03195800
C	4.41440600	-4.04218500	-0.63317700
C	3.13287100	-4.90024300	-0.31454600
O	2.18213800	-3.88668500	0.08892300
H	-0.58651500	-3.06462500	-1.81657900

H	2.49288900	0.62861600	2.17929700
C	-0.42157300	0.01295300	5.82339500
H	-1.43908700	0.41933300	5.84153900
H	-0.44424500	-0.93487100	6.37352300
H	0.21857000	0.70017200	6.37593400
C	2.08131100	1.33020800	4.76056600
H	1.52958800	2.20933900	5.11149200
H	2.47495000	0.81702700	5.64475600
H	2.93546900	1.68818100	4.18062400
C	-2.79521600	-4.60876700	-1.58554600
H	-3.82098000	-4.31415300	-1.83416400
H	-2.20339200	-4.55170100	-2.50204700
H	-2.82334200	-5.65930700	-1.27578400
C	-3.99181700	-4.34685600	1.17752200
H	-4.39803600	-4.94631200	0.36318800
H	-3.78933800	-5.02248000	2.01670100
H	-4.77754600	-3.65566400	1.50332200
C	-2.53957800	-2.54645900	3.04030200
H	-3.43492800	-3.05827100	3.37506000
C	-1.87496600	-1.73216500	3.91056100
H	-2.25474400	-1.61137400	4.91884300
H	2.73759000	-0.18260800	-0.57215500
C	2.54585300	-5.59726800	-1.55166300
H	3.18807000	-6.40776500	-1.91188900
H	1.57435000	-6.02488200	-1.28503400
H	2.38434500	-4.87919800	-2.35953600
C	3.28940800	-5.90551900	0.82648000
H	2.33912400	-6.42214400	0.99316500
H	4.04560500	-6.66095700	0.58537400
H	3.57225700	-5.41493100	1.75981000
C	5.28334700	-4.56741400	-1.77626600
H	6.12552700	-3.88766400	-1.93675700
H	5.68800700	-5.55873300	-1.54282200
H	4.72084700	-4.62936300	-2.70945200
C	5.28744700	-3.77395400	0.60327300
H	5.82315900	-4.67020300	0.93319400
H	6.02306800	-3.00517400	0.35085800
H	4.68384300	-3.40085400	1.43547300
C	2.93843600	-2.58577200	-4.86372000
H	3.21529400	-2.43108900	-5.91173000
H	3.65643500	-2.06512400	-4.22536900
H	3.01173800	-3.65580300	-4.64720200
C	0.51550900	-2.90118000	-5.43299500
H	0.67974900	-3.97461700	-5.29535400
H	-0.51918800	-2.68047800	-5.16189900
H	0.65026500	-2.67447000	-6.49646000
C	2.27697200	0.16437900	-5.61015900
H	2.08314800	-0.15211200	-6.64147100
H	2.11070900	1.24426200	-5.55074500
H	3.32592700	-0.02521100	-5.37602600
C	-0.10205100	-0.09604300	-4.85882300
H	-0.78450800	-0.59755000	-4.16648100
H	-0.16682200	0.97993300	-4.67342700
H	-0.43785300	-0.29058600	-5.88287800

TS3

Ir	-0.21444300	-0.09868200	-0.31068500
N	0.52654000	-1.66622000	1.03600600
C	0.29227000	-1.47915400	2.36548500
C	0.79208300	-2.36522000	3.34941700
C	1.57714900	-3.48067100	2.93689300
C	1.81003000	-3.64888400	1.57162600
C	1.25936100	-2.71379700	0.66934400
C	-0.49412000	-0.33370900	2.74977500
N	-0.92247400	0.48977100	1.75819500
C	-1.66620900	1.53656900	2.08602200
C	-2.03058600	1.86428000	3.41073700
C	-1.58738500	1.04261000	4.44694000
C	-0.79575800	-0.09419100	4.11143100

B	0.44492200	-0.80348600	-2.09868300
O	0.92335900	-0.09250900	-3.20391600
C	1.39238500	-1.01557600	-4.21042200
C	0.63278500	-2.34002200	-3.83262600
O	0.44841600	-2.18162500	-2.40504500
B	1.49671000	0.95367100	-0.26673400
O	1.55851600	2.34501900	-0.18332800
C	2.89755300	2.72496900	0.20413700
C	3.74561500	1.48491100	-0.26695900
O	2.77990100	0.40702400	-0.18916200
H	1.40554300	-2.82128700	-0.40084200
H	-2.01307900	2.13838900	1.25332000
C	-1.92556100	1.32343400	5.89089800
H	-2.47751300	0.48856800	6.33791000
H	-1.01852900	1.46576800	6.48964300
H	-2.53648000	2.21914400	6.00033900
C	-2.88360200	3.09125000	3.63450900
H	-3.83343100	2.84469100	4.12191400
H	-2.37638500	3.83217000	4.26266300
H	-3.11784600	3.57221400	2.68188300
C	2.62593600	-4.78835500	1.00583800
H	2.20169500	-5.76438300	1.26653400
H	2.66563800	-4.72586900	-0.08413600
H	3.65746900	-4.77177000	1.37500600
C	2.12766400	-4.43662700	3.96676900
H	2.71878000	-5.23087600	3.51149600
H	2.77030000	-3.91649400	4.68623700
H	1.32169700	-4.90978400	4.53975300
C	0.47667400	-2.09048200	4.72327100
H	0.84920500	-2.75773300	5.49242300
C	-0.28068600	-1.01461400	5.08522700
H	-0.49608500	-0.84844400	6.13488800
C	2.89143200	2.90868100	1.73042500
H	3.85705300	3.26242000	2.10632100
H	2.12874900	3.64747900	1.99315300
H	2.64242700	1.97154600	2.23632100
C	4.93837900	1.13325500	0.62273000
H	5.43420800	0.23950900	0.23191400
H	5.67265500	1.94648100	0.63779400
H	4.62901300	0.92382800	1.64865200
C	4.19762000	1.58862000	-1.73002200
H	4.95709700	2.36519300	-1.86733200
H	4.62671300	0.63095000	-2.03674500
H	3.34837000	1.79951700	-2.38463800
C	3.24325300	4.04975600	-0.47577400
H	2.57909100	4.83611400	-0.10422000
H	4.27508600	4.34874100	-0.25946200
H	3.11556500	3.98736700	-1.55776900
C	1.05490300	-0.44373000	-5.58856800
H	1.60303400	0.49123400	-5.74045100
H	1.34190200	-1.13716600	-6.38733100
H	-0.01080800	-0.22713100	-5.68065800
C	-0.76431700	-2.43285400	-4.46464800
H	-0.70868300	-2.60136700	-5.54551200
H	-1.30558600	-3.26752900	-4.01180700
H	-1.34442100	-1.52749100	-4.27066100
C	1.40876100	-3.63297500	-4.08495100
H	0.81259800	-4.48908800	-3.75349200
H	1.61994200	-3.76486600	-5.15203300
H	2.35573100	-3.64893000	-3.54082900
C	2.91666100	-1.13092600	-4.05771300
H	3.36065600	-0.13825900	-4.16852400
H	3.18212500	-1.50619600	-3.06553600
H	3.35555400	-1.78919000	-4.81491400
B	-2.66367100	-1.45404000	-0.69163900
O	-2.72706000	-1.83230200	0.63426200
C	-3.34974000	-3.14718800	0.70892300
C	-3.17590500	-3.69779800	-0.76142300
O	-3.02100600	-2.47898600	-1.53602900

B	-2.60749300	0.16270300	-1.35119400
O	-2.67388600	0.28284900	-2.73074200
C	-3.15669200	1.60617300	-3.06464500
C	-3.90458900	2.03802700	-1.73819100
O	-3.27542000	1.21706000	-0.72113600
C	-3.73110200	3.50809300	-1.35291300
H	-4.15759000	4.16741000	-2.11667400
H	-4.25432200	3.70506200	-0.41147100
H	-2.67883800	3.76576900	-1.22023300
C	-5.39638400	1.67008600	-1.72633500
H	-5.79275100	1.83393600	-0.71993700
H	-5.97433300	2.28028100	-2.42761200
H	-5.54357700	0.61636300	-1.97846600
C	-1.93991400	2.47794800	-3.40757700
H	-1.37740800	1.99288100	-4.20926800
H	-2.24556400	3.47308500	-3.74759000
H	-1.26251600	2.58262000	-2.55844100
C	-4.05634800	1.48032500	-4.29646500
H	-4.50438700	2.44526200	-4.55783000
H	-3.45746200	1.14583600	-5.14845900
H	-4.85528600	0.75262400	-4.14294800
C	-4.81191600	-2.90325900	1.10789800
H	-4.83392000	-2.35803700	2.05567400
H	-5.33037900	-2.29647600	0.36084300
H	-5.35944000	-3.84208800	1.23674500
C	-2.64374300	-3.95889900	1.79373600
H	-2.78154600	-3.47268700	2.76403800
H	-3.06398900	-4.96840200	1.85839000
H	-1.57229400	-4.03955300	1.60437200
C	-4.38043800	-4.46132900	-1.31381200
H	-4.18296600	-4.74495900	-2.35134500
H	-4.56217000	-5.37752400	-0.74130900
H	-5.28794100	-3.85528800	-1.30003600
C	-1.89990500	-4.52451200	-0.96732700
H	-1.94090700	-5.47066600	-0.41787800
H	-1.79479500	-4.75078300	-2.03154500
H	-1.01118300	-3.96723000	-0.66601600
H	-0.65993500	1.17200900	-1.13379900

INT4

Ir	-0.16614100	-0.14588500	-0.29540300
N	0.70658700	-1.60738700	1.03870000
C	0.52550800	-1.39670200	2.37414800
C	1.07983600	-2.26221800	3.34810100
C	1.84781500	-3.38175500	2.92270900
C	2.01787600	-3.57417500	1.55129400
C	1.42795000	-2.65959300	0.65712600
C	-0.24876000	-0.25300500	2.77552000
N	-0.74357400	0.53979000	1.79101000
C	-1.46414000	1.59852500	2.13612400
C	-1.74350500	1.96160000	3.47063100
C	-1.24065300	1.16506900	4.50037500
C	-0.47145100	0.02071900	4.14715600
B	0.54230300	-0.85372100	-2.07434000
O	0.89639100	-0.09329800	-3.18746100
C	1.47126500	-0.95210300	-4.19594200
C	0.92249300	-2.36689600	-3.78246300
O	0.75498300	-2.21482200	-2.35187300
B	1.70600400	0.87258000	-0.09910300
O	1.82724000	2.25234400	0.04855400
C	3.17342300	2.56765100	0.47652600
C	3.98103100	1.30503100	-0.00261000
O	2.96078500	0.27270200	-0.01137700
H	1.53086700	-2.78774400	-0.41486800
H	-1.86381700	2.17871500	1.31138000
C	-1.49229100	1.48408600	5.95427000
H	-2.02094200	0.66430200	6.45417900
H	-0.55141100	1.63688000	6.49533500
H	-2.09202600	2.38579800	6.07619600

C	-2.57733900	3.19815800	3.71387600
H	-3.49591500	2.96941900	4.26572500
H	-2.02987700	3.95280200	4.28977100
H	-2.86822200	3.65539000	2.76515100
C	2.81076400	-4.72211000	0.96954200
H	2.40164500	-5.69390500	1.26760400
H	2.80055600	-4.67982000	-0.12199800
H	3.85795700	-4.69640200	1.29117200
C	2.44939900	-4.31715500	3.94277900
H	3.01969000	-5.11877400	3.47411300
H	3.12496900	-3.78284600	4.62050300
H	1.67312800	-4.78079800	4.56252300
C	0.83458100	-1.96036100	4.73050000
H	1.24826100	-2.61244900	5.49142500
C	0.09785100	-0.87738900	5.11088300
H	-0.06020000	-0.68880600	6.16689700
C	3.13231900	2.71851600	2.00549000
H	4.10152500	3.02060500	2.41573000
H	2.39560700	3.48450200	2.26410300
H	2.82756900	1.78318100	2.48324300
C	5.11142600	0.86076700	0.92612300
H	5.58174800	-0.04150400	0.52341000
H	5.88294500	1.63451100	1.00838100
H	4.74344500	0.62876400	1.92747200
C	4.50679300	1.43523700	-1.43837100
H	5.31339900	2.17194300	-1.51144100
H	4.89642700	0.46687300	-1.76411900
H	3.70362600	1.72024000	-2.12232900
C	3.58873700	3.89251400	-0.16335000
H	2.94443800	4.69531300	0.20776900
H	4.62466200	4.14728400	0.08749000
H	3.48975500	3.86000600	-1.24967600
C	1.01951300	-0.45598800	-5.56993200
H	1.42829900	0.54311800	-5.74958600
H	1.37755800	-1.11608900	-6.36822800
H	-0.06803200	-0.39006100	-5.62977500
C	-0.46580300	-2.66607700	-4.36835300
H	-0.42190600	-2.83009500	-5.45028000
H	-0.86025200	-3.57093200	-3.89816200
H	-1.16193500	-1.85212800	-4.15262900
C	1.86766500	-3.53899200	-4.04509700
H	1.40607700	-4.46819500	-3.69609200
H	2.07122000	-3.64840000	-5.11606000
H	2.81918900	-3.41605700	-3.52326200
C	2.99825100	-0.84308300	-4.07827500
H	3.28859500	0.20448100	-4.19650600
H	3.33753700	-1.17552300	-3.09372700
H	3.51150700	-1.42988400	-4.84739100
B	-1.85247800	-1.59046200	-0.29411400
O	-2.26514800	-2.04843400	0.95500700
C	-3.23797900	-3.10998800	0.79400800
C	-3.01365300	-3.54516700	-0.70601600
O	-2.42132400	-2.36047200	-1.29458100
B	-1.90924500	0.49502000	-1.27803900
O	-2.20612700	0.35860300	-2.63035900
C	-3.15953600	1.37822400	-2.99747300
C	-3.88474200	1.65720900	-1.62900900
O	-2.84872900	1.34148100	-0.66629100
C	-4.32880500	3.10299900	-1.40714800
H	-5.06639900	3.40738400	-2.15779900
H	-4.79567700	3.19767100	-0.42150100
H	-3.48457800	3.79379200	-1.44927100
C	-5.05830300	0.70258600	-1.36906700
H	-5.37416100	0.80304100	-0.32642200
H	-5.91624500	0.92573900	-2.01139400
H	-4.75433600	-0.33326900	-1.53866800
C	-2.35922100	2.58412500	-3.51550000
H	-1.70878600	2.24851200	-4.32739700
H	-3.01220100	3.37659900	-3.89589800

H	-1.72065100	3.00314600	-2.73288300
C	-4.05398800	0.83188100	-4.11021700
H	-4.84639500	1.54420000	-4.36540700
H	-3.45406100	0.65991100	-5.00877900
H	-4.51106800	-0.11765900	-3.82663300
C	-4.62097800	-2.50292900	1.06681400
H	-4.62229500	-2.06639100	2.06988400
H	-4.85308400	-1.70677000	0.35673700
H	-5.41334500	-3.25712900	1.01968300
C	-2.94160100	-4.19158100	1.83524300
H	-3.09325300	-3.78302000	2.83897200
H	-3.61132500	-5.05034700	1.71574700
H	-1.90987100	-4.54159100	1.76834000
C	-4.28493800	-3.88827600	-1.48260000
H	-4.02644800	-4.12351500	-2.51907700
H	-4.78666200	-4.76168900	-1.05153900
H	-4.98893900	-3.05420000	-1.49418700
C	-1.98817800	-4.67752000	-0.86642500
H	-2.36770400	-5.62941700	-0.48112000
H	-1.76165000	-4.79988400	-1.92861400
H	-1.05237700	-4.43718100	-0.35540900
H	-0.34114900	1.21676100	-1.11050500

TS4

Ir	-0.24962500	-0.15226800	-0.29587200
N	0.54821300	-1.56512900	1.18798200
C	0.32478100	-1.25552900	2.49851000
C	0.80425100	-2.07515900	3.55071200
C	1.53360800	-3.25460700	3.23221200
C	1.73756000	-3.55285400	1.88463900
C	1.22341600	-2.67927100	0.90645600
C	-0.43088400	-0.06677400	2.79035000
N	-0.88486700	0.65809700	1.73708400
C	-1.58788900	1.75565100	1.98537600
C	-1.89023900	2.22370000	3.28133100
C	-1.43666100	1.49338300	4.38021700
C	-0.68890300	0.30867800	4.13219900
B	0.49885800	-0.96266000	-2.01847300
O	0.36626300	-0.45501800	-3.30795700
C	1.24243900	-1.17077300	-4.20233300
C	1.47370700	-2.52467200	-3.43311300
O	1.29240100	-2.12064100	-2.05724400
B	1.66060400	0.84300400	-0.06801900
O	2.03295300	1.45839400	1.12350700
C	3.45192400	1.75682100	1.07496700
C	3.72766500	1.79419000	-0.47327500
O	2.70039900	0.90744100	-0.98540200
H	1.36465300	-2.87735700	-0.15091300
H	-1.94188300	2.28498400	1.10628400
C	-1.71943500	1.92365300	5.79927000
H	-2.30228200	1.16563300	6.33547800
H	-0.79027000	2.07172700	6.36123600
H	-2.27971300	2.85760100	5.83806500
C	-2.69667000	3.49521600	3.41099600
H	-3.63662600	3.32998300	3.94936900
H	-2.14531100	4.27467700	3.94886800
H	-2.94696000	3.89147700	2.42398000
C	2.49213000	-4.77512100	1.41393600
H	2.01476500	-5.70307800	1.74843800
H	2.53520700	-4.80011600	0.32275000
H	3.52265400	-4.78647000	1.78619100
C	2.05826000	-4.14014700	4.33607800
H	2.59256900	-5.00558900	3.94478100
H	2.74729200	-3.59286700	4.98965600
H	1.24337300	-4.51161200	4.96799800
C	0.52603100	-1.66767000	4.89926900
H	0.88834600	-2.27911400	5.71796600
C	-0.18044800	-0.53441700	5.17591900
H	-0.36619200	-0.26522400	6.20977600

C	4.17550500	0.60780800	1.79299400
H	5.25445400	0.78132800	1.85836900
H	3.77948500	0.52076300	2.80894200
H	4.00582700	-0.34355600	1.28118100
C	5.09357200	1.26237800	-0.90542000
H	5.17665800	1.30612300	-1.99540400
H	5.90329400	1.86644800	-0.48113400
H	5.23526600	0.22427500	-0.59935900
C	3.47693000	3.17250000	-1.10306200
H	4.24987500	3.89676700	-0.82602900
H	3.47672500	3.06692600	-2.19139300
H	2.50243400	3.56842500	-0.80437600
C	3.69873500	3.07078500	1.81486800
H	3.44395900	2.95022800	2.87227100
H	4.75206300	3.36610700	1.75316000
H	3.08579300	3.87922100	1.41255600
C	0.54724900	-1.30961200	-5.55798300
H	0.40388800	-0.31744500	-5.99669200
H	1.15177800	-1.90153600	-6.25467900
H	-0.43411900	-1.77789300	-5.46034500
C	0.40584000	-3.58606600	-3.73630400
H	0.49768600	-3.97947600	-4.75403700
H	0.52291800	-4.41663000	-3.03392500
H	-0.59727300	-3.17702400	-3.59753300
C	2.87071100	-3.12772800	-3.58319500
H	2.94268500	-4.04450500	-2.98864200
H	3.07810100	-3.38992100	-4.62666800
H	3.64267500	-2.43806800	-3.23658100
C	2.52045000	-0.33117200	-4.35348100
H	2.24590700	0.65448300	-4.74123000
H	3.00317700	-0.18281500	-3.38481800
H	3.23194000	-0.78732400	-5.05025600
B	-1.86725000	-1.61196900	-0.33458900
O	-3.11939300	-1.50935800	0.27029800
C	-3.88494400	-2.71196800	-0.00845300
C	-2.75729900	-3.75042500	-0.36631700
O	-1.68782200	-2.89579400	-0.83762100
B	-1.82182100	0.68519700	-1.39731800
O	-2.47859900	0.19768200	-2.51876600
C	-3.26195500	1.26632800	-3.10188600
C	-3.52189600	2.19612500	-1.86107700
O	-2.34831300	1.93938300	-1.05151800
C	-3.58063200	3.69134600	-2.17203600
H	-4.41909700	3.91978600	-2.83922300
H	-3.72546300	4.25679800	-1.24606100
H	-2.65834900	4.04132700	-2.63899900
C	-4.74409000	1.77974600	-1.03065800
H	-4.76041000	2.36412500	-0.10533100
H	-5.68285600	1.96235000	-1.56339900
H	-4.68411700	0.72365100	-0.75746400
C	-2.38697500	1.92052800	-4.18171300
H	-2.06772000	1.14781400	-4.88535600
H	-2.92877100	2.69630400	-4.73292700
H	-1.48561800	2.35947300	-3.74673300
C	-4.51492500	0.67034100	-3.74172900
H	-5.15090800	1.45750800	-4.16189000
H	-4.22490000	0.00083800	-4.55707600
H	-5.10173900	0.09453400	-3.02453800
C	-4.81071000	-2.40083100	-1.19183800
H	-5.47080700	-1.57219300	-0.91883700
H	-4.23291800	-2.09891600	-2.06830800
H	-5.43693400	-3.25951600	-1.45440900
C	-4.71705900	-3.05143200	1.22814300
H	-5.43763600	-2.24953800	1.41516700
H	-5.27774500	-3.98160800	1.08287100
H	-4.09403500	-3.15572500	2.11848100
C	-3.10890800	-4.74290500	-1.47511100
H	-2.24772000	-5.38774100	-1.67430700
H	-3.94953500	-5.38241300	-1.18303900

H	-3.36502700	-4.23144300	-2.40443800
C	-2.21823500	-4.51074700	0.85524800
H	-2.94435200	-5.23372300	1.24095700
H	-1.31530600	-5.05360100	0.56129500
H	-1.95012700	-3.82289800	1.66180700
H	0.01208200	1.24329500	-1.03779700

INT5

Ir	-0.46562000	-0.40416300	-0.38777900
N	0.83643900	-1.49495400	1.10591700
C	0.66078200	-1.17316600	2.41418300
C	1.34827300	-1.84905400	3.45256600
C	2.22889200	-2.91608000	3.11198300
C	2.36653800	-3.24977200	1.76463000
C	1.64547700	-2.50323600	0.80898800
C	-0.26822200	-0.11219900	2.72369700
N	-0.93197200	0.46882800	1.68890600
C	-1.79103800	1.44430900	1.95975400
C	-2.06162700	1.92007900	3.26189000
C	-1.40002000	1.33295800	4.33969700
C	-0.47554100	0.28319100	4.06808000
B	0.17783600	-1.22174200	-2.12851000
O	0.86513000	-0.49769900	-3.11171800
C	1.44797700	-1.40976100	-4.06176500
C	0.55106400	-2.68957700	-3.88349900
O	0.14251000	-2.57944600	-2.49887800
B	1.73257900	1.37077700	-0.28152200
O	1.80401100	2.38306100	0.63523200
C	3.18199600	2.45297600	1.10891300
C	3.97058200	1.68504400	-0.02790100
O	2.94278400	0.82537200	-0.60181600
H	1.71289700	-2.75457400	-0.24466100
H	-2.30070600	1.86263700	1.09570400
C	-1.64366500	1.77372200	5.76287000
H	-2.02245900	0.94643700	6.37406700
H	-0.71861300	2.12430600	6.23492400
H	-2.37004400	2.58412000	5.81923500
C	-3.06553600	3.03908900	3.41687900
H	-3.92159700	2.73739900	4.03094900
H	-2.62167200	3.92339200	3.88771200
H	-3.45054300	3.34379000	2.44095100
C	3.24701200	-4.37633100	1.27580700
H	2.94375900	-5.34077300	1.69782500
H	3.19002100	-4.46196100	0.18806700
H	4.29877800	-4.21981400	1.54082500
C	2.96995900	-3.65779600	4.19795000
H	3.59881200	-4.45059200	3.79348600
H	3.61532500	-2.98265300	4.77181700
H	2.27383900	-4.11769200	4.90872200
C	1.11640800	-1.42189200	4.80332400
H	1.63904400	-1.92108300	5.61163100
C	0.25385100	-0.40593500	5.09485500
H	0.10911000	-0.11548400	6.12948500
C	3.22115700	1.74548300	2.46880200
H	4.21336200	1.81091700	2.92593300
H	2.50175500	2.22430300	3.13866500
H	2.94747500	0.69159000	2.38004300
C	5.11829700	0.80230000	0.45757600
H	5.56667300	0.28673100	-0.39634300
H	5.89759300	1.40344800	0.93789500
H	4.77408900	0.04561100	1.16455200
C	4.45205100	2.59911800	-1.16222700
H	5.26906000	3.25089500	-0.83802200
H	4.81148100	1.97885800	-1.98749900
H	3.63819100	3.22245300	-1.54224500
C	3.55070300	3.92625900	1.27679800
H	2.92246900	4.37247100	2.05304400
H	4.59638700	4.03617600	1.58315700
H	3.39596300	4.48921700	0.35501500

C	1.39657600	-0.76787900	-5.44925800
H	2.02974500	0.12483700	-5.46358600
H	1.76382700	-1.45542900	-6.21982900
H	0.38107700	-0.46227000	-5.70666300
C	-0.72570400	-2.64579000	-4.73668300
H	-0.50668400	-2.75165000	-5.80449400
H	-1.38012400	-3.46967200	-4.43840700
H	-1.26687600	-1.71078300	-4.57013400
C	1.26891100	-4.02472500	-4.07883700
H	0.57231900	-4.84764200	-3.89110700
H	1.64114600	-4.12598500	-5.10452200
H	2.11153200	-4.13480500	-3.39264000
C	2.91179800	-1.63075700	-3.64680800
H	3.40640300	-0.65813400	-3.57645700
H	2.97320200	-2.10394300	-2.66285500
H	3.45617100	-2.24981900	-4.36806800
B	-1.81112500	-1.90791200	-0.25473400
O	-1.60659200	-3.01148500	0.58605300
C	-2.82858000	-3.77622800	0.66370300
C	-3.56987400	-3.35253300	-0.65716800
O	-3.05759200	-2.01748100	-0.87448700
B	-1.84803700	0.56456100	-1.54298700
O	-2.18766400	0.34875500	-2.88020100
C	-3.08093800	1.38566400	-3.33204600
C	-3.70836900	1.89474300	-1.98290100
O	-2.64735500	1.61466300	-1.04012000
C	-4.02707700	3.38919000	-1.93474700
H	-4.77885900	3.65699900	-2.68560600
H	-4.43175300	3.64911800	-0.95100000
H	-3.13574500	3.99687400	-2.10252100
C	-4.93329400	1.07866000	-1.54149200
H	-5.20111600	1.36703700	-0.51986100
H	-5.80093800	1.26242200	-2.18393100
H	-4.70436700	0.01024900	-1.54047200
C	-2.22254400	2.44692000	-4.03881000
H	-1.64853700	1.96078700	-4.83252100
H	-2.83156200	3.23991300	-4.48607300
H	-1.51097000	2.90056800	-3.34348200
C	-4.07619600	0.77689300	-4.32056700
H	-4.82425100	1.51346100	-4.63512400
H	-3.54381300	0.43521500	-5.21359200
H	-4.58938300	-0.08278800	-3.88637900
C	-3.56122700	-3.32789600	1.93843400
H	-2.89487600	-3.46494100	2.79534800
H	-3.82699800	-2.26852000	1.88253100
H	-4.47305700	-3.90769300	2.11548900
C	-2.47103400	-5.25943000	0.76311500
H	-1.93508500	-5.44658700	1.69909100
H	-3.37050900	-5.88531100	0.75873100
H	-1.82629600	-5.56836900	-0.06155400
C	-5.09344300	-3.28435400	-0.55459600
H	-5.51178700	-2.95916900	-1.51197200
H	-5.51650000	-4.26767400	-0.31974100
H	-5.41465800	-2.57470600	0.21044400
C	-3.16171800	-4.19527400	-1.87480400
H	-3.55172600	-5.21696300	-1.81501000
H	-3.56492200	-3.72402700	-2.77571000
H	-2.07408700	-4.22763900	-1.97559800
H	0.69902200	1.12277200	-0.86461200

2a’

B	0.47707100	9.08253100	-1.22377300
O	0.94171000	9.76863700	-2.32643700
C	2.34215100	10.09432000	-2.09066800
C	2.43892500	10.03219200	-0.51573100
O	1.36313000	9.11272500	-0.17477100
C	-0.91275000	8.34893400	-1.17148200
H	-0.87605700	7.57303100	-1.95366000
C	2.11208600	11.36508600	0.17058300

H	2.00056000	11.19251200	1.24442800
H	2.90617300	12.10256700	0.02059900
H	1.17379200	11.78667000	-0.20112300
C	3.74724800	9.46711500	0.03453700
H	3.69851400	9.43027400	1.12647500
H	3.93400700	8.45477700	-0.32771700
H	4.59512000	10.10134300	-0.24491000
C	3.16691100	9.01048100	-2.79708200
H	2.97164300	8.02190900	-2.37223100
H	2.88657500	8.98486800	-3.85361100
H	4.24031400	9.21085400	-2.73053500
C	2.63217200	11.46062900	-2.70836000
H	3.66291300	11.76945700	-2.50517100
H	2.50390200	11.40758300	-3.79328200
H	1.95737400	12.22876500	-2.32651800
Si	-2.29281900	9.50584000	-1.76932900
Cl	-4.13342900	8.48417000	-1.53235400
C	-2.42160200	11.06906400	-0.72688900
H	-1.52283100	11.68510000	-0.84784000
H	-3.28437200	11.66977400	-1.03073600
H	-2.53263400	10.83469800	0.33622500
C	-2.12961100	9.89717400	-3.59956500
H	-1.15694600	10.35947100	-3.79398100
H	-2.19763100	8.98524400	-4.20105000
C	-1.18746800	7.68361800	0.19700900
H	-0.37663300	7.00144100	0.47149400
H	-1.25508100	8.42986000	0.99548700
H	-2.12232900	7.11640600	0.19136700
H	-2.91988100	10.57875800	-3.92944000

TS1a’

Ir	1.00106100	-0.00094400	0.22257300
N	-0.18996700	-1.79833800	0.96835100
C	-0.43115700	-1.78833800	2.30535100
C	-1.15208400	-2.82495100	2.94792700
C	-1.65315300	-3.90158100	2.16258100
C	-1.40257500	-3.88461800	0.79035200
C	-0.66066300	-2.81202700	0.25241700
C	0.07487300	-0.67365400	3.06464300
N	0.74025200	0.30647900	2.39195600
C	1.23207500	1.32536300	3.08964000
C	1.08819700	1.47418200	4.48303600
C	0.39540900	0.49254000	5.19215000
C	-0.12446000	-0.61536400	4.46617400
B	2.85551600	-0.84660000	0.76649400
O	4.00330400	-1.02200900	-0.00611700
C	4.98675200	-1.76426400	0.75671300
C	4.52395700	-1.49959100	2.23538600
O	3.10181900	-1.28540200	2.07053300
B	1.64276700	-0.81662500	-1.58888700
O	2.15688400	-0.15535100	-2.69643000
C	2.62551200	-1.12142100	-3.66719600
C	1.85369400	-2.43480900	-3.24686600
O	1.53903000	-2.18344800	-1.85389100
B	2.07756400	1.68579200	-0.19145600
O	1.85105800	2.57896100	-1.23686100
C	2.95405600	3.50654800	-1.31922200
C	3.49131300	3.51038900	0.15899600
O	3.11443300	2.18974700	0.62176500
H	-0.41985300	-2.78422000	-0.80379600
H	1.79815900	2.04957500	2.51531900
C	0.19555800	0.57823700	6.68556800
H	-0.86978900	0.60144000	6.94246000
H	0.62966700	-0.29061200	7.19315300
H	0.65597800	1.47101900	7.10798900
C	1.69600800	2.69286300	5.13843100
H	0.93952600	3.30677100	5.63979200
H	2.44532600	2.41967300	5.88960000
H	2.19034800	3.32198900	4.39451300

C	-1.88547200	-4.96168700	-0.15269900
H	-2.97778900	-5.04584300	-0.14811600
H	-1.57852600	-4.73866000	-1.17708200
H	-1.47968400	-5.94532000	0.10908000
C	-2.43082900	-5.01683100	2.81842300
H	-2.74055900	-5.77417800	2.09876000
H	-1.83331700	-5.51669600	3.58929900
H	-3.33448900	-4.63593100	3.30809100
C	-1.34358100	-2.73255700	4.36725300
H	-1.89140700	-3.51652200	4.87792900
C	-0.85247000	-1.68483500	5.08849400
H	-1.01750000	-1.65553700	6.15952000
C	-1.04140100	1.22999600	-0.24097400
H	0.16504700	0.25837300	-1.11501400
H	-0.63117100	2.20821500	0.03493500
C	2.42864600	4.85005100	-1.82593100
H	3.22361000	5.60379700	-1.84606000
H	2.05226600	4.73180300	-2.84630800
H	1.60958100	5.22374700	-1.20792100
C	2.77822500	4.53225200	1.05762900
H	3.06728700	4.35457600	2.09865300
H	3.05110700	5.56171600	0.80506400
H	1.69157900	4.43323900	0.98111500
C	5.00549200	3.66497800	0.30138300
H	5.34201000	4.62845600	-0.09729100
H	5.28432600	3.62288400	1.35908700
H	5.53841600	2.86788500	-0.21959400
C	3.95968900	2.92634700	-2.32542900
H	3.44521400	2.76139300	-3.27538000
H	4.80269500	3.60414400	-2.49597400
H	4.33967800	1.95993100	-1.98550200
C	4.86851500	-3.23837800	0.34329700
H	5.03598900	-3.32204900	-0.73409800
H	5.60856900	-3.86498800	0.85141900
H	3.86959100	-3.62599700	0.55913900
C	6.37609500	-1.23186600	0.40542600
H	7.14919500	-1.71916500	1.01019800
H	6.59459100	-1.43597400	-0.64713600
H	6.44043800	-0.15326800	0.55884800
C	5.10334900	-0.20658400	2.82915100
H	4.60056300	-0.00112700	3.77938100
H	6.17784600	-0.28933800	3.02242700
H	4.92237200	0.64109600	2.16321200
C	4.73419800	-2.66276400	3.20412900
H	5.79879700	-2.89866300	3.31183500
H	4.34820600	-2.39131700	4.19155200
H	4.20968200	-3.56137900	2.87393000
C	2.29224900	-0.60017500	-5.06644700
H	2.83459400	0.33288900	-5.24569700
H	2.59613700	-1.31888100	-5.83555000
H	1.22636000	-0.39606100	-5.18190800
C	0.52204800	-2.63018500	-3.98551200
H	0.67567300	-2.85385200	-5.04597000
H	-0.01151300	-3.47508600	-3.53856000
H	-0.12051900	-1.75103100	-3.90010000
C	2.67606900	-3.72205900	-3.32692900
H	2.07445600	-4.56298000	-2.96831300
H	2.97209400	-3.93666400	-4.35960900
H	3.57444400	-3.66485600	-2.71062300
C	4.14889000	-1.21893100	-3.50189600
H	4.58483400	-0.23349100	-3.69046500
H	4.40856000	-1.50573200	-2.48140600
H	4.59122600	-1.92896900	-4.20819700
Si	-1.76250400	1.47207800	-1.95872600
C	-3.29076200	2.58926600	-1.94454700
H	-4.08474100	2.19949800	-1.30136700
H	-3.02379700	3.59036400	-1.58435400
H	-3.69890900	2.69473100	-2.95509100
C	-0.57462700	2.07346200	-3.28106000

H	0.34648900	1.48714700	-3.30124500
H	-1.05644100	2.03535300	-4.26405400
Cl	-2.49873000	-0.42601100	-2.62183100
H	-0.29266900	3.11089800	-3.07314000
C	-2.13079700	0.79551100	0.75015400
H	-1.77270500	0.78135600	1.78314100
H	-2.99662500	1.47413800	0.72741100
H	-2.50966200	-0.20458700	0.51728100

INT2a'

Ir	0.82467800	0.06815700	-0.01852100
N	-0.33679100	-1.79839400	0.73522200
C	-0.42252700	-1.86370800	2.08789400
C	-1.07759700	-2.92862500	2.75521300
C	-1.67075300	-3.96340700	1.97857000
C	-1.57395600	-3.87545100	0.59067400
C	-0.89255200	-2.77414900	0.02909100
C	0.17028000	-0.79075300	2.84133200
N	0.77812800	0.21723200	2.14989100
C	1.30304600	1.22881500	2.84070500
C	1.27716400	1.32051100	4.24610400
C	0.67564700	0.29694900	4.97756800
C	0.10237500	-0.78799600	4.25701600
B	2.72107700	-0.79609300	0.49023000
O	3.82024500	-0.02459400	0.84141000
C	4.92498500	-0.89472800	1.19737400
C	4.18651500	-2.23909200	1.55968600
O	2.95663500	-2.13499300	0.79426500
B	1.70114800	-1.00033700	-1.60224500
O	2.89170500	-0.77012000	-2.27981200
C	2.88540600	-1.54629900	-3.50376200
C	1.90241600	-2.72223200	-3.14492100
O	1.02981800	-2.09021900	-2.17420900
B	1.82732800	1.79098400	-0.47931100
O	2.25529700	2.17605700	-1.74529800
C	3.11311000	3.33442600	-1.62419800
C	2.62754800	3.96556600	-0.26717600
O	2.13053000	2.80166200	0.44098900
H	-0.77401800	-2.69647700	-1.04431300
H	1.76393700	2.00857300	2.24526200
C	0.61289700	0.32281900	6.48486800
H	-0.42509800	0.34249800	6.83651500
H	1.08256600	-0.56794400	6.91683100
H	1.11743300	1.19589400	6.89774700
C	1.90874300	2.53531400	4.88648300
H	1.18185800	3.10984400	5.47117900
H	2.72736300	2.26137200	5.56132800
H	2.31850300	3.19988500	4.12269800
C	-2.16485400	-4.90305400	-0.34637600
H	-3.25048400	-4.98758300	-0.22541800
H	-1.97071700	-4.62999000	-1.38628200
H	-1.73919700	-5.89901500	-0.18029900
C	-2.38005400	-5.10788300	2.66067600
H	-2.76149000	-5.83520500	1.94453500
H	-1.70913100	-5.63783500	3.34615000
H	-3.22947800	-4.74965100	3.25370700
C	-1.11894100	-2.90435400	4.18883200
H	-1.61339800	-3.71204600	4.71653900
C	-0.55846500	-1.88675900	4.90189100
H	-0.61715700	-1.90241100	5.98410300
C	-1.09798700	1.31064500	0.03871200
H	0.57507900	0.15386200	-1.59857600
H	-0.74593500	2.32714600	0.26065100
C	2.92234400	4.21776400	-2.85671400
H	3.50566600	5.14146300	-2.77265200
H	3.26704900	3.68239000	-3.74646700
H	1.87381000	4.47920700	-3.00885700
C	1.44803000	4.93434300	-0.43024500
H	1.04115500	5.17060800	0.55746500

H	1.75360600	5.87109100	-0.90714000
H	0.64827400	4.48559800	-1.02438900
C	3.72366300	4.61576600	0.57663900
H	4.17405800	5.46403100	0.04971500
H	3.29502900	4.99357300	1.51057700
H	4.51112500	3.90309600	0.82750300
C	4.55782300	2.81525600	-1.56714400
H	4.74670500	2.21409500	-2.46062200
H	5.28580200	3.63279100	-1.53758500
H	4.70311900	2.17109900	-0.69654900
C	5.82634000	-1.00528800	-0.03945700
H	6.17891000	-0.00662700	-0.31173600
H	6.69986700	-1.63792600	0.14808900
H	5.27065300	-1.40107300	-0.89188200
C	5.69781600	-0.24864600	2.34755500
H	6.51505200	-0.89541500	2.68545600
H	6.13473100	0.69506300	2.00827000
H	5.05026000	-0.03167100	3.19915800
C	3.78197000	-2.33513000	3.03806100
H	3.11400000	-3.19148400	3.16756500
H	4.65062300	-2.47544400	3.68923900
H	3.24681200	-1.43845700	3.36128900
C	4.91573600	-3.51617600	1.14353900
H	5.86973400	-3.61260100	1.67328800
H	4.30187200	-4.38696800	1.39249400
H	5.11310800	-3.53857300	0.07037300
C	2.35266100	-0.62742200	-4.61267400
H	2.97530700	0.26972700	-4.65590800
H	2.37612500	-1.11596800	-5.59190100
H	1.32739200	-0.30976600	-4.40531900
C	1.04945700	-3.22945300	-4.30735100
H	1.67916000	-3.65063100	-5.09861800
H	0.38010300	-4.02149400	-3.95730000
H	0.43588000	-2.43539000	-4.73647700
C	2.58824200	-3.90110100	-2.44112000
H	1.82094500	-4.58947800	-2.07461500
H	3.24523400	-4.45312000	-3.12060900
H	3.16508300	-3.55866900	-1.57943300
C	4.31694700	-1.97120900	-3.82541000
H	4.93182100	-1.08329300	-3.99875900
H	4.76614900	-2.53937700	-3.00879300
H	4.34731300	-2.58504000	-4.73222400
Si	-1.86685200	1.36450800	-1.65872700
C	-0.91836000	2.35666900	-2.95510000
H	-0.91601500	3.41814800	-2.68596100
H	0.12157400	2.03429900	-3.05212800
H	-1.41456100	2.26768100	-3.92754100
C	-2.36546100	-0.32179700	-2.35455400
H	-2.96525700	-0.19885100	-3.26206000
H	-1.48023900	-0.91745800	-2.60126600
Cl	-3.75227200	2.41541700	-1.54534800
C	-2.12236600	0.92161700	1.12164300
H	-2.52694900	-0.08614200	0.97087600
H	-1.69790100	0.94927000	2.13123700
H	-2.98019200	1.60627000	1.12548200
H	-2.96587400	-0.88398100	-1.63191200

TSa-ISO'

Ir	0.75236200	-0.14554200	-0.61050100
N	-0.80784700	-1.55354500	-0.05040100
C	-1.05674700	-1.73640700	1.27917600
C	-2.05799600	-2.62176100	1.75090400
C	-2.82831700	-3.36290800	0.81552000
C	-2.54383900	-3.18145900	-0.53746600
C	-1.53358000	-2.27514400	-0.90938200
C	-0.24613200	-1.00474300	2.20480600
N	0.71460300	-0.20472300	1.67767000
C	1.51554100	0.42982300	2.52628900
C	1.40766200	0.36597300	3.93009300

C	0.40427000	-0.42529200	4.48852800
C	-0.43945900	-1.14656100	3.60133600
B	0.76216100	-0.56632900	-2.64402400
O	1.64179000	0.01398900	-3.55818200
C	1.30751100	-0.40076500	-4.90171200
C	0.47789900	-1.71417400	-4.64578600
O	-0.05598300	-1.48147600	-3.31273300
B	2.16312300	-1.71641700	-0.54452200
O	3.15752600	-2.05007100	-1.46641000
C	4.04109200	-3.03419500	-0.87166600
C	3.11727300	-3.69022500	0.21707200
O	2.20145900	-2.61300900	0.52731200
B	2.36543900	1.29834800	-0.53841800
O	2.33615600	2.62172800	-0.94052300
C	3.69182300	3.13411900	-0.94336300
C	4.41449900	2.17953400	0.08453700
O	3.58212400	0.98107000	0.04009200
H	-1.29403700	-2.14016500	-1.95532100
H	2.30753000	1.01439100	2.08047300
C	0.21092400	-0.54113800	5.98044100
H	-0.80855000	-0.26274200	6.26930500
H	0.37343500	-1.57005200	6.32225900
H	0.89664700	0.10151300	6.53195800
C	2.38523600	1.16110400	4.76410400
H	1.87631000	1.90996300	5.38128300
H	2.96098500	0.51850500	5.43914100
H	3.09627500	1.69061000	4.12664800
C	-3.27656600	-3.91069300	-1.64027800
H	-4.34847600	-3.68428400	-1.63323300
H	-2.88574100	-3.62045400	-2.61814000
H	-3.17114300	-4.99746500	-1.54963900
C	-3.90576700	-4.30547200	1.29117100
H	-4.41117600	-4.79712500	0.46042000
H	-3.49257200	-5.08780500	1.93823400
H	-4.66672600	-3.77378700	1.87368300
C	-2.24506700	-2.73033600	3.16961600
H	-3.01602500	-3.39198400	3.54735900
C	-1.47842300	-2.02770300	4.05017500
H	-1.65044900	-2.14293900	5.11426700
C	-0.64612900	1.74335700	-0.56126100
H	1.60283000	0.74092200	-1.60982800
C	2.27345500	-4.85503200	-0.32369700
H	2.87944300	-5.74541500	-0.52033300
H	1.51395800	-5.11442600	0.41961700
H	1.76080100	-4.57004000	-1.24650300
C	3.82491900	-4.11520600	1.50367600
H	3.09517000	-4.53330600	2.20401600
H	4.57983200	-4.88393500	1.30428300
H	4.31048400	-3.26758200	1.99068300
C	4.53424200	-3.98214400	-1.96460600
H	5.13003200	-3.42364500	-2.69280000
H	5.16982900	-4.76912900	-1.54323700
H	3.70735400	-4.45432200	-2.49851000
C	5.23059000	-2.26710700	-0.27492400
H	5.97255000	-2.94155900	0.16532000
H	5.71502800	-1.69544400	-1.07151500
H	4.89600700	-1.55679900	0.48478000
C	1.34922900	-2.97473000	-4.55900500
H	1.76254100	-3.24894700	-5.53470100
H	2.16831800	-2.83032200	-3.85055800
H	0.73347600	-3.80756200	-4.20515000
C	-0.69106200	-1.94317100	-5.60239600
H	-1.22037800	-2.86101800	-5.32707600
H	-1.40590400	-1.11902700	-5.57419400
H	-0.33387400	-2.06066100	-6.63112100
C	2.60692700	-0.60116800	-5.68262900
H	2.40600200	-0.98756800	-6.68790300
H	3.12039200	0.35913700	-5.78781600
H	3.28083300	-1.28841200	-5.16862700

C	0.48684300	0.72977000	-5.53824500
H	-0.44671800	0.89698100	-4.99637500
H	1.06943100	1.65419300	-5.49561400
H	0.25080400	0.52057600	-6.58656100
C	4.39505600	2.70928000	1.52339600
H	4.73530200	1.91884900	2.19929800
H	5.06403400	3.56691800	1.64151000
H	3.39042200	3.01876300	1.82428400
C	5.83933300	1.77688500	-0.29493500
H	6.24555000	1.09977200	0.46212100
H	5.86706700	1.26127200	-1.25588900
H	6.49164900	2.65516000	-0.34827300
C	4.20845600	2.98717500	-2.38238300
H	4.20170100	1.94194000	-2.70291500
H	3.54480000	3.54276600	-3.05012900
H	5.22166500	3.38583900	-2.49255700
C	3.65378900	4.61091100	-0.55264700
H	4.66524200	5.02802000	-0.50047700
H	3.09539600	5.17049900	-1.30793300
H	3.16005000	4.76587600	0.40815800
H	-0.18005100	2.36352800	-1.33685800
Si	-2.40147800	1.55143400	-1.11661300
Cl	-3.31371600	3.51256800	-1.24772900
C	-2.63067900	0.87814000	-2.86489300
H	-2.13632300	-0.08853200	-2.99102900
H	-3.69358100	0.77146300	-3.10544400
H	-2.19669100	1.57623100	-3.58819500
C	-3.56897300	0.66415700	0.08339600
H	-3.40777300	-0.41786800	0.08378500
H	-3.43388300	1.02949500	1.10645500
C	-0.56588100	2.52561200	0.76810600
H	-1.07783600	3.49682700	0.71175100
H	-1.02038000	1.97617100	1.60232200
H	0.47055800	2.73962300	1.05503800
H	-4.60979500	0.84972600	-0.20008800

INT2a-ISO’

Ir	1.05875600	-0.15287100	-0.58638600
N	-0.57744000	-1.49316700	-0.09093800
C	-0.75135300	-1.79049400	1.23019100
C	-1.75597200	-2.68984200	1.66498300
C	-2.61042900	-3.29680000	0.70277300
C	-2.39971500	-2.99112400	-0.64049800
C	-1.36826000	-2.09334800	-0.97757600
C	0.12689300	-1.17206000	2.18910100
N	1.05840200	-0.30623400	1.71691400
C	1.88105400	0.26881200	2.58472100
C	1.85149700	0.03785700	3.97666000
C	0.89675100	-0.84152200	4.48647400
C	0.00327500	-1.46405600	3.56977800
B	1.13717500	-0.42002400	-2.62045800
O	2.22169200	-0.09869000	-3.42702200
C	1.99467800	-0.52305400	-4.78698700
C	0.79048500	-1.53451000	-4.63689000
O	0.18005600	-1.10686200	-3.39218400
B	2.24601300	-1.91824200	-0.53983400
O	3.35455100	-2.23598800	-1.31886000
C	4.02673400	-3.38223300	-0.73779300
C	2.87158500	-4.06350100	0.08594500
O	2.00558300	-2.93827000	0.37814600
B	2.14315000	1.65179400	-0.72396400
O	2.26821800	2.48862300	-1.81350500
C	3.27493700	3.48917600	-1.52285000
C	3.27263400	3.52058100	0.05301600
O	2.82563500	2.17659000	0.37689900
H	-1.15888000	-1.86189000	-2.01446600
H	2.59163800	0.96786600	2.15628200
C	0.78911400	-1.13672100	5.96270500
H	-0.19100400	-0.84089000	6.35421800

H	0.90511500	-2.20795000	6.16235100
H	1.54751900	-0.60902500	6.54051100
C	2.85276000	0.76530600	4.84383800
H	2.36119800	1.41247600	5.57876600
H	3.49354200	0.07056400	5.39816500
H	3.50272900	1.39570400	4.23217400
C	-3.22619000	-3.57519300	-1.76284900
H	-4.28518100	-3.31190200	-1.66545700
H	-2.88254700	-3.19734600	-2.72832000
H	-3.16188200	-4.66861000	-1.79065700
C	-3.69794800	-4.24448800	1.14505300
H	-4.28388600	-4.61227100	0.30319300
H	-3.27921700	-5.11464500	1.66368000
H	-4.38847500	-3.75606200	1.84164600
C	-1.86311200	-2.95517000	3.07171500
H	-2.62967200	-3.63785300	3.41973200
C	-1.02547600	-2.37660400	3.97756100
H	-1.13997300	-2.60893200	5.03025300
C	-0.52160400	1.61745900	-0.36793400
H	2.59796700	0.17526400	-0.87133500
C	2.04734100	-5.06787900	-0.73216900
H	2.61507700	-5.97768500	-0.95153400
H	1.15961300	-5.34814900	-0.15761700
H	1.71323900	-4.63000900	-1.67631000
C	3.30102300	-4.70199000	1.40676200
H	2.42470100	-5.11118800	1.91869500
H	4.00545400	-5.52335400	1.23529200
H	3.76923500	-3.97446200	2.07210500
C	4.61505600	-4.23119500	-1.86391400
H	5.36919500	-3.65117500	-2.40400600
H	5.10121000	-5.12854700	-1.46542300
H	3.85176800	-4.53997800	-2.58045300
C	5.15860200	-2.83212300	0.14364000
H	5.76358200	-3.63292200	0.58084000
H	5.80998800	-2.20461800	-0.47097200
H	4.76149900	-2.21366700	0.95355600
C	1.24287200	-2.98834100	-4.43322700
H	1.68230000	-3.40977700	-5.34284700
H	1.97601100	-3.05330100	-3.62585700
H	0.37477800	-3.59734000	-4.16162900
C	-0.26149400	-1.46996400	-5.74403600
H	-1.06626500	-2.18093400	-5.53102000
H	-0.70390400	-0.47569100	-5.82470200
H	0.17279900	-1.73794300	-6.71322300
C	3.29438400	-1.14161500	-5.31024200
H	3.16370600	-1.55241100	-6.31766200
H	4.06656300	-0.36816000	-5.35978900
H	3.65568500	-1.92967700	-4.64801400
C	1.65123100	0.71733900	-5.62264800
H	0.72455000	1.18524500	-5.28710700
H	2.45486100	1.45049900	-5.51032400
H	1.55460100	0.47228200	-6.68546700
C	2.25646000	4.50556800	0.64718800
H	2.17033900	4.32350900	1.72249900
H	2.57162500	5.54334700	0.50079200
H	1.26506800	4.38065900	0.20527300
C	4.64140600	3.73921800	0.69781100
H	4.54394600	3.72278500	1.78792700
H	5.35165700	2.96226900	0.40968500
H	5.05479800	4.71292500	0.41441300
C	4.59309300	2.97516600	-2.12048800
H	4.90280000	2.03738700	-1.65119100
H	4.44023000	2.77820600	-3.18456700
H	5.40164600	3.70530300	-2.01329200
C	2.87451300	4.80044400	-2.19698400
H	3.58587400	5.59720100	-1.95362600
H	2.87365700	4.66833700	-3.28264200
H	1.87545300	5.12150700	-1.89806400
Si	-1.50193900	2.03732900	-1.90210100

Cl	-2.91705600	3.58965000	-1.35950700
C	-0.52348200	2.85575700	-3.28754100
H	0.42429000	2.34492300	-3.46061200
H	-1.11114100	2.87256900	-4.21194800
H	-0.29095300	3.89082800	-3.02107300
C	-2.63131200	0.69033200	-2.59030200
H	-2.03818800	-0.05865000	-3.12242200
H	-3.20199800	0.19567700	-1.79815400
C	-1.42991700	1.44850400	0.86642000
H	-2.07570600	0.56566700	0.79948100
H	-0.85370300	1.36068000	1.79265900
H	-2.09693000	2.31081900	0.99108300
H	0.05996900	2.53048300	-0.18928100
H	-3.34642300	1.13213800	-3.29161100

TS2a-ISO’

Ir	1.06315500	-0.20602900	-0.61409900
N	-0.60814200	-1.52321300	-0.10754500
C	-0.79323800	-1.79932100	1.21693800
C	-1.80991700	-2.67935100	1.66204900
C	-2.65659500	-3.30389600	0.70337400
C	-2.41753200	-3.04327200	-0.64452800
C	-1.38070400	-2.15353600	-0.98923500
C	0.10425900	-1.19537200	2.16816900
N	1.06623400	-0.37253500	1.68363700
C	1.92644100	0.16445800	2.53840500
C	1.90038000	-0.05709100	3.93196200
C	0.90751000	-0.88403600	4.45648400
C	-0.02011600	-1.47375400	3.55138400
B	1.16088100	-0.47077600	-2.64739300
O	2.22788900	-0.12368100	-3.46405700
C	2.00699900	-0.56528100	-4.81933800
C	0.81333300	-1.59019000	-4.66273400
O	0.20774600	-1.17487600	-3.41230400
B	2.24558900	-1.93474000	-0.57629900
O	3.37334700	-2.22298200	-1.33937400
C	4.06626500	-3.34986400	-0.74916600
C	2.91776800	-4.06615900	0.05394100
O	2.01630000	-2.96787200	0.33459100
B	1.80140200	1.78208800	-0.66422300
O	2.09202800	2.56394000	-1.78634100
C	3.17741800	3.45633200	-1.46219200
C	3.04897700	3.58945200	0.10411200
O	2.45078700	2.32116200	0.46707600
H	-1.15193700	-1.95261700	-2.02887200
H	2.65602000	0.83679300	2.10033900
C	0.79784500	-1.16223300	5.93582600
H	-0.17065800	-0.83256200	6.32903400
H	0.88145500	-2.23487100	6.14445400
H	1.57526800	-0.65336700	6.50515400
C	2.94441100	0.62639800	4.78433600
H	2.49196700	1.29762100	5.52272700
H	3.55951200	-0.09530700	5.33330200
H	3.61431400	1.22490300	4.16235200
C	-3.22195600	-3.66350400	-1.76349300
H	-4.27984500	-3.38401200	-1.70567300
H	-2.84783300	-3.33075200	-2.73430900
H	-3.17099300	-4.75766800	-1.74445700
C	-3.75922300	-4.22942400	1.15522000
H	-4.34675600	-4.60231900	0.31667300
H	-3.35476500	-5.09700300	1.68948300
H	-4.44552600	-3.72038900	1.84099600
C	-1.92943800	-2.91675000	3.07294200
H	-2.71242800	-3.57599100	3.42968100
C	-1.07817700	-2.34635800	3.97169000
H	-1.19938700	-2.56086200	5.02745200
C	-0.30430800	1.83048000	-0.42161200
H	2.53103400	0.32841900	-0.90191000
C	2.13609300	-5.09207100	-0.77938300

H	2.73228800	-5.98522300	-0.99194700
H	1.24782500	-5.39878800	-0.21929600
H	1.80454400	-4.66072200	-1.72733300
C	3.34471400	-4.69684800	1.37966800
H	2.47284000	-5.13380800	1.87616800
H	4.07608700	-5.49629300	1.21753000
H	3.78014400	-3.95807700	2.05483000
C	4.69890900	-4.17938400	-1.86617100
H	5.44622900	-3.57684000	-2.39076800
H	5.20244700	-5.06417600	-1.46104100
H	3.95791300	-4.50713300	-2.59766000
C	5.16738200	-2.77121500	0.15330700
H	5.78761800	-3.55587000	0.59847000
H	5.81040600	-2.12275500	-0.44813900
H	4.73929800	-2.16706400	0.95825300
C	1.28142100	-3.04055000	-4.46839200
H	1.72192200	-3.45340200	-5.38143500
H	2.01778300	-3.10225200	-3.66377700
H	0.42070700	-3.65971800	-4.19613300
C	-0.24817700	-1.53337800	-5.76153200
H	-1.04489900	-2.25153700	-5.54216700
H	-0.70006000	-0.54289300	-5.83733800
H	0.17951000	-1.79642400	-6.73499100
C	3.31512200	-1.17410800	-5.33396500
H	3.19220300	-1.59502800	-6.33819400
H	4.07863700	-0.39241900	-5.38748100
H	3.68345600	-1.95201200	-4.66378300
C	1.65396900	0.66273300	-5.66898300
H	0.72128500	1.12486400	-5.34222900
H	2.44984000	1.40499800	-5.56201800
H	1.56259600	0.40574900	-6.72949000
C	2.09731200	4.71021000	0.55011800
H	1.89863700	4.60155900	1.62038200
H	2.53389400	5.69963400	0.38122700
H	1.13959100	4.66829700	0.02550500
C	4.37649500	3.72404400	0.85211600
H	4.18923800	3.78828800	1.92892100
H	5.02753600	2.86685500	0.67098600
H	4.90674400	4.63400800	0.55113200
C	4.47993700	2.77239200	-1.90841300
H	4.65269900	1.84378700	-1.35764300
H	4.39098200	2.51370400	-2.96645300
H	5.35046700	3.42375600	-1.77831500
C	2.98670800	4.75827400	-2.24080700
H	3.75758400	5.49106700	-1.97819300
H	3.06498300	4.55624800	-3.31299900
H	2.00623600	5.20055000	-2.05429100
Si	-1.47390600	2.05390100	-1.87638000
Cl	-2.87421500	3.58477800	-1.27430400
C	-0.64053700	2.80530300	-3.38642500
H	0.29598300	2.29375600	-3.61308600
H	-1.30612900	2.76096900	-4.25524100
H	-0.39844800	3.85545900	-3.19987800
C	-2.60816000	0.63551000	-2.37543300
H	-2.04762000	-0.10591900	-2.95111700
H	-3.06492200	0.14665700	-1.51027900
C	-0.99489200	1.77426000	0.95364500
H	-1.56015200	0.84918900	1.10229200
H	-0.26432900	1.85139800	1.76364800
H	-1.70270200	2.60306500	1.07355600
H	0.16445500	2.82221800	-0.45368200
H	-3.41396500	1.02696000	-3.00476900

TS2a’

Ir	1.26800300	0.09246600	0.05412600
N	-0.56817600	-1.22228300	-0.27892300
C	-0.95702200	-1.99315100	0.76677000
C	-2.00343900	-2.94197400	0.64851700
C	-2.63985800	-3.11189400	-0.61360600

C	-2.19632900	-2.33380400	-1.68501800
C	-1.15874200	-1.40858900	-1.45400800
C	-0.25439800	-1.82611800	2.01391500
N	0.78585300	-0.95110900	2.04640500
C	1.44156200	-0.81004400	3.19229400
C	1.12446200	-1.49778500	4.38310500
C	0.06321700	-2.40028000	4.37133400
C	-0.64323200	-2.58006100	3.14835400
B	3.28355600	0.32122000	0.43531100
O	4.30839100	0.67436800	-0.43794400
C	5.58706900	0.46368500	0.20163300
C	5.20914600	0.46563800	1.73216500
O	3.82225000	0.04784800	1.70120800
B	2.18899800	-1.66960800	-0.66517600
O	2.99111800	-1.84176100	-1.78392700
C	2.98920500	-3.23742600	-2.16565200
C	2.55922800	-3.96245500	-0.83073500
O	1.85837800	-2.91328000	-0.12172700
B	1.74462500	2.10547100	-0.37636300
O	1.41698100	2.68842600	-1.61170200
C	2.23965400	3.85825500	-1.81936300
C	2.64777000	4.25041300	-0.34927600
O	2.58198900	2.97364100	0.33998200
H	-0.79217100	-0.79074600	-2.26682500
H	2.28583200	-0.13341700	3.16141200
C	-0.33664400	-3.18114200	5.59923700
H	-0.22185500	-4.25931100	5.43768800
H	0.26588300	-2.91179500	6.46625900
H	-1.38661900	-3.00367500	5.85730000
C	1.96177600	-1.22052800	5.60991300
H	1.36070100	-0.80834800	6.42819900
H	2.44731300	-2.12794000	5.98545400
H	2.74718200	-0.49641300	5.38152300
C	-2.77932800	-2.42855800	-3.07582900
H	-3.84463900	-2.17330800	-3.08783800
H	-2.26951200	-1.74104000	-3.75518300
H	-2.68000300	-3.43717800	-3.49123100
C	-3.75700900	-4.11484400	-0.76575600
H	-4.14946000	-4.13889700	-1.78198500
H	-3.41664700	-5.12630100	-0.51654400
H	-4.59048000	-3.88091600	-0.09380700
C	-2.37094800	-3.68852900	1.81735200
H	-3.17765200	-4.40979200	1.75283400
C	-1.72905400	-3.50861400	3.00630300
H	-2.03911900	-4.09024700	3.86704500
C	-0.06646400	1.91845000	0.81509600
H	1.44644500	0.38514600	-1.46791400
H	0.18745800	2.89524700	0.38932100
C	1.41426500	4.90927000	-2.56272500
H	1.98450300	5.83509300	-2.69688300
H	1.15120500	4.53078000	-3.55509000
H	0.48766600	5.14492400	-2.03583900
C	1.66234600	5.21191700	0.33497300
H	1.90360400	5.26406400	1.40051300
H	1.73805300	6.22091900	-0.08263900
H	0.62273200	4.88893900	0.24359100
C	4.06178600	4.81471500	-0.21026100
H	4.15894700	5.75475400	-0.76418400
H	4.27622700	5.02346800	0.84247200
H	4.81152400	4.11654200	-0.58207200
C	3.42921400	3.41494500	-2.68502600
H	3.04178800	2.99068000	-3.61598700
H	4.08735100	4.25258900	-2.93902600
H	4.00845200	2.63862400	-2.17936700
C	6.12484900	-0.88629600	-0.29298100
H	6.17186900	-0.86583800	-1.38464100
H	7.12573600	-1.09492600	0.09840000
H	5.45676400	-1.70003500	-0.00585900
C	6.55012200	1.57235900	-0.22384900

H	7.52457600	1.44968800	0.26190600
H	6.70526200	1.52636100	-1.30593700
H	6.16452100	2.56147400	0.02346100
C	5.24520700	1.86059200	2.37158700
H	4.82831500	1.79839200	3.38190200
H	6.26670400	2.24625300	2.45131700
H	4.63463800	2.56114900	1.79912000
C	5.99363600	-0.52411100	2.59460700
H	7.06137500	-0.27921800	2.59956200
H	5.63608000	-0.47623400	3.62809200
H	5.87372400	-1.55098900	2.24442600
C	1.96082700	-3.38300000	-3.29918100
H	2.23878200	-2.70805700	-4.11336900
H	1.92618800	-4.40382100	-3.69312400
H	0.95801600	-3.10580900	-2.96178700
C	1.60235700	-5.14295800	-1.01319300
H	2.06598800	-5.94068800	-1.60374100
H	1.34120900	-5.55707500	-0.03449200
H	0.67712700	-4.83834800	-1.50567200
C	3.73868700	-4.39986800	0.04915400
H	3.35309800	-4.72112100	1.02104000
H	4.29032000	-5.23437600	-0.39529300
H	4.43514600	-3.57676500	0.22293000
C	4.37710200	-3.60820100	-2.68898100
H	4.59538500	-3.02615000	-3.58911200
H	5.15651200	-3.39460100	-1.95633200
H	4.42607500	-4.67051000	-2.95266200
Si	-1.85007900	1.95493900	0.24944100
C	-2.05494600	1.99273600	-1.62509400
H	-1.15396000	2.40837800	-2.08552200
H	-2.21302400	0.98934800	-2.03108000
H	-2.91490000	2.60768700	-1.90609300
C	-3.08170100	0.77892000	1.07115900
H	-3.03578000	-0.22882200	0.65040500
H	-2.90366900	0.70941800	2.14899200
Cl	-2.58176200	3.88493100	0.88004200
C	0.11435500	2.10065400	2.33411200
H	-0.35714300	3.02834900	2.68358200
H	-0.32581800	1.28133200	2.90910100
H	1.17664100	2.15763300	2.59384200
H	-4.09808100	1.15877100	0.92688100

1b

C	0.04573000	0.87990700	3.52253200
H	-0.34729000	1.89608300	3.43821400
H	-0.30861000	0.29874300	2.66756400
H	-0.35766400	0.42876200	4.43694900
C	2.08537900	1.74493200	4.74561600
H	1.74460600	1.31781300	5.69636500
H	3.17756900	1.77971900	4.75662600
H	1.70645000	2.76736700	4.67296400
Cl	2.18929900	1.66020600	2.01366300
C	2.14121400	-0.53389700	3.64635100
H	1.80052100	-1.02584900	4.56525400
H	1.80313800	-1.12740200	2.79332200
H	3.23392700	-0.52040400	3.64869500
C	1.57215800	0.88326900	3.59276200

2b

B	1.12777600	2.77930900	-0.87077700
O	1.77114400	3.05331000	-2.05164900
C	3.18107500	3.26468900	-1.75452400
C	3.14878300	3.64270000	-0.22195800
O	1.92425400	2.99001000	0.23018200
C	-0.36793900	2.27022700	-0.85124100
C	2.96391100	5.14400100	0.03303500
H	2.74831300	5.30074400	1.09353400
H	3.86509100	5.70969500	-0.22102600
H	2.12779500	5.54625300	-0.54566400

C	4.31350300	3.10743400	0.60786600
H	4.17978800	3.39280200	1.65525100
H	4.37728500	2.01903200	0.56106800
H	5.26326700	3.52833200	0.26191800
C	3.89307600	1.93617700	-2.04168000
H	3.52492700	1.13731400	-1.39174900
H	3.69412100	1.64588100	-3.07679800
H	4.97591800	2.01953800	-1.90967300
C	3.71099200	4.35529100	-2.68344400
H	4.75764500	4.58566900	-2.45849800
H	3.65648500	4.00998400	-3.71965400
H	3.12657100	5.27348300	-2.60454200
H	-0.95775600	2.96093000	-1.46734900
Cl	-2.79306600	1.50807200	0.18625100
C	-0.37510200	1.02348200	1.36922000
H	0.64898500	1.33322700	1.60018500
H	-0.91786600	0.87665800	2.30649700
H	-0.34118100	0.06838600	0.83785200
C	-1.17216600	3.42454300	1.27115300
H	-0.17122100	3.80128500	1.50451200
H	-1.69717800	4.17128200	0.66907600
H	-0.40101100	1.31508800	-1.39155300
H	-1.72152600	3.28811500	2.20607000
C	-1.04156400	2.10227400	0.51583300

TS1b

Ir	1.00811900	-0.01083900	-0.05863200
N	-0.17192500	-1.81518100	0.75171900
C	-0.48915400	-1.73889200	2.06941900
C	-1.22309300	-2.75715000	2.72793300
C	-1.64333000	-3.89295700	1.97857300
C	-1.31096600	-3.94581600	0.62506300
C	-0.57318100	-2.87875300	0.06833900
C	-0.04907000	-0.57287900	2.79487000
N	0.65075500	0.37774500	2.11326700
C	1.06927900	1.45245400	2.77700500
C	0.82775300	1.67515800	4.14855300
C	0.10960800	0.72042200	4.86749800
C	-0.34254300	-0.43883300	4.17474900
B	2.86059800	-0.83559100	0.54101300
O	3.97160400	-0.11983100	0.97121300
C	5.07482700	-1.03580100	1.18444200
C	4.33054700	-2.40580800	1.40294500
O	3.09803400	-2.20701300	0.66768900
B	1.73052800	-0.92240800	-1.77266000
O	2.98732000	-0.81430000	-2.35607400
C	2.97960100	-1.48650100	-3.63696200
C	1.81338900	-2.52400900	-3.45688500
O	0.95674200	-1.84231200	-2.50407300
B	2.01343300	1.68667300	-0.57714700
O	2.31360700	2.12253700	-1.86840600
C	3.19027600	3.26805900	-1.78964100
C	2.83658000	3.85602500	-0.37537800
O	2.41936400	2.66965900	0.34116200
H	-0.29172800	-2.88631000	-0.98043600
H	1.62992700	2.16998900	2.18573500
C	-0.19292000	0.88778200	6.33641000
H	-1.27373100	0.90525400	6.51772600
H	0.21842800	0.05843400	6.92296700
H	0.22335900	1.81260600	6.73474900
C	1.36309400	2.94636100	4.76617800
H	0.56016400	3.57016800	5.17442600
H	2.06323300	2.73845800	5.58288300
H	1.89533100	3.53896700	4.01872000
C	-1.70182900	-5.09221000	-0.27838100
H	-2.78905300	-5.21635000	-0.33306600
H	-1.33909700	-4.91922500	-1.29441300
H	-1.28120900	-6.04388800	0.06512300
C	-2.42711400	-4.99287300	2.65267400

H	-2.65931600	-5.80701900	1.96664600
H	-1.86927500	-5.41756300	3.49485500
H	-3.37558500	-4.61556900	3.05215800
C	-1.50806600	-2.58814000	4.12390400
H	-2.07078900	-3.35458200	4.64474900
C	-1.08871300	-1.48718600	4.81074800
H	-1.32557100	-1.39893800	5.86490600
C	-1.08865100	1.01670100	-0.40137500
H	0.15220600	0.29988600	-1.37173500
H	-0.89576400	1.98823800	0.05556500
C	2.89755000	4.19145600	-2.97177800
H	3.49887100	5.10586300	-2.91796200
H	3.14725800	3.67915100	-3.90576500
H	1.84270600	4.46967900	-3.01446600
C	1.64090800	4.81989500	-0.40240100
H	1.32182900	5.02399800	0.62417100
H	1.89551300	5.77347000	-0.87592000
H	0.79502600	4.38089300	-0.93777000
C	4.00404700	4.49565000	0.37636400
H	4.40547900	5.35212200	-0.17644300
H	3.66266800	4.85842900	1.35141200
H	4.80948800	3.77962600	0.54753600
C	4.63053400	2.73989900	-1.87568400
H	4.73841600	2.16439500	-2.79899900
H	5.36564500	3.55157300	-1.88449500
H	4.84718100	2.07031700	-1.03945700
C	5.93364000	-1.00753200	-0.08786000
H	6.28738900	0.01412400	-0.25361800
H	6.80743000	-1.66191500	-0.00448700
H	5.34423400	-1.29956100	-0.95990300
C	5.89246800	-0.54089800	2.37761400
H	6.70957700	-1.23279300	2.61051400
H	6.33240800	0.43211800	2.13985100
H	5.27385900	-0.41911900	3.26890500
C	3.93929700	-2.65695800	2.86750300
H	3.26382100	-3.51631900	2.91123200
H	4.81092700	-2.87193400	3.49402800
H	3.41476400	-1.79303900	3.28525500
C	5.04406300	-3.63494700	0.84080400
H	6.00525500	-3.79766400	1.34103700
H	4.42631600	-4.52381000	1.00197800
H	5.22372900	-3.53901300	-0.23146600
C	2.68632000	-0.42194700	-4.70466400
H	3.43194200	0.37280500	-4.62143700
H	2.73114900	-0.83711800	-5.71677300
H	1.70309200	0.03033200	-4.55289200
C	1.00118500	-2.82132700	-4.71646200
H	1.62824600	-3.28209300	-5.48761300
H	0.19412800	-3.52135700	-4.47875900
H	0.54980800	-1.91730200	-5.12925700
C	2.26970800	-3.83474200	-2.79883900
H	1.38949400	-4.43579900	-2.54974500
H	2.90220800	-4.42677700	-3.46788400
H	2.81599100	-3.63727000	-1.87270500
C	4.35972100	-2.09664000	-3.88065600
H	5.10537700	-1.29908200	-3.94894700
H	4.65480200	-2.76523300	-3.06966800
H	4.38071600	-2.65902400	-4.82069000
H	-1.67891900	0.40287600	0.28045600
C	-1.20702700	2.18566500	-2.66531600
H	-1.07544800	3.16201300	-2.19187500
H	-0.21669200	1.82037200	-2.95719700
H	-1.81838700	2.31625700	-3.56207100
C	-2.23570100	-0.11668000	-2.37477200
H	-2.87781600	0.05926600	-3.24182600
H	-1.32783200	-0.63313400	-2.70563200
Cl	-3.53156300	2.00879200	-1.21382600
H	-2.76586100	-0.77232700	-1.67849200
C	-1.85794200	1.19971200	-1.69794700

INT2b

Ir	0.90360400	0.01695300	-0.08043900
N	-0.29194400	-1.82637600	0.67248200
C	-0.51720100	-1.80404300	2.01022200
C	-1.28133500	-2.80193900	2.66608200
C	-1.83409500	-3.86081800	1.89185600
C	-1.58572400	-3.86665200	0.51976800
C	-0.80654500	-2.82628500	-0.03074700
C	0.05730300	-0.71830000	2.76103700
N	0.78232900	0.21817700	2.08228300
C	1.32235900	1.22185300	2.77270100
C	1.18957900	1.37982800	4.16635000
C	0.45186500	0.43901500	4.88370200
C	-0.13186200	-0.64084500	4.16345900
B	2.80632000	-0.83428600	0.44683500
O	3.89380900	-0.05194900	0.80893600
C	4.99875300	-0.91070300	1.18816700
C	4.26482700	-2.25753600	1.54804500
O	3.05113600	-2.17091900	0.75596500
B	1.81376600	-1.03282700	-1.65101000
O	2.99847700	-0.77136700	-2.32949200
C	3.01302700	-1.54866800	-3.55198500
C	2.06128000	-2.75023500	-3.19238800
O	1.17304100	-2.14247900	-2.22187300
B	1.86281100	1.75385300	-0.56100300
O	2.15190100	2.18999300	-1.85131200
C	3.04673900	3.32268400	-1.77550700
C	2.72923300	3.90254300	-0.34727900
O	2.28266700	2.71952800	0.36143500
H	-0.57294000	-2.81502100	-1.08911700
H	1.87994200	1.94282000	2.18561500
C	0.26101000	0.54371900	6.37673700
H	-0.80045600	0.63676100	6.63344000
H	0.63928700	-0.34839200	6.88853700
H	0.77777000	1.40784700	6.79323500
C	1.85494000	2.57527700	4.80850400
H	1.12633400	3.22945100	5.29988800
H	2.58847900	2.27614900	5.56546500
H	2.37914800	3.16992000	4.05712000
C	-2.11225900	-4.93388800	-0.41131300
H	-3.20718400	-4.97322900	-0.40803800
H	-1.79365500	-4.73748400	-1.43779800
H	-1.74861400	-5.93036700	-0.13674900
C	-2.65950500	-4.93337400	2.55992200
H	-3.00963100	-5.68012600	1.84781300
H	-2.08194800	-5.45584600	3.33107400
H	-3.54064700	-4.50557500	3.05167200
C	-1.46058900	-2.69174100	4.08491700
H	-2.04522700	-3.44306200	4.60356800
C	-0.91337800	-1.66525300	4.79578800
H	-1.07285000	-1.61795600	5.86683700
C	-1.03043800	1.13266800	0.05496500
H	0.63773200	0.09022500	-1.65315100
H	-0.81598200	2.11298300	0.49259400
C	2.74332200	4.26332700	-2.94113000
H	3.36255400	5.16590900	-2.89211800
H	2.96232300	3.75579800	-3.88531600
H	1.69311100	4.56067600	-2.95656500
C	1.56691500	4.90518600	-0.33933800
H	1.27226400	5.10213400	0.69567800
H	1.84745100	5.85666200	-0.80235000
H	0.69747200	4.50441200	-0.86565800
C	3.92803600	4.49048800	0.39751900
H	4.35047600	5.34114700	-0.14835000
H	3.61153000	4.85076000	1.38183300
H	4.71191600	3.74593100	0.54651600
C	4.47598800	2.77249700	-1.89791800
H	4.55679200	2.21322200	-2.83379100

H	5.22393700	3.57234600	-1.90518300
H	4.69585600	2.08198700	-1.07986100
C	5.91959300	-1.02293300	-0.03427000
H	6.26588000	-0.02281600	-0.30974600
H	6.79685800	-1.64497400	0.17061500
H	5.38063200	-1.43174100	-0.89137400
C	5.74969200	-0.25085900	2.34501400
H	6.56756000	-0.88845100	2.69856200
H	6.18263900	0.69478000	2.00595500
H	5.08811400	-0.03431100	3.18585600
C	3.82869800	-2.34120400	3.01860400
H	3.16511300	-3.20186900	3.14219100
H	4.68373000	-2.46728200	3.69040100
H	3.27912500	-1.44531400	3.31995600
C	5.01404200	-3.53247400	1.16190300
H	5.95663600	-3.61454900	1.71407900
H	4.40244000	-4.40618700	1.40623200
H	5.23575100	-3.56448600	0.09372200
C	2.45642200	-0.64573600	-4.66262700
H	3.05670500	0.26639400	-4.70923800
H	2.49008100	-1.13566000	-5.64091000
H	1.42435300	-0.35221400	-4.45347300
C	1.22069700	-3.27943900	-4.35430900
H	1.85966300	-3.68023000	-5.14872900
H	0.57563200	-4.09151600	-4.00438300
H	0.58280600	-2.50233700	-4.77921300
C	2.77886100	-3.91139400	-2.49034300
H	2.03062700	-4.62041500	-2.12370700
H	3.45017100	-4.44493800	-3.17061000
H	3.34628700	-3.55450800	-1.62827200
C	4.45535600	-1.93641800	-3.87241700
H	5.04741000	-1.03307200	-4.04554100
H	4.91819100	-2.49203600	-3.05479600
H	4.50249300	-2.55002400	-4.77870900
H	-1.61956500	0.57399300	0.79017600
C	-1.32153800	2.31229300	-2.19887400
H	-1.15315500	3.28584600	-1.73150800
H	-0.36254000	1.95314000	-2.58347200
H	-2.01409100	2.44945600	-3.03402200
C	-2.33565100	0.01895400	-1.84261700
H	-3.06713300	0.21492300	-2.63114900
H	-1.47168800	-0.48794700	-2.28589400
Cl	-3.54580700	2.14378600	-0.59310200
H	-2.78809300	-0.65212500	-1.10785600
C	-1.87796400	1.31867200	-1.18316000

INT2b-ISO

Ir	0.78363400	-0.43693500	-0.20922800
N	-0.45267300	-2.33383500	0.23651400
C	-0.63201700	-2.61922000	1.55248700
C	-1.44811400	-3.69727200	1.97712700
C	-2.09679400	-4.49796100	0.99282500
C	-1.88563500	-4.19022900	-0.35158800
C	-1.05112800	-3.09668000	-0.66855000
C	0.05361300	-1.79847200	2.52546700
N	0.82015300	-0.77725300	2.06765200
C	1.49190600	-0.04718200	2.94491200
C	1.44498900	-0.24405100	4.34206900
C	0.64221400	-1.26996200	4.83995600
C	-0.07113000	-2.07866900	3.90861300
B	0.72759700	-0.38917000	-2.24890900
O	1.72242500	0.12174900	-3.08354600
C	1.35169200	-0.03763500	-4.46947600
C	0.21863700	-1.13726000	-4.40346900
O	-0.25046700	-1.02373400	-3.03673000
B	2.35738100	-2.07697100	-0.34495200
O	2.51236400	-2.86850100	-1.46648800
C	3.42784200	-3.94850700	-1.13152000
C	3.30612900	-4.01903700	0.44060900

O	2.90143200	-2.66553100	0.78009300
B	1.72304800	1.38852700	-0.31687200
O	1.23118900	2.58140100	-0.82929100
C	2.28893900	3.56748000	-0.84996500
C	3.27686500	3.02523200	0.25119200
O	2.98617300	1.60568500	0.25633100
H	-0.87373800	-2.81286300	-1.70184100
H	2.11032400	0.73788900	2.52048500
C	0.51981300	-1.53978200	6.31989800
H	-0.52137700	-1.45695700	6.65144100
H	0.85747600	-2.55276100	6.56726500
H	1.11088200	-0.84142000	6.91166400
C	2.25854500	0.67401700	5.22395700
H	1.62522600	1.23973700	5.91620400
H	2.98812100	0.12071500	5.82558400
H	2.81066800	1.39666900	4.61843800
C	-2.51537200	-4.96251500	-1.48742900
H	-3.60929800	-4.91134800	-1.45519500
H	-2.19637700	-4.55519200	-2.44970100
H	-2.23688500	-6.02191500	-1.46638400
C	-2.98263000	-5.64324200	1.41904000
H	-3.41726200	-6.16258500	0.56539700
H	-2.42316500	-6.37994200	2.00685900
H	-3.80777700	-5.28940200	2.04726400
C	-1.57245300	-3.93644300	3.38713100
H	-2.20023200	-4.75089500	3.73036500
C	-0.91543400	-3.17008900	4.30412800
H	-1.03338600	-3.39050000	5.35903500
C	-1.05848200	0.60071600	0.27618100
H	2.45332400	-0.76001000	-0.41675400
H	-1.51193600	-0.00611900	1.06837000
C	2.19781800	-4.96255400	0.92657400
H	2.46714700	-6.01190100	0.77141200
H	2.04179800	-4.80384600	1.99720500
H	1.25301900	-4.76693700	0.41417500
C	4.60856900	-4.32674100	1.17939800
H	4.42588100	-4.32719600	2.25796700
H	4.99280700	-5.31396100	0.90127200
H	5.37670200	-3.58069600	0.96947300
C	2.98854500	-5.20884400	-1.87440400
H	3.08944700	-5.05440400	-2.95252900
H	3.61431200	-6.06370300	-1.59648600
H	1.94627700	-5.45651200	-1.66624000
C	4.81704500	-3.50901400	-1.61458100
H	5.56737400	-4.28983900	-1.45674900
H	4.76699600	-3.29013400	-2.68456600
H	5.14380700	-2.60072100	-1.10133900
C	0.74529000	-2.56982400	-4.57860300
H	1.10551400	-2.75055600	-5.59647200
H	1.54841800	-2.77986600	-3.86890400
H	-0.07112800	-3.27299600	-4.38289500
C	-0.96541700	-0.90447800	-5.34336300
H	-1.71163600	-1.69149400	-5.19620900
H	-1.44969600	0.05499400	-5.15446200
H	-0.64755200	-0.93476600	-6.39137600
C	2.60072700	-0.44793400	-5.25405500
H	2.36421800	-0.63655300	-6.30710600
H	3.33664000	0.36064500	-5.21342000
H	3.06251300	-1.34222600	-4.83189500
C	0.85913200	1.32583100	-4.97771000
H	-0.01421500	1.67223700	-4.42019900
H	1.65527500	2.06166000	-4.83739000
H	0.60431800	1.29459800	-6.04202100
C	2.96625600	3.55655500	1.65880200
H	3.57301100	3.00766400	2.38642800
H	3.20261900	4.62072400	1.75715300
H	1.91169100	3.41232200	1.91056600
C	4.76300300	3.21316800	-0.05505000
H	5.36215600	2.77120400	0.74708700

H	5.04207000	2.72463700	-0.99014300
H	5.02153200	4.27566000	-0.12391000
C	2.88880600	3.55342600	-2.26371800
H	3.27198900	2.56169700	-2.51586300
H	2.09960200	3.79610000	-2.98066900
H	3.69133500	4.28988700	-2.37396400
C	1.67936200	4.93954100	-0.55970400
H	2.45289300	5.71453700	-0.51835700
H	0.98106400	5.20332200	-1.35924500
H	1.12587800	4.94661800	0.38108500
H	-0.72879400	1.52885000	0.75175700
Cl	-3.50055200	1.88319400	0.40156000
C	-1.80582200	1.98029100	-1.75907700
H	-1.09790200	1.51132900	-2.44620500
H	-2.68886000	2.29588700	-2.32225400
H	-1.31762500	2.85284200	-1.32504300
C	-2.93775200	-0.22394200	-1.26531300
H	-2.27577100	-0.75567400	-1.95341700
H	-3.26133000	-0.90601600	-0.47399700
H	-3.82254200	0.10810400	-1.81601000
C	-2.17838000	0.96483700	-0.68773100

TSb-ISO

Ir	0.48021100	-0.43590500	-0.25351800
N	-0.97097800	-2.02217700	0.26066800
C	-0.97731200	-2.48329400	1.54189800
C	-1.84316200	-3.51820200	1.97322300
C	-2.77647700	-4.06709500	1.05278700
C	-2.79801900	-3.53876800	-0.23746900
C	-1.87645800	-2.52951200	-0.57590100
C	-0.09016100	-1.84631700	2.46761000
N	0.63732000	-0.79684600	1.99943900
C	1.46540400	-0.20114500	2.85467200
C	1.62197700	-0.56872700	4.20556500
C	0.86769500	-1.62976400	4.70629800
C	-0.01859800	-2.28768400	3.81234400
B	0.41629100	-0.82574100	-2.29847800
O	0.99350700	-0.05329700	-3.30152900
C	0.52055900	-0.51040900	-4.59031500
C	0.10773700	-1.99408500	-4.27496600
O	-0.23985700	-1.91771900	-2.86479100
B	2.12568000	-1.76752400	-0.37929200
O	3.14262200	-1.79135800	-1.33687900
C	4.19125200	-2.68756900	-0.88909800
C	3.41528900	-3.63801500	0.09440300
O	2.33784500	-2.78568000	0.55243000
B	1.83732000	1.19495100	-0.17792100
O	1.70656500	2.43111400	-0.79951200
C	2.97176700	3.13084700	-0.71706900
C	3.61497400	2.48698400	0.56509300
O	3.00441800	1.16139500	0.57953200
H	-1.85382800	-2.14223800	-1.58356700
H	2.06664700	0.59744100	2.44278100
C	0.97023600	-2.08032800	6.14263000
H	0.00688800	-1.98671800	6.65708700
H	1.26855100	-3.13287900	6.20668300
H	1.70030600	-1.49618900	6.70231100
C	2.60334700	0.21146200	5.04939100
H	2.11397900	0.69613200	5.90151200
H	3.39571900	-0.43072100	5.44963800
H	3.08270400	0.99449100	4.45769200
C	-3.76078100	-4.00561900	-1.30466100
H	-4.80331800	-3.84606600	-1.00816200
H	-3.59569400	-3.45971000	-2.23631000
H	-3.64186800	-5.07300900	-1.52168000
C	-3.71272600	-5.16848300	1.48467900
H	-4.36719600	-5.48715600	0.67379400
H	-3.15744800	-6.04855900	1.82884400
H	-4.34951100	-4.84435600	2.31587400

C	-1.74117400	-3.95266000	3.33718300
H	-2.38008700	-4.75783100	3.68165300
C	-0.86724500	-3.37464400	4.20888400
H	-0.82249800	-3.73233700	5.23126000
C	-1.31950300	0.99793800	-0.37467600
H	1.01156100	0.56086000	-1.33103200
H	-1.01361800	1.75975100	-1.09385000
C	2.76891900	-4.84229000	-0.60637500
H	3.51296300	-5.58042300	-0.92294400
H	2.07928800	-5.32704400	0.09084000
H	2.19582100	-4.52405400	-1.48115900
C	4.21140100	-4.10992100	1.31118900
H	3.57499200	-4.73367800	1.94658800
H	5.07743100	-4.70952700	1.00956500
H	4.56248100	-3.26763700	1.91035500
C	4.80956200	-3.37398900	-2.10678500
H	5.28040500	-2.62427100	-2.74974200
H	5.58163900	-4.09027200	-1.80388600
H	4.06088500	-3.90264900	-2.69952800
C	5.24916500	-1.82370800	-0.18651900
H	6.09886900	-2.42050700	0.16089200
H	5.62119200	-1.07698900	-0.89400400
H	4.81657300	-1.29017100	0.66336200
C	1.27347300	-2.98488100	-4.39057100
H	1.57351800	-3.13787200	-5.43196100
H	2.13536500	-2.63503500	-3.81753500
H	0.95950500	-3.94977100	-3.98079600
C	-1.10583700	-2.51313800	-5.04424900
H	-1.33347100	-3.53552700	-4.72688700
H	-1.98981500	-1.89685200	-4.86889300
H	-0.90599300	-2.53345600	-6.12091900
C	1.65008900	-0.35101900	-5.60699300
H	1.35729300	-0.74662500	-6.58578500
H	1.88285900	0.71080000	-5.72894700
H	2.55860500	-0.85920900	-5.27963100
C	-0.66720100	0.38352000	-4.97531700
H	-1.48215600	0.29061300	-4.25215100
H	-0.33935400	1.42645500	-4.97413900
H	-1.05215300	0.14400300	-5.97166000
C	3.21142000	3.19961100	1.86236700
H	3.56109200	2.61600200	2.71958300
H	3.66372500	4.19341800	1.93042200
H	2.12625000	3.30772000	1.93678800
C	5.13170600	2.31176900	0.51731200
H	5.47651200	1.82348400	1.43381200
H	5.43896200	1.69463900	-0.32803300
H	5.63310300	3.28290600	0.44400700
C	3.74107300	2.81057600	-2.00724400
H	3.93590500	1.73833400	-2.09558400
H	3.12975800	3.10942200	-2.86264000
H	4.69263400	3.34927300	-2.05545900
C	2.69339200	4.63057300	-0.63071000
H	3.62396900	5.19603000	-0.51096200
H	2.21241300	4.96354900	-1.55458400
H	2.02631900	4.87307400	0.19833400
H	-2.09345400	0.38499700	-0.85056100
Cl	-3.37343100	2.85440500	0.17551700
C	-2.62569700	0.76086600	1.80888300
H	-1.89413100	0.12031400	2.31025500
H	-3.15702400	1.33742000	2.57039800
H	-3.34767700	0.12365500	1.29172700
C	-0.96880200	2.65459200	1.52894600
H	-0.18098800	2.08701100	2.03765900
H	-0.49724400	3.32481400	0.80743100
H	-1.50260700	3.24980500	2.27451800
C	-1.90214000	1.67448700	0.82599100

TS2b

Ir	1.66809300	-0.19908900	-0.24670500

N	-0.23412800	-1.40246200	0.06082800
C	-0.62760700	-1.51546900	1.35558500
C	-1.76325600	-2.27533800	1.72872200
C	-2.50310400	-2.95041800	0.71658900
C	-2.07050000	-2.83160800	-0.60292100
C	-0.93028200	-2.04519000	-0.86298700
C	0.14580500	-0.82596400	2.35865800
N	1.23613500	-0.12445400	1.94587800
C	1.92298700	0.56225400	2.85061300
C	1.61470800	0.58578300	4.22693800
C	0.52385500	-0.15472800	4.68039500
C	-0.24061500	-0.87755700	3.72050500
B	3.72152800	0.01298200	-0.01055800
O	4.70334800	0.46645600	-0.88646200
C	6.01786800	0.21390700	-0.33097600
C	5.71082300	0.02520200	1.20615900
O	4.31502000	-0.35163500	1.20774500
B	2.51071800	-2.13681200	-0.32758800
O	3.59567200	-2.64178700	-1.04000900
C	3.73884500	-4.06201200	-0.77276000
C	2.30526500	-4.44900200	-0.26134500
O	1.83546200	-3.19696600	0.28906600
B	2.10524100	1.85078100	-0.68350100
O	2.15413900	2.47352600	-1.93884500
C	2.95697400	3.67356700	-1.87383900
C	2.98784300	3.98502800	-0.32289800
O	2.72723000	2.69036500	0.27128300
H	-0.57381800	-1.94257400	-1.88154600
H	2.76655400	1.12189100	2.46802100
C	0.13657200	-0.19574000	6.13846400
H	-0.87020300	0.20958000	6.29259700
H	0.13077000	-1.22397400	6.51729300
H	0.82261800	0.38071300	6.75854800
C	2.49070800	1.41246200	5.13947200
H	1.91872800	2.18633000	5.66329300
H	2.98044300	0.79595500	5.90156300
H	3.27490500	1.91253800	4.56643700
C	-2.77310500	-3.48605100	-1.76886600
H	-3.80112000	-3.12288100	-1.87460100
H	-2.25215500	-3.26726700	-2.70408100
H	-2.81762900	-4.57540800	-1.66199900
C	-3.72228800	-3.75883000	1.08666400
H	-4.17524700	-4.23547600	0.21774600
H	-3.47245600	-4.54659200	1.80625200
H	-4.48602600	-3.12597800	1.55298200
C	-2.11648500	-2.32363000	3.11847300
H	-2.98277600	-2.90022500	3.42231100
C	-1.39532700	-1.65690500	4.06443100
H	-1.70225200	-1.71510000	5.10254800
C	0.11624300	1.60300000	-0.33476400
H	1.87289600	-0.31918700	-1.80474900
H	-0.41228400	1.12445800	0.48479100
C	1.34745700	-4.84189700	-1.39687900
H	1.60920400	-5.81171900	-1.83166800
H	0.33099000	-4.90824600	-0.99866100
H	1.35432900	-4.09246600	-2.19319600
C	2.25766100	-5.50932300	0.83936200
H	1.21840500	-5.67804000	1.13768300
H	2.66691600	-6.46339700	0.48931800
H	2.81242800	-5.19429000	1.72460400
C	4.18292000	-4.74722300	-2.06581700
H	5.17783600	-4.39019700	-2.34721900
H	4.23852400	-5.83380700	-1.93596500
H	3.50516400	-4.52714300	-2.89243700
C	4.81544400	-4.24516000	0.30604500
H	4.96288400	-5.30278800	0.54578600
H	5.76767100	-3.85147100	-0.05689600
H	4.55000000	-3.71062200	1.22149300
C	5.82354100	1.32072800	2.02496000

H	6.85590900	1.67823800	2.09251700
H	5.20094000	2.10851100	1.59660200
H	5.46955300	1.12273600	3.04198600
C	6.51868200	-1.07663600	1.89333600
H	6.19770900	-1.17008300	2.93553100
H	6.37789700	-2.04294000	1.40929400
H	7.58800300	-0.83754100	1.89094000
C	6.95281300	1.38389800	-0.64799600
H	7.94883900	1.19571000	-0.23177300
H	7.05860900	1.48870600	-1.73140200
H	6.59245400	2.33217500	-0.24885600
C	6.55999800	-1.04086000	-1.02906100
H	5.87481200	-1.87703700	-0.90312800
H	6.63223400	-0.83651300	-2.10139000
H	7.55578800	-1.31409900	-0.66468700
C	1.88217200	4.94676400	0.14582000
H	1.86439300	4.95241700	1.23991300
H	2.06526200	5.96998200	-0.19742200
H	0.89286000	4.64508900	-0.20344500
C	4.32827600	4.51025100	0.19233100
H	4.28181200	4.64194200	1.27798300
H	5.14333700	3.82457200	-0.03535700
H	4.56185400	5.48289100	-0.25403500
C	4.32590000	3.33566300	-2.47905900
H	4.78281800	2.49989800	-1.95288600
H	4.17660400	3.02842300	-3.51862800
H	5.00100000	4.19798600	-2.47295500
C	2.30567400	4.76463200	-2.73034900
H	2.92098600	5.67122100	-2.72900900
H	2.22879400	4.41470800	-3.76392500
H	1.30538900	5.03532600	-2.39035100
H	0.30985900	2.59854000	0.07144500
C	-1.19836700	3.22886000	-1.79772000
H	-0.29097600	3.73302300	-2.13938000
H	-1.96182000	3.32620300	-2.57412400
H	-1.56001100	3.72214300	-0.89141400
C	-0.47126700	1.06933400	-2.83776700
H	0.46359700	1.50825800	-3.19367400
H	-0.29621600	0.00539600	-2.68977400
H	-1.25348500	1.20586800	-3.59023800
Cl	-2.53244200	0.98763100	-1.02236300
C	-0.85603600	1.76184200	-1.53557400

TS2b-ISO

Ir	1.29941100	-0.30545700	-0.50333100
N	-0.40221800	-1.53249300	0.09804600
C	-0.60417400	-1.69245700	1.43794800
C	-1.64862300	-2.50318800	1.94571700
C	-2.50383000	-3.18140300	1.03145900
C	-2.25733900	-3.02715200	-0.33206200
C	-1.19639900	-2.19411300	-0.73875900
C	0.29613300	-1.02306800	2.34221900
N	1.29061300	-0.27814000	1.79492400
C	2.13935300	0.33756500	2.60663900
C	2.07898100	0.26259200	4.01555900
C	1.06585100	-0.49645400	4.60136500
C	0.14247900	-1.16061800	3.74342600
B	1.26657700	-0.60117200	-2.51835500
O	2.37271000	-0.75308600	-3.35527900
C	1.94147300	-0.74877700	-4.73418600
C	0.43046800	-1.18111300	-4.61836300
O	0.09125900	-0.74780900	-3.27635300
B	2.43037500	-2.05004900	-0.51873300
O	3.77364100	-2.11687800	-0.16391000
C	4.15326100	-3.50451500	-0.01078300
C	3.06673600	-4.24539400	-0.87562900
O	1.94654700	-3.32457100	-0.81452900
B	2.03169100	1.66055800	-0.74452700
O	2.24126500	2.34395500	-1.94533800

C	3.37183200	3.22987600	-1.77711200
C	3.35875700	3.49892800	-0.22414300
O	2.77597100	2.27710400	0.28694300
H	-0.98850000	-2.04262500	-1.79156600
H	2.89234800	0.94426300	2.11523900
C	0.92641800	-0.62624800	6.09859700
H	-0.03842700	-0.23406600	6.44036200
H	0.97632000	-1.67556400	6.41112200
H	1.70936200	-0.08586100	6.62977400
C	3.11549700	1.01647100	4.81590600
H	2.65732900	1.75870900	5.47864400
H	3.71471500	0.34500200	5.44091000
H	3.80093700	1.54659600	4.15060700
C	-3.08210900	-3.70327700	-1.40231800
H	-4.12938900	-3.38248500	-1.37134500
H	-2.69366200	-3.46330800	-2.39470500
H	-3.06983400	-4.79350300	-1.29724700
C	-3.63166600	-4.04018400	1.54824200
H	-4.22033900	-4.47051100	0.73868400
H	-3.25155200	-4.86721500	2.15924400
H	-4.31187700	-3.45858000	2.18040400
C	-1.78832600	-2.60894000	3.37058700
H	-2.58893300	-3.21723400	3.77561500
C	-0.93826000	-1.97147700	4.22581300
H	-1.07892900	-2.08273700	5.29503100
C	-0.02011900	1.72787700	-0.24215200
H	2.78484600	0.15552700	-0.80618500
C	3.46084800	-4.37660300	-2.35357300
H	4.28757900	-5.07993600	-2.49535700
H	2.59940300	-4.74756500	-2.91740000
H	3.74407000	-3.40577900	-2.76789400
C	2.61470000	-5.60117000	-0.33389400
H	1.83817600	-6.01533200	-0.98419300
H	3.44745400	-6.31283600	-0.30913300
H	2.20027200	-5.51615700	0.67255200
C	5.59513400	-3.67030500	-0.49107900
H	6.26433300	-3.09602500	0.15658800
H	5.90651800	-4.72014900	-0.45272100
H	5.72198100	-3.30408800	-1.51130300
C	4.06229400	-3.83135500	1.48792300
H	4.39792400	-4.85014400	1.70700300
H	4.69944400	-3.13393000	2.03924100
H	3.03808800	-3.71436500	1.85310300
C	0.22699600	-2.70300000	-4.66366500
H	0.44071600	-3.11529400	-5.65476300
H	0.86105100	-3.20472500	-3.92807300
H	-0.81687900	-2.93028200	-4.42395800
C	-0.51997000	-0.50031500	-5.60305800
H	-1.54646600	-0.82787800	-5.41101000
H	-0.49109000	0.58604000	-5.50498500
H	-0.27024200	-0.76453800	-6.63651200
C	2.82681700	-1.71068400	-5.52832300
H	2.50848400	-1.76536800	-6.57538100
H	3.86050900	-1.35305700	-5.50915900
H	2.81225900	-2.71734200	-5.10708800
C	2.13242900	0.68001200	-5.26550500
H	1.56247200	1.40321300	-4.67798600
H	3.18952600	0.94559200	-5.17476400
H	1.84713900	0.76737200	-6.31891600
C	2.45491300	4.67248100	0.18444500
H	2.32585200	4.65896500	1.27080000
H	2.89499700	5.63526300	-0.09481200
H	1.46426300	4.60518800	-0.27161000
C	4.73934600	3.67265700	0.41097700
H	4.63186400	3.83672700	1.48803600
H	5.36137000	2.78809600	0.26342900
H	5.25912700	4.54087200	-0.00854900
C	4.62325200	2.47145800	-2.24770800
H	4.80483300	1.58304700	-1.63717600

H	4.46472800	2.13866400	-3.27657900
H	5.51674300	3.10356800	-2.22077300
C	3.16036200	4.46624400	-2.65098600
H	3.97354800	5.18720400	-2.51195000
H	3.14842200	4.17213000	-3.70466200
H	2.21425000	4.96401500	-2.43062000
H	-0.44979200	1.31509700	0.67238300
Cl	-2.15677400	3.47735300	-0.28002300
C	-0.73652700	2.71914700	-2.51560100
H	-0.19836800	1.97232800	-3.10423200
H	-1.61748100	3.04706300	-3.07422200
H	-0.07488400	3.57163500	-2.36294300
C	-2.17666200	0.97600200	-1.42088500
H	-1.72195600	0.20044900	-2.04218200
H	-2.50441300	0.53969500	-0.47412300
H	0.43221800	2.66334300	0.08879800
H	-3.05367200	1.36787500	-1.94268300
C	-1.15149900	2.08038000	-1.19685600

1c

Si	1.63681000	0.94754100	3.47475200
C	-0.25680900	0.86687600	3.45512400
H	-0.69759100	1.86729500	3.38189800
H	-0.62329800	0.28183700	2.60424800
H	-0.64619900	0.40157600	4.36752000
C	2.20515100	1.98167300	4.95863200
H	1.88007700	1.53601800	5.90542300
H	3.29610500	2.07344300	4.99367900
H	1.79249000	2.99570900	4.91343900
C	2.32039600	-0.82284200	3.61153000
H	1.90248100	-1.27946900	4.51963400
H	1.91303300	-1.41033400	2.77681900
C	3.85405100	-0.94996200	3.63021200
H	4.30387300	-0.54098800	2.71880700
H	4.29318800	-0.41351200	4.47852800
H	4.17350200	-1.99545400	3.70796500
C	2.24885500	1.75955300	1.87468200
H	1.92831300	1.19531500	0.99176900
H	1.85812100	2.77830900	1.77413600
H	3.34212400	1.82382700	1.84659200

2c

B	-0.29677200	1.27387800	0.24835200
O	-0.27085500	0.30238400	-0.73087100
C	1.07293800	-0.24927100	-0.77701500
C	1.93410100	0.90557300	-0.12994600
O	0.96307700	1.58039200	0.71474700
C	-1.60065900	1.96744300	0.74807600
H	-2.41108300	1.23112500	0.81797600
C	2.43107000	1.93981100	-1.14985100
H	2.82958100	2.80397000	-0.61127100
H	3.22404100	1.53464400	-1.78579600
H	1.61721900	2.28757400	-1.79225600
C	3.09504000	0.43950700	0.74710900
H	3.60329300	1.30887100	1.17418600
H	2.74973800	-0.18605100	1.57173900
H	3.82676300	-0.12595400	0.16044000
C	1.04963200	-1.53553100	0.06115400
H	0.80495500	-1.32512500	1.10615400
H	0.27972000	-2.20222300	-0.33682100
H	2.00975900	-2.05929100	0.02819400
C	1.41261600	-0.57991700	-2.22937600
H	2.44155900	-0.94446300	-2.31683400
H	0.74383100	-1.36561900	-2.59248100
H	1.29386300	0.28827000	-2.88002000
H	-1.44057700	2.39034400	1.74730800
Si	-2.21350700	3.36804400	-0.39050500
C	-2.68247500	2.64331500	-2.07677100
H	-3.53708700	1.96255100	-1.99536500

H	-2.95048100	3.42983700	-2.79116700
H	-1.85203500	2.06782200	-2.49898500
C	-3.72205700	4.21998200	0.39882400
H	-3.38814200	4.71457200	1.32171400
H	-4.03659700	5.03320300	-0.27070800
C	-4.92459500	3.31075000	0.70644400
H	-4.65763300	2.51478700	1.41037000
H	-5.75440800	3.87218200	1.15115200
H	-5.30834900	2.82890900	-0.19959700
C	-0.85005700	4.66634900	-0.60849900
H	-0.55610100	5.09820900	0.35465800
H	0.05044100	4.23766200	-1.06063500
H	-1.18588800	5.48754500	-1.25188600

TS1c

Ir	0.99457800	-0.00975800	-0.01982400
N	-0.17836900	-1.81373100	0.79513100
C	-0.46411700	-1.75522600	2.12093800
C	-1.17975700	-2.78383700	2.78413000
C	-1.61418200	-3.91194900	2.03145500
C	-1.31197900	-3.94799700	0.67039300
C	-0.59095800	-2.87168100	0.10913500
C	-0.00730100	-0.59849900	2.85042300
N	0.67616600	0.36045700	2.16407000
C	1.10642400	1.42838000	2.83102200
C	0.89741300	1.63357100	4.21073100
C	0.19984200	0.66802800	4.93543200
C	-0.26822400	-0.48234200	4.23881600
B	2.85115800	-0.84520400	0.56239800
O	3.96308200	-0.12872600	0.99421700
C	5.07000000	-1.04220900	1.19595800
C	4.33053900	-2.41445700	1.41362700
O	3.09548200	-2.21693600	0.68329500
B	1.70828200	-0.92381200	-1.73543300
O	2.95446000	-0.79783700	-2.33959800
C	2.93519800	-1.47494300	-3.61722300
C	1.79667500	-2.53633400	-3.40860900
O	0.94118500	-1.86395100	-2.44930100
B	1.99482100	1.68838500	-0.54097200
O	2.33438900	2.11228100	-1.82648700
C	3.19436900	3.26835800	-1.73297400
C	2.78025900	3.87262300	-0.34305900
O	2.35921500	2.69083600	0.37708400
H	-0.33112900	-2.86763400	-0.94516300
H	1.64845500	2.15604100	2.23487000
C	-0.06340700	0.81436800	6.41433300
H	-1.13885400	0.83074700	6.62581000
H	0.36203000	-0.02403100	6.97751400
H	0.36545500	1.73252300	6.81494000
C	1.44589000	2.89799900	4.83108300
H	0.65343900	3.51288600	5.27217300
H	2.17055500	2.68111700	5.62378900
H	1.95414300	3.50326200	4.07707200
C	-1.71862400	-5.08528200	-0.23758100
H	-2.80667500	-5.20912600	-0.27388800
H	-1.37478600	-4.90095300	-1.25809200
H	-1.29131100	-6.04017500	0.08829700
C	-2.37862600	-5.02278100	2.71020500
H	-2.62822300	-5.82689900	2.01840500
H	-1.79800900	-5.45939300	3.53070400
H	-3.31657200	-4.65335600	3.14077800
C	-1.43150400	-2.63293400	4.18856800
H	-1.97950400	-3.40757700	4.71315600
C	-0.99763600	-1.53986000	4.87912600
H	-1.20881600	-1.46569300	5.93988300
C	-1.06181800	1.05561400	-0.36165400
H	0.14539400	0.26816900	-1.34502400
H	-0.83758700	2.02184400	0.10309600
C	2.93836400	4.17130500	-2.93927800

H	3.51921500	5.09794600	-2.86947900
H	3.24068400	3.65309600	-3.85433300
H	1.88131100	4.42660200	-3.03423600
C	1.56818100	4.81270000	-0.43019400
H	1.20883900	5.02772200	0.58090700
H	1.82166800	5.76289200	-0.91132900
H	0.75180900	4.34663600	-0.98809800
C	3.90807000	4.54667900	0.43826700
H	4.30878200	5.40556900	-0.11144400
H	3.52690000	4.91283700	1.39727800
H	4.72296500	3.85050200	0.64311100
C	4.64288900	2.75574000	-1.75543000
H	4.78829700	2.16023800	-2.66069400
H	5.36909600	3.57559600	-1.75776200
H	4.83708500	2.10813900	-0.89665200
C	5.91958200	-1.00696500	-0.08246700
H	6.26935000	0.01616900	-0.24721700
H	6.79559700	-1.65958900	-0.00848400
H	5.32413800	-1.29667900	-0.95122000
C	5.89512800	-0.55081000	2.38545300
H	6.71504800	-1.24226900	2.60982000
H	6.33189000	0.42394700	2.14879600
H	5.28239300	-0.43394400	3.28148900
C	3.94524800	-2.67004500	2.87912200
H	3.27200200	-3.53117400	2.92307000
H	4.81954100	-2.88439300	3.50225000
H	3.41988500	-1.80840100	3.30053000
C	5.04564900	-3.64038000	0.84634400
H	6.00915700	-3.80147200	1.34274600
H	4.43098200	-4.53137500	1.00764800
H	5.22104700	-3.54166400	-0.22637100
C	2.59535700	-0.42130000	-4.68258200
H	3.31817100	0.39524500	-4.61021800
H	2.63768700	-0.83664500	-5.69487100
H	1.60128900	0.00184200	-4.51761500
C	0.97237900	-2.86448600	-4.65268100
H	1.59942800	-3.31425300	-5.43045800
H	0.18822600	-3.58406400	-4.39730000
H	0.49034600	-1.97579700	-5.06382100
C	2.29253800	-3.83070300	-2.74643600
H	1.42943600	-4.44701400	-2.47566200
H	2.92505300	-4.41627300	-3.42129900
H	2.85019000	-3.61354900	-1.83166400
C	4.32335600	-2.05478600	-3.88775400
H	5.04821700	-1.24062200	-3.98041100
H	4.65289100	-2.70841700	-3.07787100
H	4.33612200	-2.62537100	-4.82302400
H	-1.68087000	0.47582900	0.33169300
Si	-2.02293600	1.34198200	-1.95750100
C	-1.01596900	2.40046800	-3.16752100
H	-0.94420500	3.43391100	-2.80755800
H	0.00699600	2.02745700	-3.27813800
H	-1.48483000	2.43243000	-4.15801500
C	-2.45331500	-0.31230600	-2.80212800
H	-2.87116000	-0.08658700	-3.79379700
H	-1.51568500	-0.85329800	-2.98482600
C	-3.42887200	-1.22074400	-2.03300400
H	-3.02981200	-1.49766300	-1.05070000
H	-4.39392800	-0.72939700	-1.86459700
H	-3.63132000	-2.15327100	-2.57483500
C	-3.64075100	2.27180400	-1.57223000
H	-3.43336200	3.25345200	-1.13058300
H	-4.23509700	2.43887400	-2.47879200
H	-4.26790800	1.72089300	-0.86245000

INT2c

Ir	0.94014100	0.04316600	-0.05499500
N	-0.30370700	-1.76337900	0.69559500
C	-0.51930100	-1.74559200	2.03510800

C	-1.31265600	-2.72252200	2.68786500
C	-1.90810100	-3.75379900	1.90815600
C	-1.66943900	-3.75559500	0.53438800
C	-0.85757800	-2.73834300	-0.01289000
C	0.09808900	-0.68759700	2.79123800
N	0.84677500	0.23004300	2.11383600
C	1.42844700	1.20716600	2.80793300
C	1.31506300	1.35604500	4.20451200
C	0.55171500	0.43467200	4.92062400
C	-0.07600000	-0.61726000	4.19613800
B	2.83114900	-0.83394000	0.46100200
O	3.92981400	-0.06153100	0.81709300
C	5.02675000	-0.93018100	1.19464400
C	4.27971300	-2.26676200	1.56571100
O	3.06400800	-2.17148600	0.77920800
B	1.83355200	-1.02760800	-1.61917100
O	3.01571400	-0.78228900	-2.31051300
C	3.00769200	-1.55925600	-3.53222800
C	2.04816200	-2.75070800	-3.16104600
O	1.17732800	-2.13414100	-2.18175000
B	1.91417400	1.76904200	-0.53719500
O	2.25402100	2.18614800	-1.82178200
C	3.13152000	3.33038500	-1.72730700
C	2.73701500	3.93397700	-0.32987200
O	2.28771300	2.75654900	0.38512600
H	-0.63103000	-2.72641800	-1.07244800
H	2.00100500	1.91652700	2.22134200
C	0.37974700	0.53079800	6.41673000
H	-0.67624700	0.64513600	6.68702500
H	0.74369200	-0.37399600	6.91676500
H	0.92067900	1.37935700	6.83471700
C	2.02880800	2.52097200	4.85103300
H	1.32971500	3.19413600	5.35960800
H	2.76173300	2.18904000	5.59490000
H	2.56293000	3.10613500	4.09911000
C	-2.23849100	-4.79575900	-0.40248500
H	-3.33406800	-4.80136600	-0.38835700
H	-1.92439500	-4.59770500	-1.42995800
H	-1.90338500	-5.80609800	-0.14236000
C	-2.76528500	-4.80324800	2.57313600
H	-3.15967700	-5.52167400	1.85500700
H	-2.19585400	-5.36356700	3.32370100
H	-3.61834900	-4.34875900	3.08981400
C	-1.47528500	-2.62046200	4.10943600
H	-2.07981500	-3.35732900	4.62618300
C	-0.88630300	-1.62091600	4.82564000
H	-1.03303200	-1.58027600	5.89888600
C	-0.94888600	1.21022700	0.07262700
H	0.66282300	0.12051700	-1.62716400
H	-0.70247900	2.20668800	0.46428200
C	2.87675000	4.24384100	-2.92580200
H	3.47458300	5.15971400	-2.85806400
H	3.15948600	3.72495700	-3.84665500
H	1.82336900	4.51814400	-3.00643800
C	1.54899400	4.90406900	-0.40060000
H	1.20244300	5.11768300	0.61498300
H	1.82457600	5.85151600	-0.87503700
H	0.71724700	4.46393400	-0.95595300
C	3.88539900	4.57089400	0.45248400
H	4.30674000	5.42283000	-0.09237900
H	3.51689100	4.94046000	1.41508800
H	4.68330800	3.85247100	0.64833700
C	4.57133400	2.79507700	-1.76720700
H	4.70013600	2.21002000	-2.68172300
H	5.31006200	3.60364900	-1.76404200
H	4.76111000	2.13105300	-0.92004300
C	5.93952200	-1.05911900	-0.03231300
H	6.29475300	-0.06419000	-0.31509600
H	6.81115000	-1.68976700	0.17068400

H	5.39079200	-1.46587100	-0.88420200
C	5.79147200	-0.27301900	2.34408000
H	6.60433200	-0.91766900	2.69648400
H	6.23293800	0.66611900	1.99788300
H	5.13690900	-0.04521500	3.18750600
C	3.85041500	-2.33755200	3.03908300
H	3.17849100	-3.19055200	3.17068500
H	4.70743400	-2.46892300	3.70743100
H	3.31158500	-1.43440100	3.33805900
C	5.01317800	-3.55200700	1.18320700
H	5.95815700	-3.64114400	1.73030100
H	4.39351100	-4.41758000	1.43611700
H	5.22792900	-3.59258400	0.11394100
C	2.44706000	-0.65068900	-4.63632500
H	3.05602200	0.25532600	-4.68944500
H	2.46393600	-1.14067000	-5.61511500
H	1.42085000	-0.34646300	-4.41423700
C	1.19049700	-3.27337600	-4.31354900
H	1.81740300	-3.68108100	-5.11417600
H	0.54147200	-4.07884200	-3.95565400
H	0.55534900	-2.49075000	-4.73231100
C	2.76242600	-3.91840800	-2.46606000
H	2.01123200	-4.61908400	-2.08957400
H	3.42067400	-4.45966200	-3.15314000
H	3.34331800	-3.56595200	-1.61114800
C	4.44240200	-1.96151600	-3.86954700
H	5.04112000	-1.06393000	-4.04983700
H	4.90938100	-2.52125000	-3.05698400
H	4.47326800	-2.57587500	-4.77608900
H	-1.55647400	0.72163000	0.84963600
Si	-2.00439900	1.40315100	-1.46487400
C	-2.33680500	-0.26320800	-2.32244600
H	-1.40490400	-0.73367600	-2.65302300
H	-2.83715300	-0.96354400	-1.64282700
H	-2.98299900	-0.13742600	-3.19951000
C	-3.73233100	2.11208500	-1.02487000
H	-4.31876400	2.20418000	-1.95081500
H	-4.25956200	1.36105600	-0.41845700
C	-3.72954300	3.45707900	-0.27925000
H	-3.17407600	3.38787900	0.66308000
H	-3.25626800	4.24625900	-0.87493900
H	-4.74368600	3.79990100	-0.03769900
C	-1.20184100	2.57525400	-2.72839600
H	-0.16300900	2.29680600	-2.93002700
H	-1.75197700	2.57326900	-3.67701600
H	-1.19611100	3.60798700	-2.35880300

INT2c-ISO

Ir	1.24948500	-0.27079900	-0.37838900
N	-0.39321100	-1.54870600	0.23088000
C	-0.49082600	-1.82775800	1.56289200
C	-1.46936400	-2.71703300	2.06979600
C	-2.39246800	-3.31637800	1.16721400
C	-2.28892200	-2.99202300	-0.18516200
C	-1.27038300	-2.10974500	-0.59666900
C	0.43542800	-1.18337700	2.45527400
N	1.32214800	-0.31197700	1.90718500
C	2.19657300	0.28250600	2.70901400
C	2.26266700	0.06994500	4.10215100
C	1.35040100	-0.80788900	4.69010500
C	0.40388500	-1.45468600	3.84552900
B	1.16564500	-0.56946900	-2.39633700
O	2.25053500	-0.73408100	-3.25793400
C	1.77944700	-0.69829900	-4.62350900
C	0.27400500	-1.13439400	-4.46858200
O	-0.02868300	-0.70348400	-3.11913000
B	2.42112400	-2.03772900	-0.36844600
O	3.76702000	-2.12254400	-0.02578600
C	4.14223000	-3.51603300	0.09196100

C	3.03541800	-4.23338400	-0.76664200
O	1.92440500	-3.30368900	-0.67450600
B	2.22158100	1.54710800	-0.71011800
O	2.41337300	2.18163300	-1.92932300
C	3.37063700	3.25151800	-1.75077700
C	3.22583500	3.57608000	-0.21592300
O	2.78629700	2.30069500	0.32324500
H	-1.15682700	-1.84160400	-1.64163200
H	2.86685800	0.98474700	2.22418000
C	1.34510600	-1.07984100	6.17479700
H	0.37745100	-0.82347500	6.62087800
H	1.52544700	-2.14051800	6.38478900
H	2.11058600	-0.50614300	6.69674900
C	3.31333700	0.81825400	4.88919300
H	2.86602200	1.47533100	5.64337700
H	3.99379000	0.13661800	5.41149800
H	3.91671600	1.44132400	4.22483500
C	-3.21634000	-3.54800800	-1.24067600
H	-4.26073200	-3.28115600	-1.04486900
H	-2.95511400	-3.15484800	-2.22584100
H	-3.16201900	-4.64094700	-1.29568000
C	-3.44656200	-4.26465000	1.68366400
H	-4.08844800	-4.63438300	0.88450100
H	-2.99167800	-5.13377700	2.17275300
H	-4.08849200	-3.77639200	2.42565200
C	-1.47984400	-2.96818400	3.48357000
H	-2.21729000	-3.65134900	3.88941200
C	-0.58737400	-2.37397800	4.32696100
H	-0.63016500	-2.59799000	5.38694900
C	-0.39562200	1.31826400	-0.61598800
H	2.79275300	0.03440300	-0.64671500
C	3.40197900	-4.34650300	-2.25342300
H	4.22346300	-5.05077500	-2.41939500
H	2.52866900	-4.70689400	-2.80545100
H	3.67966000	-3.37152100	-2.66176500
C	2.57859400	-5.59244300	-0.23717800
H	1.78395800	-5.98638600	-0.87800500
H	3.40295200	-6.31431700	-0.24085600
H	2.18545900	-5.51893700	0.77863100
C	5.57362300	-3.67758200	-0.42008400
H	6.25751900	-3.11584600	0.22336600
H	5.88295100	-4.72869300	-0.40573800
H	5.68075400	-3.29493100	-1.43653900
C	4.08004600	-3.87106100	1.58548000
H	4.41438300	-4.89575300	1.77762500
H	4.73221600	-3.18765500	2.13681100
H	3.06449100	-3.75599200	1.97450000
C	0.07651000	-2.65711500	-4.51392700
H	0.25582600	-3.06318600	-5.51449300
H	0.74052900	-3.15821800	-3.80483100
H	-0.95599800	-2.89212300	-4.23598500
C	-0.70437500	-0.45174100	-5.42421600
H	-1.72335800	-0.79141300	-5.21376000
H	-0.68286700	0.63322100	-5.30951900
H	-0.47526300	-0.69942600	-6.46663200
C	2.64282100	-1.63877500	-5.46505300
H	2.28866700	-1.67800000	-6.50128400
H	3.67423800	-1.27392400	-5.47562200
H	2.64972100	-2.65260200	-5.06072400
C	1.94476500	0.74476200	-5.12348700
H	1.36792000	1.44164700	-4.51156700
H	2.99814400	1.02499600	-5.03496300
H	1.64619900	0.85095200	-6.17166900
C	2.13306900	4.61166200	0.07919800
H	1.96412300	4.66084700	1.15829300
H	2.42086800	5.60953100	-0.26635500
H	1.18934400	4.33662800	-0.39796900
C	4.52120200	3.97393500	0.49159400
H	4.32077700	4.14879000	1.55328100

H	5.27916800	3.19222100	0.41531300
H	4.92978800	4.89945200	0.07144900
C	4.75061500	2.68863800	-2.12399900
H	5.03544300	1.86357100	-1.46521200
H	4.70712200	2.29901000	-3.14446600
H	5.52945300	3.45677100	-2.07952400
C	3.00396500	4.39533400	-2.69586200
H	3.66311400	5.25733000	-2.54559400
H	3.11535100	4.06241900	-3.73186400
H	1.97027800	4.71680500	-2.55660900
H	-0.04866300	2.12658400	-1.26968500
Si	-1.40182700	2.11973400	0.75582200
C	-0.44308800	2.60066700	2.32947100
H	0.55865100	2.96698700	2.08790500
H	-0.97265700	3.38758600	2.87991500
H	-0.32479800	1.74903900	3.00686100
C	-2.16547900	3.75338900	0.09994200
H	-2.76535300	4.20294200	0.90501400
H	-1.34542300	4.46333200	-0.08213000
C	-3.02103600	3.62624500	-1.17192400
H	-3.88349400	2.96892900	-1.01085700
H	-3.41165300	4.59548700	-1.50651100
H	-2.44288500	3.20144100	-1.99980900
H	-1.09376200	0.75329900	-1.24613800
C	-2.86328300	1.02095500	1.29920800
H	-3.48101600	0.72772900	0.44280300
H	-2.53040500	0.10294900	1.79440700
H	-3.51055400	1.55596400	2.00486600

TSc-ISO

Ir	0.47244600	-0.48670200	-0.33760700
N	-0.97794100	-2.06636100	0.18736800
C	-1.03336600	-2.47220500	1.48650700
C	-1.93024800	-3.47390300	1.93335100
C	-2.84105500	-4.04934900	1.00713500
C	-2.80607600	-3.58317300	-0.30651300
C	-1.85917300	-2.60211200	-0.65802600
C	-0.15299700	-1.82407700	2.41117800
N	0.61537300	-0.81718700	1.92281700
C	1.46926600	-0.23797100	2.76229300
C	1.59879900	-0.56901100	4.12548700
C	0.78766200	-1.57428400	4.65282400
C	-0.11491000	-2.22492600	3.77012800
B	0.46226500	-0.88200900	-2.37223700
O	1.17150600	-0.17725900	-3.34486600
C	0.74262600	-0.60271000	-4.65672300
C	0.15896100	-2.03250500	-4.36116900
O	-0.27368200	-1.90030500	-2.98115000
B	2.13919500	-1.80520500	-0.43242000
O	3.11896300	-1.90486800	-1.42526400
C	4.20663000	-2.72881000	-0.93848400
C	3.49561600	-3.59759600	0.16205500
O	2.41134100	-2.73439700	0.57732500
B	1.79074000	1.17057100	-0.24300500
O	1.54734400	2.46107900	-0.70082800
C	2.79026900	3.20257200	-0.69240300
C	3.62709700	2.43776300	0.40112800
O	3.04021300	1.10435500	0.36405500
H	-1.79379400	-2.26067900	-1.68103600
H	2.11080900	0.51766200	2.32981500
C	0.85266100	-1.97820100	6.10528700
H	-0.11513400	-1.83601100	6.59972300
H	1.11572300	-3.03695800	6.21077900
H	1.59241600	-1.39855500	6.65696400
C	2.61012800	0.18899000	4.95376400
H	2.13763900	0.72182900	5.78645200
H	3.36815000	-0.47761900	5.37992200
H	3.12924400	0.93089100	4.34318600
C	-3.73397700	-4.08977300	-1.38675300

H	-4.78512100	-3.90459300	-1.13899500
H	-3.52581700	-3.59110900	-2.33620200
H	-3.62175200	-5.16776800	-1.54824900
C	-3.81059700	-5.11502900	1.45542500
H	-4.44542400	-5.45700300	0.63832300
H	-3.28300400	-5.98924700	1.85397600
H	-4.46659300	-4.74466300	2.25159700
C	-1.87636900	-3.85427800	3.31622500
H	-2.54581700	-4.62796500	3.67464400
C	-1.00682300	-3.26857500	4.18728200
H	-0.99575400	-3.58938600	5.22280700
C	-1.30940400	0.92955700	-0.35725300
H	0.99003800	0.54421300	-1.39600200
H	-1.06168300	1.71387800	-1.08421600
C	2.86210000	-4.88304100	-0.39204400
H	3.61689200	-5.62509200	-0.67279500
H	2.22039900	-5.32073700	0.37839700
H	2.24051400	-4.66874400	-1.26569100
C	4.35029300	-3.92698600	1.38643400
H	3.75617300	-4.50077300	2.10447700
H	5.22225900	-4.53108700	1.11158500
H	4.69799500	-3.02107300	1.88655800
C	4.79473300	-3.51488200	-2.11015300
H	5.22341500	-2.82047300	-2.83921500
H	5.59390700	-4.18477900	-1.77323200
H	4.03612900	-4.11129200	-2.62023600
C	5.26792200	-1.77537500	-0.36898000
H	6.15341300	-2.31232200	-0.01299000
H	5.57934800	-1.08670100	-1.15953100
H	4.85624400	-1.17817100	0.44794400
C	1.22352900	-3.13781400	-4.39086800
H	1.57740700	-3.33311500	-5.40824300
H	2.07483300	-2.86916500	-3.76087000
H	0.78563100	-4.06096200	-3.99874300
C	-1.04619000	-2.43466500	-5.20950300
H	-1.39809800	-3.42500400	-4.90362400
H	-1.87306300	-1.73077800	-5.09630400
H	-0.77774800	-2.48828800	-6.27007500
C	1.94823900	-0.57555900	-5.59557200
H	1.68508900	-0.96280500	-6.58615300
H	2.29337000	0.45543400	-5.71749900
H	2.77726300	-1.16220100	-5.19635200
C	-0.31864100	0.40088000	-5.13281500
H	-1.19130800	0.39599600	-4.47388400
H	0.11225800	1.40554100	-5.10890500
H	-0.64938300	0.19208600	-6.15534800
C	3.42872300	2.99370500	1.81728800
H	3.91571300	2.32513900	2.53410300
H	3.87978000	3.98457700	1.92705400
H	2.36875900	3.06601000	2.07502400
C	5.12010100	2.30761600	0.10155500
H	5.60747100	1.73471500	0.89604300
H	5.29620700	1.78949200	-0.84211400
H	5.59567700	3.29339200	0.05599100
C	3.38130200	3.08614300	-2.10570900
H	3.57856600	2.04327300	-2.36785200
H	2.65329800	3.47625100	-2.82201400
H	4.30794100	3.65970400	-2.20772500
C	2.48359400	4.66598600	-0.37553100
H	3.40517400	5.25484200	-0.31267500
H	1.86693100	5.08846900	-1.17384600
H	1.93544900	4.77259400	0.56217000
H	-2.11144300	0.33586300	-0.82520000
Si	-2.06812700	1.79843000	1.11220500
C	-2.97484600	0.60395000	2.29312500
H	-2.28685900	-0.06919600	2.81430600
H	-3.53943300	1.15228100	3.05662200
H	-3.69121500	-0.01841400	1.74352500
C	-0.78786600	2.78804300	2.13404900

H	-0.11651600	2.06840400	2.62184300
H	-0.16255600	3.34389300	1.42292200
C	-1.36397100	3.74658100	3.19007900
H	-1.98522100	3.21572800	3.92123100
H	-0.57715900	4.26562000	3.75285500
H	-1.99440600	4.51653800	2.73213100
C	-3.39346600	3.04066300	0.51447700
H	-2.95108000	3.81014800	-0.12947900
H	-4.16072300	2.52692100	-0.07700900
H	-3.90200500	3.55050800	1.34166000

TS2c-ISO

Ir	1.25967500	-0.31459000	-0.63547800
N	-0.48032700	-1.54016000	-0.12451500
C	-0.75807200	-1.69273900	1.20229100
C	-1.84123000	-2.48779100	1.65213800
C	-2.65802100	-3.15245500	0.69439900
C	-2.33930800	-2.99952600	-0.65363100
C	-1.24235000	-2.18622800	-1.00341400
C	0.09671100	-1.03202100	2.15684000
N	1.12464000	-0.29334000	1.67018600
C	1.92769900	0.31632100	2.53113400
C	1.78692700	0.24126800	3.93383300
C	0.73632100	-0.51066500	4.45872800
C	-0.13923500	-1.16740100	3.54763200
B	1.37356800	-0.62305600	-2.64954500
O	2.40725500	-0.22207000	-3.48900600
C	2.08164100	-0.54228800	-4.85633400
C	1.02946600	-1.69744600	-4.68438800
O	0.43975200	-1.36984300	-3.40087900
B	2.38402600	-2.08080500	-0.53196700
O	3.52805600	-2.42365400	-1.24935900
C	4.14465000	-3.57731200	-0.62906100
C	2.93274800	-4.23765600	0.12616100
O	2.07630400	-3.09712700	0.37685300
B	1.99914400	1.64475500	-0.80465400
O	2.18971900	2.40660400	-1.95345300
C	3.28200500	3.32155100	-1.71971800
C	3.22145900	3.51391800	-0.15863500
O	2.68713500	2.24137200	0.27559700
H	-0.95945400	-2.05891600	-2.04233300
H	2.71027500	0.92107400	2.08534600
C	0.51026400	-0.63901000	5.94567000
H	-0.47612400	-0.25530400	6.23049300
H	0.55177400	-1.68700600	6.26370800
H	1.25589700	-0.09003700	6.52025500
C	2.78085500	0.98771700	4.79334700
H	2.29173200	1.73694300	5.42578800
H	3.33493200	0.31340600	5.45587700
H	3.51019300	1.50929400	4.16898900
C	-3.11685100	-3.65942600	-1.76884400
H	-4.16447800	-3.33868700	-1.77874700
H	-2.68566400	-3.40293400	-2.73907100
H	-3.10984300	-4.75139300	-1.67961500
C	-3.82226500	-3.99783500	1.14982700
H	-4.36832300	-4.42583100	0.30951900
H	-3.48573300	-4.82610700	1.78400100
H	-4.53191200	-3.40781300	1.74065700
C	-2.06090400	-2.59385300	3.06671500
H	-2.88994800	-3.19309800	3.42550000
C	-1.25291500	-1.96727100	3.96850300
H	-1.45405000	-2.07871600	5.02797500
C	-0.16511700	1.57324000	-0.44658700
H	2.76030000	0.13325600	-0.89119200
H	-0.75872700	1.07883600	0.33288300
C	2.13517000	-5.21902000	-0.74534700
H	2.69274800	-6.14135500	-0.93761300
H	1.20842300	-5.48064100	-0.22602200
H	1.86882500	-4.76608200	-1.70390700

C	3.27613000	-4.89396600	1.46350400
H	2.36490400	-5.28855200	1.92357000
H	3.97260500	-5.72864800	1.32593400
H	3.72105700	-4.18017100	2.15920800
C	4.78544800	-4.43871200	-1.71705100
H	5.58383000	-3.87435400	-2.20795900
H	5.22647800	-5.34656100	-1.29031700
H	4.06238700	-4.72988800	-2.48118000
C	5.23176000	-3.04451300	0.31775500
H	5.80263800	-3.85326000	0.78535500
H	5.92317500	-2.42199600	-0.25684400
H	4.79625400	-2.42379000	1.10590400
C	1.67846700	-3.08251500	-4.53975200
H	2.11284500	-3.43057500	-5.48241400
H	2.45943800	-3.06216000	-3.77527000
H	0.91402600	-3.80302500	-4.23156100
C	-0.07698900	-1.74369400	-5.73734700
H	-0.77259200	-2.55719100	-5.50696000
H	-0.64594000	-0.81272800	-5.76856500
H	0.33790700	-1.93188500	-6.73370500
C	3.36849600	-0.94113800	-5.58063800
H	3.16110200	-1.26940300	-6.60545100
H	4.03954600	-0.07848200	-5.63236700
H	3.89218400	-1.74010200	-5.05319100
C	1.49155500	0.72052100	-5.50110300
H	0.56179800	1.01646500	-5.00939000
H	2.20537300	1.54033900	-5.38436100
H	1.29493700	0.58030900	-6.56931200
C	2.24398600	4.61473500	0.27937000
H	2.08255300	4.53767500	1.35889900
H	2.63508700	5.61385100	0.06215500
H	1.27648400	4.50843300	-0.21692500
C	4.57473600	3.72641000	0.52109900
H	4.43450900	3.83196200	1.60187600
H	5.24611200	2.88359500	0.34734000
H	5.05812900	4.63926000	0.15644800
C	4.56903700	2.62706600	-2.19413900
H	4.77764200	1.72819800	-1.60796600
H	4.43289700	2.31485800	-3.23231400
H	5.43693400	3.29230900	-2.13665600
C	3.05158200	4.59048600	-2.53995900
H	3.83265400	5.33205700	-2.33884200
H	3.08436500	4.34781900	-3.60613700
H	2.07967400	5.03970500	-2.33006600
H	0.21000000	2.46271900	0.07564700
Si	-1.39518300	2.18282000	-1.74550500
C	-0.64277700	3.34960800	-3.03747000
H	0.23285100	2.90941500	-3.51854400
H	-1.37947200	3.61323900	-3.80548900
H	-0.31648900	4.28439800	-2.56755400
C	-2.28681500	0.74973000	-2.63187000
H	-1.53147400	0.12504300	-3.12216300
H	-2.74319600	0.11336400	-1.86035600
C	-3.36399400	1.16881900	-3.64813000
H	-2.94063400	1.77324700	-4.45828000
H	-3.84693200	0.30056200	-4.11411200
H	-4.15562800	1.76608100	-3.18109900
C	-2.72939300	3.19090700	-0.82024600
H	-3.26746900	2.57419200	-0.09048200
H	-2.28215400	4.02916500	-0.27293800
H	-3.47096900	3.60983800	-1.51086600

TS2c

Ir	1.74135500	-0.17273900	-0.20146800
N	-0.17618200	-1.33080800	0.07390300
C	-0.59441100	-1.45763000	1.35864900
C	-1.74857700	-2.20614300	1.69904100
C	-2.46960500	-2.86689300	0.66434100
C	-2.00500200	-2.74167700	-0.64545300

C	-0.85509900	-1.95894200	-0.87436200
C	0.17973200	-0.80372900	2.38501100
N	1.30271500	-0.13791300	2.00246300
C	1.99569200	0.50732800	2.93226500
C	1.65684900	0.52791800	4.30187200
C	0.52965100	-0.17656800	4.72298600
C	-0.23613700	-0.86292700	3.73782900
B	3.79847300	-0.00456500	0.03752900
O	4.79483200	0.37586100	-0.85661100
C	6.10077900	0.14223600	-0.27615300
C	5.77858600	0.04282100	1.26720800
O	4.37817600	-0.31634200	1.27744000
B	2.54272700	-2.13178500	-0.29810200
O	3.65502800	-2.65379800	-0.95493600
C	3.76184600	-4.07623900	-0.68629300
C	2.29518000	-4.44386000	-0.26367500
O	1.81398500	-3.18847400	0.26741000
B	2.25349100	1.83738400	-0.70272800
O	2.23426800	2.38721300	-1.99200500
C	3.08023500	3.55746700	-2.04192300
C	3.18583300	3.96572200	-0.51994700
O	2.94916100	2.71020400	0.16388000
H	-0.47102100	-1.84295500	-1.88172500
H	2.87726600	1.02515400	2.57810500
C	0.10919100	-0.22055500	6.17178900
H	-0.89291100	0.20243400	6.30708000
H	0.07638000	-1.25114600	6.54309600
H	0.79196000	0.33860600	6.81097500
C	2.54324000	1.31018900	5.24285700
H	1.98946300	2.09909100	5.76377400
H	2.99316200	0.66700900	6.00743700
H	3.35747600	1.78725100	4.69259400
C	-2.68019600	-3.39150500	-1.83076200
H	-3.71231800	-3.04475400	-1.95087500
H	-2.14597000	-3.15611000	-2.75438300
H	-2.70977400	-4.48249800	-1.73580200
C	-3.69996700	-3.67341400	1.00001800
H	-4.13232000	-4.14500300	0.11797300
H	-3.46925100	-4.46534000	1.72129700
H	-4.47385700	-3.04193400	1.45142100
C	-2.13293900	-2.26023600	3.08071300
H	-3.01618000	-2.82281600	3.36121400
C	-1.41820400	-1.61637900	4.04741500
H	-1.74849300	-1.67805400	5.07822400
C	0.25438900	1.64874600	-0.25074200
H	1.90858200	-0.27767700	-1.76395900
H	-0.27743300	1.27169500	0.62788100
C	1.39944900	-4.81273400	-1.45661700
H	1.67253900	-5.78164200	-1.88637000
H	0.36074600	-4.86972800	-1.11835500
H	1.46207000	-4.05454700	-2.24226100
C	2.16695200	-5.51029800	0.82471600
H	1.10966100	-5.66195200	1.06306600
H	2.57805800	-6.46956600	0.49132600
H	2.67688600	-5.21100600	1.74197500
C	4.27258800	-4.76370600	-1.95318300
H	5.28602700	-4.41731700	-2.17517500
H	4.30807400	-5.85129200	-1.82400000
H	3.64581000	-4.53355900	-2.81644900
C	4.76878500	-4.27772000	0.45572600
H	4.88323800	-5.33747400	0.70417200
H	5.74809800	-3.90103000	0.15163600
H	4.45865800	-3.73716400	1.35360300
C	5.90005800	1.38205200	2.01098000
H	6.93527600	1.73409500	2.06262500
H	5.28386700	2.14761600	1.53507300
H	5.54182100	1.24673500	3.03705900
C	6.56328900	-1.03159700	2.02172000
H	6.23289100	-1.06141000	3.06481100

H	6.40795000	-2.02122700	1.59122500
H	7.63671100	-0.81192300	2.01518400
C	7.04716500	1.28563800	-0.65121500
H	8.03957100	1.11490900	-0.21906200
H	7.15935100	1.32889500	-1.73825900
H	6.68951800	2.25594000	-0.30748500
C	6.64162700	-1.15347100	-0.89552900
H	5.95139500	-1.97734300	-0.72698200
H	6.72401100	-1.01272700	-1.97729000
H	7.63265000	-1.40872700	-0.50607500
C	2.09981400	4.95698200	-0.06741900
H	2.11709500	5.02077300	1.02488100
H	2.27569400	5.95927600	-0.47099800
H	1.09983400	4.64101300	-0.37167500
C	4.54856300	4.51604100	-0.09967300
H	4.54973700	4.72367800	0.97512600
H	5.35071900	3.81071100	-0.31370600
H	4.76658700	5.45362500	-0.62289900
C	4.41274500	3.12966500	-2.67269300
H	4.86640900	2.31978800	-2.10367700
H	4.21188400	2.75338700	-3.68036800
H	5.11307900	3.96767200	-2.75528300
C	2.43022800	4.61251600	-2.94100100
H	3.06909400	5.49961300	-3.01744600
H	2.30545500	4.20253500	-3.94751800
H	1.45077000	4.92727500	-2.58102800
H	0.53032100	2.65679800	0.07218700
Si	-0.98287300	1.83716200	-1.68238800
C	-1.31244000	3.67772200	-2.04608700
H	-0.46385300	4.14366600	-2.55351400
H	-2.18494500	3.78743200	-2.70172400
H	-1.51429300	4.25064800	-1.13381500
C	-0.47459400	1.02724800	-3.31805000
H	0.52437400	1.37359000	-3.59454200
H	-0.43498600	-0.06470000	-3.25638800
C	-2.69194900	1.12869100	-1.17787600
H	-3.36056100	1.27128300	-2.03971700
H	-2.61771200	0.04320700	-1.04291100
H	-1.17742100	1.29266900	-4.11712200
C	-3.33597500	1.75418300	0.07195100
H	-3.47707100	2.83441900	-0.04529600
H	-4.32083100	1.32141100	0.28873000
H	-2.71348900	1.60583000	0.96202400

1d

C	-1.04016100	0.86119600	-0.21098800
H	-0.94277300	1.91501100	0.06813700
H	-1.43985400	0.32012000	0.65380700
Si	-2.16472500	0.66060400	-1.71285000
C	-3.89987000	1.30861900	-1.35127300
H	-4.35055700	0.77503400	-0.50712700
H	-3.87695100	2.37387200	-1.10117000
H	-4.56232700	1.18280300	-2.21466400
C	-1.42547400	1.63818000	-3.18267400
H	-0.41901200	1.29630700	-3.43723600
H	-2.04255400	1.55743600	-4.08116800
C	-2.23279400	-1.15134500	-2.26000400
H	-1.23707900	-1.54030800	-2.49956700
H	-2.64825000	-1.78048800	-1.46474200
H	-2.86441800	-1.28225100	-3.14553400
Cl	-1.28038500	3.42482000	-2.82443600
H	-0.03461600	0.47391500	-0.40846700

2d

B	1.12002300	3.61844300	-0.87368900
O	1.86406300	3.16499700	-1.93820600
C	3.25897500	3.12064200	-1.52841900
C	3.28177100	4.11430300	-0.30109100
O	1.90718700	4.05467700	0.17107900

C	-0.44184700	3.61906700	-0.83582600
H	-0.80614000	4.63074500	-0.61420400
C	3.54521300	5.57245300	-0.70131000
H	3.34392800	6.21692200	0.15872400
H	4.58289200	5.72699800	-1.01212400
H	2.88835100	5.88578300	-1.51764400
C	4.20575300	3.71228500	0.84697800
H	4.11948500	4.44052000	1.65849600
H	3.94844900	2.73043900	1.24824700
H	5.25070000	3.69339800	0.51993400
C	3.55967600	1.66581500	-1.14343400
H	2.94896300	1.34331300	-0.29535300
H	3.32424300	1.01906400	-1.99303700
H	4.61325000	1.52406000	-0.88413700
C	4.12468400	3.53500800	-2.71708800
H	5.18250700	3.57088700	-2.43621200
H	4.01374000	2.80436000	-3.52343500
H	3.83372500	4.51165800	-3.10750700
H	-0.84238300	3.33228200	-1.81487900
Si	-1.18261100	2.49348700	0.49407200
C	-0.80780600	3.14633900	2.22234300
H	-1.31900000	4.09844600	2.39656600
H	-1.13270900	2.44368100	2.99758000
H	0.26673900	3.31786800	2.34056000
C	-3.08055900	2.41695200	0.25071300
H	-3.35264300	2.02668700	-0.73324400
H	-3.56209300	1.78874300	1.00472600
C	-0.56851200	0.71148300	0.30158600
H	-0.74218600	0.32748600	-0.70950700
H	0.50793400	0.64831800	0.49592800
H	-1.06732600	0.03745400	1.00684800
Cl	-3.86753000	4.06130400	0.38117200

TS1d

Ir	0.99082600	0.00364100	-0.01132400
N	-0.18096400	-1.79972100	0.80290400
C	-0.45617800	-1.74606900	2.13124700
C	-1.17508700	-2.77192100	2.79372400
C	-1.63102300	-3.88866000	2.03598100
C	-1.34719500	-3.91537900	0.67159000
C	-0.61600100	-2.84455700	0.11263100
C	0.01244400	-0.59593300	2.86328100
N	0.68970500	0.36675500	2.17570000
C	1.13231300	1.42837800	2.84429800
C	0.94173100	1.62418400	4.22795100
C	0.24907100	0.65610900	4.95382600
C	-0.23254700	-0.48762600	4.25523500
B	2.84647400	-0.83191300	0.56484800
O	3.96086400	-0.11871000	0.99379000
C	5.06358900	-1.03667200	1.19974600
C	4.31820900	-2.40622200	1.41739200
O	3.08349400	-2.20382100	0.68765200
B	1.69694100	-0.90157000	-1.73455600
O	2.95694600	-0.80974900	-2.31537400
C	2.94367300	-1.47612700	-3.59895600
C	1.76322600	-2.49793400	-3.42562600
O	0.90990000	-1.80217200	-2.47954300
B	1.98947500	1.70230700	-0.53480300
O	2.31378300	2.12885400	-1.82516600
C	3.18352100	3.27913400	-1.73836700
C	2.78874800	3.88202500	-0.34244500
O	2.36961400	2.70083500	0.37985100
H	-0.37230200	-2.83612400	-0.94462500
H	1.67009400	2.15818300	2.24710800
C	0.00457600	0.79289300	6.43680500
H	-1.06817700	0.81731300	6.66086200
H	0.42850400	-0.05418600	6.98788300
H	0.44664400	1.70365300	6.83994600
C	1.50354000	2.88153000	4.85087200

H	0.71953900	3.49681600	5.30624600
H	2.23689900	2.65555400	5.63295600
H	2.00549200	3.49008900	4.09525600
C	-1.78831500	-5.03270300	-0.24418000
H	-2.87874300	-5.13503200	-0.26159100
H	-1.46387000	-4.83732900	-1.26865100
H	-1.37127200	-5.99929500	0.06030100
C	-2.40266800	-4.99525100	2.71326600
H	-2.66294600	-5.79329500	2.01844400
H	-1.82279200	-5.44133900	3.52915700
H	-3.33529700	-4.61858100	3.14910400
C	-1.41042700	-2.62946100	4.20169300
H	-1.95959800	-3.40347300	4.72597900
C	-0.96092400	-1.54515600	4.89626300
H	-1.16032800	-1.47738900	5.95968500
C	-1.08744600	1.03867900	-0.35583000
H	0.13409400	0.28875100	-1.32868100
H	-0.89029000	2.00122600	0.12776900
C	2.92139800	4.18753600	-2.93919400
H	3.51312800	5.10757700	-2.87463700
H	3.20649200	3.66861600	-3.85935800
H	1.86626900	4.45572900	-3.01995600
C	1.58072400	4.82870100	-0.41348600
H	1.23271500	5.04116000	0.60202000
H	1.83416400	5.77958800	-0.89320700
H	0.75636200	4.36923900	-0.96525700
C	3.92904100	4.54839700	0.42720700
H	4.32958100	5.40472700	-0.12656500
H	3.56048500	4.91672900	1.39024100
H	4.74135800	3.84678400	0.62351500
C	4.62794600	2.75637600	-1.77776400
H	4.76084600	2.16443400	-2.68731600
H	5.35954100	3.57133600	-1.78346600
H	4.82644500	2.10339000	-0.92408800
C	5.91731700	-1.00614200	-0.07597500
H	6.27196300	0.01542200	-0.24043500
H	6.79036900	-1.66223900	0.00182100
H	5.32365300	-1.29470500	-0.94622200
C	5.88712400	-0.54681600	2.39100600
H	6.70384300	-1.24101000	2.61847800
H	6.32813000	0.42603300	2.15436300
H	5.27234200	-0.42666700	3.28516800
C	3.93230800	-2.66093900	2.88280900
H	3.25607200	-3.51968100	2.92653500
H	4.80601500	-2.87852900	3.50559500
H	3.40986800	-1.79783000	3.30480000
C	5.02795500	-3.63484200	0.84918300
H	5.99097100	-3.80023000	1.34509200
H	4.40955600	-4.52323100	1.01040900
H	5.20328300	-3.53625800	-0.22356800
C	2.66784400	-0.40380700	-4.66332800
H	3.42326900	0.38106500	-4.57431100
H	2.71090400	-0.81552900	-5.67694700
H	1.69002000	0.06048100	-4.51296400
C	0.95714400	-2.78811000	-4.69062500
H	1.58432300	-3.26128200	-5.45416300
H	0.13751800	-3.47481600	-4.45743600
H	0.52395500	-1.87913100	-5.11227300
C	2.19823700	-3.81270600	-2.76125800
H	1.30843600	-4.40028400	-2.51468100
H	2.82598400	-4.41523800	-3.42547600
H	2.74277400	-3.61925400	-1.83321200
C	4.31547400	-2.10508000	-3.84140600
H	5.07306700	-1.31836900	-3.90470600
H	4.59814800	-2.78125100	-3.03228500
H	4.33050000	-2.66413100	-4.78356000
H	-1.70790500	0.43017200	0.30927900
Si	-2.01627400	1.32938400	-1.95776100
C	-1.02833700	2.42567400	-3.14792600

H	-1.05359300	3.46698100	-2.80426900
H	0.02324300	2.12638000	-3.19470800
H	-1.44646100	2.40941300	-4.16097700
C	-2.28006200	-0.33807300	-2.86752000
H	-2.73544600	-0.20338800	-3.85192800
H	-1.34357500	-0.88798700	-2.98467800
C	-3.71809600	2.11022800	-1.66005400
H	-3.62056900	3.07383300	-1.14713500
H	-4.24752600	2.28872700	-2.60330300
H	-4.34733200	1.46251100	-1.04174400
Cl	-3.40811100	-1.46756600	-1.95666900

INT2d

Ir	0.95485300	0.16900300	0.49062100
N	-0.22372300	-1.58191200	1.45775300
C	-0.21359800	-1.54666400	2.81378400
C	-0.93487000	-2.47899400	3.60046100
C	-1.69681200	-3.48592100	2.94350400
C	-1.67904600	-3.51428900	1.55021200
C	-0.92208000	-2.54036700	0.86468700
C	0.58116000	-0.52503700	3.44473700
N	1.26218900	0.34388300	2.64325800
C	2.01675700	1.27834200	3.21866800
C	2.14939500	1.43358300	4.61290600
C	1.45688000	0.56504800	5.45574700
C	0.65084500	-0.44431800	4.85799600
B	2.85410500	-0.81694000	0.67675400
O	4.03547600	-0.10637800	0.84479200
C	5.13175500	-1.03540000	1.03548700
C	4.38612900	-2.33791100	1.51726600
O	3.06265600	-2.16795800	0.94615100
B	1.51181900	-0.93604100	-1.20057100
O	2.57485100	-0.73902300	-2.07706500
C	2.33142300	-1.50500500	-3.28220800
C	1.38395900	-2.64873900	-2.76236700
O	0.71794600	-2.00069700	-1.65165800
B	1.91717200	1.84267100	-0.16450800
O	1.98468600	2.28914400	-1.48320700
C	2.94968200	3.36196200	-1.56290600
C	2.91891700	3.94034700	-0.10160200
O	2.55509900	2.77042400	0.67059700
H	-0.86394100	-2.55301500	-0.21808000
H	2.53071500	1.94437900	2.53504300
C	1.54269900	0.67223900	6.95846100
H	0.55651900	0.86164200	7.39764200
H	1.91868900	-0.25705400	7.40124000
H	2.20421500	1.47938500	7.27201100
C	3.03725300	2.54699200	5.11969200
H	2.47881100	3.26573100	5.72958000
H	3.85921300	2.16539100	5.73565300
H	3.47737900	3.09479900	4.28340800
C	-2.44312600	-4.52962500	0.73384800
H	-3.52388000	-4.43753300	0.88779000
H	-2.25055300	-4.38661200	-0.33216300
H	-2.15932500	-5.55703900	0.98683700
C	-2.49283400	-4.47947500	3.75394600
H	-3.01301800	-5.19748600	3.12048700
H	-1.84800900	-5.04448500	4.43663400
H	-3.24687500	-3.97227500	4.36658400
C	-0.85720300	-2.36080100	5.02779800
H	-1.41062500	-3.05857500	5.64602900
C	-0.10273300	-1.39486700	5.62502000
H	-0.06934500	-1.34147400	6.70716300
C	-0.78485800	1.47123900	0.95302600
H	0.40027700	0.26792900	-1.00407500
H	-0.39426000	2.46407700	1.21642900
C	2.51485400	4.33509600	-2.65798700
H	3.19265500	5.19432200	-2.71391600
H	2.53483300	3.82706200	-3.62671300

H	1.49997400	4.70230700	-2.49375300
C	1.81865900	4.98916300	0.11625600
H	1.71816800	5.18110500	1.18866600
H	2.05355200	5.93584800	-0.38082600
H	0.85415400	4.63102800	-0.25302000
C	4.25534300	4.47158100	0.41677300
H	4.60896500	5.30920600	-0.19457100
H	4.13671100	4.83472200	1.44290600
H	5.02015100	3.69304400	0.41969700
C	4.29952600	2.72544800	-1.92895100
H	4.18114100	2.16831900	-2.86205700
H	5.08305900	3.47750300	-2.06954000
H	4.61484600	2.01750400	-1.15829800
C	5.82599200	-1.19401200	-0.32386400
H	6.17854600	-0.21392800	-0.65721700
H	6.68700300	-1.86754000	-0.26644000
H	5.12728800	-1.56517600	-1.07617000
C	6.10601400	-0.43535300	2.04939800
H	6.92311200	-1.13136400	2.26873800
H	6.54355200	0.47940100	1.63880500
H	5.60707900	-0.17737900	2.98544400
C	4.20551700	-2.41106600	3.04115900
H	3.51792800	-3.22869300	3.27585400
H	5.15331200	-2.60057100	3.55500700
H	3.77619800	-1.48576300	3.43455200
C	4.97596400	-3.65260400	1.00757400
H	5.99036600	-3.80278600	1.39327000
H	4.35817700	-4.48878200	1.34849600
H	5.01344200	-3.68367900	-0.08269300
C	1.65410400	-0.55773700	-4.28439000
H	2.29592800	0.31472200	-4.43068800
H	1.49229100	-1.03766600	-5.25494400
H	0.69340200	-0.19899700	-3.90605300
C	0.32159700	-3.11175500	-3.75964200
H	0.78502700	-3.54371600	-4.65329800
H	-0.30323100	-3.88436700	-3.30086200
H	-0.32889900	-2.29098400	-4.06720100
C	2.13842400	-3.86131500	-2.19880500
H	1.42230200	-4.52642400	-1.70722000
H	2.64581500	-4.42780700	-2.98618700
H	2.86921200	-3.55219100	-1.44855000
C	3.67087900	-1.97807900	-3.84448000
H	4.27727100	-1.11169900	-4.12408700
H	4.23404400	-2.56293600	-3.11502800
H	3.52317700	-2.59110900	-4.74034900
H	-1.24581500	1.07860200	1.87033600
Si	-2.14693600	1.70036000	-0.29817700
C	-1.54791800	2.07306900	-2.06349100
H	-0.67483300	2.73255700	-2.05153200
H	-1.23706000	1.15780500	-2.57975500
H	-2.33493100	2.54496400	-2.66275500
C	-3.17643700	0.08938300	-0.50294500
H	-3.94855400	0.19608100	-1.26929400
H	-2.54355200	-0.75842900	-0.77386000
C	-3.36662800	3.06565100	0.21606500
H	-2.85920000	4.03547700	0.27980800
H	-4.19088700	3.17010300	-0.49949800
H	-3.80166500	2.85416100	1.19829600
Cl	-4.06737900	-0.41727000	1.02102500

INT2d-ISO

Ir	1.24228500	-0.28014100	-0.41021700
N	-0.41158000	-1.55505400	0.17546200
C	-0.53893000	-1.82138200	1.50817300
C	-1.53518000	-2.69859500	2.00217600
C	-2.44280700	-3.30089600	1.08538500
C	-2.30510500	-2.99395300	-0.26828300
C	-1.27182100	-2.12250900	-0.66607000
C	0.37352400	-1.17628500	2.41454900

N	1.27820800	-0.31605100	1.87843600
C	2.14070300	0.27721400	2.69400100
C	2.17452500	0.07666700	4.09011100
C	1.24196500	-0.78784600	4.66570400
C	0.30946300	-1.43516400	3.80596100
B	1.19244700	-0.58446200	-2.42896500
O	2.29162500	-0.74626000	-3.27139900
C	1.84260100	-0.71517200	-4.64506700
C	0.33670500	-1.15753000	-4.51361300
O	0.01030200	-0.72396500	-3.16984800
B	2.41351900	-2.04667300	-0.37469100
O	3.75268000	-2.13048000	-0.00932800
C	4.12373800	-3.52436200	0.12250900
C	3.03032500	-4.24490800	-0.75075200
O	1.91938500	-3.31221000	-0.68351000
B	2.23213300	1.53290800	-0.72268200
O	2.43925500	2.17388200	-1.93490400
C	3.38812500	3.24864200	-1.73484500
C	3.21774600	3.56250800	-0.20041100
O	2.77802400	2.28067700	0.32372900
H	-1.13309100	-1.86627800	-1.71099000
H	2.82741400	0.96959100	2.21824800
C	1.19883800	-1.04342200	6.15247600
H	0.22687200	-0.76197200	6.57333400
H	1.35229600	-2.10511300	6.37762300
H	1.96418500	-0.47967000	6.68523200
C	3.21303300	0.82413600	4.89379400
H	2.75340200	1.49372200	5.62926900
H	3.87380400	0.14216800	5.44023200
H	3.83854200	1.43388900	4.23757400
C	-3.21147100	-3.55682600	-1.33828900
H	-4.25844800	-3.28219700	-1.16920000
H	-2.92547300	-3.17703000	-2.32181800
H	-3.16203600	-4.65056300	-1.37889300
C	-3.51693900	-4.23318000	1.58878900
H	-4.14154000	-4.60984300	0.77931400
H	-3.08109900	-5.09807900	2.10195000
H	-4.17287200	-3.72834000	2.30692100
C	-1.57929500	-2.93560200	3.41772900
H	-2.33279700	-3.60652800	3.81393300
C	-0.70022000	-2.34094300	4.27431900
H	-0.76955500	-2.55229200	5.33538000
C	-0.39552000	1.30739100	-0.68987700
H	2.79038100	0.02308500	-0.65565000
H	-0.04271400	2.10629700	-1.35157400
C	3.42196800	-4.36767200	-2.23029200
H	4.24468600	-5.07450700	-2.37793000
H	2.55760700	-4.72979600	-2.79510500
H	3.70908900	-3.39584300	-2.63973500
C	2.56169200	-5.59967600	-0.22086400
H	1.77700700	-5.99588800	-0.87243400
H	3.38458800	-6.32302200	-0.20682600
H	2.15207400	-5.51949400	0.78788500
C	5.56317900	-3.69098000	-0.36453900
H	6.23726900	-3.12796000	0.28802200
H	5.86980300	-4.74264800	-0.34028100
H	5.68841100	-3.31314300	-1.38072500
C	4.03537200	-3.87054600	1.61670600
H	4.36435300	-4.89468300	1.82032800
H	4.67932700	-3.18528600	2.17530600
H	3.01349600	-3.75142400	1.98759000
C	0.14675700	-2.68110000	-4.55744300
H	0.34448800	-3.08912200	-5.55367700
H	0.80126900	-3.17732400	-3.83616600
H	-0.88916600	-2.92022300	-4.29619400
C	-0.62906800	-0.48183300	-5.48671900
H	-1.64989300	-0.82498100	-5.29136500
H	-0.61363400	0.60363700	-5.37576600
H	-0.38259300	-0.73228100	-6.52444700

C	2.72356700	-1.65365600	-5.47022100
H	2.38562600	-1.69752900	-6.51162800
H	3.75323500	-1.28386700	-5.46596900
H	2.72902700	-2.66627000	-5.06284100
C	2.00999100	0.72771700	-5.14447800
H	1.41904700	1.42301600	-4.54422900
H	3.06027800	1.01308700	-5.03792600
H	1.72932600	0.83077600	-6.19785200
C	2.11216400	4.58693100	0.08586000
H	1.92271700	4.62138000	1.16226400
H	2.39849900	5.59105400	-0.24216800
H	1.18009400	4.31110000	-0.41326300
C	4.49942600	3.96478700	0.52875900
H	4.28224300	4.13286200	1.58820800
H	5.26341500	3.18835400	0.45937800
H	4.90814700	4.89500600	0.11940700
C	4.77631900	2.69488300	-2.09030000
H	5.05565300	1.86784400	-1.43160600
H	4.75004800	2.31066200	-3.11340200
H	5.55034100	3.46672800	-2.03027000
C	3.02968800	4.39637300	-2.67808800
H	3.68193900	5.26065100	-2.51204400
H	3.15883200	4.07091300	-3.71437200
H	1.99237000	4.71188300	-2.55289900
H	-1.08967900	0.73762000	-1.32132500
Si	-1.41747400	2.14563500	0.64413700
C	-2.13883200	3.77846700	-0.01184900
H	-2.62746000	3.63390500	-0.98201500
H	-2.87815700	4.19577100	0.68036100
H	-1.35217000	4.52920400	-0.15120000
C	-2.93092100	1.01164900	1.00516900
H	-3.54813600	0.87737400	0.11307400
H	-2.64075900	0.02200400	1.36227100
C	-0.57367800	2.51705500	2.30049200
H	-0.49246600	1.62254800	2.92469500
H	0.43782000	2.90216200	2.14162500
H	-1.14600000	3.26151400	2.86336900
Cl	-4.05811900	1.68239800	2.29127000

TSd-ISO

Ir	0.45683300	-0.49000000	-0.32772300
N	-0.93100900	-2.11588500	0.19239200
C	-1.00579900	-2.49381400	1.49945000
C	-1.87160100	-3.52312000	1.94446400
C	-2.72350100	-4.16501000	1.00555500
C	-2.66301400	-3.73564500	-0.31958800
C	-1.75282300	-2.71912600	-0.66772300
C	-0.17345700	-1.79431500	2.43197800
N	0.57782800	-0.77714200	1.93921900
C	1.39449000	-0.15654300	2.78591000
C	1.49564000	-0.44647500	4.16059500
C	0.69766100	-1.46073100	4.69128300
C	-0.15874800	-2.16171800	3.80069800
B	0.46505900	-0.83753300	-2.36964900
O	1.19388900	-0.10572400	-3.30713900
C	0.77297100	-0.47707800	-4.63900200
C	0.16703300	-1.90891200	-4.40302400
O	-0.27727600	-1.82151800	-3.02192300
B	2.12605400	-1.80334600	-0.40280500
O	3.04009500	-2.00503100	-1.44048000
C	4.12892500	-2.83318500	-0.96129200
C	3.45836800	-3.59499900	0.23877600
O	2.43631200	-2.66070100	0.65812900
B	1.81406400	1.14197400	-0.22189800
O	1.53691600	2.47616100	-0.50214500
C	2.79123200	3.19897700	-0.58709800
C	3.74384300	2.31720000	0.30574200
O	3.12188200	1.00160600	0.21902200
H	-1.66680200	-2.40255700	-1.69796600

H	2.02987400	0.60582300	2.35614500
C	0.72995000	-1.82255700	6.15562200
H	-0.25653600	-1.69544100	6.61578100
H	1.02063100	-2.86956300	6.29896300
H	1.43452600	-1.20548000	6.71248400
C	2.45976300	0.36342200	4.99572100
H	1.94530200	0.91013000	5.79355200
H	3.21881300	-0.27005600	5.46785900
H	2.98096200	1.09875300	4.37907100
C	-3.52440000	-4.31835700	-1.41642100
H	-4.59174300	-4.17931100	-1.21174800
H	-3.30622600	-3.83660500	-2.37231900
H	-3.35321200	-5.39343000	-1.54119000
C	-3.65697400	-5.26310500	1.45184700
H	-4.24239300	-5.66103700	0.62335400
H	-3.10330100	-6.09745500	1.89744700
H	-4.36022900	-4.90161600	2.21085700
C	-1.84564400	-3.86579800	3.33804800
H	-2.49542100	-4.65590500	3.69664900
C	-1.02617600	-3.22534600	4.21897200
H	-1.03535900	-3.51908000	5.26242000
C	-1.37122000	0.87659700	-0.38982900
H	0.94950700	0.60323000	-1.33966800
H	-1.15297200	1.64203000	-1.14612200
C	2.74088400	-4.88531100	-0.18664300
H	3.44622900	-5.67894700	-0.45396300
H	2.12627700	-5.23835700	0.64665900
H	2.08145200	-4.70148300	-1.03920000
C	4.37644800	-3.87846000	1.42791800
H	3.80821900	-4.37689300	2.21951000
H	5.20439400	-4.53669800	1.14178600
H	4.79083700	-2.95683500	1.84066000
C	4.61576900	-3.72115200	-2.10634600
H	5.02220100	-3.09601100	-2.90722400
H	5.41157600	-4.39544300	-1.77023200
H	3.80747000	-4.32271400	-2.52675900
C	5.25530000	-1.88506800	-0.52426400
H	6.13991000	-2.43176800	-0.18096900
H	5.54442000	-1.26580700	-1.37825800
H	4.91531200	-1.21598200	0.26959000
C	1.21556700	-3.02763400	-4.46408700
H	1.57763500	-3.18822200	-5.48458400
H	2.06252300	-2.79802100	-3.81347600
H	0.75949300	-3.95890800	-4.11394900
C	-1.03614900	-2.26058900	-5.27603100
H	-1.40430100	-3.25698700	-5.01186500
H	-1.85396500	-1.54988900	-5.14274300
H	-0.75869000	-2.27762700	-6.33550100
C	1.98905200	-0.43175000	-5.56339500
H	1.73106100	-0.77487300	-6.57136900
H	2.35094700	0.59784700	-5.64067800
H	2.80463900	-1.04677700	-5.17955900
C	-0.26914200	0.55865100	-5.08777600
H	-1.14782300	0.54334200	-4.43700600
H	0.17626100	1.55531400	-5.02469700
H	-0.59373300	0.39148900	-6.11983500
C	3.74959700	2.73319400	1.78227800
H	4.27114600	1.96788000	2.36516300
H	4.27068000	3.68383900	1.92994000
H	2.73400600	2.83857200	2.17397200
C	5.18027900	2.19652700	-0.20163200
H	5.75431700	1.54722000	0.46591700
H	5.21533600	1.76385800	-1.20241200
H	5.66971300	3.17619600	-0.22329100
C	3.18613600	3.21301600	-2.07162700
H	3.31409300	2.19819700	-2.45786300
H	2.38263800	3.68643600	-2.64232800
H	4.10844300	3.77710800	-2.24149000
C	2.56908300	4.62798500	-0.09379200

H	3.50687700	5.19379700	-0.10212700
H	1.86326900	5.13612500	-0.75683900
H	2.15651900	4.65233000	0.91637200
H	-2.15662000	0.24194000	-0.83078400
Si	-2.15467800	1.77303800	1.04506300
C	-3.01607300	0.63416500	2.29777600
H	-2.32110200	-0.06047500	2.77875500
H	-3.49807900	1.22237800	3.08596000
H	-3.79360800	0.03723500	1.80646200
C	-0.80968700	2.76684500	1.99326900
H	-0.08703000	2.10634700	2.47477100
H	-0.26235700	3.43403900	1.32531500
C	-3.45068000	3.03850900	0.46362400
H	-3.01353200	3.75743300	-0.23869800
H	-4.27373600	2.53384100	-0.05601100
H	-3.87510300	3.60256800	1.30103300
Cl	-1.47635200	3.82159800	3.34332600

TS2d-ISO

Ir	1.26208200	-0.29570400	-0.40596400
N	-0.43793700	-1.52182000	0.18749100
C	-0.54129900	-1.81456300	1.51593600
C	-1.53673400	-2.68908900	2.01379900
C	-2.47093200	-3.26070500	1.10344600
C	-2.35935600	-2.92623200	-0.24583800
C	-1.32261300	-2.05932100	-0.64697200
C	0.39823400	-1.19841200	2.41629600
N	1.30401100	-0.34051700	1.87808500
C	2.18978500	0.22938800	2.68528600
C	2.24758600	0.00661600	4.07756300
C	1.31576400	-0.85773000	4.65563900
C	0.35877800	-1.48039600	3.80364200
B	1.21742100	-0.61137700	-2.42291200
O	2.30159400	-0.82166400	-3.27236800
C	1.84676300	-0.76313900	-4.64366600
C	0.32186800	-1.13160300	-4.50605600
O	0.02381600	-0.68966300	-3.15791500
B	2.41732900	-2.03963400	-0.38160200
O	3.76583300	-2.10552400	-0.04646900
C	4.15352100	-3.49309000	0.09077300
C	3.04936100	-4.23432300	-0.75125100
O	1.92928700	-3.31612700	-0.66282600
B	1.95581300	1.65431600	-0.72403000
O	2.32094200	2.23023200	-1.94824400
C	3.34658700	3.21472100	-1.70196600
C	3.04622300	3.64502000	-0.21541500
O	2.43883700	2.44662300	0.33384100
H	-1.19803300	-1.78647300	-1.68993400
H	2.87351100	0.92322300	2.20704700
C	1.29764400	-1.13755000	6.13839200
H	0.33788300	-0.84966400	6.58225300
H	1.44049300	-2.20496700	6.34225300
H	2.08060500	-0.59323300	6.66553900
C	3.30797000	0.73016300	4.87426300
H	2.86814000	1.39156300	5.62880500
H	3.97150400	0.03219900	5.39651400
H	3.92745400	1.34511100	4.21722800
C	-3.29561800	-3.45462000	-1.30756300
H	-4.33510300	-3.17005800	-1.11074000
H	-3.02528800	-3.05780000	-2.28881900
H	-3.26167000	-4.54775400	-1.37234300
C	-3.54380800	-4.19299300	1.60981200
H	-4.19298400	-4.54167200	0.80714400
H	-3.10573600	-5.07572600	2.08964400
H	-4.17550200	-3.69975200	2.35720100
C	-1.55386600	-2.95340500	3.42525800
H	-2.30513600	-3.62546900	3.82409500
C	-0.65173200	-2.38382700	4.27544600
H	-0.70162400	-2.61510700	5.33344700

C	-0.22215900	1.52713500	-0.69325400
H	2.76284100	0.14618500	-0.66882600
H	0.06747800	2.37279200	-1.32608100
C	3.40763200	-4.36141700	-2.23897700
H	4.23400600	-5.06062700	-2.40210700
H	2.53366500	-4.73561700	-2.78090200
H	3.67377600	-3.38867200	-2.65984100
C	2.61186900	-5.59203800	-0.20215100
H	1.81825500	-6.00337000	-0.83328700
H	3.44455500	-6.30428600	-0.20179900
H	2.22382200	-5.51065200	0.81507000
C	5.58379500	-3.65098900	-0.42547100
H	6.26642900	-3.07448900	0.20617000
H	5.90133600	-4.69936600	-0.39713500
H	5.68272400	-3.28243700	-1.44790000
C	4.10109700	-3.82667900	1.58977000
H	4.44662600	-4.84493000	1.79574300
H	4.74860200	-3.12872900	2.12825900
H	3.08581300	-3.71648500	1.98109200
C	0.05796100	-2.64409800	-4.55523000
H	0.22802100	-3.05624400	-5.55484400
H	0.69384400	-3.17495800	-3.84186200
H	-0.98619700	-2.83391900	-4.28644500
C	-0.61466900	-0.40503700	-5.47076800
H	-1.64998000	-0.70010900	-5.27279800
H	-0.54588900	0.67772400	-5.35372200
H	-0.38491600	-0.66066400	-6.51102500
C	2.67554900	-1.74010600	-5.47829200
H	2.32990500	-1.76154300	-6.51789000
H	3.72213300	-1.42139100	-5.47841400
H	2.63377600	-2.75399300	-5.07629900
C	2.08284100	0.67206000	-5.13800600
H	1.54167900	1.39457200	-4.52315500
H	3.14883400	0.89983400	-5.04848100
H	1.79134400	0.79599800	-6.18619000
C	2.02414700	4.78589200	-0.11547800
H	1.72763400	4.91798300	0.92906000
H	2.44282700	5.73328700	-0.46902400
H	1.12656500	4.56786300	-0.69907500
C	4.27891400	3.98504800	0.62287900
H	3.97273700	4.24053700	1.64222900
H	4.97165000	3.14327300	0.67684000
H	4.81038000	4.84763600	0.20665100
C	4.70586900	2.51475600	-1.86630800
H	4.83366900	1.71344300	-1.13329100
H	4.74839600	2.06242900	-2.86056900
H	5.54141200	3.21538100	-1.76762600
C	3.21144600	4.32853100	-2.74086200
H	3.92690900	5.13607600	-2.55036200
H	3.41714400	3.92468500	-3.73648200
H	2.20408400	4.74911000	-2.75125700
H	-0.85219400	0.93471600	-1.36709500
Si	-1.33328800	2.25359000	0.64214200
C	-2.77961900	1.11651100	1.10348100
H	-2.44659500	0.18303800	1.56658700
H	-3.44557200	1.62046900	1.81227900
H	-3.36899300	0.85780400	0.21662200
C	-0.37638500	2.62859900	2.26256600
H	-0.14423600	1.71686400	2.81211500
H	0.56258000	3.14854900	2.07311300
C	-2.07137100	3.88667300	0.01605000
H	-1.29225100	4.62763400	-0.19372800
H	-2.62738000	3.72207200	-0.91421400
H	-2.75656100	4.32514100	0.74830700
Cl	-1.33093700	3.66979600	3.43604500

TS2d

Ir	1.70693800	-0.18418000	-0.19794400
N	-0.19118900	-1.38849000	0.05850500

C	-0.60404800	-1.53654700	1.34362900
C	-1.74703200	-2.30481600	1.67673500
C	-2.46427300	-2.96031100	0.63525200
C	-2.00643900	-2.81223100	-0.67461300
C	-0.86607100	-2.01289100	-0.89568400
C	0.16431500	-0.88659200	2.37732400
N	1.27376500	-0.19490900	2.00220600
C	1.96881300	0.43469500	2.94083200
C	1.64172400	0.41908900	4.31336200
C	0.52347600	-0.30460300	4.72575000
C	-0.24161600	-0.97832800	3.73110500
B	3.76196400	0.02007900	0.01363000
O	4.72776400	0.45406600	-0.88891700
C	6.05163300	0.21487700	-0.35200200
C	5.77111500	0.06307200	1.19365200
O	4.37866400	-0.32926900	1.22575500
B	2.54549700	-2.12787100	-0.24276700
O	3.55565700	-2.68973000	-1.01674200
C	3.71563900	-4.09283800	-0.67520000
C	2.34738400	-4.42870200	0.02501300
O	1.93012200	-3.13307800	0.51283500
B	2.16110500	1.85413300	-0.66472000
O	2.12473000	2.43119500	-1.94375800
C	2.95282900	3.61690100	-1.97432000
C	3.06532800	3.99117200	-0.44382600
O	2.84987800	2.71786900	0.21437100
H	-0.49047900	-1.87991400	-1.90448600
H	2.84180300	0.97083700	2.59184200
C	0.11328000	-0.38413300	6.17593500
H	-0.89450200	0.01985100	6.32591200
H	0.09950600	-1.42234500	6.52662200
H	0.79117800	0.17311300	6.82188600
C	2.52892500	1.18626300	5.26586500
H	1.97366600	1.96094700	5.80623500
H	2.98623800	0.52948400	6.01422700
H	3.33770700	1.67927900	4.72160900
C	-2.67675700	-3.45387200	-1.86685300
H	-3.71612600	-3.12541500	-1.97307200
H	-2.15588200	-3.19011300	-2.79029900
H	-2.68291400	-4.54672600	-1.79071500
C	-3.68335000	-3.78649900	0.96360500
H	-4.46348200	-3.17090800	1.42601100
H	-4.11218100	-4.25081800	0.07612900
H	-3.44049200	-4.58542100	1.67301600
C	-2.12524200	-2.38572100	3.05878600
H	-3.00071000	-2.96301200	3.33338900
C	-1.41287800	-1.75118800	4.03323200
H	-1.73682700	-1.83539300	5.06442900
C	0.18889200	1.62232100	-0.22565200
H	1.89576600	-0.27747700	-1.75902800
H	-0.34237500	1.20804200	0.63612600
C	1.27100000	-4.92258400	-0.95396900
H	1.48871900	-5.93041200	-1.32146200
H	0.30615100	-4.94618500	-0.43940300
H	1.18055700	-4.25394200	-1.81445500
C	2.43114600	-5.38681500	1.21458200
H	1.43211500	-5.52966100	1.63779700
H	2.81082500	-6.36777800	0.90849100
H	3.07504800	-4.99250600	2.00251800
C	4.00084100	-4.86357100	-1.96495500
H	4.94876000	-4.52178000	-2.39024000
H	4.08503100	-5.93874200	-1.77134700
H	3.22347000	-4.70196700	-2.71353800
C	4.90902800	-4.22762700	0.28025800
H	5.06226800	-5.26885600	0.58027100
H	5.81990700	-3.88841900	-0.21835800
H	4.76171800	-3.62169200	1.17739100
C	5.87792600	1.38281600	1.97360200
H	6.90592000	1.75657400	2.01453200

H	5.23554200	2.14799700	1.53295200
H	5.54391400	1.20917900	3.00202500
C	6.60174200	-1.01148700	1.89598700
H	6.29706300	-1.08366100	2.94477200
H	6.46745800	-1.99113900	1.43687100
H	7.66775700	-0.75933200	1.87233300
C	6.97981500	1.37689100	-0.71364300
H	7.98348400	1.19950300	-0.31125100
H	7.06559800	1.45592700	-1.80099000
H	6.62464200	2.33329200	-0.33019700
C	6.57866900	-1.05645800	-1.03047300
H	5.89397100	-1.88765300	-0.87367400
H	6.63209300	-0.87696900	-2.10824300
H	7.57968600	-1.32572300	-0.67776600
C	1.97071600	4.95775400	0.04028400
H	1.99619600	4.99652200	1.13353600
H	2.13043500	5.97119700	-0.34125000
H	0.97177300	4.63705800	-0.26336400
C	4.42460400	4.54822900	-0.02161500
H	4.43143300	4.73189500	1.05748300
H	5.23279400	3.85678800	-0.25706200
H	4.62791800	5.49955000	-0.52546000
C	4.28527100	3.22580300	-2.62777300
H	4.75797700	2.41040200	-2.08318900
H	4.08064500	2.87113400	-3.64249500
H	4.97040900	4.07738400	-2.69701900
C	2.27939400	4.68331800	-2.84214300
H	2.90649600	5.57980200	-2.90362700
H	2.14996300	4.29700700	-3.85737700
H	1.30011900	4.97869300	-2.46563300
H	0.43004600	2.63232000	0.11818500
Si	-1.04168000	1.79453000	-1.65806400
C	-2.71391300	1.02405300	-1.18721300
H	-3.15495300	1.58115900	-0.35183600
H	-3.40941300	1.08556700	-2.03072900
H	-2.64454200	-0.02329000	-0.88336700
C	-1.43139500	3.60513400	-2.06051200
H	-1.75077100	4.14809000	-1.16321800
H	-0.57006200	4.12651500	-2.48405900
H	-2.24444000	3.66266000	-2.79200900
C	-0.39178700	0.98129300	-3.26459400
H	-0.12020400	-0.06666800	-3.13957000
H	0.49467300	1.50491700	-3.62201100
Cl	-1.62866500	1.03233900	-4.62169800

1e

Si	1.49924400	0.89288800	3.54291400
C	-0.38323300	0.73488100	3.58466200
H	-0.87940000	1.70807000	3.49807300
H	-0.74385500	0.10377200	2.76532500
H	-0.71738900	0.28279300	4.52575900
C	2.07328700	1.97759900	4.98078800
H	1.79706000	1.52811800	5.94171400
H	3.16058500	2.10764600	4.97482900
H	1.62005300	2.97455100	4.94725500
C	2.30300400	-0.81317000	3.59933800
H	1.98125400	-1.31592200	4.52240400
H	1.88157700	-1.40683500	2.77709700
C	3.83919100	-0.81274400	3.50574600
H	4.17542400	-0.33093600	2.58265400
H	4.29242900	-0.27065700	4.34315100
H	4.24501100	-1.83065400	3.51893800
O	2.00006300	1.55052800	2.08207900
C	1.67172700	2.85051800	1.63022100
H	2.13195900	3.00154700	0.64853300
H	0.58638000	2.98761400	1.52134300
H	2.05048900	3.62933500	2.30717100

2e

B	2.67716600	1.46541500	0.18608300
O	2.25053100	1.84128800	-1.06941900
C	2.75552900	3.18077500	-1.32944100
C	3.03134800	3.72472200	0.12705000
O	3.27519700	2.49911100	0.87249300
C	2.44297400	0.04509000	0.78755200
C	1.80735300	4.39345600	0.76778100
H	2.02183900	4.58464500	1.82303200
H	1.57073200	5.34827200	0.28852200
H	0.92908700	3.74398500	0.71758600
C	4.25676800	4.62619600	0.26405200
H	4.38315400	4.91392700	1.31170100
H	5.16923100	4.12130200	-0.05795300
H	4.13626800	5.54134900	-0.32538400
C	4.02975000	3.01467000	-2.16854000
H	4.79671800	2.46194900	-1.61865500
H	3.78794500	2.44773500	-3.07181500
H	4.44671600	3.98068400	-2.46908000
C	1.70086800	3.94980900	-2.12285800
H	2.01928000	4.98307600	-2.29707700
H	1.55537800	3.47309500	-3.09666800
H	0.73924100	3.96368500	-1.60707400
H	2.31166100	-0.69708700	-0.00762600
Si	0.92812700	0.04115200	1.93432800
C	0.29670200	-1.72610400	2.16051500
H	1.07796800	-2.36371600	2.59011200
H	-0.56813600	-1.75787000	2.83170200
H	-0.00341600	-2.17726800	1.20834100
C	1.36982400	0.84129900	3.58300700
H	2.13500600	0.21908500	4.06885800
H	1.86457900	1.79547100	3.36145600
C	0.18572400	1.06561900	4.53951300
H	-0.29288700	0.11984000	4.81779300
H	0.50385200	1.55387400	5.46782600
H	-0.57975600	1.69481200	4.07546600
H	3.30221300	-0.26053900	1.39806600
O	-0.28621100	1.01141700	1.29493200
C	-0.92134000	0.77430400	0.04754000
H	-1.73169500	1.50168500	-0.06723900
H	-0.22199000	0.89925100	-0.78845300
H	-1.36160900	-0.23138300	-0.00496900

TS1e

Ir	1.01647000	0.00202700	-0.01560000
N	-0.17138500	-1.79268400	0.79692900
C	-0.45835500	-1.73258000	2.12244700
C	-1.18467200	-2.75480300	2.78364700
C	-1.63106800	-3.87683900	2.02876300
C	-1.32931700	-3.91357600	0.66771800
C	-0.59547800	-2.84468800	0.10884800
C	0.00771600	-0.58051300	2.85359000
N	0.69782700	0.37452200	2.16838700
C	1.13803400	1.43740300	2.83695700
C	0.93243600	1.64152200	4.21735000
C	0.22671000	0.68077500	4.94051200
C	-0.25188200	-0.46427200	4.24218000
B	2.86601800	-0.84212600	0.56431300
O	3.98299100	-0.13266900	0.99374800
C	5.08372000	-1.05389400	1.19536300
C	4.33488600	-2.42106600	1.41442600
O	3.09986800	-2.21514900	0.68611700
B	1.72094300	-0.91441000	-1.73414400
O	2.97025800	-0.80234800	-2.33399500
C	2.94707500	-1.47717800	-3.61308000
C	1.79318300	-2.52340100	-3.41116900
O	0.94365700	-1.84204700	-2.45238100
B	2.02736000	1.69426400	-0.53615400
O	2.36573200	2.11698900	-1.82269000
C	3.23212000	3.26899200	-1.73068900

C	2.82371400	3.87463600	-0.33978800
O	2.39837600	2.69463100	0.38105500
H	-0.33512300	-2.84209000	-0.94525800
H	1.68580100	2.16153900	2.24173200
C	-0.03417300	0.82658700	6.41982500
H	-1.10926800	0.85130200	6.63211200
H	0.38480500	-0.01636600	6.98101300
H	0.40248600	1.74041900	6.82186100
C	1.49341500	2.89931400	4.83998400
H	0.70729200	3.52091500	5.28296500
H	2.21642800	2.67368400	5.63173100
H	2.00717500	3.50124100	4.08701000
C	-1.74978300	-5.04367600	-0.24277600
H	-2.83919900	-5.15433200	-0.27827800
H	-1.40508000	-4.86064700	-1.26319700
H	-1.33331000	-6.00428500	0.08031300
C	-2.40801000	-4.98036000	2.70504300
H	-2.66456700	-5.78113300	2.01197200
H	-1.83336400	-5.42374000	3.52611000
H	-3.34288300	-4.60169900	3.13414000
C	-1.43500300	-2.60373900	4.18831100
H	-1.99057700	-3.37384400	4.71152500
C	-0.99062800	-1.51620200	4.88086900
H	-1.20099900	-1.44196600	5.94174900
C	-1.04479200	1.07261600	-0.37552000
H	0.16628900	0.28897200	-1.34083300
H	-0.80912700	2.03615800	0.08783500
C	2.97866900	4.17383500	-2.93607400
H	3.56553900	5.09671500	-2.86720400
H	3.27567500	3.65423500	-3.85204600
H	1.92313400	4.43632100	-3.02863500
C	1.61571300	4.82024000	-0.42440400
H	1.25893800	5.03602500	0.58744300
H	1.87276500	5.76967800	-0.90508400
H	0.79656600	4.35841600	-0.98186600
C	3.95610800	4.54340400	0.43934900
H	4.36002900	5.40008200	-0.11142700
H	3.57848700	4.91178100	1.39890200
H	4.76796500	3.84335900	0.64306400
C	4.67780800	2.74881900	-1.75616300
H	4.81884400	2.15377800	-2.66241300
H	5.40803400	3.56506200	-1.75879300
H	4.87038300	2.09930100	-0.89846500
C	5.93283000	-1.02524800	-0.08350100
H	6.29044700	-0.00485600	-0.24851700
H	6.80378700	-1.68458200	-0.00968600
H	5.33485100	-1.31062000	-0.95192900
C	5.91294700	-0.56763000	2.38410900
H	6.72826500	-1.26463800	2.60800200
H	6.35621300	0.40400800	2.14673100
H	5.30176000	-0.44632100	3.28061500
C	3.95015400	-2.67398100	2.88048700
H	3.27118000	-3.53054000	2.92550300
H	4.82395200	-2.89424000	3.50220100
H	3.43123800	-1.80883500	3.30276700
C	5.04046700	-3.65190600	0.84588800
H	6.00363000	-3.81962000	1.34073900
H	4.41989000	-4.53858800	1.00825400
H	5.21478000	-3.55437800	-0.22712200
C	2.62639400	-0.41726800	-4.67792300
H	3.36174200	0.38781500	-4.60306900
H	2.66498400	-0.83224800	-5.69045300
H	1.63860900	0.02106100	-4.51521100
C	0.96868800	-2.83686000	-4.65881300
H	1.59231700	-3.29283700	-5.43569400
H	0.17401900	-3.54644400	-4.40815000
H	0.49997600	-1.94074500	-5.06927400
C	2.26930100	-3.82590500	-2.75052000
H	1.39716100	-4.43119500	-2.48400600

H	2.89610800	-4.41824600	-3.42476200
H	2.82717700	-3.61817900	-1.83363400
C	4.32808300	-2.07594200	-3.87868900
H	5.06513800	-1.27207100	-3.96475700
H	4.64396500	-2.73745100	-3.06973400
H	4.33710800	-2.64317800	-4.81601200
H	-1.66744500	0.49271600	0.31322900
Si	-1.96929100	1.32882700	-1.98690600
C	-0.97270700	2.42719300	-3.16130800
H	-0.98274800	3.47214800	-2.82779700
H	0.07673000	2.11902100	-3.20655900
H	-1.39225300	2.40480900	-4.17320300
C	-2.42161200	-0.30199200	-2.82966900
H	-2.82739000	-0.05891300	-3.82132400
H	-1.49762100	-0.86784500	-3.00581400
C	-3.43298000	-1.17164100	-2.06193600
H	-3.03577700	-1.48067900	-1.08832700
H	-4.36257600	-0.62470000	-1.87681700
H	-3.68565300	-2.08479800	-2.61533800
O	-3.47531700	2.04649000	-1.70821800
C	-3.65563200	3.29155300	-1.06956800
H	-3.17213400	4.11436700	-1.61732100
H	-4.72900600	3.50701800	-1.02092000
H	-3.26321000	3.29095200	-0.04193200

INT2e

Ir	0.94233800	0.01786800	-0.10103800
N	-0.28968000	-1.81017300	0.62546100
C	-0.53138200	-1.79582500	1.96061400
C	-1.31837800	-2.78757800	2.59883100
C	-1.87701400	-3.83186900	1.80901600
C	-1.61117300	-3.83016800	0.44034100
C	-0.80952700	-2.79709900	-0.09211800
C	0.05211800	-0.72686600	2.72812300
N	0.79872000	0.20338200	2.06570200
C	1.34936100	1.19041400	2.77072600
C	1.20521500	1.33785300	4.16464500
C	0.44388400	0.40342000	4.86570600
C	-0.15102700	-0.65930200	4.12926200
B	2.83288000	-0.82791000	0.45710600
O	3.91213500	-0.03951200	0.83561200
C	5.01595300	-0.89300800	1.22879000
C	4.28249800	-2.24114800	1.58440000
O	3.07973800	-2.16248700	0.77597300
B	1.87812100	-1.04383900	-1.64803000
O	3.07299200	-0.78770200	-2.31129200
C	3.09950500	-1.56498700	-3.53320400
C	2.14306300	-2.76517700	-3.18261900
O	1.24401000	-2.15536400	-2.22386000
B	1.90701300	1.75601800	-0.56042300
O	2.27798400	2.17264000	-1.83562300
C	3.13164000	3.33360600	-1.72058300
C	2.68589100	3.93466900	-0.33745800
O	2.23744800	2.75105200	0.36892400
H	-0.56148200	-2.78293100	-1.14675800
H	1.92190800	1.90893800	2.19554700
C	0.24110900	0.49662400	6.35813600
H	-0.82136600	0.59763700	6.60767100
H	0.60655400	-0.40367100	6.86507700
H	0.76293400	1.35178500	6.78679000
C	1.88547400	2.51507700	4.82483600
H	1.16472100	3.17502000	5.32021300
H	2.60994600	2.19571500	5.58235400
H	2.42320600	3.11030500	4.08348500
C	-2.13942600	-4.88292200	-0.50621000
H	-3.23468500	-4.91124300	-0.51431500
H	-1.80876600	-4.67969000	-1.52743400
H	-1.78867700	-5.88567500	-0.23754700
C	-2.72474600	-4.89899100	2.45792000

H	-3.08022400	-5.63194900	1.73418000
H	-2.16182200	-5.43951700	3.22755100
H	-3.60338100	-4.46387600	2.94785800
C	-1.51165200	-2.68751400	4.01666500
H	-2.11284000	-3.43501800	4.52191500
C	-0.95584400	-1.67667600	4.74343900
H	-1.12503700	-1.63772900	5.81341000
C	-0.96339100	1.16815800	-0.01668200
H	0.70258100	0.09498000	-1.67987700
H	-0.73984900	2.16496000	0.38604300
C	2.89526100	4.23810900	-2.92939300
H	3.47352600	5.16523100	-2.84689200
H	3.21514100	3.72205000	-3.83963000
H	1.83987100	4.49223700	-3.04218600
C	1.48305800	4.88284400	-0.44499100
H	1.10711400	5.09701500	0.55997500
H	1.75419200	5.83197500	-0.91848400
H	0.67246000	4.42576100	-1.01767000
C	3.79996400	4.59362700	0.47541200
H	4.21750400	5.45534200	-0.05682200
H	3.39846300	4.95325900	1.42846600
H	4.60725300	3.89140100	0.69042700
C	4.58089700	2.82303300	-1.71710200
H	4.74452700	2.23481300	-2.62401200
H	5.30533900	3.64416000	-1.69882600
H	4.75810200	2.16752400	-0.86062600
C	5.94965200	-1.00560700	0.01617500
H	6.29448100	-0.00496200	-0.25904200
H	6.82718300	-1.62367400	0.23182900
H	5.42043000	-1.41865800	-0.84492000
C	5.75253700	-0.22809300	2.39198000
H	6.56720600	-0.86336500	2.75691000
H	6.18821100	0.71709900	2.05506500
H	5.08092000	-0.01007800	3.22451000
C	3.82825200	-2.32056600	3.04979000
H	3.16657600	-3.18339500	3.16852600
H	4.67544100	-2.44074200	3.73257300
H	3.27172500	-1.42559200	3.34091200
C	5.04214800	-3.51453700	1.21356100
H	5.97808700	-3.58993700	1.77794200
H	4.43133300	-4.38987500	1.45406600
H	5.27726700	-3.55015900	0.14840900
C	2.55459300	-0.66163700	-4.64925200
H	3.15491300	0.25084200	-4.68792300
H	2.59920900	-1.15076200	-5.62755900
H	1.52023900	-0.36875100	-4.45092700
C	1.31660000	-3.29662800	-4.35356100
H	1.96513300	-3.70011600	-5.13888200
H	0.66634200	-4.10713600	-4.00959000
H	0.68482400	-2.52000600	-4.78820000
C	2.85195400	-3.92481100	-2.46915500
H	2.09893100	-4.63217700	-2.10916500
H	3.53099400	-4.46081200	-3.13982800
H	3.40936100	-3.56530200	-1.60160900
C	4.54474800	-1.95394600	-3.83875000
H	5.13895000	-1.05096700	-4.00654500
H	4.99898900	-2.50917800	-3.01600200
H	4.60081400	-2.56826800	-4.74409200
H	-1.58452600	0.66107000	0.73591800
Si	-1.93448800	1.35377900	-1.59618500
C	-1.09709700	2.46223800	-2.87252400
H	-1.13063900	3.50300600	-2.53063700
H	-0.04819500	2.19441600	-3.02708300
H	-1.61831500	2.42298300	-3.83507600
C	-2.36025300	-0.34287500	-2.35712300
H	-1.42111800	-0.85410800	-2.60559400
H	-2.83011800	-0.96096600	-1.57802600
C	-3.27582600	-0.29117600	-3.59237100
H	-2.80623100	0.26103300	-4.41409700

H	-3.51621100	-1.29288700	-3.97078100
H	-4.22131200	0.21444600	-3.36889000
C	-4.38110100	1.73048900	-0.42071800
H	-3.98866900	1.66873400	0.60572800
H	-5.21073400	2.44752000	-0.42081700
H	-4.79319400	0.74302400	-0.68335500
O	-3.40646900	2.16614600	-1.33961600

INT2e-ISO

Ir	1.26151200	-0.24804500	-0.36900000
N	-0.39160600	-1.52092300	0.22775700
C	-0.49750600	-1.80483100	1.55541500
C	-1.48348100	-2.68917400	2.05230400
C	-2.41264000	-3.26877900	1.14394600
C	-2.30719800	-2.93014400	-0.20408600
C	-1.27521500	-2.05934300	-0.60550100
C	0.42538700	-1.16845500	2.45700800
N	1.31570000	-0.29405400	1.92122500
C	2.18202600	0.30037800	2.73290500
C	2.23634100	0.08258300	4.12575500
C	1.32326500	-0.80304000	4.70040100
C	0.38538300	-1.44753800	3.84519100
B	1.17567900	-0.52579900	-2.38898500
O	2.26099300	-0.70001600	-3.24912200
C	1.79405400	-0.65440000	-4.61567600
C	0.28221700	-1.06883500	-4.46553700
O	-0.01800700	-0.63553600	-3.11652400
B	2.41109100	-2.02918200	-0.36445500
O	3.75156600	-2.13674500	-0.00670300
C	4.10348400	-3.53659300	0.10683600
C	2.99649600	-4.23106700	-0.77025500
O	1.89951600	-3.28452100	-0.68703800
B	2.29315100	1.53915000	-0.69080400
O	2.43717800	2.20965800	-1.89696100
C	3.44158500	3.23918300	-1.73957400
C	3.40124100	3.51133300	-0.18771800
O	2.94109400	2.23671500	0.33211900
H	-1.15779000	-1.78323700	-1.64770300
H	2.86120300	0.99936900	2.25468000
C	1.30776700	-1.08388100	6.18349100
H	0.33783800	-0.82748300	6.62478000
H	1.48303700	-2.14638100	6.38820900
H	2.07166100	-0.51592300	6.71411900
C	3.27904200	0.82920100	4.92540200
H	2.82540400	1.47773800	5.68328400
H	3.96006100	0.14634300	5.44547400
H	3.88352300	1.46057200	4.26978400
C	-3.24408100	-3.45942500	-1.26503000
H	-4.28440900	-3.18117800	-1.06338800
H	-2.98008500	-3.05526600	-2.24508400
H	-3.20499300	-4.55216600	-1.33666400
C	-3.47726900	-4.21148900	1.64982100
H	-4.12248100	-4.56606700	0.84636000
H	-3.03240200	-5.09062700	2.13019600
H	-4.11491100	-3.72472000	2.39654500
C	-1.49901800	-2.95404300	3.46362000
H	-2.23889400	-3.64007400	3.86063900
C	-0.60858000	-2.36976200	4.31596100
H	-0.65513100	-2.60344200	5.37374500
C	-0.38348200	1.33504600	-0.56799900
H	2.81264800	0.01154300	-0.63726000
C	3.38013300	-4.34156600	-2.25297000
H	4.19144000	-5.05888600	-2.41315500
H	2.50773000	-4.68331300	-2.81811300
H	3.67976300	-3.36912000	-2.65182200
C	2.51134300	-5.58559900	-0.25453200
H	1.71918000	-5.96355700	-0.90791800
H	3.32442400	-6.32014500	-0.25136200
H	2.10570900	-5.51124700	0.75626300

C	5.53859400	-3.71749900	-0.38807000
H	6.22290000	-3.17075800	0.26774600
H	5.83108100	-4.77343900	-0.37662700
H	5.66481600	-3.33031800	-1.40064100
C	4.01635100	-3.89989400	1.59715100
H	4.33062100	-4.93136900	1.78678300
H	4.67283700	-3.23117100	2.16122400
H	2.99804000	-3.76971900	1.97392700
C	0.06348300	-2.58870200	-4.51325100
H	0.24027200	-2.99612400	-5.51372800
H	0.71819900	-3.09968100	-3.80250600
H	-0.97294500	-2.80940300	-4.23850900
C	-0.68383800	-0.37145600	-5.42303700
H	-1.70795400	-0.69722000	-5.21563800
H	-0.64740300	0.71299800	-5.30696900
H	-0.45531500	-0.62105400	-6.46512800
C	2.64590100	-1.60578800	-5.45681000
H	2.29338800	-1.63882000	-6.49382900
H	3.68245600	-1.25565500	-5.46480300
H	2.63760100	-2.62001000	-5.05355300
C	1.98128300	0.78693500	-5.11305900
H	1.41428800	1.49120900	-4.50030100
H	3.03886700	1.05105000	-5.02422600
H	1.68496000	0.89930900	-6.16123800
C	2.37241700	4.57630700	0.21238900
H	2.26154200	4.57597700	1.30005300
H	2.68526200	5.57709300	-0.10170000
H	1.39442200	4.36309400	-0.22577900
C	4.75264400	3.83016400	0.45211200
H	4.62438200	3.97589300	1.52928000
H	5.46919700	3.02036200	0.30403400
H	5.17536000	4.75199200	0.03782600
C	4.77512600	2.64389300	-2.21679600
H	5.06867900	1.78528100	-1.60661000
H	4.65556600	2.29438000	-3.24558900
H	5.58267600	3.38268100	-2.19339700
C	3.06025100	4.42984600	-2.61874700
H	3.76057200	5.26107400	-2.48161300
H	3.09267900	4.13171800	-3.67069700
H	2.05065500	4.78346900	-2.40168300
H	-0.03167400	2.21247400	-1.12511500
Si	-1.41587600	1.97634500	0.83468800
C	-0.49036400	3.01061500	2.12821200
H	-0.16512900	3.96414500	1.69917700
H	-1.13387200	3.24737700	2.98403700
H	0.39862400	2.49559800	2.50227200
C	-2.84271000	3.08266900	0.20147200
H	-3.40906100	3.46801700	1.06229300
H	-2.39598800	3.97098800	-0.26808500
C	-3.80272100	2.40241300	-0.78915300
H	-4.27281300	1.51740600	-0.34501600
H	-4.60673200	3.07402400	-1.11477100
H	-3.27117400	2.06623400	-1.68573300
H	-1.05547600	0.82479200	-1.26858400
O	-2.13036600	0.65116700	1.62230900
C	-3.08529000	0.79321700	2.65006400
H	-3.35735300	-0.20418800	3.01494800
H	-2.69874500	1.36739300	3.50560100
H	-4.00388800	1.28768700	2.30078000

TSe-ISO

Ir	0.50656600	-0.46201800	-0.36604100
N	-1.00069600	-1.98442600	0.15110100
C	-1.11605000	-2.34329000	1.45865800
C	-2.07801300	-3.28074400	1.90658200
C	-2.98841600	-3.83906700	0.97006300
C	-2.88499900	-3.42456900	-0.35674300
C	-1.87733900	-2.50588300	-0.70707300
C	-0.22313600	-1.72241000	2.39026800

N	0.62277600	-0.78501000	1.89775800
C	1.49906900	-0.24519500	2.73908700
C	1.57597200	-0.54643400	4.11304100
C	0.68217200	-1.47564700	4.64592700
C	-0.24194000	-2.08815800	3.75853300
B	0.51118900	-0.84910700	-2.40109300
O	1.25459700	-0.15800700	-3.35940800
C	0.83730500	-0.56685300	-4.68010100
C	0.21252000	-1.98260200	-4.40119900
O	-0.23932300	-1.84600200	-3.02801000
B	2.11656400	-1.84328600	-0.44805100
O	3.13737600	-1.95202100	-1.39747400
C	4.15809400	-2.84813300	-0.89421900
C	3.34959700	-3.72696200	0.12838500
O	2.29379200	-2.82490300	0.53315700
B	1.87771500	1.15105300	-0.25326100
O	1.65280900	2.46589600	-0.64895600
C	2.91794900	3.16923600	-0.66350300
C	3.77245300	2.33960100	0.36765300
O	3.14374800	1.02711500	0.30651300
H	-1.76066000	-2.20328800	-1.73795900
H	2.20102300	0.45243300	2.30181500
C	0.68358700	-1.84077300	6.11030000
H	-0.28885500	-1.63206500	6.57059600
H	0.88420200	-2.90895300	6.25256600
H	1.43796500	-1.28667900	6.66851800
C	2.62269700	0.15739300	4.94552400
H	2.17273400	0.75666900	5.74507600
H	3.31161800	-0.55181800	5.41765200
H	3.21806900	0.83216400	4.32645800
C	-3.80116600	-3.92200600	-1.45124400
H	-4.84865100	-3.67265400	-1.24829000
H	-3.53447900	-3.46991900	-2.40941300
H	-3.74169000	-5.00961200	-1.57120000
C	-4.02860700	-4.83493700	1.42083500
H	-4.65547100	-5.17054400	0.59491500
H	-3.56281200	-5.72196000	1.86548000
H	-4.68752000	-4.40155800	2.18191300
C	-2.08645400	-3.62098000	3.30119400
H	-2.80904100	-4.34482900	3.66073800
C	-1.20728000	-3.06255400	4.18039700
H	-1.24085400	-3.35555500	5.22377300
C	-1.24225600	0.99275300	-0.34783300
H	1.06709400	0.56624200	-1.40778700
H	-0.97012700	1.82941500	-1.00418400
C	2.67785600	-4.94854300	-0.51726500
H	3.40522000	-5.71933400	-0.79297800
H	1.97248100	-5.38191600	0.19790600
H	2.11716300	-4.66053100	-1.41049500
C	4.12390100	-4.15956700	1.37385200
H	3.46515600	-4.73113500	2.03511900
H	4.97409900	-4.79890400	1.11063400
H	4.49550500	-3.29847200	1.93250400
C	4.77001200	-3.61141300	-2.06872000
H	5.26920400	-2.90837400	-2.74254800
H	5.51758500	-4.33382200	-1.72186100
H	4.01234500	-4.14734100	-2.64330400
C	5.23190700	-1.97664000	-0.22537800
H	6.06574300	-2.57611000	0.15492300
H	5.62292600	-1.27093400	-0.96373800
H	4.80502000	-1.39481500	0.59492200
C	1.24931000	-3.11426800	-4.41995400
H	1.61396600	-3.31389500	-5.43266900
H	2.09756100	-2.87006000	-3.77605100
H	0.78224000	-4.02776400	-4.03904200
C	-0.98837200	-2.35045400	-5.27089200
H	-1.37061400	-3.33217500	-4.97404000
H	-1.79818200	-1.62550100	-5.16837500
H	-0.70409100	-2.40769900	-6.32716500

C	2.05942000	-0.56681800	-5.59798600
H	1.80384000	-0.94261600	-6.59495300
H	2.43308200	0.45548400	-5.70876600
H	2.86591800	-1.17653300	-5.18718800
C	-0.18952100	0.46569600	-5.16955200
H	-1.07244200	0.48093400	-4.52455300
H	0.26694900	1.45872500	-5.13463000
H	-0.50913200	0.26935800	-6.19807500
C	3.64718900	2.84989800	1.80919500
H	4.13304500	2.13704400	2.48273400
H	4.13962500	3.81867400	1.93686700
H	2.60082200	2.95298100	2.10883500
C	5.24797900	2.17571200	0.00447600
H	5.74767500	1.56142400	0.75934800
H	5.37032900	1.68479600	-0.96202700
H	5.75199300	3.14782300	-0.02757900
C	3.44947500	3.08691900	-2.10274000
H	3.59677500	2.04864100	-2.41172800
H	2.70952500	3.53081600	-2.77426700
H	4.39225400	3.63030400	-2.22033500
C	2.67073900	4.62876500	-0.28331900
H	3.61223500	5.18699500	-0.23929300
H	2.03578900	5.09797100	-1.04017700
H	2.16554700	4.71848300	0.68002100
H	-2.05242900	0.45232400	-0.85932300
Si	-1.92516100	1.73282200	1.20024700
C	-0.67288900	2.81542700	2.13555900
H	-0.14566700	3.45787100	1.42186700
H	-1.14659800	3.46046200	2.88462000
H	0.07844100	2.20424800	2.64665400
C	-3.45530300	2.84160600	0.90196200
H	-3.80190200	3.24691300	1.86420400
H	-3.12553700	3.71555900	0.32186400
C	-4.62545800	2.15230700	0.17982000
H	-4.98175900	1.28190700	0.74267800
H	-5.48032300	2.82495500	0.03616700
H	-4.32075000	1.79161400	-0.80866700
O	-2.42652400	0.46492800	2.21732700
C	-3.04673700	0.68266900	3.46405000
H	-3.13822700	-0.27898100	3.98299800
H	-2.46630200	1.35872100	4.11090600
H	-4.05705500	1.10602300	3.35893900

TS2e-ISO

Ir	1.31416100	-0.31895700	-0.39925500
N	-0.38253800	-1.54555500	0.19176500
C	-0.49746100	-1.82305300	1.52010000
C	-1.50184700	-2.68418200	2.01990800
C	-2.44083300	-3.24874000	1.11064900
C	-2.32539200	-2.91801100	-0.23833200
C	-1.27420800	-2.06939600	-0.64088100
C	0.43879900	-1.20384300	2.42101300
N	1.34907000	-0.35184000	1.88246800
C	2.22924900	0.22565500	2.68884600
C	2.28198700	0.01090200	4.08254400
C	1.34827300	-0.85045300	4.66121800
C	0.39279100	-1.47625900	3.80993800
B	1.26292000	-0.62666200	-2.41638200
O	2.34512700	-0.84318600	-3.26907700
C	1.88872600	-0.78481800	-4.63914000
C	0.36149600	-1.14178600	-4.49827100
O	0.06854500	-0.69493000	-3.15161200
B	2.47122200	-2.06027500	-0.38823700
O	3.82201900	-2.12763000	-0.05839100
C	4.20774100	-3.51483100	0.08058900
C	3.10201100	-4.25578200	-0.75927400
O	1.98332800	-3.33770500	-0.66897800
B	1.97566500	1.63623500	-0.73464500
O	2.28484600	2.22074800	-1.97187400

C	3.29945000	3.22420500	-1.76324100
C	3.04635400	3.64693200	-0.26598700
O	2.49369000	2.43485300	0.30395000
H	-1.14651300	-1.80089500	-1.68423300
H	2.91627500	0.91519100	2.20891000
C	1.32842600	-1.12664300	6.14505500
H	0.36474900	-0.84828700	6.58672600
H	1.48069000	-2.19227800	6.35183000
H	2.10509100	-0.57415400	6.67336700
C	3.34249400	0.73577700	4.87843900
H	2.90436600	1.39779600	5.63369200
H	4.00754500	0.03907900	5.40067300
H	3.96102000	1.35053800	4.22019200
C	-3.27043400	-3.43238100	-1.29921000
H	-4.30523900	-3.13171900	-1.10133800
H	-2.99559900	-3.03808400	-2.28027600
H	-3.25364600	-4.52595500	-1.36593100
C	-3.52393900	-4.16938500	1.61801500
H	-4.17773400	-4.51038600	0.81563700
H	-3.09582200	-5.05752600	2.09706700
H	-4.15013900	-3.67054600	2.36645700
C	-1.52391600	-2.94285600	3.43229900
H	-2.27853200	-3.61130000	3.83160600
C	-0.62169100	-2.37440700	4.28347600
H	-0.67416100	-2.60307600	5.34208100
C	-0.18461700	1.48799900	-0.60845600
H	2.81243800	0.13808200	-0.65882900
C	3.45814500	-4.38367400	-2.24756500
H	4.28329000	-5.08413300	-2.41211300
H	2.58263000	-4.75636700	-2.78806300
H	3.72479300	-3.41117100	-2.66861400
C	2.66486300	-5.61338700	-0.20917100
H	1.86991800	-6.02429700	-0.83893900
H	3.49712300	-6.32624600	-0.20970300
H	2.27805700	-5.53125200	0.80845900
C	5.63757300	-3.67598200	-0.43606100
H	6.32142400	-3.09923500	0.19412400
H	5.95362900	-4.72482500	-0.40603000
H	5.73637000	-3.30939100	-1.45923000
C	4.15548800	-3.84650300	1.58021500
H	4.49983000	-4.86492200	1.78775900
H	4.80376300	-3.14828800	2.11749600
H	3.14036400	-3.73421700	1.97127400
C	0.08711800	-2.65271900	-4.54394700
H	0.25330400	-3.06849300	-5.54278800
H	0.72003500	-3.18608300	-3.82978300
H	-0.95793000	-2.83458300	-4.27327100
C	-0.57146100	-0.41127900	-5.46361700
H	-1.60844200	-0.69816600	-5.26251000
H	-0.49460000	0.67127000	-5.34948200
H	-0.34552700	-0.67140100	-6.50367600
C	2.70928300	-1.76992700	-5.47266300
H	2.36222800	-1.79158800	-6.51185200
H	3.75825800	-1.45895200	-5.47483700
H	2.66069500	-2.78241500	-5.06793200
C	2.13389500	0.64718400	-5.13857100
H	1.59999900	1.37468400	-4.52334200
H	3.20210500	0.86677300	-5.05347300
H	1.84016500	0.77035500	-6.18630400
C	1.99955000	4.76163700	-0.12245500
H	1.74010000	4.87762300	0.93367000
H	2.37968300	5.72137300	-0.48637400
H	1.08514600	4.52437800	-0.67213700
C	4.30202800	4.01926200	0.52331600
H	4.02929300	4.27006100	1.55333800
H	5.01498100	3.19338300	0.55295200
H	4.79759000	4.89259100	0.08538100
C	4.66542800	2.55135100	-1.97767000
H	4.83426700	1.75331100	-1.24948300

H	4.68068400	2.09900600	-2.97270400
H	5.49025100	3.26821600	-1.90937000
C	3.10434800	4.33709800	-2.79375300
H	3.81158200	5.15743500	-2.62819800
H	3.27948300	3.93934100	-3.79766000
H	2.08962800	4.73879800	-2.76469500
H	0.07440100	2.41263800	-1.13794600
Si	-1.24446400	2.02931700	0.82891800
C	-0.31255200	2.64449600	2.36440900
H	0.69897600	2.95097000	2.08591200
H	-0.81898800	3.49222800	2.83959500
H	-0.21688200	1.85032900	3.11221800
C	-2.40678800	3.44144000	0.28214000
H	-3.04244200	3.72735700	1.13314600
H	-1.78461800	4.32619500	0.08152600
C	-3.28402000	3.13174100	-0.94270500
H	-3.93050700	2.26625500	-0.75938500
H	-3.93096600	3.97558600	-1.21178000
H	-2.67163100	2.89345600	-1.81890000
H	-0.81174300	0.96209300	-1.33649100
O	-2.20856300	0.70610300	1.27008500
C	-3.18521400	0.79259900	2.28561100
H	-3.57653800	-0.21234900	2.48068700
H	-2.77600900	1.18188400	3.23027100
H	-4.02973400	1.43424500	1.99408600

TS2e

Ir	1.61498700	-0.15219400	-0.13946300
N	-0.29300500	-1.31980200	0.20018400
C	-0.62738300	-1.51001000	1.50160000
C	-1.74048500	-2.29966700	1.88033600
C	-2.51805900	-2.92315300	0.86246800
C	-2.15599500	-2.70777900	-0.46710300
C	-1.03173600	-1.89840700	-0.73548600
C	0.18538800	-0.86799800	2.50454500
N	1.25521100	-0.14247100	2.08093600
C	1.97415100	0.50368800	2.98998400
C	1.71952800	0.46266600	4.37714500
C	0.65239500	-0.30703000	4.83857000
C	-0.14487400	-0.99042800	3.87660300
B	3.67694900	0.02128400	0.04350700
O	4.64187100	0.42958200	-0.87371700
C	5.96710300	0.19295800	-0.33995600
C	5.69411400	0.06306000	1.21004000
O	4.29835600	-0.30977300	1.25723200
B	2.42313500	-2.10385400	-0.27875700
O	3.50707100	-2.60921500	-0.99392300
C	3.62576100	-4.03681500	-0.75843500
C	2.17807300	-4.41337200	-0.28345200
O	1.72503100	-3.17137500	0.30231200
B	2.10300800	1.85163900	-0.66074600
O	2.05574200	2.38670400	-1.95821900
C	2.87061000	3.57878000	-2.03080500
C	3.00500500	3.99570500	-0.51196800
O	2.79676500	2.74346100	0.18481200
H	-0.74496200	-1.68235200	-1.75708300
H	2.80817700	1.07481700	2.60371800
C	0.32541500	-0.42130000	6.30752500
H	-0.67956900	-0.03914000	6.52098200
H	0.34904100	-1.46596500	6.63774900
H	1.02842000	0.13620900	6.92592100
C	2.62704600	1.25167100	5.29220100
H	2.07288400	1.99687900	5.87367500
H	3.14927300	0.60371800	6.00500900
H	3.38581300	1.78293200	4.71295600
C	-2.91492500	-3.28610200	-1.63871200
H	-3.96718900	-2.98243100	-1.62935800
H	-2.48318300	-2.94025400	-2.58051500
H	-2.88985100	-4.38179300	-1.64216500

C	-3.70275700	-3.77945900	1.23776200
H	-4.17090100	-4.23239300	0.36418700
H	-3.40626600	-4.58928900	1.91345100
H	-4.46796900	-3.19058500	1.75697800
C	-2.03235600	-2.42550300	3.27979000
H	-2.87957900	-3.02675200	3.58990300
C	-1.27761300	-1.79907100	4.22753400
H	-1.53956400	-1.91356500	5.27334300
C	0.10978200	1.64139600	-0.17068400
H	1.74838000	-0.26389000	-1.70189200
H	-0.33760400	1.35958700	0.78767400
C	1.23026000	-4.74412600	-1.44691100
H	1.47769800	-5.70236600	-1.91463000
H	0.20655800	-4.80296600	-1.06622500
H	1.26418900	-3.96406700	-2.21248100
C	2.09355300	-5.50993300	0.77864700
H	1.04687300	-5.66853200	1.05593400
H	2.48959800	-6.45910600	0.40097500
H	2.64144100	-5.23696400	1.68210500
C	4.08371100	-4.69848400	-2.05865000
H	5.08922600	-4.35115700	-2.31320800
H	4.11946600	-5.78867100	-1.95385800
H	3.42439700	-4.44778600	-2.89147500
C	4.67870800	-4.25925500	0.33710500
H	4.80864800	-5.32392600	0.55509500
H	5.64294900	-3.86902100	0.00323700
H	4.40147700	-3.74258200	1.25954800
C	5.82584700	1.39073800	1.97286300
H	6.85953100	1.74972000	2.00332700
H	5.19268300	2.16029600	1.52580500
H	5.49520200	1.23551600	3.00524300
C	6.51171400	-1.01691900	1.92011600
H	6.21609400	-1.06726600	2.97276700
H	6.34989300	-2.00041600	1.47768200
H	7.58259900	-0.78821100	1.88205300
C	6.89457900	1.34910600	-0.72287200
H	7.90030100	1.17838900	-0.32269700
H	6.97513800	1.41155500	-1.81179900
H	6.54043800	2.31093400	-0.35228300
C	6.49367400	-1.08797000	-1.00095200
H	5.81446300	-1.91930100	-0.82376300
H	6.53730200	-0.92717300	-2.08219900
H	7.49924400	-1.34450400	-0.65157100
C	1.92113800	4.98103400	-0.04059400
H	1.96177600	5.05124400	1.05052600
H	2.07926000	5.98204700	-0.45433600
H	0.91621800	4.65682900	-0.32022200
C	4.37164800	4.55983900	-0.12255900
H	4.39192600	4.77112200	0.95130300
H	5.17474500	3.85935600	-0.34898900
H	4.57226600	5.49718500	-0.65311300
C	4.19742100	3.18282900	-2.69298800
H	4.68301600	2.38411100	-2.13447400
H	3.98284500	2.80159900	-3.69604000
H	4.87553800	4.03713900	-2.79169200
C	2.17074900	4.61644900	-2.91316700
H	2.77489800	5.52749200	-2.98971300
H	2.05046200	4.21172000	-3.92260500
H	1.18333700	4.88929200	-2.53981400
H	0.36428500	2.69272700	-0.01154500
Si	-1.23741200	1.61456200	-1.49913000
C	-2.89231500	1.17724100	-0.68899700
H	-3.17698700	1.95143800	0.03383300
H	-3.69147900	1.10524600	-1.43412500
H	-2.84972200	0.22627200	-0.15006900
C	-1.41335200	3.31649000	-2.32842300
H	-1.44285400	4.08438900	-1.54183500
H	-0.49496200	3.50857300	-2.89405000
C	-2.63587200	3.47171400	-3.25045500

H	-3.57476900	3.35881800	-2.69747800
H	-2.65981900	4.45542900	-3.73479200
H	-2.63689400	2.71606700	-4.04359200
O	-1.06613600	0.45409800	-2.71084700
C	-0.16313400	0.57725200	-3.80549400
H	-0.03613100	-0.41283200	-4.25791600
H	-0.57065200	1.25006900	-4.57344600
H	0.81735200	0.94780600	-3.49226700

1f

C	-3.24167700	0.44158700	-1.85432700
C	-3.57785800	1.68164900	-1.28140700
C	-2.61316200	2.47758900	-0.66118400
C	-1.28729900	2.04655900	-0.60083200
C	-0.93032000	0.81854000	-1.16222200
C	-1.89772800	0.02827200	-1.78225100
H	-0.53486300	2.66428700	-0.11852100
H	-1.59940200	-0.92550200	-2.21065500
H	-4.60490300	2.03534800	-1.31629400
H	-2.89638600	3.43194600	-0.22599000
Si	-4.53392100	-0.62703000	-2.70020600
C	-6.10950400	0.33367500	-3.05628100
H	-5.90827700	1.17415000	-3.73006600
H	-6.84702900	-0.31656800	-3.53701200
H	-6.56247900	0.72885300	-2.14219000
C	-3.84671400	-1.42202200	-4.25904600
H	-3.56768400	-0.65264900	-4.98791300
H	-2.95889300	-2.02609800	-4.04950300
H	-4.59301100	-2.07579200	-4.72107100
Cl	-5.05915000	-2.20547200	-1.39892400
H	0.10050600	0.47834300	-1.11726500

2f

B	1.28687900	1.25229100	-1.00839700
O	2.35493300	1.47321400	-0.16958100
C	3.33078900	0.41706500	-0.40115900
C	2.44964500	-0.72271600	-1.05379100
O	1.34533400	0.03079900	-1.63464300
C	0.12198900	2.28031100	-1.21221100
C	1.83744000	-1.68726200	-0.03028000
H	1.10686700	-2.32365100	-0.53729500
H	2.59761700	-2.33239600	0.42030400
H	1.31654800	-1.15077900	0.76686600
C	3.13119600	-1.51061000	-2.17106500
H	2.42796800	-2.23816200	-2.58621600
H	3.46008300	-0.86034200	-2.98329400
H	3.99837300	-2.05917500	-1.78869900
C	4.38467600	0.99640600	-1.35390100
H	3.94295800	1.27807800	-2.31390300
H	4.81336900	1.89588900	-0.90363500
H	5.19461000	0.28502200	-1.54024400
C	3.97501600	0.05572900	0.93567700
H	4.67776200	-0.77578500	0.81832000
H	4.53114600	0.91615600	1.31867300
H	3.22812100	-0.22189200	1.68142200
H	0.54065700	3.23749400	-1.55060500
Si	-0.83671100	2.68478800	0.35700200
Cl	-2.39008200	4.01445700	-0.18890200
H	-0.56725300	1.93377800	-1.98888600
C	0.23247100	3.59972900	1.60043400
H	1.12057900	3.00301000	1.83244100
H	0.56590200	4.55701500	1.18752000
H	-0.30698700	3.80456600	2.52989900
C	-1.63731200	1.16095900	1.11249400
C	-1.67378500	-0.06773800	0.42777200
C	-2.22805400	1.22048700	2.38924000
C	-2.27747600	-1.19222100	0.99593000
H	-1.21938700	-0.15671500	-0.55486600
C	-2.83311800	0.10091700	2.95912000

H	-2.22271300	2.15444100	2.94613000
C	-2.85879800	-1.10918100	2.26151500
H	-2.29517000	-2.13089300	0.44892500
H	-3.28439300	0.17091200	3.94499300
H	-3.33036600	-1.98260600	2.70348200

3

B	1.01739000	1.66084000	-0.57977100
O	1.71471100	1.27303500	-1.69842100
C	2.78261100	0.38451300	-1.26272500
C	2.95422500	0.78688900	0.25504000
O	1.62944200	1.28802400	0.59286300
C	3.93244500	1.94986000	0.46926900
H	3.84290600	2.30284200	1.50020200
H	4.96880900	1.64309600	0.29892000
H	3.70516000	2.78846400	-0.19484700
C	3.28961300	-0.36442400	1.20077400
H	3.34961100	0.01010200	2.22664100
H	2.52762900	-1.14510600	1.17187200
H	4.25731200	-0.80937800	0.94649600
C	2.27015900	-1.04869900	-1.45902800
H	1.39144300	-1.24584900	-0.83860400
H	1.98126400	-1.18082300	-2.50524900
H	3.03875200	-1.78908100	-1.21788600
C	4.00556700	0.62982900	-2.14394800
H	4.85426700	0.02372600	-1.81004400
H	3.77586100	0.34994900	-3.17604700
H	4.30270900	1.67976200	-2.13876300
C	-2.13380200	3.45877900	-1.91008300
C	-2.75973100	3.84274200	-0.72410400
C	-2.19362000	3.54232500	0.53018400
C	-0.97648600	2.84094200	0.54511600
C	-0.32690700	2.43946700	-0.63531600
C	-0.92737100	2.76034500	-1.86425300
H	-0.50790800	2.59137500	1.49360800
H	-0.44054600	2.45722800	-2.78727600
H	-3.69968200	4.38714600	-0.77937000
H	-2.58804800	3.70269800	-2.86658200
Si	-3.04776300	4.04901700	2.12305900
Cl	-2.73299100	6.11611800	2.43161300
C	-2.32895200	3.16019400	3.61448300
H	-2.83333800	3.48681200	4.52921800
H	-2.46149900	2.07631900	3.52203100
H	-1.26055800	3.36342000	3.73272200
C	-4.90878500	3.80383100	2.00789600
H	-5.14389600	2.74289000	1.86558600
H	-5.40221900	4.14334400	2.92393000
H	-5.34098600	4.36015600	1.17071800

TS1f

Ir	1.00972600	0.24681100	0.12233700
N	-0.22288500	-1.54616700	0.85459500
C	-0.38277000	-1.60847700	2.20101600
C	-1.06894800	-2.67509600	2.83139800
C	-1.63166700	-3.70113400	2.02158200
C	-1.46818300	-3.60931200	0.63961000
C	-0.74939900	-2.51409800	0.11541500
C	0.18588000	-0.54463900	2.98660300
N	0.81037800	0.47088300	2.32388400
C	1.37911500	1.43797000	3.04423300
C	1.35387600	1.49013000	4.45216500
C	0.69965400	0.47531000	5.15184900
C	0.10036300	-0.57475500	4.40066200
B	2.82319800	-0.65605100	0.68779600
O	3.96056000	-0.00170300	1.14408800
C	5.02469600	-0.96872700	1.32771100
C	4.22472900	-2.31387200	1.49753100
O	3.00376000	-2.03967800	0.76651000
B	1.68412200	-0.64272700	-1.62939800

O	2.93871800	-0.54778000	-2.21606800
C	2.90502100	-1.20268000	-3.50772100
C	1.71560500	-2.21594300	-3.33216200
O	0.88251700	-1.52711700	-2.36386900
B	2.07458200	1.93332600	-0.32489500
O	2.32907600	2.41892300	-1.60729600
C	3.30206100	3.48284300	-1.52395900
C	3.08558200	4.01485900	-0.05857300
O	2.59071500	2.83383900	0.61861000
H	-0.57545200	-2.42647800	-0.95249900
H	1.89067400	2.20202400	2.46725700
C	0.62124900	0.46692000	6.65889500
H	-0.41984800	0.48373000	7.00115800
H	1.08159500	-0.43647900	7.07500300
H	1.12639600	1.32702500	7.09761500
C	2.03959600	2.65372900	5.13071900
H	1.33696400	3.25457100	5.71909300
H	2.83139100	2.31932300	5.81003900
H	2.49819200	3.31173500	4.38921700
C	-2.02003600	-4.63076300	-0.32763100
H	-3.11173500	-4.70204700	-0.26569700
H	-1.76625700	-4.36118700	-1.35571200
H	-1.61682700	-5.63200400	-0.13919600
C	-2.38426300	-4.84079700	2.66365900
H	-2.73457800	-5.56413000	1.92768000
H	-1.75600400	-5.37800800	3.38297900
H	-3.25963500	-4.47193600	3.21060200
C	-1.15607500	-2.66743000	4.26327600
H	-1.67759100	-3.47494300	4.76449300
C	-0.59386200	-1.67400800	5.00893100
H	-0.67549400	-1.71147800	6.08930100
H	0.34578800	0.57272100	-1.29971800
C	3.01872300	4.49470700	-2.63406000
H	3.69900500	5.35130000	-2.57115500
H	3.16668400	4.01771800	-3.60749600
H	1.99100700	4.86076200	-2.59452900
C	1.99877700	5.09502500	0.03666800
H	1.75409500	5.26635600	1.08927500
H	2.33054200	6.04469700	-0.39509600
H	1.08679100	4.78189400	-0.47815000
C	4.35089600	4.48568300	0.65901400
H	4.80967700	5.33259800	0.13690200
H	4.09957900	4.81453600	1.67262800
H	5.08475400	3.68214100	0.73779000
C	4.68545900	2.84729900	-1.73255600
H	4.69237100	2.32719100	-2.69410100
H	5.48308300	3.59758900	-1.73826300
H	4.89398300	2.10955500	-0.95370100
C	5.88925500	-0.93270200	0.05962800
H	6.28467100	0.07876100	-0.07074000
H	6.73541800	-1.62439500	0.12366000
H	5.29275400	-1.17225900	-0.82352100
C	5.85773900	-0.55055600	2.53946000
H	6.64210800	-1.28585600	2.75061000
H	6.34123800	0.40959700	2.33660500
H	5.24120800	-0.43234100	3.43263700
C	3.81831800	-2.59926800	2.95157400
H	3.10201900	-3.42585300	2.96294000
H	4.67744600	-2.87927700	3.56955100
H	3.33486500	-1.72794500	3.40194900
C	4.89107700	-3.55076700	0.89605200
H	5.84550900	-3.76375300	1.39024900
H	4.24099700	-4.42084300	1.03002700
H	5.07449200	-3.42804800	-0.17281900
C	2.62726900	-0.11613500	-4.55656800
H	3.39710400	0.65556000	-4.47523200
H	2.64596100	-0.51928500	-5.57421600
H	1.65977500	0.36219600	-4.38470700
C	0.88684000	-2.47392700	-4.58943900

H	1.49841800	-2.93136700	-5.37480700
H	0.06899800	-3.16343900	-4.35790900
H	0.44912400	-1.55344500	-4.97980300
C	2.14411200	-3.54540100	-2.69393600
H	1.25055900	-4.12536800	-2.44317500
H	2.75549800	-4.14526800	-3.37552600
H	2.70222000	-3.37200500	-1.77015800
C	4.26769700	-1.84216500	-3.77071400
H	5.03277900	-1.06263500	-3.83120400
H	4.55088300	-2.53224700	-2.97362200
H	4.26746400	-2.38877900	-4.72017200
C	-0.99982300	1.28477500	-0.77950300
H	-1.59783600	0.52207000	-1.29253000
Si	-2.07513500	2.07758600	0.50425300
C	-1.28676300	3.40482900	1.57733400
H	-2.05365600	3.95941200	2.12677500
H	-0.57394000	2.98632900	2.29207900
H	-0.74674700	4.11667700	0.94729100
C	-3.05471400	0.84831800	1.55444000
C	-3.77282000	-0.20291300	0.95376600
C	-3.15044100	0.98577300	2.95035500
C	-4.55214500	-1.07693400	1.71203500
H	-3.73879000	-0.33150600	-0.12521600
C	-3.92768900	0.11362000	3.71664800
H	-2.61876300	1.79051500	3.45136500
C	-4.63198000	-0.91974800	3.09807800
H	-5.10632300	-1.87316200	1.22169000
H	-3.98885800	0.24574600	4.79371900
H	-5.24684000	-1.59277000	3.69017100
Cl	-3.59485500	3.15660300	-0.56695400
H	-0.76723100	2.05834600	-1.52005000

INT2f

Ir	1.02951900	0.00842300	0.06359400
N	-0.18619800	-1.84768200	0.69901100
C	-0.34211700	-1.95703600	2.04206600
C	-1.10769900	-2.99219400	2.63334700
C	-1.74604800	-3.94058600	1.78409700
C	-1.58354900	-3.80017900	0.40694600
C	-0.78985700	-2.73674600	-0.07709000
C	0.29997900	-0.96666600	2.86655300
N	1.00781400	0.02152200	2.24686200
C	1.60374900	0.94349700	3.00079100
C	1.54671700	0.96430100	4.40887000
C	0.82693200	-0.03195100	5.06800300
C	0.18327700	-1.02581700	4.27784500
B	2.94878700	-0.85954200	0.47126600
O	4.04888100	-0.08582600	0.81731000
C	5.16500700	-0.95331200	1.13791600
C	4.44361900	-2.31194000	1.48211300
O	3.20264900	-2.20228700	0.73745900
B	1.91695200	-0.96323100	-1.57192600
O	3.04866300	-0.61706100	-2.30260500
C	3.01604800	-1.33864300	-3.55911200
C	2.14791900	-2.60091800	-3.19536100
O	1.30704800	-2.08739900	-2.13460000
B	1.97620900	1.77544500	-0.32043300
O	2.25655100	2.30177800	-1.57838400
C	3.13493600	3.44000500	-1.42522900
C	2.79887200	3.92218900	0.03376800
O	2.37685100	2.68807600	0.66519100
H	-0.62885600	-2.61004500	-1.14007200
H	2.14426100	1.70958900	2.45609700
C	0.71551700	-0.07342000	6.57226200
H	-0.33018700	-0.00336600	6.89283200
H	1.11252500	-1.01293100	6.97325300
H	1.26056700	0.74387500	7.04374700
C	2.27003000	2.07846300	5.13021900
H	1.58177000	2.69523300	5.71885200

H	3.03161100	1.69329300	5.81724200
H	2.77191700	2.73422200	4.41511400
C	-2.21907400	-4.72947400	-0.60032500
H	-3.31168700	-4.72667200	-0.52112700
H	-1.96372600	-4.42257200	-1.61693300
H	-1.88298200	-5.76434600	-0.46965100
C	-2.57333600	-5.05234300	2.38237300
H	-2.98808700	-5.70702200	1.61648300
H	-1.97475700	-5.67196600	3.05991000
H	-3.41082000	-4.65283200	2.96591200
C	-1.20510600	-3.02651900	4.06400600
H	-1.78396300	-3.81319700	4.53476000
C	-0.59001300	-2.09342500	4.84555100
H	-0.68939900	-2.15515200	5.92316400
C	-0.86410800	1.14154400	0.34442200
H	0.72147700	0.16309500	-1.49792800
H	-0.59792900	2.10746400	0.79794600
C	2.82784500	4.44903000	-2.53118700
H	3.43257800	5.35553800	-2.41651600
H	3.06462000	4.00699700	-3.50353200
H	1.77335700	4.73093700	-2.53996400
C	1.61088600	4.89268100	0.09683500
H	1.30637000	5.01546200	1.14056100
H	1.86762500	5.87869600	-0.30327300
H	0.75602100	4.50211100	-0.46080400
C	3.97961700	4.49249000	0.81973900
H	4.38200800	5.38710300	0.33187900
H	3.65112600	4.78025500	1.82386600
H	4.78234400	3.76038600	0.92340400
C	4.57208000	2.91694200	-1.57063800
H	4.66209200	2.40956300	-2.53477000
H	5.30926700	3.72593900	-1.53204600
H	4.79739900	2.18591600	-0.79020300
C	6.04923200	-1.02864700	-0.11396400
H	6.38570000	-0.02044800	-0.37122000
H	6.93231400	-1.65503200	0.04772000
H	5.48579600	-1.41365100	-0.96632800
C	5.94970500	-0.32504000	2.29010700
H	6.77641700	-0.97202900	2.60368400
H	6.37436200	0.62898100	1.96422600
H	5.31237700	-0.13040900	3.15479300
C	4.06065200	-2.44280600	2.96382000
H	3.40434900	-3.30947800	3.08401300
H	4.93942700	-2.58628600	3.60063500
H	3.51957500	-1.55907400	3.31231100
C	5.17766000	-3.57358700	1.02892000
H	6.13690700	-3.67632600	1.54803600
H	4.57133500	-4.45392500	1.26196000
H	5.36559800	-3.56957500	-0.04611100
C	2.34252900	-0.41467900	-4.58505100
H	2.89111200	0.53017600	-4.61931000
H	2.33990200	-0.85376600	-5.58783400
H	1.31133700	-0.19045200	-4.29965700
C	1.24899200	-3.11342300	-4.32107900
H	1.84514800	-3.44283500	-5.17930100
H	0.67010900	-3.97237400	-3.96765500
H	0.54568000	-2.34888800	-4.65500400
C	2.96517900	-3.76049100	-2.60930300
H	2.27704900	-4.51924400	-2.22539100
H	3.60586500	-4.22929100	-3.36316300
H	3.58097900	-3.42203500	-1.77360900
C	4.44967900	-1.63837700	-3.99282900
H	4.98187100	-0.70016800	-4.17522300
H	4.99759900	-2.19435100	-3.22977800
H	4.46228700	-2.22001100	-4.92109300
H	-1.45875500	0.59914700	1.09174900
Si	-1.93590100	1.50064000	-1.12071100
Cl	-2.34794700	-0.28357500	-2.22587200
C	-3.65715800	2.14730500	-0.64234200

C	-3.88294000	2.67852400	0.64009500
C	-4.73101200	2.16993200	-1.55226400
C	-5.12249300	3.21341400	1.00061900
H	-3.07886100	2.67034800	1.37200900
C	-5.97185500	2.70101400	-1.19939600
H	-4.59956900	1.75371400	-2.54801900
C	-6.17060600	3.22611300	0.08000400
H	-5.26998600	3.61608200	1.99966800
H	-6.78549300	2.70325400	-1.92032600
H	-7.13708400	3.63921800	0.35688100
C	-1.17072600	2.68892300	-2.36721600
H	-1.14027500	3.69807200	-1.93838200
H	-0.14692800	2.39717500	-2.61598000
H	-1.76518100	2.73709300	-3.28487300

INT2f-ISO

Ir	1.28533700	-0.28285300	-0.39695300
N	-0.35907200	-1.55397400	0.21672700
C	-0.46758600	-1.81451200	1.55140800
C	-1.46445400	-2.67818600	2.06395500
C	-2.39759200	-3.26690100	1.16528200
C	-2.27740200	-2.96741900	-0.19090200
C	-1.23799200	-2.11417500	-0.60892100
C	0.46661800	-1.17737700	2.44114000
N	1.36684200	-0.32176900	1.88946600
C	2.25210900	0.26195100	2.68769700
C	2.31564000	0.05451700	4.08187000
C	1.38719500	-0.80328700	4.67422400
C	0.42847600	-1.43755000	3.83336800
B	1.20039800	-0.58992100	-2.41589600
O	2.28559400	-0.76057000	-3.27329200
C	1.81617500	-0.72496700	-4.64073300
C	0.30850000	-1.15376400	-4.48696200
O	0.00631200	-0.71927700	-3.13712300
B	2.46230500	-2.04090200	-0.38039300
O	3.80664600	-2.11848800	-0.03468200
C	4.18503200	-3.51147800	0.09012900
C	3.08112800	-4.23522000	-0.76727900
O	1.96743100	-3.30704200	-0.68129200
B	2.29385500	1.52411500	-0.73105500
O	2.47320000	2.15886900	-1.94783200
C	3.40225500	3.25589400	-1.76824000
C	3.26519600	3.56479400	-0.22815900
O	2.84701700	2.27660100	0.30491200
H	-1.11420300	-1.86286800	-1.65666900
H	2.93302000	0.95158500	2.19971500
C	1.37685600	-1.06704800	6.16026500
H	0.41676400	-0.78332900	6.60626800
H	1.52909300	-2.13105800	6.37507400
H	2.15781900	-0.51118500	6.67846100
C	3.38110500	0.78614800	4.86478400
H	2.94771200	1.45270800	5.61870400
H	4.05017800	0.09340600	5.38708000
H	3.99446300	1.39662800	4.19787000
C	-3.22178600	-3.50438900	-1.23998100
H	-4.24811900	-3.16491100	-1.06185800
H	-2.92759800	-3.15988900	-2.23408800
H	-3.23411100	-4.59958900	-1.25907300
C	-3.49193800	-4.16124400	1.69150700
H	-4.09093400	-4.58751900	0.88728500
H	-3.08394400	-4.99129100	2.27849700
H	-4.16736200	-3.59737800	2.34513900
C	-1.48669900	-2.91179400	3.48014200
H	-2.24315800	-3.57082400	3.89046800
C	-0.58333100	-2.33074100	4.32090600
H	-0.63402500	-2.54324800	5.38286500
C	-0.35600100	1.31203200	-0.66638500
H	2.82813400	0.02916700	-0.67597900
C	3.44992300	-4.35388800	-2.25293300

H	4.27278000	-5.05750100	-2.41440200
H	2.57806100	-4.71823900	-2.80458800
H	3.72666800	-3.38038100	-2.66538400
C	2.62596800	-5.59251600	-0.23227200
H	1.83314800	-5.99087700	-0.87250900
H	3.45183200	-6.31259400	-0.23160200
H	2.23119000	-5.51534600	0.78260000
C	5.61743600	-3.67195100	-0.41892700
H	6.29922700	-3.10626600	0.22324300
H	5.92860300	-4.72237100	-0.39953400
H	5.72545300	-3.29360400	-1.43687900
C	4.12072800	-3.85924700	1.58507900
H	4.45694000	-4.88225900	1.78237000
H	4.77056200	-3.17209100	2.13447100
H	3.10416700	-3.74490400	1.97171600
C	0.10373400	-2.67543400	-4.52924300
H	0.28190700	-3.08384400	-5.52897800
H	0.76494900	-3.17880300	-3.81904500
H	-0.93010000	-2.90508000	-4.25190500
C	-0.66557100	-0.46747300	-5.44404600
H	-1.68621600	-0.80312200	-5.23538000
H	-0.63997000	0.61743700	-5.33005500
H	-0.43561500	-0.71679700	-6.48581400
C	2.67633600	-1.67127300	-5.47871200
H	2.32314700	-1.71104500	-6.51517300
H	3.70931700	-1.31098100	-5.48899600
H	2.67813100	-2.68425600	-5.07224000
C	1.98949200	0.71634600	-5.14229900
H	1.41354000	1.41741300	-4.53429200
H	3.04379900	0.99202400	-5.05113400
H	1.69456500	0.82159700	-6.19150100
C	2.16341100	4.58354500	0.08931400
H	2.01213000	4.62504500	1.17164600
H	2.43672400	5.58717400	-0.25093300
H	1.21248700	4.30981000	-0.37381900
C	4.56244900	3.97034100	0.47161300
H	4.36954600	4.13150100	1.53679300
H	5.32996300	3.19966400	0.37991600
H	4.95487700	4.90566600	0.05813100
C	4.79133700	2.73244500	-2.16279300
H	5.10394800	1.90675600	-1.51735400
H	4.74663200	2.35454200	-3.18762900
H	5.55105600	3.51923900	-2.11715500
C	2.99102500	4.39662500	-2.69805900
H	3.62766100	5.27520800	-2.54766400
H	3.09900100	4.07629700	-3.73835600
H	1.95075800	4.68736800	-2.54197600
H	0.01590100	2.13694400	-1.27988700
Si	-1.34247200	2.05354600	0.71474800
Cl	-2.11615000	3.93718000	0.01788500
H	-1.04945300	0.77229900	-1.32113600
C	-0.45687500	2.52928900	2.31087400
H	-0.25579300	1.65383100	2.93486300
H	0.50392800	2.99563100	2.07897700
H	-1.05510600	3.23828600	2.89099100
C	-2.89583500	1.04758200	1.14220300
C	-3.76195800	0.60334100	0.12440300
C	-3.25136100	0.74718800	2.46891300
C	-4.92948800	-0.10257900	0.41674500
H	-3.52821500	0.82665800	-0.91376600
C	-4.41552900	0.03513500	2.77093700
H	-2.61643000	1.07925500	3.28600400
C	-5.25929600	-0.39057600	1.74415500
H	-5.58804800	-0.41893200	-0.38843300
H	-4.66780000	-0.17678800	3.80696900
H	-6.17327800	-0.93186500	1.97535300

TSf-ISO

Ir	0.37746600	-0.63959000	-0.25497800

N	-0.87270000	-2.37253000	0.30156800
C	-0.83058800	-2.79129500	1.59629000
C	-1.56402000	-3.91232500	2.05527000
C	-2.41673800	-4.59876900	1.14950700
C	-2.49276800	-4.11846300	-0.15690100
C	-1.69957200	-3.01419100	-0.52386700
C	-0.01994500	-2.03537800	2.50344100
N	0.59612900	-0.93261300	2.00523500
C	1.37340200	-0.24038600	2.83388100
C	1.57255600	-0.55306600	4.19297200
C	0.92623500	-1.66839100	4.72608200
C	0.10687400	-2.43563000	3.85612200
B	0.34126200	-1.01259100	-2.29666700
O	0.92909100	-0.21623200	-3.27719300
C	0.48303800	-0.65515600	-4.58102900
C	0.07568100	-2.14731600	-4.29911200
O	-0.29959300	-2.09602200	-2.89504400
B	2.13145800	-1.81631800	-0.39874900
O	3.08896300	-1.83477200	-1.41523700
C	4.22913800	-2.61533000	-0.97681300
C	3.58938400	-3.54956900	0.11340800
O	2.47668200	-2.75058400	0.58177200
B	1.60661500	1.08976900	-0.19635300
O	1.26359000	2.37448700	-0.60341800
C	2.46720300	3.17982300	-0.66234100
C	3.41133800	2.43953000	0.35843300
O	2.88879700	1.07863000	0.33557600
H	-1.71243900	-2.66007300	-1.54482000
H	1.89577600	0.60096200	2.39945500
C	1.07606000	-2.06790000	6.17326900
H	0.10958900	-2.04529400	6.68994700
H	1.46573700	-3.08806200	6.26372500
H	1.75419900	-1.40686400	6.71230100
C	2.47758400	0.33978500	5.01009000
H	1.94944100	0.78709900	5.85933800
H	3.33531300	-0.21056400	5.41254900
H	2.86786200	1.15709600	4.39979500
C	-3.37910500	-4.73553000	-1.21437900
H	-4.43689200	-4.69751800	-0.93132100
H	-3.27237100	-4.20411600	-2.16292500
H	-3.12718900	-5.78653000	-1.39467000
C	-3.21610000	-5.79051700	1.61496700
H	-3.79720100	-6.23310200	0.80623400
H	-2.56418900	-6.57181900	2.02129900
H	-3.91498300	-5.50747500	2.41066300
C	-1.41478800	-4.29589600	3.42981700
H	-1.95836400	-5.15917300	3.79614400
C	-0.61374900	-3.59966100	4.28400700
H	-0.52833600	-3.92271300	5.31519300
C	-1.55607000	0.59476200	-0.28585700
H	0.74468500	0.45876800	-1.30811700
H	-1.40420800	1.37967600	-1.03528100
C	3.00031400	-4.84518100	-0.46468500
H	3.78024000	-5.54369400	-0.78514300
H	2.39821400	-5.33397500	0.30694000
H	2.34864000	-4.63291900	-1.31661400
C	4.49017300	-3.87502500	1.30502700
H	3.94406700	-4.50090000	2.01758500
H	5.38290400	-4.42537500	0.98771400
H	4.80619400	-2.96954400	1.82639200
C	4.82734100	-3.33664200	-2.18468300
H	5.19454400	-2.59960500	-2.90524200
H	5.67284700	-3.96719900	-1.88760600
H	4.09017900	-3.96284100	-2.69045400
C	5.25397600	-1.62652900	-0.40214100
H	6.17271300	-2.12884000	-0.08185400
H	5.51276100	-0.89963900	-1.17713000
H	4.83196700	-1.07585500	0.44167000
C	1.25149500	-3.12685000	-4.40824600

H	1.57215400	-3.26098100	-5.44611600
H	2.09900000	-2.77861100	-3.81321200
H	0.93794300	-4.10063300	-4.01960100
C	-1.11938300	-2.66099100	-5.10027000
H	-1.34173700	-3.69289500	-4.81082000
H	-2.01260700	-2.05833300	-4.92495000
H	-0.90140700	-2.65487300	-6.17360600
C	1.62939400	-0.47098800	-5.57457900
H	1.35809600	-0.85422300	-6.56444100
H	1.85539400	0.59447600	-5.67666100
H	2.53577500	-0.97647900	-5.23740200
C	-0.70481200	0.23572200	-4.97353200
H	-1.53202100	0.12408100	-4.26705800
H	-0.38491700	1.28102600	-4.94996100
H	-1.06987000	0.01012100	-5.98054000
C	3.28286500	2.96254200	1.79485600
H	3.83289000	2.29608900	2.46640800
H	3.70742900	3.96576800	1.89668700
H	2.23867600	2.99825500	2.11758400
C	4.88560900	2.38813500	-0.04060100
H	5.45175800	1.82820900	0.70959900
H	5.02313400	1.89335400	-1.00296200
H	5.30848900	3.39687900	-0.09980300
C	2.96763000	3.11500100	-2.11314200
H	3.19501400	2.08724700	-2.40850500
H	2.17727000	3.48319900	-2.77262400
H	3.85819200	3.73328100	-2.26273300
C	2.10958600	4.62025100	-0.29822500
H	3.00243700	5.25470800	-0.28831000
H	1.41819600	5.02188800	-1.04413300
H	1.62345000	4.68663800	0.67677700
H	-2.31859600	-0.08263200	-0.69371700
Si	-2.22526600	1.40997400	1.21937100
C	-1.02044700	2.52758500	2.14887800
H	-0.46238700	3.12310700	1.42054200
H	-1.54033700	3.20835000	2.82924200
H	-0.30067600	1.93972300	2.72852000
C	-3.06748000	0.22255600	2.43586900
C	-3.96544800	-0.76029200	1.97855500
C	-2.85746200	0.30948700	3.82349300
C	-4.62454400	-1.61434600	2.86374100
H	-4.16350500	-0.85122200	0.91330600
C	-3.51076800	-0.54350000	4.71675200
H	-2.17910500	1.06131100	4.21881900
C	-4.39765900	-1.50847100	4.23808200
H	-5.32199500	-2.35667500	2.48328500
H	-3.33412100	-0.44895200	5.78543500
H	-4.91401400	-2.16837700	4.93049700
Cl	-3.81144500	2.76373100	0.66604100

TS2f-ISO

Ir	1.29581900	-0.33166000	-0.38562800
N	-0.38186200	-1.56551700	0.25696300
C	-0.45928800	-1.83813900	1.59093500
C	-1.44549800	-2.70218500	2.12143700
C	-2.40177200	-3.28075800	1.23940000
C	-2.31298000	-2.97162000	-0.11699700
C	-1.28043400	-2.11684100	-0.55198300
C	0.50028600	-1.21213200	2.46262500
N	1.39315900	-0.36028200	1.89496500
C	2.29748000	0.21809400	2.67336400
C	2.39221700	0.00550100	4.06535600
C	1.47508000	-0.85267000	4.67441400
C	0.49377700	-1.47985300	3.85339200
B	1.20553800	-0.64993600	-2.40112300
O	2.26931400	-0.85838500	-3.27544400
C	1.78282600	-0.79290100	-4.63585500
C	0.26123400	-1.16076400	-4.46481600
O	-0.00539800	-0.72473200	-3.10739800

B	2.47113500	-2.05155100	-0.38456100
O	3.82569500	-2.10055800	-0.07414800
C	4.23191800	-3.48397300	0.05365300
C	3.12135900	-4.23683000	-0.76951500
O	1.99192300	-3.33297700	-0.65648500
B	1.92634900	1.64819300	-0.71151800
O	2.27175300	2.23415000	-1.93746800
C	3.29246500	3.22610500	-1.70037400
C	3.02485800	3.63423400	-0.19986300
O	2.42955800	2.42738600	0.34593600
H	-1.17657200	-1.86081000	-1.60126500
H	2.96667000	0.90972400	2.17225900
C	1.49911900	-1.12450300	6.15886700
H	0.54953200	-0.84362400	6.62850800
H	1.65657900	-2.18970800	6.36379900
H	2.29183700	-0.57139400	6.66189200
C	3.47733400	0.73069400	4.82679700
H	3.06360000	1.39104200	5.59698300
H	4.15951100	0.03366500	5.32593700
H	4.07339700	1.34683900	4.14956400
C	-3.28309200	-3.49810700	-1.14763400
H	-4.30271900	-3.15098000	-0.94718500
H	-3.00688500	-3.15263000	-2.14659100
H	-3.30379900	-4.59305300	-1.17003200
C	-3.48406700	-4.17873200	1.78439300
H	-4.11316900	-4.58487600	0.99288200
H	-3.06121400	-5.02307400	2.33991200
H	-4.13338300	-3.62638300	2.47342600
C	-1.43382000	-2.94682400	3.53606000
H	-2.18071200	-3.60835300	3.95999200
C	-0.50928000	-2.37297300	4.35912500
H	-0.53499900	-2.59284700	5.42049300
C	-0.18663000	1.51910900	-0.65783500
H	2.77470100	0.15011600	-0.69322100
C	3.45227500	-4.35473900	-2.26428400
H	4.28356700	-5.04356500	-2.44544500
H	2.57239400	-4.73758400	-2.79039300
H	3.69805000	-3.37748100	-2.68697400
C	2.71100300	-5.60140000	-0.21635100
H	1.91150700	-6.02113900	-0.83438900
H	3.55236600	-6.30316600	-0.23250200
H	2.33989600	-5.52738400	0.80775000
C	5.65409800	-3.62440900	-0.48885600
H	6.34162800	-3.04088100	0.13085500
H	5.98410800	-4.66911300	-0.46800100
H	5.72957200	-3.25344000	-1.51236500
C	4.21054300	-3.82058500	1.55267200
H	4.57168000	-4.83510700	1.75016800
H	4.85964300	-3.11614300	2.08064600
H	3.20144600	-3.72341800	1.96297500
C	-0.00481600	-2.67280500	-4.51368000
H	0.14046300	-3.08083500	-5.51885700
H	0.64818700	-3.20697400	-3.81830900
H	-1.04226500	-2.86303000	-4.22024700
C	-0.69636400	-0.42911600	-5.40447400
H	-1.72706400	-0.72598200	-5.18617900
H	-0.62538000	0.65296200	-5.28268100
H	-0.48886500	-0.67856700	-6.45087800
C	2.59113800	-1.76667300	-5.49390200
H	2.22223400	-1.78202500	-6.52556500
H	3.63786600	-1.44925300	-5.51576400
H	2.55697800	-2.78267400	-5.09644000
C	2.00921400	0.64465000	-5.12760100
H	1.48390900	1.36480200	-4.49639100
H	3.07736800	0.87046200	-5.06201600
H	1.69338200	0.77483300	-6.16791800
C	2.00719100	4.77282400	-0.05099800
H	1.74027600	4.88063100	1.00418900
H	2.41928400	5.72708300	-0.39404200

H	1.08861000	4.57438200	-0.60747900
C	4.27881600	3.95793900	0.61358900
H	3.99733000	4.20091900	1.64311900
H	4.97057100	3.11394600	0.64015000
H	4.80295300	4.82476200	0.19694500
C	4.65396900	2.54274600	-1.90982400
H	4.80458900	1.72733400	-1.19692600
H	4.67859800	2.11184800	-2.91422600
H	5.48552300	3.24859200	-1.81506400
C	3.11813600	4.35427800	-2.71789400
H	3.83470200	5.16327500	-2.53773700
H	3.29479200	3.96645000	-3.72544900
H	2.10866800	4.76858700	-2.68902700
H	0.06653200	2.39274000	-1.26402800
Si	-1.23074600	2.14723700	0.75196000
Cl	-1.97282800	4.06792300	0.14947100
H	-0.81065100	0.92694000	-1.33424200
C	-0.39305200	2.48878800	2.40348500
H	-0.28421000	1.57420800	2.99330400
H	0.60743900	2.88907500	2.22400500
H	-0.96723900	3.21199100	2.98986100
C	-2.79899600	1.11727500	1.02274800
C	-3.60279300	0.73220100	-0.06754900
C	-3.23911200	0.76103700	2.30963000
C	-4.79358100	0.02924100	0.11825400
H	-3.30227700	0.99999700	-1.07768500
C	-4.42784300	0.05247600	2.50419700
H	-2.65286900	1.04600800	3.17914300
C	-5.20921100	-0.31411500	1.40772500
H	-5.40262800	-0.24176400	-0.74044600
H	-4.74776900	-0.20281000	3.51131600
H	-6.14141100	-0.85317900	1.55610700

TS2f

Ir	1.60719600	-0.22723000	-0.09019700
N	-0.24898700	-1.47878100	0.23656500
C	-0.58149800	-1.67510000	1.53891500
C	-1.68693100	-2.47592000	1.91403300
C	-2.46028800	-3.10298400	0.89476900
C	-2.09319300	-2.89368500	-0.43336900
C	-0.97802200	-2.07153500	-0.69583000
C	0.22930300	-1.03476400	2.54521600
N	1.29130900	-0.29450400	2.12704200
C	2.02050900	0.33328900	3.04062200
C	1.77796400	0.26634900	4.42907100
C	0.71157200	-0.50779900	4.88447100
C	-0.09154500	-1.17766700	3.91729000
B	3.65846800	0.06918400	0.00196000
O	4.54971900	0.57961200	-0.93722100
C	5.91218600	0.35572300	-0.49936100
C	5.73902200	0.12459100	1.05233700
O	4.36363800	-0.31210000	1.15448700
B	2.49958200	-2.12892400	-0.23425400
O	3.48316200	-2.62953100	-1.08133300
C	3.70663900	-4.03603100	-0.79436100
C	2.38562400	-4.44004000	-0.04217500
O	1.95995000	-3.17726800	0.51923700
B	1.92692200	1.86734400	-0.47837100
O	1.83127000	2.50967200	-1.72581600
C	2.60527500	3.73254100	-1.71849400
C	2.78290000	4.01675100	-0.17305900
O	2.62619300	2.70308000	0.41922200
H	-0.66909000	-1.89385100	-1.72016200
H	2.84967700	0.91349800	2.65637100
C	0.39211200	-0.64230000	6.35329400
H	-0.61267200	-0.26514200	6.57606000
H	0.41965700	-1.69113200	6.66966900
H	1.09674400	-0.09118700	6.97550700
C	2.69458400	1.03701200	5.35063700

H	2.14711900	1.77579700	5.94647700
H	3.21786900	0.37586800	6.05035600
H	3.45223500	1.57375500	4.77497000
C	-2.84197900	-3.48347500	-1.60455800
H	-3.87943700	-3.13395700	-1.63393700
H	-2.37346100	-3.19100200	-2.54719700
H	-2.86171300	-4.57821300	-1.56947600
C	-3.64751600	-3.95587200	1.26835500
H	-4.12000300	-4.40273400	0.39413100
H	-3.35562600	-4.76928300	1.94182600
H	-4.40793500	-3.36222500	1.78875200
C	-1.97616200	-2.61262400	3.31279500
H	-2.82083400	-3.21873900	3.61982700
C	-1.22054200	-1.99276300	4.26406400
H	-1.47904900	-2.11788500	5.30946400
C	0.03502000	1.55320200	-0.02933800
H	1.71797300	-0.27239200	-1.66149600
H	-0.49634400	1.01309400	0.75574400
C	1.27472300	-4.92085700	-0.98850300
H	1.49870500	-5.90546200	-1.41113000
H	0.33701700	-4.99198500	-0.43020000
H	1.12690600	-4.21645600	-1.81168800
C	2.55457600	-5.44284200	1.10007600
H	1.58279100	-5.62861900	1.56764300
H	2.94094500	-6.40045000	0.73415700
H	3.22910200	-5.06406400	1.86972100
C	3.95481700	-4.75639600	-2.12023100
H	4.87249900	-4.37386100	-2.57621500
H	4.07787800	-5.83419900	-1.96601500
H	3.13976900	-4.59436200	-2.82762200
C	4.94851400	-4.15607800	0.09891100
H	5.15886300	-5.20011500	0.35085000
H	5.81913200	-3.75938900	-0.42865400
H	4.82024400	-3.59018800	1.02464600
C	5.86065100	1.40864800	1.88721200
H	6.87904400	1.81001500	1.88222300
H	5.17251200	2.17478100	1.52488600
H	5.59636700	1.17760600	2.92446500
C	6.64496300	-0.95449700	1.64638000
H	6.41243200	-1.08574800	2.70783200
H	6.50936800	-1.91493000	1.14911500
H	7.69912200	-0.66674900	1.56611200
C	6.78294700	1.56006000	-0.86534500
H	7.81536800	1.39533600	-0.53715100
H	6.79480600	1.69050800	-1.95107800
H	6.42814900	2.48768100	-0.41590200
C	6.42704000	-0.86875200	-1.26833400
H	5.77681300	-1.72646900	-1.10497200
H	6.40472000	-0.64105100	-2.33812500
H	7.45603500	-1.12277600	-0.99324600
C	1.69515600	4.92893800	0.41867200
H	1.77319500	4.90469200	1.51001200
H	1.81802000	5.96599100	0.09056000
H	0.68855100	4.61067400	0.14094500
C	4.14977700	4.57924100	0.21719500
H	4.20491100	4.69742600	1.30406100
H	4.95996400	3.92290900	-0.09808100
H	4.30862700	5.56390500	-0.23585600
C	3.91598500	3.44358300	-2.46186600
H	4.45179000	2.61872300	-1.99541500
H	3.67259100	3.14270600	-3.48540900
H	4.55947800	4.32839400	-2.51215900
C	1.83469000	4.81727200	-2.47553700
H	2.39613000	5.75845200	-2.47127200
H	1.70776100	4.51150500	-3.51837900
H	0.84465600	5.00092000	-2.05870900
H	0.18934600	2.53741600	0.42348200
Si	-1.10339200	1.77202500	-1.51208600
Cl	-2.92426100	0.73109500	-1.01873100

C	-0.58754400	1.04883400	-3.16568600
H	0.31345900	1.55916000	-3.51351800
H	-0.33944100	-0.00996600	-3.06171100
H	-1.38504000	1.14623200	-3.90883600
C	-1.68779900	3.55863200	-1.72130400
C	-2.04792000	4.33503400	-0.60359000
C	-1.81830900	4.14832600	-2.99090700
C	-2.51091000	5.64392000	-0.74483700
H	-1.97402500	3.90920000	0.39467000
C	-2.28063800	5.45787700	-3.14041000
H	-1.55194800	3.58251600	-3.87928300
C	-2.62635900	6.20957500	-2.01662800
H	-2.78273100	6.22135600	0.13483000
H	-2.36961400	5.89063100	-4.13337200
H	-2.98555500	7.22882600	-2.13037100

TS5

Ir	0.39133300	-0.63515000	-0.07020800
N	-0.85185800	-2.54083100	0.38609400
C	-0.84756800	-2.89646400	1.69665100
C	-1.60206000	-3.99389500	2.18080900
C	-2.38539400	-4.74867900	1.26196400
C	-2.35817000	-4.37726500	-0.08167600
C	-1.57245400	-3.26751800	-0.46013500
C	-0.03121000	-2.12311500	2.59925400
N	0.68684300	-1.09103800	2.08459900
C	1.47979000	-0.40335400	2.89584000
C	1.60239200	-0.65131400	4.27802400
C	0.85758800	-1.68842800	4.83739700
C	0.02345500	-2.45501800	3.97524700
B	0.40044600	-0.77463600	-2.14340500
O	1.14561400	-0.04251300	-3.05058800
C	0.67180900	-0.31070200	-4.38873900
C	-0.04571000	-1.70194200	-4.21983900
O	-0.44064200	-1.66521900	-2.82404400
B	2.12440500	-1.83833900	-0.36653700
O	2.89978700	-2.02845600	-1.51000500
C	4.11096400	-2.73800500	-1.14944600
C	3.67736900	-3.47989600	0.16568500
O	2.64162000	-2.60933400	0.67800200
B	1.65765800	0.95509400	-0.23813200
O	1.37114000	2.16932600	-0.85796200
C	2.57826400	2.95532000	-0.95537400
C	3.45179000	2.37306300	0.21939600
O	2.93494000	1.02485900	0.34900500
H	-1.51938700	-2.95216500	-1.49794900
H	2.06911600	0.37341500	2.42196400
C	0.92344100	-2.01454900	6.30935200
H	-0.06511900	-1.93614600	6.77577100
H	1.27598600	-3.03939900	6.47266200
H	1.59542400	-1.34505300	6.84567800
C	2.53805300	0.22269300	5.08036500
H	2.01067800	0.76412400	5.87360000
H	3.33399000	-0.36115400	5.55574200
H	3.01441700	0.96568100	4.43638900
C	-3.13853000	-5.10006100	-1.15504500
H	-4.21656200	-5.07218400	-0.96156300
H	-2.96560000	-4.63890300	-2.13050800
H	-2.84805900	-6.15386900	-1.23134500
C	-3.21720900	-5.90727500	1.75408700
H	-3.72076600	-6.42382200	0.93729700
H	-2.60158600	-6.64307700	2.28321900
H	-3.98710700	-5.56307000	2.45412900
C	-1.54199000	-4.29008500	3.58332600
H	-2.12767900	-5.11445700	3.97351000
C	-0.76911800	-3.55921800	4.43556600
H	-0.75112400	-3.81803300	5.48803200
H	-0.60022000	0.33273100	-0.89488800
C	3.02610900	-4.84725400	-0.09291200

H	3.75595600	-5.59496600	-0.42005800
H	2.56567500	-5.19999200	0.83464000
H	2.24140400	-4.76960500	-0.85056500
C	4.76878700	-3.61598500	1.22763600
H	4.36012100	-4.11384100	2.11241500
H	5.60630800	-4.21826400	0.85828000
H	5.14983100	-2.64072100	1.53598400
C	4.51349900	-3.64889200	-2.30903700
H	4.75466200	-3.04077400	-3.18618000
H	5.40124300	-4.23886200	-2.05501700
H	3.70947200	-4.33392500	-2.58480900
C	5.19478200	-1.67519900	-0.91744800
H	6.16476900	-2.12419100	-0.67996100
H	5.30505000	-1.08332000	-1.83079300
H	4.90500600	-0.99541600	-0.11284500
C	0.90229400	-2.89767100	-4.38860400
H	1.21827400	-3.02266900	-5.42932300
H	1.78451300	-2.77970700	-3.75586400
H	0.38087200	-3.80935700	-4.08020700
C	-1.29740000	-1.89806200	-5.07483000
H	-1.73698500	-2.87892100	-4.86689000
H	-2.05333500	-1.13860300	-4.86646900
H	-1.05177300	-1.86262700	-6.14183300
C	1.87089000	-0.31284400	-5.33700800
H	1.56982000	-0.58556200	-6.35465400
H	2.31054700	0.68827700	-5.37253400
H	2.64244100	-1.00677500	-4.99921500
C	-0.28966200	0.82805400	-4.76198100
H	-1.15303000	0.85321400	-4.09127400
H	0.23792400	1.78022500	-4.65975800
H	-0.64890600	0.74004800	-5.79254900
C	3.21916900	3.08379400	1.56173900
H	3.72476700	2.52183600	2.35380100
H	3.62291900	4.10102600	1.56084100
H	2.15428000	3.13556800	1.80574300
C	4.95227000	2.29612400	-0.06380400
H	5.46747800	1.84919900	0.79229200
H	5.16225800	1.68213600	-0.94065100
H	5.37302600	3.29472100	-0.22598900
C	3.17858100	2.69418100	-2.34494700
H	3.40290100	1.63425900	-2.48297400
H	2.43959200	2.97215100	-3.10117600
H	4.08567700	3.28372700	-2.51405900
C	2.20937500	4.43321800	-0.82026700
H	3.10254200	5.06754800	-0.84136400
H	1.56855700	4.72504900	-1.65734000
H	1.66192900	4.63198100	0.10322900
C	-3.76241400	0.68500100	1.21644000
C	-3.53774000	1.92064700	1.84800400
C	-2.26871600	2.49823900	1.82072900
C	-1.20942200	1.84677800	1.18480100
C	-1.37482500	0.58615200	0.58384100
C	-2.67213400	0.04986900	0.58731400
H	-0.24191100	2.33500700	1.13940200
H	-2.84330400	-0.89992500	0.08840600
H	-4.35139700	2.44242200	2.34572000
H	-2.10418000	3.46639700	2.28865600
Si	-5.45998500	-0.09372300	1.17625700
C	-6.80417700	1.07790400	1.77610900
H	-6.85615200	1.96647700	1.13686900
H	-7.78045000	0.58378100	1.75034800
H	-6.62472700	1.40783000	2.80365500
C	-5.87363600	-0.78421700	-0.52481800
H	-5.90819400	0.02727400	-1.26063400
H	-5.12196500	-1.50565400	-0.85868400
H	-6.84662100	-1.28558200	-0.52047900
Cl	-5.50404400	-1.75853900	2.49863000

INT6

Ir	1.00830100	-0.29549200	-0.49450600
N	-0.22388700	-2.17755200	0.07565600
C	-0.70478700	-2.13126100	1.34332700
C	-1.58910800	-3.11332200	1.85302900
C	-2.00790300	-4.17027000	0.99539700
C	-1.50488200	-4.19638100	-0.30393000
C	-0.61111300	-3.17855700	-0.70281600
C	-0.28288100	-1.03322500	2.17201300
N	0.54468900	-0.09767100	1.62285200
C	0.94881500	0.91560900	2.38575000
C	0.56400300	1.09123600	3.73045200
C	-0.28891100	0.15593200	4.31313900
C	-0.72596300	-0.93867400	3.51403900
B	2.88434300	-1.00376600	0.32960100
O	3.25435300	-2.30907000	0.63960600
C	4.55339700	-2.30127900	1.29157700
C	4.67241700	-0.81282300	1.79540600
O	3.78328600	-0.10639500	0.89546900
B	2.19431500	-1.30258900	-1.89879000
O	3.44398300	-0.97805900	-2.41438900
C	3.64348000	-1.72414500	-3.64042600
C	2.70637500	-2.97397700	-3.43019300
O	1.69247500	-2.44017900	-2.54525600
B	1.90489500	1.50198600	-0.86028700
O	2.06790800	2.51790800	0.09111200
C	2.53276700	3.72022300	-0.57418700
C	3.16205800	3.13545100	-1.89199600
O	2.38724500	1.93311400	-2.09016500
H	-0.17859900	-3.18552500	-1.69701700
H	1.59996000	1.63176300	1.89817700
C	-0.75371900	0.27915700	5.74324000
H	-1.84558700	0.35692600	5.79722500
H	-0.46459600	-0.59955000	6.33091800
H	-0.33498300	1.15748700	6.23377500
C	1.09795900	2.29785800	4.46765000
H	0.29029500	2.94614100	4.82510500
H	1.69624900	2.01101600	5.33973200
H	1.73485800	2.89580500	3.81172100
C	-1.88065400	-5.25333900	-1.31517600
H	-2.94808100	-5.21151600	-1.55837200
H	-1.32706600	-5.10614800	-2.24557100
H	-1.66068900	-6.26388400	-0.95422200
C	-2.97496300	-5.21259500	1.50053900
H	-3.21561600	-5.94944700	0.73487000
H	-2.56532600	-5.74998700	2.36363200
H	-3.91559000	-4.75156400	1.82192200
C	-2.02606500	-2.98674200	3.21312000
H	-2.70287600	-3.72888700	3.62119600
C	-1.61122600	-1.95635500	4.00401800
H	-1.96393100	-1.89850500	5.02729600
C	2.01953700	-3.48288700	-4.69856200
H	2.75673800	-3.83514800	-5.42823400
H	1.36545600	-4.32496400	-4.45162300
H	1.40997800	-2.70807300	-5.16665100
C	3.38247800	-4.14095500	-2.69947500
H	2.62450900	-4.89060000	-2.45332700
H	4.14838200	-4.62003500	-3.31752800
H	3.83279800	-3.80417100	-1.76488100
C	3.19318700	-0.81233200	-4.79182000
H	3.75803600	0.12186300	-4.74028700
H	3.36795300	-1.27274500	-5.76948400
H	2.13295600	-0.55997400	-4.70698300
C	5.12978200	-2.04559300	-3.78670600
H	5.31257600	-2.63987400	-4.68863200
H	5.69819600	-1.11506200	-3.87531100
H	5.51309400	-2.59583800	-2.92602500
C	5.60453100	-2.65962000	0.23326100
H	5.39162300	-3.65693600	-0.16159100
H	5.57758200	-1.94951900	-0.59641900

H	6.61384800	-2.67402200	0.65609900
C	4.54312400	-3.36563200	2.38948600
H	4.40991000	-4.35282600	1.93740400
H	5.48935800	-3.37222100	2.94181900
H	3.72734100	-3.20989300	3.09785000
C	6.06499400	-0.19078700	1.68131900
H	6.02761600	0.84821100	2.02209900
H	6.78987000	-0.72446700	2.30583800
H	6.42322500	-0.19201500	0.65056200
C	4.13632900	-0.60622900	3.21978600
H	4.78934600	-1.06459200	3.96930700
H	4.08103000	0.46681800	3.42395100
H	3.13210100	-1.02259600	3.33308600
C	1.30445200	4.60555600	-0.82729900
H	0.81377900	4.81622000	0.12783600
H	1.57978700	5.56064100	-1.28614700
H	0.58012600	4.10049200	-1.47109800
C	3.51266700	4.43932500	0.35308000
H	3.94619200	5.31749300	-0.13806200
H	2.98713500	4.78454100	1.24949400
H	4.32333000	3.78023800	0.66883700
C	3.01650300	4.01931000	-3.13010300
H	3.53951900	4.97324200	-2.99902400
H	3.45464800	3.51071400	-3.99406100
H	1.96845800	4.22278100	-3.35753700
C	4.62601500	2.70159200	-1.71991300
H	4.92395700	2.12408000	-2.59916100
H	5.29919900	3.55986500	-1.62207800
H	4.73897600	2.05494100	-0.84597500
C	-3.15204600	0.59961900	-1.86558300
C	-3.51278700	1.76622900	-1.16800700
C	-2.59185100	2.37754700	-0.31877100
C	-1.31701700	1.82892500	-0.14701800
C	-0.90937400	0.65267700	-0.80403800
C	-1.85692300	0.07759100	-1.67045400
H	-0.61878400	2.34102800	0.51082400
H	0.97920700	-0.24189000	-2.09120200
H	-1.58222400	-0.82585800	-2.21256700
H	-4.50237700	2.20052500	-1.28836000
H	-2.86611700	3.28768300	0.21132900
Si	-4.33434500	-0.23587700	-3.04246200
C	-5.93908100	0.72278000	-3.26251400
H	-5.74385400	1.71221600	-3.69150900
H	-6.61480800	0.18832000	-3.93748500
H	-6.45833600	0.86184700	-2.30963900
C	-3.53497800	-0.60613800	-4.70529500
H	-3.26816400	0.33144200	-5.20643800
H	-2.61848100	-1.19141400	-4.58496100
H	-4.21272900	-1.16472000	-5.35849900
Cl	-4.89625700	-2.13064200	-2.24641900

TS6

Ir	1.65167200	-0.18570500	-0.09853900
N	-0.23380400	-1.36021000	0.14766600
C	-0.66214900	-1.51687900	1.42468500
C	-1.84439400	-2.23311400	1.73032400
C	-2.60431100	-2.79361000	0.66449100
C	-2.14461600	-2.61304900	-0.64082400
C	-0.95167100	-1.88790100	-0.83400200
C	0.12239000	-0.91442700	2.47212700
N	1.23430300	-0.21981700	2.11097700
C	1.94892300	0.35939100	3.06730600
C	1.63567200	0.29438700	4.44157200
C	0.50520000	-0.42043300	4.83618900
C	-0.27975600	-1.04221800	3.82368500
B	3.71573200	-0.05287200	0.11370900
O	4.69057300	0.27707800	-0.82190700
C	6.00374000	0.03975700	-0.25977000
C	5.71953500	0.06983900	1.28994600

O	4.32467500	-0.31111800	1.35323200
B	2.48063000	-2.14285600	-0.07602000
O	3.33571900	-2.76059600	-0.98269100
C	3.22975700	-4.19771400	-0.82197300
C	2.71761400	-4.33135300	0.65888900
O	2.01378000	-3.08027500	0.84681800
B	2.23201600	1.76133300	-0.70319400
O	2.26748800	2.17486900	-2.02986000
C	2.93665100	3.44576800	-2.13699000
C	2.97269200	3.95764300	-0.63518200
O	2.79772100	2.73688800	0.12889300
H	-0.56787900	-1.72956700	-1.83598900
H	2.82932900	0.89150900	2.73054100
C	0.10279900	-0.54271100	6.28565100
H	-0.89542900	-0.12370100	6.45689800
H	0.06925000	-1.59225700	6.59950100
H	0.79640800	-0.02305200	6.94624700
C	2.54762200	1.00463300	5.41490300
H	2.01478800	1.76877400	5.99180600
H	2.99822800	0.30921600	6.13183900
H	3.36194300	1.50192000	4.88265900
C	-2.86620600	-3.14524300	-1.85606700
H	-3.85207400	-2.68271000	-1.97106600
H	-2.30124000	-2.92658600	-2.76501700
H	-3.00834300	-4.23007400	-1.80461400
C	-3.87551000	-3.54994800	0.96251800
H	-4.59234400	-2.91778000	1.49835200
H	-4.36269800	-3.90167900	0.05378600
H	-3.67859300	-4.42301900	1.59537700
C	-2.21815600	-2.35317100	3.11088200
H	-3.11544500	-2.90425400	3.36864100
C	-1.47331100	-1.78965100	4.10454800
H	-1.79308300	-1.90353100	5.13430700
H	1.81280200	-0.33687200	-1.65548300
C	1.74242000	-5.48222300	0.90885400
H	2.21471200	-6.45023900	0.70819300
H	1.42508300	-5.47355000	1.95609000
H	0.84924600	-5.39498900	0.28729400
C	3.84742500	-4.37222900	1.69742200
H	3.41197400	-4.27236700	2.69585000
H	4.40671500	-5.31258600	1.65841000
H	4.54120100	-3.54033100	1.55401600
C	2.20901600	-4.68320600	-1.86342900
H	2.54182000	-4.36650900	-2.85566000
H	2.11060500	-5.77352900	-1.86259100
H	1.22368200	-4.24433400	-1.68335500
C	4.58931100	-4.83587900	-1.10425000
H	4.54391200	-5.92272000	-0.97309600
H	4.88224100	-4.63215700	-2.13830500
H	5.36968100	-4.44232600	-0.45119400
C	5.82908900	1.47167200	1.90840200
H	6.85978700	1.84016900	1.91450300
H	5.19574900	2.18267700	1.37346200
H	5.48473900	1.42689600	2.94700400
C	6.53528400	-0.92347700	2.11804000
H	6.22071400	-0.87445500	3.16508500
H	6.39654200	-1.94938100	1.77308100
H	7.60375300	-0.68490300	2.07582800
C	6.96851600	1.11864200	-0.75396600
H	7.96443300	0.97503600	-0.32017300
H	7.06412300	1.05499000	-1.84174300
H	6.62707300	2.12383200	-0.50236600
C	6.47192600	-1.32826000	-0.77405500
H	5.78034600	-2.11508300	-0.47176700
H	6.48099500	-1.30534000	-1.86729000
H	7.48048300	-1.57126900	-0.42392200
C	1.81080000	4.89592300	-0.27332700
H	1.82593600	5.07574700	0.80598400
H	1.90031200	5.86166100	-0.78118200

H	0.84316100	4.45709600	-0.52250700
C	4.28909700	4.61254600	-0.21344700
H	4.24526000	4.87331700	0.84863400
H	5.14031500	3.94757900	-0.36557700
H	4.46671700	5.53397100	-0.77881400
C	4.32056600	3.17451000	-2.74228100
H	4.87745800	2.46784600	-2.12758400
H	4.18508700	2.71664800	-3.72653300
H	4.90237900	4.09370000	-2.86911600
C	2.13002700	4.33146300	-3.09222700
H	2.58098800	5.32546600	-3.18757500
H	2.11744400	3.87096400	-4.08460800
H	1.09636300	4.44654000	-2.76129200
C	-1.82604900	2.19338800	-1.54804200
C	-2.28065300	2.87955100	-0.40434800
C	-1.51246800	2.88917700	0.75796700
C	-0.29648400	2.19973800	0.80330800
C	0.19003600	1.47879600	-0.30089600
C	-0.59167600	1.52299400	-1.47112500
H	0.29047500	2.24162300	1.71556200
H	-0.20384800	1.02284000	-2.35363500
H	-3.22661100	3.41588600	-0.42351600
Si	-2.81984700	2.17622700	-3.13087800
C	-3.97422600	3.65611400	-3.26593700
H	-3.39811500	4.58798700	-3.29303400
H	-4.56911800	3.59645800	-4.18269900
H	-4.66818300	3.71123700	-2.42184200
C	-1.72976800	2.02195700	-4.65378800
H	-2.33705100	1.98856400	-5.56374500
H	-1.05273000	2.88073300	-4.72243600
H	-1.11600300	1.11721300	-4.62470600
Cl	-4.08344400	0.46112400	-3.13992400
H	-1.85756400	3.43826200	1.63142800
